# Reef benthos of Seychelles - A field guide

**DOI:** 10.3897/BDJ.9.e65970

**Published:** 2021-08-27

**Authors:** Nico Fassbender, Paris V Stefanoudis, Zoleka Nontlantla Filander, Gilberte Gendron, Christopher L Mah, Lydiane Mattio, Jeanne A Mortimer, Carlos J Moura, Toufiek Samaai, Kaveh Samimi-Namin, Daniel Wagner, Rowana Walton, Lucy C Woodall

**Affiliations:** 1 Nekton Foundation, Oxford, United Kingdom Nekton Foundation Oxford United Kingdom; 2 Department of Zoology, University of Oxford, Oxford, United Kingdom Department of Zoology, University of Oxford Oxford United Kingdom; 3 Department of Forestry, Fisheries and Environment, Branch Oceans and Coasts, Cape Town, South Africa Department of Forestry, Fisheries and Environment, Branch Oceans and Coasts Cape Town South Africa; 4 Sustainable Ocean Seychelles, Victoria, Seychelles Sustainable Ocean Seychelles Victoria Seychelles; 5 Smithsonian Institution National Museum of Natural History, Washington, United States of America Smithsonian Institution National Museum of Natural History Washington United States of America; 6 University of Cape Town, Rondebosch, Cape Town, South Africa University of Cape Town Rondebosch, Cape Town South Africa; 7 blue[c]weed, Brest, France blue[c]weed Brest France; 8 Seychelles’ Conservation & Climate Adaptation Trust (SeyCCAT), Victoria, Mahé, Seychelles Seychelles’ Conservation & Climate Adaptation Trust (SeyCCAT) Victoria, Mahé Seychelles; 9 Department of Biology, University of Florida, Gainesville, Florida, United States of America Department of Biology, University of Florida Gainesville, Florida United States of America; 10 Island Conservation Society (ICS), Point Larue, Mahé, Seychelles Island Conservation Society (ICS) Point Larue, Mahé Seychelles; 11 OKEANOS / DOP, University of the Azores, Horta, Portugal OKEANOS / DOP, University of the Azores Horta Portugal; 12 iZiko Museums of South Africa, Cape Town, South Africa iZiko Museums of South Africa Cape Town South Africa; 13 University of the Western Cape, Bellville, Cape Town, South Africa University of the Western Cape Bellville, Cape Town South Africa; 14 Naturalis Biodiversity Center, Leiden, Netherlands Naturalis Biodiversity Center Leiden Netherlands; 15 Conservation International, Arlington, United States of America Conservation International Arlington United States of America; 16 James Michel Blue Economy Research Institute, University of Seychelles, Anse Royale, Mahé, Seychelles James Michel Blue Economy Research Institute, University of Seychelles Anse Royale, Mahé Seychelles

**Keywords:** coral reefs, mesophotic coral ecosystems, benthos, morphotype, Seychelles, Indian Ocean

## Abstract

**Background:**

During the 2019 First Descent: Seychelles Expedition, shallow and deep reef ecosystems of the Seychelles Outer Islands were studied by deploying a variety of underwater technologies to survey their benthic flora and fauna. Submersibles, remotely operated vehicles (ROVs) and SCUBA diving teams used stereo-video camera systems to record benthic communities during transect surveys conducted at 10 m, 30 m, 60 m, 120 m, 250 m and 350 m depths. In total, ~ 45 h of video footage was collected during benthic transect surveys, which was subsequently processed using annotation software in order to assess reef biodiversity and community composition. Here, we present a photographic guide for the visual identification of the marine macrophytes, corals, sponges and other common invertebrates that inhabit Seychelles’ reefs. It is hoped that the resulting guide will aid marine biologists, conservationists, managers, divers and naturalists with the coarse identification of organisms as seen in underwater footage or live in the field.

**New information:**

A total of 184 morphotypes (= morphologically similar individuals) were identified belonging to Octocorallia (47), Porifera (35), Scleractinia (32), Asteroidea (19), Echinoidea (10), Actiniaria (9), Chlorophyta (8), Antipatharia (6), Hydrozoa (6), Holothuroidea (5), Mollusca (2), Rhodophyta (2), Tracheophyta (2), Annelida (1), Crinoidea (1), Ctenophora (1), Ochrophyta (1) and Zoantharia (1). Out of these, we identified one to phylum level, eight to class, 14 to order, 27 to family, 110 to genus and 24 to species. This represents the first attempt to catalogue the benthic diversity from shallow reefs and up to 350 m depth in Seychelles.

## Introduction

Coral reef ecosystems are some of the most diverse hotspots for life on our planet. Both shallow and deep water coral reefs are valued for their incredible diversity and species richness, yet little is known about the processes and functions of mesophotic coral ecosystems (MCEs; reefs ~ 30–150 m depth, as proposed by Rocha et al. 2018) and rariphotic reefscapes (~ 150–300 m depth, as proposed by Baldwin et al. 2018). While shallow and deeper coral reef ecosystems exist in close proximity to one another, the different conditions to which they are exposed have led to the formation of distinct forms of life and ecosystem functions within them (Holstein et al. 2019, Stefanoudis et al. 2019).

Deeper coral ecosystems provide a number of ecosystem services to support their shallow-water (< 30 m depth) counterparts and oceanic processes, harbouring unique assemblages of life and preserving biodiversity by supporting shallow reef systems (Cacciapaglia and van Woesik 2015, Muir et al. 2018, Weiss 2017). In times of severe disturbance to shallow-water coral reef ecosystems, deeper reefs have been proposed to act as refugia (Semmler et al. 2016, Smith et al. 2014), promoting the recovery of heavily impacted shallow reefs (Holstein et al. 2016). With their depth shielding them from most bleaching and hurricane events (Baird et al. 2018, Bridge et al. 2014), they are a crucial source of larval supply for many shallow-water coral reef systems (Holstein et al. 2016, Hughes et al. 2017, Norström et al. 2016). However, even deeper reefs have been found to be affected by various disturbances (Smith et al. 2019), including climate change (Rocha et al. 2018), ocean acidification (Cerrano et al. 2013) and invasive species (Andradi-Brown et al. 2016). To sustain these services in the future, mesophotic and rariphotic ecosystems should, where present, be incorporated into marine spatial planning initiatives to ensure their protection and sustainable management (Bridge et al. 2013).

To effectively protect and manage an ecosystem, it also needs to be documented and monitored. Knowledge of mesophotic and rariphotic reefscapes in Seychelles waters remains poorly known. Gaining a better understanding of deeper reef ecosystems and their communities was identified as a crucial step towards effectively protecting 30% of Seychelles’ waters as part of the Seychelles Marine Spatial Planning Initiative (2020). To effectively do so, the organisms living in those environments need to be documented, assessed and classified. This Field Identification Guide marks an important first step towards surveying and understanding these systems, encompassing benthic organisms of a variety of taxa that occur beyond depths accessible by SCUBA divers. This open-access Field ID Guide will be a valuable tool for scientists studying shallow, mesophotic and rariphotic coral reef environments in the Seychelles in the future.

## Materials and methods

**Figure 1.**
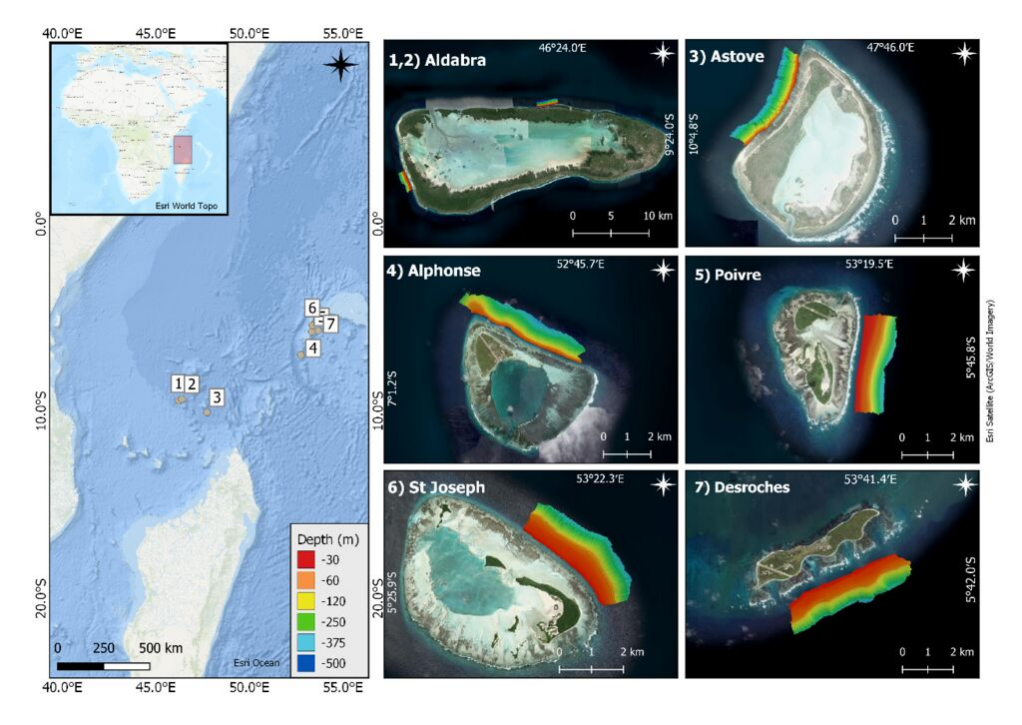
Map of the seven surveyed sites around Seychelles Outer Islands. Multibeam data were overlaid to show survey areas. Sites are listed from west to east. Survey site mean coordinates: 1) Aldabra West 1 (9°26'50.5284''S, 46°12'49.3632''E) 2) Aldabra North 1 (9°21'57.8844''S, 46°22'43.1148''E) 3) Astove West 1 (10°4'25.1652''S, 47°43'58.2024''E) 4) Alphonse North 1 (6°59'58.1424''S, 52°43'44.3856''E) 5) Poivre East 1 (5°45'52.236''S, 53°19'3.6732''E) 6) St. Joseph* North 1 (5°25'20.9316''S, 53°21'30.672''E) 7) Desroches South 1 (5°41'41.7444''S, 53°40'35.67''E) *St. Joseph was hereafter referred to as D'Arros due to initial naming when compiling datasets and the islands close proximity to one another. Maps were created in ESRI using the basemaps "World Topographic Map", "Ocean Basemap" and "World Imagery".

The Seychelles consists of 115 islands located in the Western Indian Ocean, between 480 to 1600 km from the African coast (Fig. 1).

The multidisciplinary First Descent: Seychelles Expedition, from which images in this guide were drawn, provided an opportunity to understand patterns of diversity and connectivity between the various shallow and deep reef marine ecosystems.

During the expedition, benthic and fish communities were surveyed across seven sites around Seychelles Outer Islands (Fig. 1). The fieldwork was conducted between March to April 2019 onboard the vessel *Ocean Zephyr*. SCUBA divers, remotely operated vehicles (ROVs) and submersibles were deployed to conduct transect surveys and specimen collections at ~10, 30, 60, 120, 250 and 350 m. The shallowest dives at a depth of 10 m were conducted primarily by SCUBA divers (occasionally mini-ROVs). Dives between 30–250 m were conducted primarily by submersibles, whilst an ROV was used for some dives at 250–350 m. Paralenz cameras were used for all dives, recording with a minimum resolution of 1920 × 1080 and a minimum frame rate of 30 fps. The transect survey dives followed a strict horizontal depth contour, running roughly parallel to the shore. Individual transect length hereby varied between gear types, with SCUBA transect lengths ~ 100 m and submersible and ROV transects ~ 250 m long. All gear types aimed to keep a constant altitude of 1-2 m above the benthos, allowing sufficient overlap between stereo cameras, yet staying close enough to the bottom to observe smaller benthic organisms. During sample collection dives, a maximum of five specimens from each depth per location were collected to verify some of our identifications. Hereby, a maximum of three samples were collected per morphotype, as per our permit.

All of the collected video footage was screened during and straight after the expedition in order to create image-based morphotype lists. These, along with collected specimens, were then reviewed during a taxonomic workshop that took place in the South African Institute of Aquatic Biodiversity in August 2019 (Stefanoudis et al. 2020). This process was really useful and speeded up the subsequent annotation of the transect survey video data in order to estimate the biodiversity and community composition of benthic and demersal fish assemblages of the Seychelles. For the annotation, we used the SeaGIS software EventMeasure and TransectMeasure and the results are currently being prepared for a separate publication.

## Data resources

### Authors' note

This guide is designed to aid with the identification of organisms as seen in underwater footage or in the field. For each entry, we provide a taxonomic identification and higher-order classification, information on distribution across our surveyed sites and observed depth ranges and sizes, based on our work only, a short morphological description as observed from the video footage and some representative images extracted from the video footage. Where available, an additional *ex-situ* (off-site) image of collected specimens is also provided.

Identifying taxa from images is challenging. Well-trained researchers use a combination of traditional taxonomic features and ecological information (e.g. depth, location, knowledge of the local species pool) to arrive at decisions on a taxon identification. The taxonomic level of each identification will vary depending on the type of organism in question, but in general, rarely reaches species level. This is due to a number of challenges, one of which being the often reduced quality of frames exported from video footage due to the camera moving fast or suspended sediment present within the frame. Additionally, some groups either have enormous morphological plasticity (e.g. sponges) or their unique characters are too small to be distinguished on video footage alone without the use of high-power microscopes (e.g. corals, algae). We have, therefore, placed each taxon into visually distinct morphotypes (i.e. aggregation of morphologically similar individuals) that can correspond to species or higher taxonomic level (genus, family etc.).

### How to use the Guide

All observed morphotypes are divided into 18 major classification groups, ranging from phylum to order. The choice of the taxonomic level for each major group corresponds with groups commonly recognised by the general public and experts alike, such as hard corals (Order: Scleractinia) or sponges (Phylum: Porifera). Members of each major group are then further classified into the lowest taxonomic level practical and then assigned to morphotypes. Table 1 provides an overview of all 184 recorded morphotypes.

Wherever species-level identifications are not possible, organisms are provided with a higher classification ranking (e.g. genus, family, class) followed by the use of open nomenclature (ON) signs applicable to image-based faunal analyses (e.g. indet., stet., inc.) as suggested by Horton et al. (2021). The use of ON signs allows standardisation and clarification of the uncertainty inherent in identification from image-based studies, thus enabling the subsequent use and comparability of generated datasets.

Finally, whenever *ex-situ* images of collected specimens are provided, they are accompanied by their unique sample number (e.g. SEY1_1377).

## Checklists

### Chlorophyta

#### Ulvophyceae

K.R. Mattox & K.D. Stewart, 1978

D122896D-9B42-5D18-A0C9-02BE833D1957

#### Bryopsidales

J.H. Schaffner, 1922

0F1575BC-E08E-5A93-9BA0-15EC4EE62714

#### Caulerpaceae

Kützing, 1843

2DC70C10-957B-566D-86CB-AAFBE5716CBD

#### Caulerpa

J.V. Lamouroux, 1809

F13A3CA2-CA4A-5A08-AD62-288F433E4D85

#### Caulerpa sp. indet. 1 f. sp. indet. 1

3A53C755-44F9-52E8-A492-8AC88C8D61BC

##### Materials

- **Type status:**Other material. **Taxon:** scientificName: *Caulerpa* sp. 1; kingdom: Plantae; phylum: Chlorophyta; class: Ulvophyceae; order: Bryopsidales; family: Caulerpaceae; genus: Caulerpa; scientificNameAuthorship: J. V. Lamouroux, 1809; **Location:** waterBody: Indian Ocean; country: Seychelles; locality: Aldabra N1, D'Arros N1, Desroches S1; minimumDepthInMeters: 10 m; maximumDepthInMeters: 47.6 m; locationRemarks: First Descent: Seychelles Expedition; **Identification:** identifiedBy: Nico Fassbender, Lydiane Mattio, Jeanne Mortimer, Paris Stefanoudis; dateIdentified: 2019, 2020; identificationRemarks: identified only from imagery; **Event:** samplingProtocol: Submersible OR Remotely Operated Vehicle OR SCUBA; **Record Level:** basisOfRecord: Human observation

##### Notes

A green seaweed that grows in twig-like, branched plants with a creeping stolon and multiple erect fonds. The stolon is attached to the seabed by several bunches of rhizoids. Species of *Caulerpa* are known for their plastic morphologies, which may vary greatly within the same species and between different environmental conditions (Fig. 2).

#### Caulerpa sp. indet. 2

3F72377D-92FA-5552-B22C-D2958B484969

##### Materials

- **Type status:**Other material. **Taxon:** scientificName: *Caulerpa* sp. 2; kingdom: Plantae; phylum: Chlorophyta; class: Ulvophyceae; order: Bryopsidales; family: Caulerpaceae; genus: Caulerpa; scientificNameAuthorship: Lamouroux, 1809; **Location:** waterBody: Indian Ocean; country: Seychelles; locality: Astove W1; minimumDepthInMeters: 30 m; maximumDepthInMeters: 30 m; locationRemarks: First Descent: Seychelles Expedition; **Identification:** identifiedBy: Nico Fassbender, Lydiane Mattio, Jeanne Mortimer, Paris Stefanoudis; dateIdentified: 2019, 2020; identificationRemarks: identified only from imagery; **Event:** samplingProtocol: Submersible OR Remotely Operated Vehicle OR SCUBA; **Record Level:** basisOfRecord: Human observation

##### Notes

**Figure 3.**
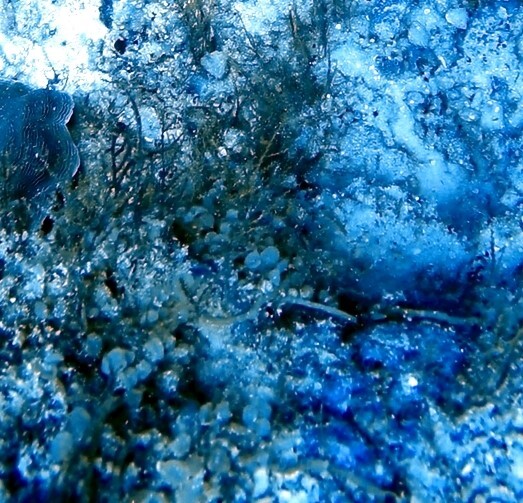
*Caulerpa* sp. indet. 2. Astove W1, 30 m.

A green seaweed with creeping stolon and erect fronds with branchlets consisting of a short pedicel ending in a rounded, disc-like to spherical appendage. Stolons are attached to the substratum by bunches of rhizoids. Species of *Caulerpa* are known for their plastic morphologies, that may vary greatly within the same species and between different environmental conditions (Fig. 3).

#### Codiaceae

Kützing, 1843

A3C83EC8-E773-5CB4-8F7C-EEBBCBE67F47

#### Codium

Stackhouse, 1797

57C04E40-EDE2-5AC1-A394-F2CBB74476FD

#### Codium sp. indet.

A50B8594-0931-5C89-B460-3EA5D7892EA4

##### Materials

- **Type status:**Other material. **Taxon:** scientificName: *Codium*; kingdom: Plantae; phylum: Chlorophyta; class: Ulvophyceae; order: Bryopsidales; family: Codiaceae; genus: Codium; scientificNameAuthorship: Stackhouse, 1797; **Location:** waterBody: Indian Ocean; country: Seychelles; locality: D'Arros N1, Desroches S1, Poivre E1; minimumDepthInMeters: 33.4 m; maximumDepthInMeters: 65.5 m; locationRemarks: First Descent: Seychelles Expedition; **Identification:** identifiedBy: Nico Fassbender, Lydiane Mattio, Jeanne Mortimer, Paris Stefanoudis; dateIdentified: 2019, 2020; identificationRemarks: identified only from imagery; **Event:** samplingProtocol: Submersible OR Remotely Operated Vehicle OR SCUBA; **Record Level:** basisOfRecord: Human observation

##### Notes

**Figure 4.**
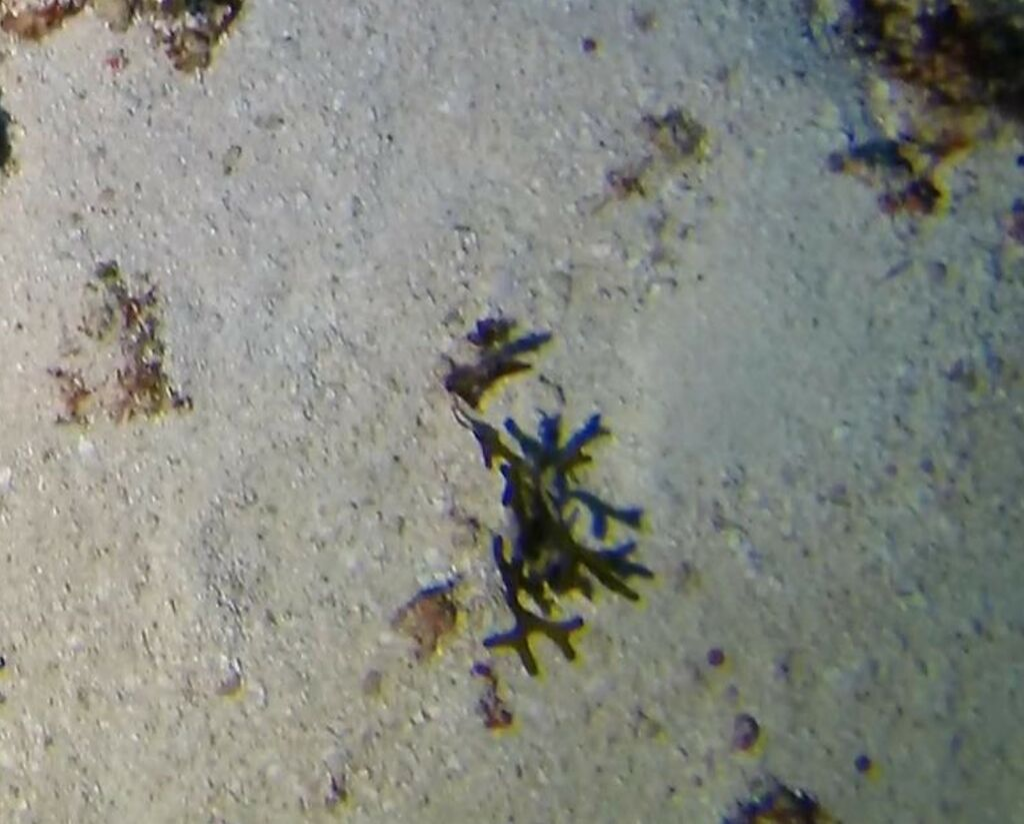
*Codium* sp. indet. Poivre E1, 60 m.

Seaweed whose thallus can display erect or prostrate forms, usually stiff. Erect forms (as observed here) display dichotomous branching, attached to the substratum by groups of rhizoids. The colour is dark green, sometimes with a brownish tint (Fig. 4).

#### Halimedaceae

Link, 1832

62A586F4-AE11-50FC-8E8A-F4AAACFFDB14

#### Halimeda

J.V.Lamouroux, 1812

BBA41176-8325-5561-9747-BF141C6146A1

#### Halimeda spp. indet.

F26EF66A-28F9-516D-B482-3E94ECA36313

##### Materials

- **Type status:**Other material. **Taxon:** scientificName: *Halimeda*; kingdom: Plantae; phylum: Chlorophyta; class: Ulvophyceae; order: Bryopsidales; family: Halimedaceae; genus: Halimeda; scientificNameAuthorship: Lamouroux, 1812; **Location:** waterBody: Indian Ocean; country: Seychelles; locality: Aldabra N1, Aldabra W1, Astove W1, Alphonse N1, D'Arros N1, Poivre E1, Desroches S1; minimumDepthInMeters: 9.5 m; maximumDepthInMeters: 70.4 m; locationRemarks: First Descent: Seychelles Expedition; **Identification:** identifiedBy: Nico Fassbender, Lydiane Mattio, Jeanne Mortimer, Paris Stefanoudis; dateIdentified: 2019, 2020; identificationRemarks: identified only from imagery; **Event:** samplingProtocol: Submersible OR Remotely Operated Vehicle OR SCUBA; **Record Level:** basisOfRecord: Human observation

##### Notes

Conspicuous, cactus-like macroalgae with jointed, disc-like and calcified segments. Individual segments can vary in shape that ranges from round to kidney-, wedge- or even cylindrical-shaped. The thallus anchors to the bottom by a dense tuft of rhizoids which varies in shape depending on the substratum. Dead specimens have been observed to lose their green colour, revealing their white calcium carbonate skeletons. Five species were identified from collections (*Halimedacylindracea*, H.aff.gracilis / H.aff.opuntia, *H.minima*, *H.micronesica*, *Halimeda* sp. indet); however, it was not possible to distinguish between them from underwater images alone (Fig. 5).

#### Udoteaceae

J. Agardh, 1887

FE2EBCCB-D90A-558A-97FE-ABD2F10E6BCF

#### Udotea

J.V.Lamouroux, 1812

6C69E35A-804F-52D3-BD01-31BF6D79CC4A

#### Udotea spp. indet.

1E5AAEDE-B0C9-5100-AEE4-D1C108F65D4A

##### Materials

- **Type status:**Other material. **Taxon:** scientificName: *Udotea*; kingdom: Plantae; phylum: Chlorophyta; class: Ulvophyceae; order: Bryopsidales; family: Udoteaceae; genus: Udotea; scientificNameAuthorship: Lamouroux, 1812; **Location:** waterBody: Indian Ocean; country: Seychelles; locality: D'Arros N1, Desroches S1, Poivre E1; minimumDepthInMeters: 3 m; maximumDepthInMeters: 36.5 m; locationRemarks: First Descent: Seychelles Expedition; **Identification:** identifiedBy: Nico Fassbender, Lydiane Mattio, Jeanne Mortimer, Paris Stefanoudis; dateIdentified: 2019, 2020; identificationRemarks: identified only from imagery; **Event:** samplingProtocol: Submersible OR Remotely Operated Vehicle OR SCUBA; **Record Level:** basisOfRecord: Human observation

##### Notes

A green calcified seaweed composed of a stipe and either a single or several fan-shaped blades. It is anchored to the bottom by uncalcified tufts of rhizoids, which vary in shape depending on the substratum, most commonly sand. *Udotea* species are common in coral reef ecosystems and occur globally from tropical to subtropical latitudes. Two species were identified from collections (*Udotea* sp. indet. 1 and sp. indet. 2); however, it was not possible to distinguish between them from underwater images alone (Fig. 6).

#### Cladophorales

Haeckel, 1894

12DDAD24-3B63-573D-A5A9-65D5E95F7794

#### Anadyomenaceae

Kützing, 1843

6E839E6C-F359-595A-B160-ED5EC09E55A1

#### Microdictyon

Decaisne, 1841

8A0DB8FC-93A3-56A2-B00B-7CB34CF18A0A

#### Microdictyon sp. indet.

1C95A86C-EE09-5D18-9E95-6AACC6130821

##### Materials

- **Type status:**Other material. **Taxon:** scientificName: *Microdictyon*; kingdom: Plantae; phylum: Chlorophyta; class: Ulvophyceae; order: Cladophorales; family: Anadyomenaceae; genus: Microdictyon; scientificNameAuthorship: Decaisne, 1841; **Location:** waterBody: Indian Ocean; country: Seychelles; locality: Poivre E1; minimumDepthInMeters: 33.4 m; maximumDepthInMeters: 36.5 m; locationRemarks: First Descent: Seychelles Expedition; **Identification:** identifiedBy: Nico Fassbender, Lydiane Mattio, Jeanne Mortimer, Paris Stefanoudis; dateIdentified: 2019, 2020; identificationRemarks: identified only from imagery; **Event:** samplingProtocol: Submersible OR Remotely Operated Vehicle OR SCUBA; **Record Level:** basisOfRecord: Human observation

##### Notes

Thin, leaf-like green algae made of complanate, monostromatic, reticulate blades that occasionally form dense mats (Fig. 7).

#### Siphonocladaceae

Schmitz, 1879

110D441C-1719-5FF0-A2A9-F443070F9A4F

#### Dictyosphaeria

Decaisne, 1842

D64AF155-32EB-5D30-9341-9B92D1732B1C

#### Dictyosphaeria sp. indet.

FA8EFB99-DBC4-5608-9679-908A2A61C564

##### Materials

- **Type status:**Other material. **Taxon:** scientificName: *Dictyosphaeria*; kingdom: Plantae; phylum: Chlorophyta; class: Ulvophyceae; order: Cladophorales; family: Siphonocladaceae; genus: Dictyosphaeria; scientificNameAuthorship: Decaisne, 1842; **Location:** waterBody: Indian Ocean; country: Seychelles; locality: Astove W1; minimumDepthInMeters: 10 m; maximumDepthInMeters: 12 m; locationRemarks: First Descent: Seychelles Expedition; **Identification:** identifiedBy: Nico Fassbender, Lydiane Mattio, Jeanne Mortimer, Paris Stefanoudis; dateIdentified: 2019, 2020; identificationRemarks: identified only from imagery; **Event:** samplingProtocol: Submersible OR Remotely Operated Vehicle OR SCUBA; **Record Level:** basisOfRecord: Human observation

##### Notes

**Figure 8.**
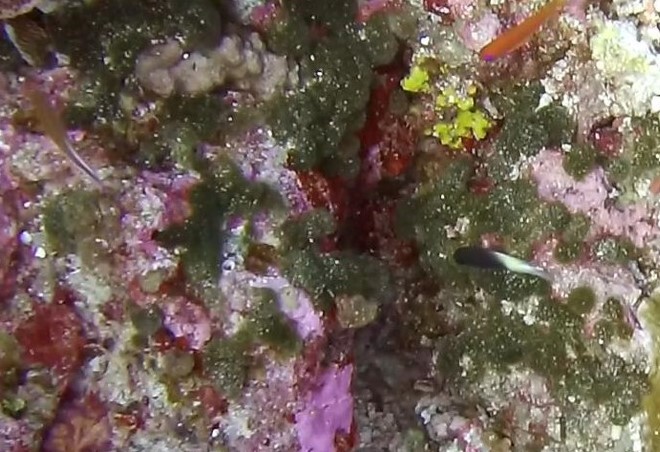
*Dictyosphaeria* sp. indet. Astove W1, 10 m.

A green algae that forms somewhat encrusting, hollow or solid, globose or flattened thalli made of vesicular segments (pseudoparenchymatous cushion of polygonal cells). They are attached to the substratum by rhizoids produced by basal vesicles (Fig. 8).

#### Ulvales

Blackman & Tansley, 1902

A73E5089-0109-51B7-AA66-C86E03C25435

#### Ulvaceae

J.V. Lamouroux ex Dumortier, 1822

F51CDB9D-A925-5446-B6EA-5DDE921BFC46

#### Ulva

Linnaeus, 1753

9215A844-ED79-502F-B7B2-63C088CB600E

#### Ulva sp. indet.

91063347-F7FA-5D38-8315-7A20B51376B1

##### Materials

- **Type status:**Other material. **Taxon:** scientificName: *Ulva* (cf.); kingdom: Plantae; phylum: Chlorophyta; class: Ulvophyceae; order: Ulvales; family: Ulvaceae; genus: Ulva; scientificNameAuthorship: Linnaeus, 1753; **Location:** waterBody: Indian Ocean; country: Seychelles; locality: Desroches S1; minimumDepthInMeters: 61.8 m; maximumDepthInMeters: 71.5 m; locationRemarks: First Descent: Seychelles Expedition; **Identification:** identifiedBy: Nico Fassbender, Lydiane Mattio, Jeanne Mortimer, Paris Stefanoudis; dateIdentified: 2019, 2020; identificationRemarks: identified only from imagery; **Event:** samplingProtocol: Submersible OR Remotely Operated Vehicle OR SCUBA; **Record Level:** basisOfRecord: Human observation

##### Notes

**Figure 9.**
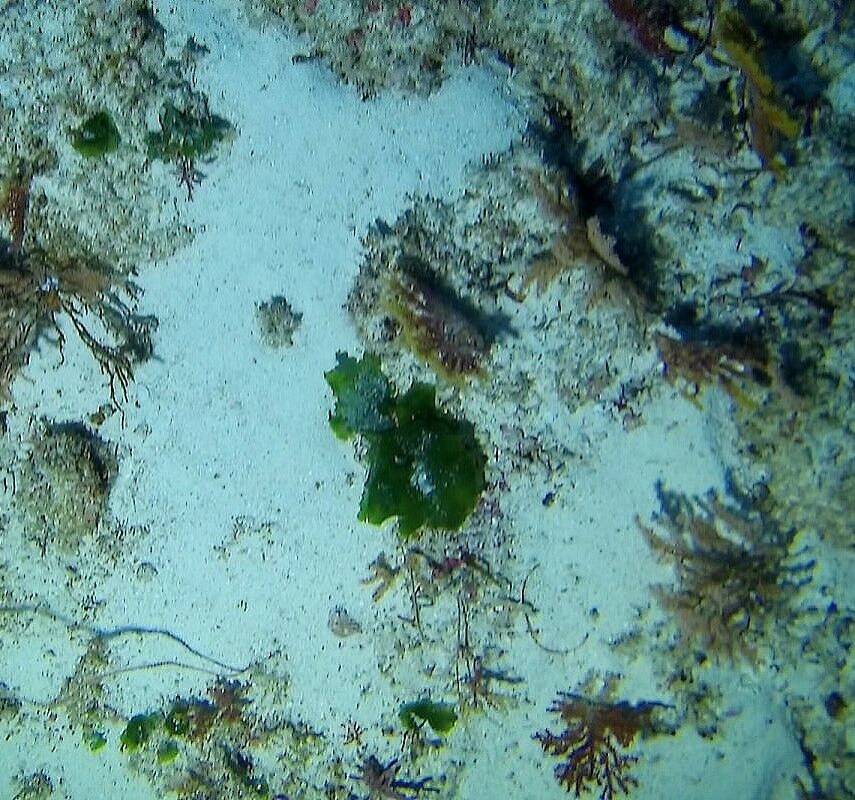
*Ulva* sp. indet. Desroches S1, 60 m.

Thallus can vary in shape and resemble lettuce leaves. The thallus is composed of two layers of cells attached to the substratum by a holdfast made of rhizoidal proliferations. The overall shape of the algae is very variable depending on the environmental conditions. Colour varies from light to dark green (Fig. 9).

### Ochrophyta

#### Phaeophyceae

Kjellman, 1891

2372FFFB-6EA0-5FBA-9E22-206CDED5F6FB

#### Dictyotales

Bory de Saint-Vincent, 1828

65A1E2DF-5974-5076-B2F2-4CE9A1B49F40

#### Dictyotaceae

Lamouroux ex Dumortier, 1822

12E444C8-2591-5255-97F5-E8330EC2C735

#### Lobophora

J.Agardh, 1894

91ACB3B7-E754-54FF-AD3D-7DF7C381629A

#### Lobophora sp. indet.

4B8C5BB4-9A06-554A-A3EC-6AB0A76F4E03

##### Materials

- **Type status:**Other material. **Taxon:** scientificName: *Lobophora*; kingdom: Plantae; phylum: Ochrophyta; class: Phaeophyceae; order: Dictyotales; family: Dictyotaceae; genus: Lobophora; scientificNameAuthorship: Agardh, 1894; **Location:** waterBody: Indian Ocean; country: Seychelles; locality: Aldabra W1, Astove W1, Alphonse N1, D'Arros N1; minimumDepthInMeters: 10 m; maximumDepthInMeters: 72 m; locationRemarks: First Descent: Seychelles Expedition; **Identification:** identifiedBy: Nico Fassbender, Lydiane Mattio, Jeanne Mortimer, Paris Stefanoudis; dateIdentified: 2019, 2020; identificationRemarks: identified only from imagery; **Event:** samplingProtocol: Submersible OR Remotely Operated Vehicle OR SCUBA; **Record Level:** basisOfRecord: Human observation

##### Notes

Brown fan-shaped blade with a firm texture. The creeping, ascendant or erect fonds can range from foliose to rounded and are attached to the substratum by rhizoids. Previously thought to be represented by only one species (*Lobophoravariegata*), genetics (Vieira et al. 2016) have recently revealed a much wider species diversity than conventional methods of identification, based on macromorphological characters alone (Fig. 10).

### Rhodophyta

#### Florideophyceae

Cronquist, 1960

CFF2F772-F471-5FFC-A9A4-B5356E1A2160

#### Ceramiales

Oltmanns, 1904

750BFC2F-5B20-5148-81EF-0D7722CE847D

#### Dasyaceae

Kützing, 1843

2C83ACDE-7315-56E3-9737-7EE75945EBA3

#### Amphisbetema

Weber-van Bosse, 1913

F39BB955-D8BD-5DDD-A2D9-D599D6912612

#### Amphisbetema indica

(J.Agardh) Weber-van Bosse, 1913

ED8E8E38-D888-5AF8-989C-97EE33C207A5

##### Materials

- **Type status:**Other material. **Taxon:** scientificName: *Amphisbetemaindica*; kingdom: Plantae; phylum: Rhodophyta; class: Florideophyceae; order: Ceramiales; family: Dasyaceae; genus: Amphisbetema; scientificNameAuthorship: Weber-van Bosse, 1913; **Location:** waterBody: Indian Ocean; country: Seychelles; locality: Desroches S1; minimumDepthInMeters: 10 m; maximumDepthInMeters: 13 m; locationRemarks: First Descent: Seychelles Expedition; **Identification:** identifiedBy: Nico Fassbender, Lydiane Mattio, Jeanne Mortimer, Paris Stefanoudis; dateIdentified: 2019, 2020; identificationRemarks: identified only from imagery; **Event:** samplingProtocol: Submersible OR Remotely Operated Vehicle OR SCUBA; **Record Level:** basisOfRecord: Human observation

##### Notes

Creeping red fleshy algae with small, arborescent and feather-like fonds arising from a decumbent rhizome-like base (Fig. 11).

#### Corallinales

P.C. Silva & H.W. Johansen, 1986

1CF76831-7202-5BD7-8196-5F698C361706

#### "ord. Corallinales" stet.

D40AE817-14B4-54A0-9EB8-C125EFAA820B

##### Materials

- **Type status:**Other material. **Taxon:** scientificName: Corallinales; kingdom: Plantae; phylum: Rhodophyta; class: Florideophyceae; order: Corallinales; scientificNameAuthorship: P.C. Silva & H.W. Johansen, 1986; **Location:** waterBody: Indian Ocean; country: Seychelles; locality: Aldabra N1, Aldabra W1, Alphonse N1, Astove W1, D'Arros N1, Desroches S1, Poivre E1; minimumDepthInMeters: 8.8 m; maximumDepthInMeters: 148.1 m; locationRemarks: First Descent: Seychelles Expedition; **Identification:** identifiedBy: Nico Fassbender, Lydiane Mattio, Jeanne Mortimer, Paris Stefanoudis; dateIdentified: 2019, 2020; identificationRemarks: identified only from imagery; **Event:** samplingProtocol: Submersible OR Remotely Operated Vehicle OR SCUBA; **Record Level:** basisOfRecord: Human observation

##### Notes

Commonly known as crustose coralline algae, these encrusting algae grow on rocks, coral fragments, shells, other algae or seagrasses. Hard and rock-like, their surface can be smooth or rough. Colours range from bright pink to purple. This group contains a variety of species that are difficult to identify from images, hence, no attempt was made to identify them at a lower taxonomic level (Fig. 12).

### Tracheophyta

#### Magnoliopsida

A0946C46-2F1C-515B-B38F-DDA9338BE15E

#### Alismatales

R.Br. ex Bercht. & J.Presl, 1820

D5A194E3-C2BD-53F0-ACBA-E29CC8CE4EF8

#### Cymodoceaceae

Vines, 1895

35F6A96F-9F1E-56BF-8538-71DA821F2FA4

#### Thalassodendron

Hartog, 1970

96B8E4E1-47F8-5739-9054-FC0F3FC7D84D

#### Thalassodendron ciliatum

(Forsskål) Hartog, 1970

0CB77927-093E-5E6A-A2E9-318DC9DA98D7

##### Materials

- **Type status:**Other material. **Taxon:** scientificName: *Thalassodendronciliatum*; kingdom: Plantae; phylum: Tracheophyta; class: Magnoliopsida; order: Alismatales; family: Cymodoceaceae; genus: Thalassodendron; scientificNameAuthorship: (Forsskål) Hartog, 1970; **Location:** waterBody: Indian Ocean; country: Seychelles; locality: Poivre E1; minimumDepthInMeters: 10 m; maximumDepthInMeters: 10 m; locationRemarks: First Descent: Seychelles Expedition; **Identification:** identifiedBy: Nico Fassbender, Lydiane Mattio, Jeanne Mortimer, Paris Stefanoudis; dateIdentified: 2019, 2020; identificationRemarks: identified only from imagery; **Event:** samplingProtocol: Submersible OR Remotely Operated Vehicle OR SCUBA; **Record Level:** basisOfRecord: Human observation

##### Notes

**Figure 13.**
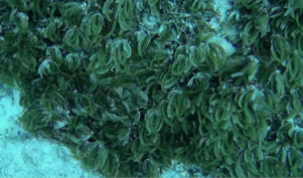
*Thalassodendronciliatum*. Poivre E1, 10 m.

This seagrass species can form dense meadows and is identified by its linear and falcate leaves arising from a rooted rhizome. Its colour is a rich green. One species (*Thalassodendronciliatum*) was identified from collections (Fig. 13).

#### Hydrocharitaceae

Jussieu, 1789

549EB67A-23C6-503A-813A-3976F727D317

#### Halophila

Du Petit-Thouars, 1806

57467647-4712-5D3C-9C03-C80BAE022307

#### Halophila sp. indet.

A2E54171-3E6D-5E82-A2ED-EF78044D0D34

##### Materials

- **Type status:**Other material. **Taxon:** scientificName: *Halophila*; kingdom: Plantae; phylum: Tracheophyta; class: Magnoliopsida; order: Alismatales; family: Hydrocharitaceae; genus: Halophila; scientificNameAuthorship: Du-Petit Thouars, 1806; **Location:** waterBody: Indian Ocean; country: Seychelles; locality: D'Arros N1; minimumDepthInMeters: 31.7 m; maximumDepthInMeters: 36.5 m; locationRemarks: First Descent: Seychelles Expedition; **Identification:** identifiedBy: Nico Fassbender, Lydiane Mattio, Jeanne Mortimer, Paris Stefanoudis; dateIdentified: 2019, 2020; identificationRemarks: identified only from imagery; **Event:** samplingProtocol: Submersible OR Remotely Operated Vehicle OR SCUBA; **Record Level:** basisOfRecord: Human observation

##### Notes

**Figure 14.**
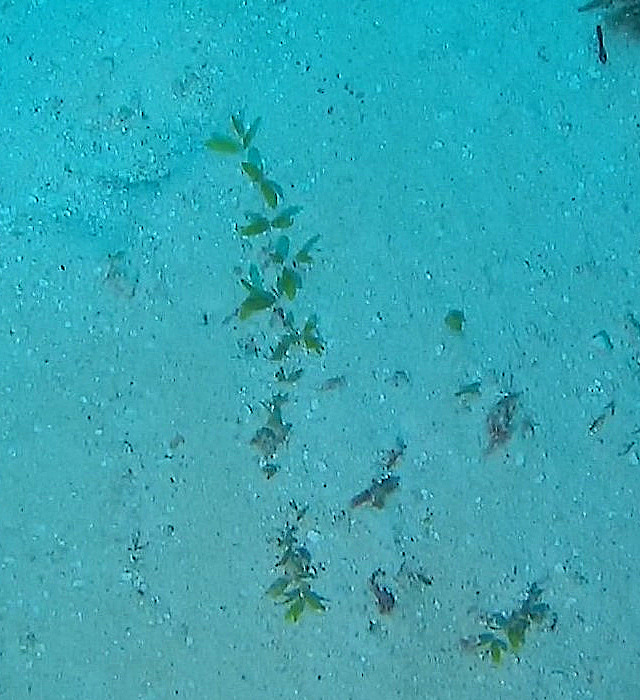
*Halophila* sp. indet. D'Arros N1, 30 m.

Species of seagrass with a creeping bifurcated stem from which arise distichously arranged linear, oblong or rounded delicate leaves. Colour light green (Fig. 14).

### Actiniaria

#### Stichodactylidae

Andres, 1883

BB047E4A-D61E-5A41-881A-8E3A29C4CFB5

#### Heteractis

Milne-Edwards & Haime, 1851

2E9F7770-F59E-51BB-AB3F-F3C2C6A7B33B

#### Heteractis magnifica

(Quoy & Gaimard, 1833)

AA9D1CF1-E49C-53ED-BB9E-D7A225771CD3

##### Materials

- **Type status:**Other material. **Taxon:** scientificName: *Heteractismagnifica*; kingdom: Animalia; phylum: Cnidaria; class: Anthozoa; order: Actiniaria; family: Stichodactylidae; genus: Heteractis; scientificNameAuthorship: Quoy & Gaimard, 1833; **Location:** waterBody: Indian Ocean; country: Seychelles; locality: D'Arros N1; minimumDepthInMeters: 31.8 m; maximumDepthInMeters: 34.3 m; locationRemarks: First Descent: Seychelles Expedition; **Identification:** identifiedBy: Nico Fassbender, Paris Stefanoudis; dateIdentified: 2019, 2020; identificationRemarks: identified only from imagery; **Event:** samplingProtocol: Submersible OR Remotely Operated Vehicle OR SCUBA; **Record Level:** basisOfRecord: Human observation

##### Notes

**Figure 15.**
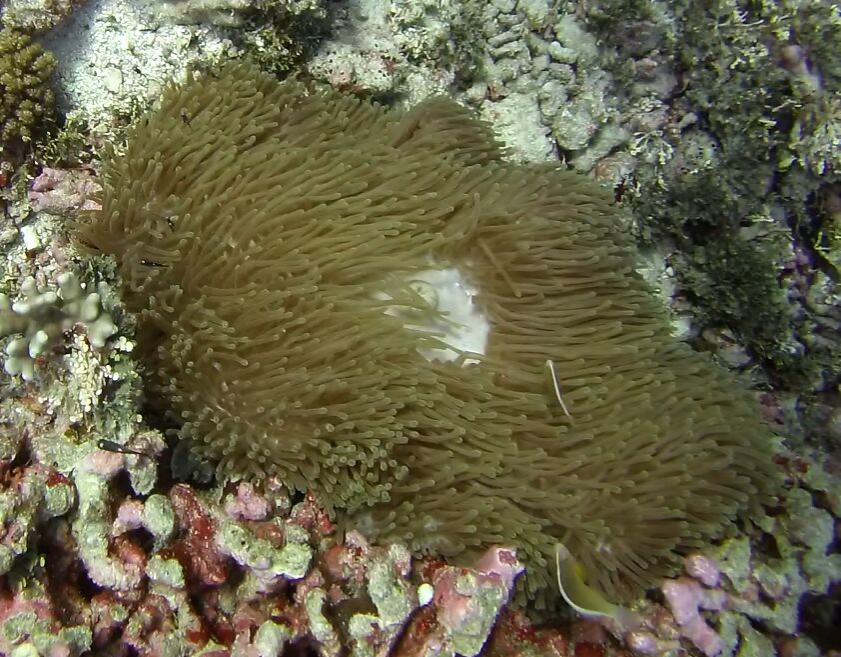
*Heteractismagnifica*. D'Arros N1, 30 m.

Oval oral disc that is flat or slightly undulating and densely covered with finger-like tentacles. Tentacles are hardly tapered or blunt, sometimes with a swollen end. Oral disc white; tentacles light brown to green. Typically found growing in comparably exposed positions. They can host anemonefish and are associated with the anemonefish species *Amphiprionakallopisos* (pictured below), in Seychelles waters. Furthermore, *Dascyllustrimaculatus* and various shrimp species may live inside the anemone. Similar-looking species include *Stichodactylamertensii*, with *H.magnifica* being much more substantial and its oral disc and tentacles of uniform colouration, with a brightly coloured column (where visible) (Fig. 15).

#### Stichodactyla

Brandt, 1835

2D8CCC87-062E-526D-A2F6-5FDB4D433DD1

#### Stichodactyla mertensii f. sp. 1 var. sp. 1

Brandt, 1835

D969BA0B-03CF-51C6-A49C-F1AC0EC18C9F

##### Materials

- **Type status:**Other material. **Taxon:** scientificName: *Stichodactylamertensii*; kingdom: Animalia; phylum: Cnidaria; class: Anthozoa; order: Actiniaria; family: Stichodactylidae; genus: Stichodactyla; scientificNameAuthorship: Brandt, 1835; **Location:** waterBody: Indian Ocean; country: Seychelles; locality: Aldabra W1, Poivre E1; minimumDepthInMeters: 32.4 m; maximumDepthInMeters: 35.4 m; locationRemarks: First Descent: Seychelles Expedition; **Identification:** identifiedBy: Nico Fassbender, Paris Stefanoudis; dateIdentified: 2019, 2020; identificationRemarks: identified only from imagery; **Event:** samplingProtocol: Submersible OR Remotely Operated Vehicle OR SCUBA; **Record Level:** basisOfRecord: Human observation

##### Notes

**Figure 16.**
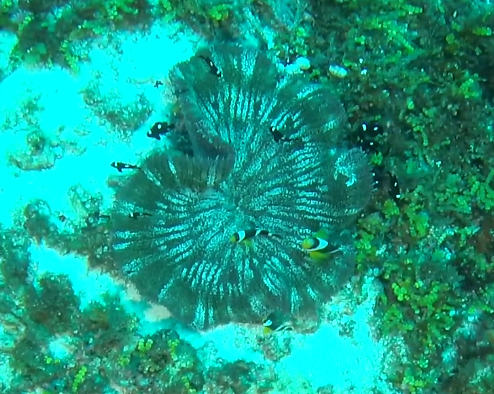
*Stichodactylamertensii*. Poivre E1, 30 m.

Meandering oral disc; surface covered in small tentacles (~ 1-2 cm), sometimes longer (~ 5 cm). Dark brown colouration with whitish stripes throughout the colony. Hosts several species of anemonefish, associated with *Amphiprionclarkii* or *Amphiprionfuscocaudatus* (see Fig. 16) in Seychelles waters.

#### Actiniaria

Hertwig, 1882

DD502C97-A21D-5527-B99A-80807CC28261

#### "ord. Actiniaria" fam. indet. sp. 1

C4CB5C2A-27F4-5712-98E7-21253C8759BB

##### Materials

- **Type status:**Other material. **Taxon:** scientificName: Actiniaria sp. 1; kingdom: Animalia; phylum: Cnidaria; class: Anthozoa; order: Actiniaria; scientificNameAuthorship: Hertwig, 1882; **Location:** waterBody: Indian Ocean; country: Seychelles; locality: Aldabra W1, Alphonse N1, D'Arros N1; minimumDepthInMeters: 230 m; maximumDepthInMeters: 254.1 m; locationRemarks: First Descent: Seychelles Expedition; **Identification:** identifiedBy: Nico Fassbender, Paris Stefanoudis; dateIdentified: 2019, 2020; identificationRemarks: identified only from imagery; **Event:** samplingProtocol: Submersible OR Remotely Operated Vehicle OR SCUBA; **Record Level:** basisOfRecord: Human observation

##### Notes

**Figure 17.**
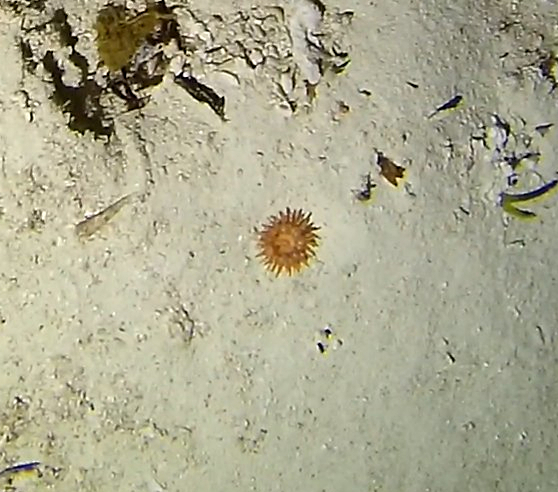
Actiniaria fam. indet. sp. 1. Aldabra W1, 250 m.

Round oral disc (~ 4 cm in diameter) with numerous short tentacles (~ 1.5 cm) along the outer edge of the disc. Colour orange to reddish. Further microscopic examination is necessary for positive taxonomic identification (Fig. 17).

#### "ord. Actiniaria" fam. indet. sp. 2

FC23B545-2491-5792-8622-C814A111448D

##### Materials

- **Type status:**Other material. **Taxon:** scientificName: Actiniaria sp. 2; kingdom: Animalia; phylum: Cnidaria; class: Anthozoa; order: Actiniaria; scientificNameAuthorship: Hertwig, 1882; **Location:** waterBody: Indian Ocean; country: Seychelles; locality: Aldabra W1; minimumDepthInMeters: 249.3 m; maximumDepthInMeters: 251.9 m; locationRemarks: First Descent: Seychelles Expedition; **Identification:** identifiedBy: Nico Fassbender, Paris Stefanoudis; dateIdentified: 2019, 2020; identificationRemarks: identified only from imagery; **Event:** samplingProtocol: Submersible OR Remotely Operated Vehicle OR SCUBA; **Record Level:** basisOfRecord: Human observation

##### Notes

**Figure 18.**
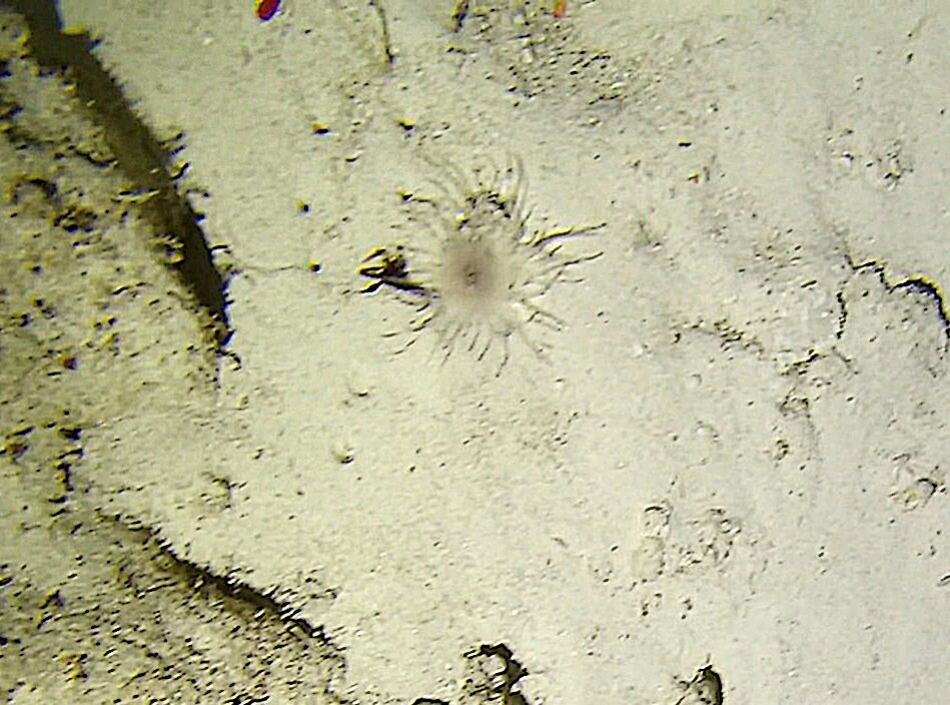
Actiniaria fam. indet. sp. 2. Aldabra W1, 250 m.

Round oral disc (~ 2.5 cm in diameter) with numerous, thin (~ 2.5 cm long) tentacles along the outer edge of the disc. Colour translucent to white. Further microscopic examination is needed for positive taxonomic identification (Fig. 18).

#### "ord. Actiniaria" fam. indet. sp. 3

E2B9AE5F-BA22-5FD6-98B2-F3A0982F778C

##### Materials

- **Type status:**Other material. **Taxon:** scientificName: Actiniaria sp. 3; kingdom: Animalia; phylum: Cnidaria; class: Anthozoa; order: Actiniaria; scientificNameAuthorship: Hertwig, 1882; **Location:** waterBody: Indian Ocean; country: Seychelles; locality: Aldabra W1; minimumDepthInMeters: 132 m; maximumDepthInMeters: 140.4 m; locationRemarks: First Descent: Seychelles Expedition; **Identification:** identifiedBy: Nico Fassbender, Paris Stefanoudis; dateIdentified: 2019, 2020; identificationRemarks: identified only from imagery; **Event:** samplingProtocol: Submersible OR Remotely Operated Vehicle OR SCUBA; **Record Level:** basisOfRecord: Human observation

##### Notes

**Figure 19.**
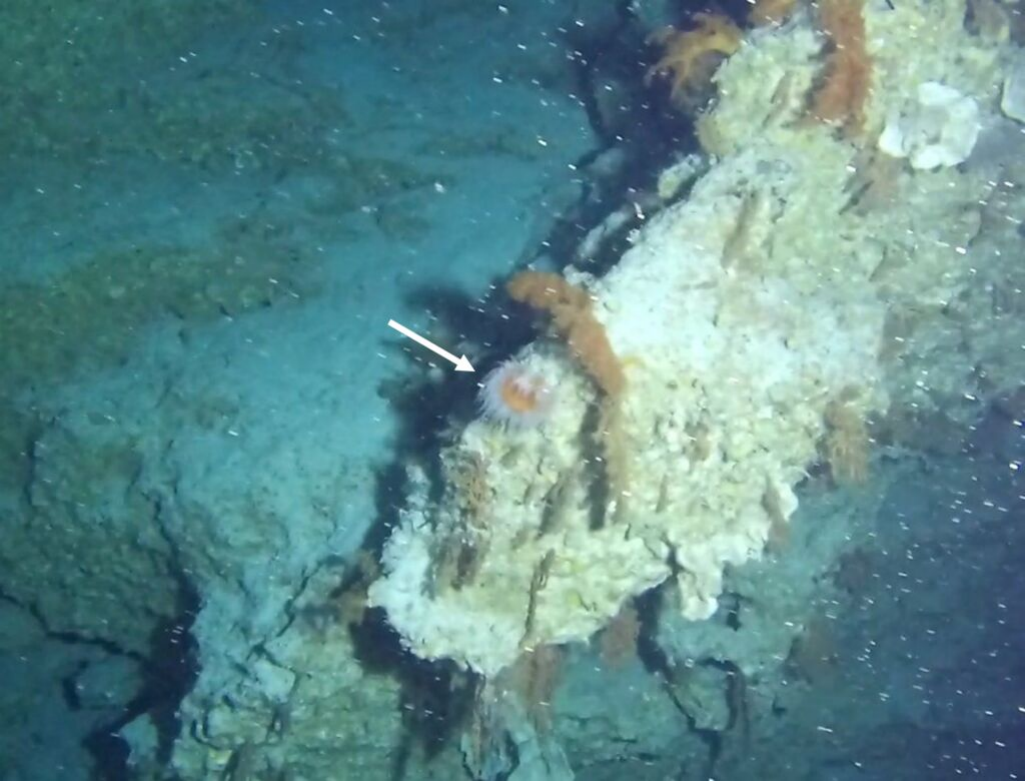
Actiniaria fam. indet. sp. 3. Aldabra W1, 120 m.

Round oral disc (7 cm in diameter) with numerous thin tentacles (~ 6 cm long) along the outer edge of the disc. Oral disc pale to bright orange, tentacles translucent to pale white. Further microscopic examination is needed for positive taxonomic identification (Fig. 19).

#### "ord. Actiniaria" fam. indet. sp. 6

563088E3-B5D4-50DB-99DE-FD94F19176AA

##### Materials

- **Type status:**Other material. **Taxon:** scientificName: Actiniaria sp. 6; kingdom: Animalia; phylum: Cnidaria; class: Anthozoa; order: Actiniaria; scientificNameAuthorship: Hertwig, 1882; **Location:** waterBody: Indian Ocean; country: Seychelles; locality: Aldabra W1; minimumDepthInMeters: 250 m; maximumDepthInMeters: 250 m; locationRemarks: First Descent: Seychelles Expedition; **Identification:** identifiedBy: Nico Fassbender, Paris Stefanoudis; dateIdentified: 2019, 2020; identificationRemarks: identified only from imagery; **Event:** samplingProtocol: Submersible OR Remotely Operated Vehicle OR SCUBA; **Record Level:** basisOfRecord: Human observation

##### Notes

Round oral disc entirely covered by thick, long (with respect to the size of the disc) tentacles. Colour of oral disc unknown; tentacles dark brown to dark red with white tips. Further microscopic examination is needed for positive taxonomic identification (Fig. 20).

### Antipatharia

#### Antipathidae

Ehrenberg, 1834

E26DD13E-4574-5CFA-8B68-25DED753F4E6

#### Antipathes

Pallas, 1766

FCC6F3D6-C3C5-5CB6-BD30-2B9DECA5A89C

#### Antipathes sp. indet.

57C3E685-9594-59B8-B2BB-7B0BD6705996

##### Materials

- **Type status:**Other material. **Taxon:** scientificName: *Antipathes*; kingdom: Animalia; phylum: Cnidaria; class: Anthozoa; order: Antipatharia; family: Antipathidae; genus: Antipathes; scientificNameAuthorship: Pallas, 1766; **Location:** waterBody: Indian Ocean; country: Seychelles; locality: Aldabra N1, Alphonse N1, Astove W1, Desroches S1; minimumDepthInMeters: 21.7 m; maximumDepthInMeters: 122 m; locationRemarks: First Descent: Seychelles Expedition; **Identification:** identifiedBy: Nico Fassbender, Paris Stefanoudis, Daniel Wagner; dateIdentified: 2019, 2020; identificationRemarks: identified only from imagery; **Event:** samplingProtocol: Submersible OR Remotely Operated Vehicle OR SCUBA; **Record Level:** basisOfRecord: Human observation

##### Notes

Colonies up to 1.7 m in height, mainly bushy and bramble-like, sparsely to densely-branched with fine, elongate branches. Light to dark brown colour (Fig. 21).

#### Leiopathidae

Haeckel, 1896

910C63E4-47C5-5266-A50B-DB7149260C67

#### Leiopathes

Haime, 1849

AA3D6E99-7FB8-50D9-A931-F62E13BE7C18

#### Leiopathes sp. indet.

98396206-1DA3-5FF3-8D8F-5340CC0260C3

##### Materials

- **Type status:**Other material. **Taxon:** scientificName: *Leiopathes*; kingdom: Animalia; phylum: Cnidaria; class: Anthozoa; order: Antipatharia; family: Leiopathidae; genus: Leiopathes; scientificNameAuthorship: Haime, 1849; **Location:** waterBody: Indian Ocean; country: Seychelles; locality: Alphonse N1; minimumDepthInMeters: 250 m; maximumDepthInMeters: 250 m; locationRemarks: First Descent: Seychelles Expedition; **Identification:** identifiedBy: Nico Fassbender, Paris Stefanoudis, Daniel Wagner; dateIdentified: 2019, 2020; identificationRemarks: identified only from imagery; **Event:** samplingProtocol: Submersible OR Remotely Operated Vehicle OR SCUBA; **Record Level:** basisOfRecord: Human observation

##### Notes

Colonies observed were large (> 2 m in height), fan-shaped and uniplanar. With thick, central stalk and several finer branches. Colour dark red to orange (Fig. 22).

#### Myriopathidae

Opresko, 2001

F7EEAC42-F185-57DF-A443-DB232BCF1520

#### Cupressopathes

Opresko, 2001

1E76C3B1-F9C1-5A41-9AD7-E8BEA33D5563

#### Cupressopathes sp. indet.

F4FC9CBB-72C3-5786-BE31-5D8142956C44

##### Materials

- **Type status:**Other material. **Taxon:** scientificName: *Cupressopathes*; kingdom: Animalia; phylum: Cnidaria; class: Anthozoa; order: Antipatharia; family: Myriopathidae; genus: Cupressopathes; scientificNameAuthorship: Opresko, 2001; **Location:** waterBody: Indian Ocean; country: Seychelles; locality: Aldabra N1, Aldabra W1, D'Arros N1, Desroches S1, Poivre E1; minimumDepthInMeters: 31.1 m; maximumDepthInMeters: 72 m; locationRemarks: First Descent: Seychelles Expedition; **Identification:** identifiedBy: Nico Fassbender, Paris Stefanoudis, Daniel Wagner; dateIdentified: 2019, 2020; identificationRemarks: identified only from imagery; **Event:** samplingProtocol: Submersible OR Remotely Operated Vehicle OR SCUBA; **Record Level:** basisOfRecord: Human observation

##### Notes

Colonies up to 20 cm in height, columnar, monopodial or very sparsely branched. Thick bottlebrush-like appearance. Irregularly pinnulate. Brownish to grey colouration. Darker coloured central axis enclosed by bushy, lighter coloured branches and polyps (Fig. 23).

#### Myriopathes

Opresko, 2001

A7AD4D62-5CE2-5F68-B9EE-2DA0429F8905

#### Myriopathes sp. indet.

E87E3955-BB63-5CA4-B73A-0DEA67588150

##### Materials

- **Type status:**Other material. **Taxon:** scientificName: *Myriopathes*; kingdom: Animalia; phylum: Cnidaria; class: Anthozoa; order: Antipatharia; family: Myriopathidae; genus: Myriopathes; scientificNameAuthorship: Opresko, 2001; **Location:** waterBody: Indian Ocean; country: Seychelles; locality: Aldabra N1, Aldabra W1, Alphonse N1, D'Arros N1, Desroches S1, Poivre E1; minimumDepthInMeters: 30 m; maximumDepthInMeters: 122.6 m; locationRemarks: First Descent: Seychelles Expedition; **Identification:** identifiedBy: Nico Fassbender, Paris Stefanoudis, Daniel Wagner; dateIdentified: 2019, 2020; identificationRemarks: identified only from imagery; **Event:** samplingProtocol: Submersible OR Remotely Operated Vehicle OR SCUBA; **Record Level:** basisOfRecord: Human observation

##### Notes

Colonies up to 1.8 m in height, densely branched, appearing rather bushy. Colouration ranges from brownish to grey and orange, with polyps coloured lighter than the branches (Fig. 24).

#### Schizopathidae

Brook, 1889

D866ECC1-9903-5BC4-80B5-AD58F252A57E

#### Bathypathes

Brook, 1889

FB22BCB6-3004-53C5-9671-0C796ECAE48C

#### Bathypathes sp. indet.

7EFD6F80-5FF9-507C-BB92-6FF4070AA5F5

##### Materials

- **Type status:**Other material. **Taxon:** scientificName: *Bathypathes*; kingdom: Animalia; phylum: Cnidaria; class: Anthozoa; order: Antipatharia; family: Schizopathidae; genus: Bathypathes; scientificNameAuthorship: Brook, 1889; **Location:** waterBody: Indian Ocean; country: Seychelles; locality: D'Arros N1; minimumDepthInMeters: 344 m; maximumDepthInMeters: 351 m; locationRemarks: First Descent: Seychelles Expedition; **Identification:** identifiedBy: Nico Fassbender, Paris Stefanoudis, Daniel Wagner; dateIdentified: 2019, 2020; identificationRemarks: identified only from imagery; **Event:** samplingProtocol: Submersible OR Remotely Operated Vehicle OR SCUBA; **Record Level:** basisOfRecord: Human observation

##### Notes

Colonies have two rows of fine and long branches on either side of the central axis and grow up to ~ 15–20 cm in height. Branches are thin and rounded, giving the colony a feather-like appearance. Colonies are pink to purple coloured (Fig. 25).

#### Stylopathidae

Opresko, 2006

37CAE267-1C9F-54F5-BF06-9A7084B5507E

#### Stylopathes

Opresko, 2006

F2FA9850-7C2C-5770-814B-193038085829

#### Stylopathes sp. indet.

479EFE03-640F-59A6-84C8-281415C98D6B

##### Materials

- **Type status:**Other material. **Taxon:** scientificName: *Stylopathes*; kingdom: Animalia; phylum: Cnidaria; class: Anthozoa; order: Antipatharia; family: Stylopathidae; genus: Stylopathes; scientificNameAuthorship: Opresko, 2006; **Location:** waterBody: Indian Ocean; country: Seychelles; locality: Aldabra W1, Alphonse N1, D'Arros N1, Poivre E1; minimumDepthInMeters: 245.6 m; maximumDepthInMeters: 350 m; locationRemarks: First Descent: Seychelles Expedition; **Identification:** identifiedBy: Nico Fassbender, Paris Stefanoudis, Daniel Wagner; dateIdentified: 2019, 2020; identificationRemarks: identified only from imagery; **Event:** samplingProtocol: Submersible OR Remotely Operated Vehicle OR SCUBA; **Record Level:** basisOfRecord: Human observation

##### Notes

**Figure 26.**
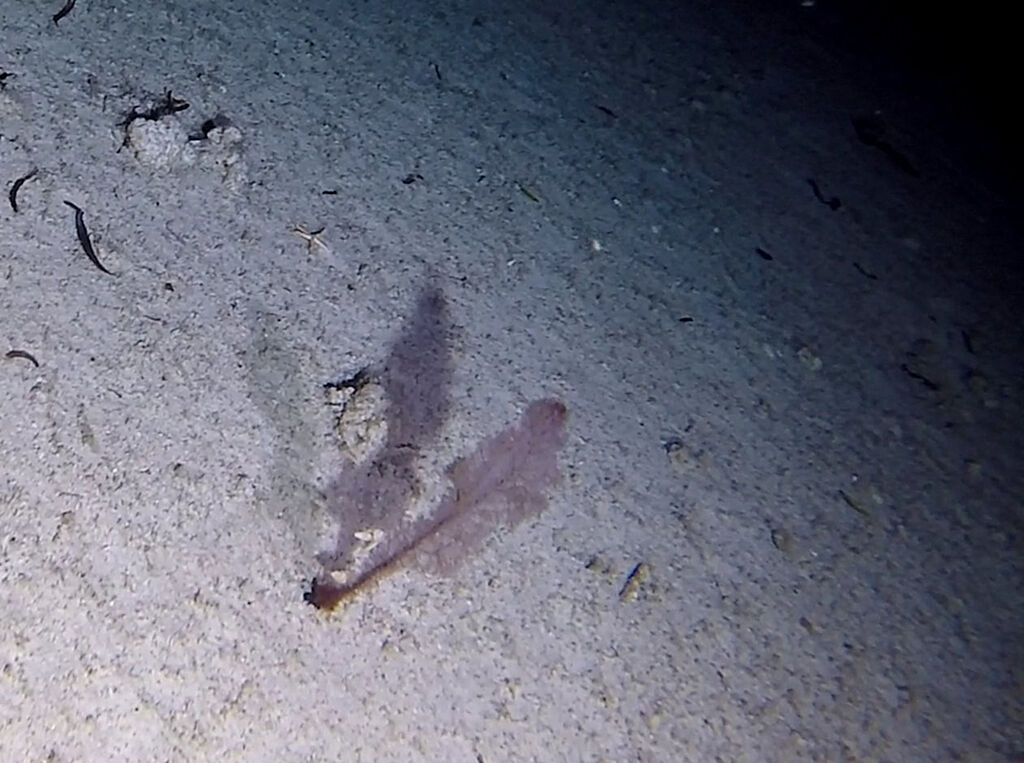
*Stylopathes* sp. indet. D'Arros N1, 350 m.

Colonies up to 50 cm in height, columnar, monopodial or very sparsely branched. Thin bottlebrush-like appearance. Irregularly pinnulate. Whitish to pink colouration. Darker coloured central axis enclosed by bushy, lighter coloured branches and polyps. Appears similar to *Cupressopathes*, but the latter has a much more pronounced bottlebrush appearance (Fig. 26).

### Octocorallia

#### Alcyonacea

Lamouroux, 1812

B6A5A5DD-F34D-51D9-80AC-C2ED2B7F778A

#### Acanthogorgiidae

Gray, 1859

CED044BB-CE9A-5827-9A42-EA134CA054E5

#### Muricella

Verrill, 1868

7EBF669C-836F-5995-853A-2A97BC443B6E

#### Muricella sp. indet.

7B9843D3-8034-56BF-9A9E-46394FE0ED8C

##### Materials

- **Type status:**Other material. **Taxon:** scientificName: *Muricella*; kingdom: Animalia; phylum: Cnidaria; class: Anthozoa; order: Alcyonacea; family: Acanthogorgiidae; genus: Muricella; scientificNameAuthorship: Verrill, 1868; **Location:** waterBody: Indian Ocean; country: Seychelles; locality: Aldabra N1, Alphonse N1, Desroches S1; minimumDepthInMeters: 44 m; maximumDepthInMeters: 117 m; locationRemarks: First Descent: Seychelles Expedition; **Identification:** identifiedBy: Nico Fassbender, Kaveh Samimi-Namin, Paris Stefanoudis; dateIdentified: 2019, 2020; identificationRemarks: identified only from imagery; **Event:** samplingProtocol: Submersible OR Remotely Operated Vehicle OR SCUBA; **Record Level:** basisOfRecord: Human observation

##### Notes

Colonies up to 1 m in height, fan-shaped and uniplanar with a high degree of ‘anastomosis’ (branch joins), giving the colonies a net-like appearance. In larger colonies, smaller branches may grow perpendicular to the main plane. Colour bright green to yellow. Occasionally with crinoid commensals (Fig. 27).

#### Alcyoniidae

Lamouroux, 1812

4BCF692A-6A4F-5C49-9A19-FE79C0E549DD

#### Lobophytum

Marenzeller, 1886

3336A62C-F14C-5E66-83B7-EF1E0C0DFA06

#### Lobophytum sp. indet.

40E35005-ECDB-5F9A-A538-9FE09075B305

##### Materials

- **Type status:**Other material. **Taxon:** scientificName: *Lobophytum*; kingdom: Animalia; phylum: Cnidaria; class: Anthozoa; order: Alcyonacea; family: Alcyoniidae; genus: Lobophytum; scientificNameAuthorship: Marenzeller, 1886; **Location:** waterBody: Indian Ocean; country: Seychelles; locality: Aldabra N1, Aldabra W1, Alphonse N1, D'Arros N1, Desroches S1, Poivre E1; minimumDepthInMeters: 8.8 m; maximumDepthInMeters: 36.3 m; locationRemarks: First Descent: Seychelles Expedition; **Identification:** identifiedBy: Nico Fassbender, Kaveh Samimi-Namin, Paris Stefanoudis; dateIdentified: 2019, 2020; identificationRemarks: identified only from imagery; **Event:** samplingProtocol: Submersible OR Remotely Operated Vehicle OR SCUBA; **Record Level:** basisOfRecord: Human observation

##### Notes

Colonies thickly encrusted with lobed projections, typically < 50 cm across (= in the longest dimension). Some species are bowl-shaped or stand more erect. Generally following the substrate, colonies look like large plates. Some form small individual bumps (1), others have long valleys and walls (2). Polyps are only present on the upper surface. Colouration brown to grey. The tips of the individual lobes are often coloured lighter than the sides. Similar species include *Pectinia*, which has deeper valleys towards the centre of the colony. *Sinularia* looks similar, but has smaller gaps between bumps (Fig. 28).

#### Paraminabea

Williams & Alderslade, 1999

670CCBD0-C19D-5D86-B03C-CE2EA902BFC9

#### Paraminabea sp. indet.

A9E8B9A9-5E9B-5FFB-A36C-4706AF41D418

##### Materials

- **Type status:**Other material. **Taxon:** scientificName: *Paraminabea*; kingdom: Animalia; phylum: Cnidaria; class: Anthozoa; order: Alcyonacea; family: Alcyoniidae; genus: Paraminabea; scientificNameAuthorship: Williams & Alderslade, 1999; **Location:** waterBody: Indian Ocean; country: Seychelles; locality: Aldabra W1, Alphonse N1; minimumDepthInMeters: 123 m; maximumDepthInMeters: 128 m; locationRemarks: First Descent: Seychelles Expedition; **Identification:** identifiedBy: Nico Fassbender, Kaveh Samimi-Namin, Paris Stefanoudis; dateIdentified: 2019, 2020; identificationRemarks: identified only from imagery; **Event:** samplingProtocol: Submersible OR Remotely Operated Vehicle OR SCUBA; **Record Level:** basisOfRecord: Human observation

##### Notes

Colonies are digitiform, with a short tapered stalk resembling a carrot, up to 10 cm long. Polyps are only extended at night. Colouration yellow, orange or red, with white polyps (Fig. 29).

#### Sarcophyton

Lesson, 1834

69551CF9-DAB2-5E00-B474-C86EDA49660D

#### Sarcophyton sp. indet.

9D9A474F-9354-5067-8C58-13F7DEEF5F55

##### Materials

- **Type status:**Other material. **Taxon:** scientificName: *Sarcophyton*; kingdom: Animalia; phylum: Cnidaria; class: Anthozoa; order: Alcyonacea; family: Alcyoniidae; genus: Sarcophyton; scientificNameAuthorship: Lesson, 1834; **Location:** waterBody: Indian Ocean; country: Seychelles; locality: Aldabra N1, Aldabra W1, D'Arros N1, Desroches S1, Poivre E1; minimumDepthInMeters: 8.8 m; maximumDepthInMeters: 36.3 m; locationRemarks: First Descent: Seychelles Expedition; **Identification:** identifiedBy: Nico Fassbender, Kaveh Samimi-Namin, Paris Stefanoudis; dateIdentified: 2019, 2020; identificationRemarks: identified only from imagery; **Event:** samplingProtocol: Submersible OR Remotely Operated Vehicle OR SCUBA; **Record Level:** basisOfRecord: Human observation

##### Notes

Colonies are lobate with conspicuous bare stalks merging into a wide, fleshy, disc-like head (polypary). The polypary is concave at the centre and wavy around the edges, giving it a mushroom appearance (especially in juveniles). Polyps are only found on top of the polypary. Colouration shades of brown, beige, yellow or green. Polyps are generally of the same colour as the colonies, but can be yellow or white in brown individuals. In downward facing videos typical for benthic surveys, the stalk will not always be visible. Maximum recorded size: 20 cm across. Similar species include *Lobophytum*, lacking the prominent stalk and the folds around the periphery (Fig. 30).

#### Sinularia

May, 1898

DBED3BC9-3C5B-5836-8B15-9F4C29B07DC7

#### Sinularia sp. indet.

9B138899-22C9-5856-980D-8CB4373B2D02

##### Materials

- **Type status:**Other material. **Taxon:** scientificName: *Sinularia*; kingdom: Animalia; phylum: Cnidaria; class: Anthozoa; order: Alcyonacea; family: Alcyoniidae; genus: Sinularia; scientificNameAuthorship: May, 1898; **Location:** waterBody: Indian Ocean; country: Seychelles; locality: Aldabra N1, Aldabra W1, Alphonse N1, D'Arros N1, Desroches S1, Poivre E1; minimumDepthInMeters: 8.8 m; maximumDepthInMeters: 39.4 m; locationRemarks: First Descent: Seychelles Expedition; **Identification:** identifiedBy: Nico Fassbender, Kaveh Samimi-Namin, Paris Stefanoudis; dateIdentified: 2019, 2020; identificationRemarks: identified only from imagery; **Event:** samplingProtocol: Submersible OR Remotely Operated Vehicle OR SCUBA; **Record Level:** basisOfRecord: Human observation

##### Notes

*Sinularia* colonies have the largest morphological variation amongst all soft corals. Colonies form low tabular mounds that can have ridged or digitate surfaces. Growth forms can be low encrusting, branching, tall and lobed,or lead and dish-like. Colonies form finger-like projections. Polyps are fully retractable. In this survey, colonies were typically < 50 cm across and their colouration ranged from grey and pale brown to pinkish and green. Similar species include *Cladiella* and *Lobophytum*, with *C.* and *L.* colonies typically having wider ridges between lobes (Fig. 31).

#### Anthothelidae

Broch, 1916

CABFA5A8-4BD7-54E9-BF6D-D3B1D6699D4F

#### Solenocaulon

Gray, 1862

5A375A7F-EB6B-59B0-808D-2D05E0673248

#### Solenocaulon sp. indet.

79E7C259-2588-560E-A40A-85E718474A3E

##### Materials

- **Type status:**Other material. **Taxon:** scientificName: *Solenocaulon*; kingdom: Animalia; phylum: Cnidaria; class: Anthozoa; order: Alcyonacea; family: Anthothelidae; genus: Solenocaulon; scientificNameAuthorship: Gray, 1862; **Location:** waterBody: Indian Ocean; country: Seychelles; locality: Aldabra N1, Aldabra W1, D'Arros N1, Desroches S1; minimumDepthInMeters: 21 m; maximumDepthInMeters: 72 m; locationRemarks: First Descent: Seychelles Expedition; **Identification:** identifiedBy: Nico Fassbender, Kaveh Samimi-Namin, Paris Stefanoudis; dateIdentified: 2019, 2020; identificationRemarks: identified only from imagery; **Event:** samplingProtocol: Submersible OR Remotely Operated Vehicle OR SCUBA; **Record Level:** basisOfRecord: Human observation

##### Notes

Colonies were up to 40 cm in height (Fig. 32b) and identified by their irregular and uniplanar branches. Polyps are conspicuous and give the colony a fuzzy appearance. Colouration ranges from red or light brown to pink and yellow, the latter two commonly encountered during this survey. Polyps are normally whitish (Fig. 32).

#### Ellisellidae

Gray, 1859

94C683FB-A558-562F-B062-54497FCFCDF0

#### "fam. Ellisellidae" gen. indet. sp. 1

7C77FD19-3410-503B-856E-596D0F0971BE

##### Materials

- **Type status:**Other material. **Taxon:** scientificName: Ellisellidae sp. 1; kingdom: Animalia; phylum: Cnidaria; class: Anthozoa; order: Alcyonacea; family: Ellisellidae; scientificNameAuthorship: Gray, 1859; **Location:** waterBody: Indian Ocean; country: Seychelles; locality: Alphonse N1, Astove W1, D'Arros N1, Poivre E1; minimumDepthInMeters: 52.9 m; maximumDepthInMeters: 120.7 m; locationRemarks: First Descent: Seychelles Expedition; **Identification:** identifiedBy: Nico Fassbender, Kaveh Samimi-Namin, Paris Stefanoudis; dateIdentified: 2019, 2020; identificationRemarks: identified only from imagery; **Event:** samplingProtocol: Submersible OR Remotely Operated Vehicle OR SCUBA; **Record Level:** basisOfRecord: Human observation

##### Notes

**Figure 33.**
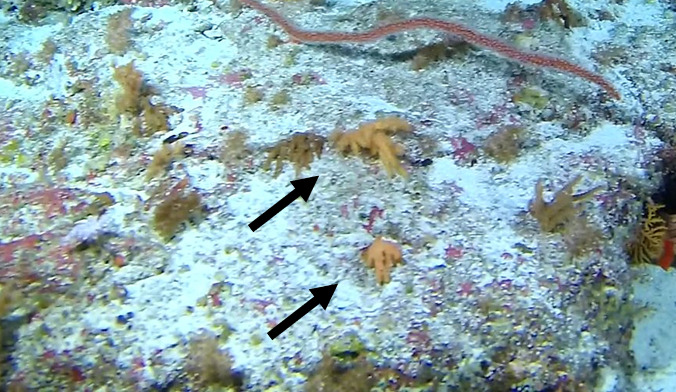
Ellisellidae gen. indet. sp. 1. Alphonse N1, 103 m.

Colonies are small (< 25 cm in height) and growing as stubby, finger-shaped branches. No central stalk was visible on the captured footage. Surface covered with polyps giving it a fuzzy appearance. Colony colour was orange with orange polyps (Fig. 33).

#### "fam. Ellisellidae" gen. indet. sp. 2

DCCDC676-CE70-5C42-BD2E-4A36E431A8DD

##### Materials

- **Type status:**Other material. **Taxon:** scientificName: Ellisellidae sp. 2; kingdom: Animalia; phylum: Cnidaria; class: Anthozoa; order: Alcyonacea; family: Ellisellidae; scientificNameAuthorship: Gray, 1859; **Location:** waterBody: Indian Ocean; country: Seychelles; locality: Aldabra N1, Aldabra W1, Alphonse N1, Astove W1, D'Arros N1, Desroches S1, Poivre E1; minimumDepthInMeters: 19.2 m; maximumDepthInMeters: 351 m; locationRemarks: First Descent: Seychelles Expedition; **Identification:** identifiedBy: Nico Fassbender, Kaveh Samimi-Namin, Paris Stefanoudis; dateIdentified: 2019, 2020; identificationRemarks: identified only from imagery; **Event:** samplingProtocol: Submersible OR Remotely Operated Vehicle OR SCUBA; **Record Level:** basisOfRecord: Human observation

##### Notes

Individuals are whip-like, forming single, unbranched colonies growing up to 2 m in height. Distal parts can be straight, heavily bent or coiled. Colour ranges from red to orange, pink, white, orange-yellow with red polyps or red with white polyps. Individuals most likely belong to the genus *Junceella* or *Viminella*, but are impossible to distinguish from video footage alone, as identification features might be extremely small and not visible on video footage. However, some might be unbranched colonies of *Ellisella* or uncoiled colonies of the black wire coral *Stichopathes* (Fig. 34).

#### Dichotella

Gray, 1870

D7EBF742-9005-52A4-960A-D968C9D6F236

#### Dichotella sp. indet.

FCAF5D16-2193-5B9E-9679-52F91D21EC7C

##### Materials

- **Type status:**Other material. **Taxon:** scientificName: *Dichotella*; kingdom: Animalia; phylum: Cnidaria; class: Anthozoa; order: Alcyonacea; family: Ellisellidae; genus: Dichotella; scientificNameAuthorship: Gray, 1870; **Location:** waterBody: Indian Ocean; country: Seychelles; locality: D'Arros N1; minimumDepthInMeters: 62.1 m; maximumDepthInMeters: 71.4 m; locationRemarks: First Descent: Seychelles Expedition; **Identification:** identifiedBy: Nico Fassbender, Kaveh Samimi-Namin, Paris Stefanoudis; dateIdentified: 2019, 2020; identificationRemarks: identified only from imagery; **Event:** samplingProtocol: Submersible OR Remotely Operated Vehicle OR SCUBA; **Record Level:** basisOfRecord: Human observation

##### Notes

**Figure 35.**
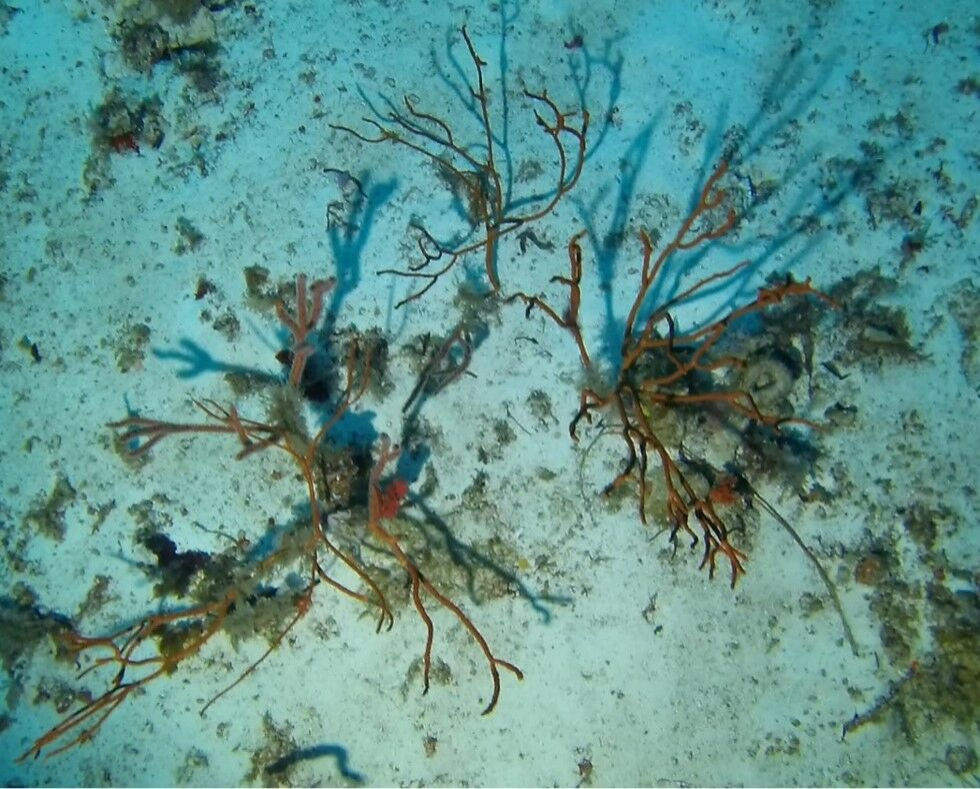
*Dichotella* sp. indet. D'Arros N1, 60 m.

Colonies with sparse or rich dichotomous branching - in this survey, the sparsely branched form, seen in Fig. 35 below, was more common. Colonies can appear bushy to planar. The maximum recorded colony size was 50 cm in height. Branches are thick and relatively short and split into smaller branches towards the periphery of the colony. Colouration is red to orange. *Ellisella* looks similar, but its colonies branch from the bottom and branches tend to be longer and whip-like (Fig. 35).

#### Ellisella

Gray, 1858

3BCAC51B-8911-57AF-B154-5796BD6EB180

#### Ellisella sp. indet.

6F6146BC-9763-5C82-8866-52FAEA6A380A

##### Materials

- **Type status:**Other material. **Taxon:** scientificName: *Ellisella*; kingdom: Animalia; phylum: Cnidaria; class: Anthozoa; order: Alcyonacea; family: Ellisellidae; genus: Ellisella; scientificNameAuthorship: Gray, 1858; **Location:** waterBody: Indian Ocean; country: Seychelles; locality: Aldabra N1, Aldabra W1, Astove W1, D'Arros N1, Desroches S1, Poivre E1; minimumDepthInMeters: 30 m; maximumDepthInMeters: 250 m; locationRemarks: First Descent: Seychelles Expedition; **Identification:** identifiedBy: Nico Fassbender, Kaveh Samimi-Namin, Paris Stefanoudis; dateIdentified: 2019, 2020; identificationRemarks: identified only from imagery; **Event:** samplingProtocol: Submersible OR Remotely Operated Vehicle OR SCUBA; **Record Level:** basisOfRecord: Human observation

##### Notes

Colonies up to 2 m in height, bush-like, with whip-like branches; branching can range from sparse to densely packed. Colouration is red to orange, pink, white, orange-yellow with red or white polyps. Similar-looking species include *Dichotella*, which in general has thicker and shorter branches that display dichotomous branching. *Dichotella* branches also become shorter towards the periphery of the colony (Fig. 36).

#### Nicella

Gray, 1870

517A0DFF-8C90-552E-BA93-561E67C1AF39

#### Nicella sp. indet.

ECA1743C-FD05-50C1-97D7-7F2892B92FA5

##### Materials

- **Type status:**Other material. **Taxon:** scientificName: *Nicella*; kingdom: Animalia; phylum: Cnidaria; class: Anthozoa; order: Alcyonacea; family: Ellisellidae; genus: Nicella; scientificNameAuthorship: Gray, 1870; **Location:** waterBody: Indian Ocean; country: Seychelles; locality: Aldabra N1, Aldabra W1, Astove W1, Desroches S1; minimumDepthInMeters: 58.6 m; maximumDepthInMeters: 148.1 m; locationRemarks: First Descent: Seychelles Expedition; **Identification:** identifiedBy: Nico Fassbender, Kaveh Samimi-Namin, Paris Stefanoudis; dateIdentified: 2019, 2020; identificationRemarks: identified only from imagery; **Event:** samplingProtocol: Submersible OR Remotely Operated Vehicle OR SCUBA; **Record Level:** basisOfRecord: Human observation

##### Notes

Colonies up to 1.5 m in height, fan-shaped, with sparse, fine branches typically growing in one plane. Colonies sometimes show dichotomous branching and never show anastomoses. Branching starts from the bottom, hence, the stalk is rarely visible. Colour white with dark-brown to black coloured polyps (Fig. 37)

#### Verrucella

Milne Edwards & Haime, 1857

BE208304-F32C-586C-8259-557CE17CCB63

#### Verrucella sp. indet.

17435BAE-5D7F-5565-A982-4B3531B5E596

##### Materials

- **Type status:**Other material. **Taxon:** scientificName: *Verrucella*; kingdom: Animalia; phylum: Cnidaria; class: Anthozoa; order: Alcyonacea; family: Ellisellidae; genus: Verrucella; scientificNameAuthorship: Milne Edwards & Haime, 1857; **Location:** waterBody: Indian Ocean; country: Seychelles; locality: Aldabra N1, Aldabra W1; minimumDepthInMeters: 21 m; maximumDepthInMeters: 64.5 m; locationRemarks: First Descent: Seychelles Expedition; **Identification:** identifiedBy: Nico Fassbender, Kaveh Samimi-Namin, Paris Stefanoudis; dateIdentified: 2019, 2020; identificationRemarks: identified only from imagery; **Event:** samplingProtocol: Submersible OR Remotely Operated Vehicle OR SCUBA; **Record Level:** basisOfRecord: Human observation

##### Notes

Large sea fans (~ 1 m in height and width), with dense, uniplanar branches that create a mesh-like appearance; conspicuous central stalk. Colouration observed here was exclusively purple; however red, orange, yellow and shades of brown are also common. More sparsely branched colonies can resemble *Nicella* (Fig. 38).

#### Gorgoniidae

Lamouroux, 1812

3B4544A0-C998-5D5E-A78F-716C337A5936

#### Rumphella

Bayer, 1955

B117F5AD-A14E-5F1F-9A7F-0885BBB8820D

#### Rumphella sp. indet.

95CE22BF-236A-56D2-8472-50564E413C72

##### Materials

- **Type status:**Other material. **Taxon:** scientificName: *Rumphella*; kingdom: Animalia; phylum: Cnidaria; class: Anthozoa; order: Alcyonacea; family: Gorgoniidae; genus: Rumphella; scientificNameAuthorship: Bayer, 1955; **Location:** waterBody: Indian Ocean; country: Seychelles; locality: Desroches S1; minimumDepthInMeters: 11.3 m; maximumDepthInMeters: 13 m; locationRemarks: First Descent: Seychelles Expedition; **Identification:** identifiedBy: Nico Fassbender, Kaveh Samimi-Namin, Paris Stefanoudis; dateIdentified: 2019, 2020; identificationRemarks: identified only from imagery; **Event:** samplingProtocol: Submersible OR Remotely Operated Vehicle OR SCUBA; **Record Level:** basisOfRecord: Human observation

##### Notes

**Figure 39.**
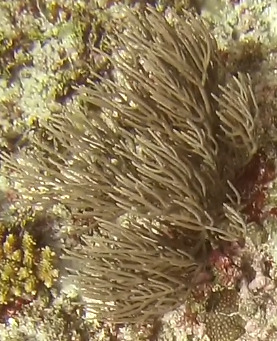
*Rumphella* sp. indet. Desroches S1, 11 m.

Colonies typically < 1 m across, appear bushy, with either sparse, whip-like branches or dense shrub-like branches that have a smooth surface and blunt tips. Light brown to greyish colour (Fig. 39).

#### Isididae

Lamouroux, 1812

2D5D7CB5-8351-559B-A5D8-3FB0B9C0D55E

#### Isis

Linnaeus, 1758

CD4D831D-934B-513A-9632-4A092E764CB2

#### Isis sp. indet.

6C1BEF6D-D254-5BE1-90E8-E9B23887262E

##### Materials

- **Type status:**Other material. **Taxon:** scientificName: *Isis*; kingdom: Animalia; phylum: Cnidaria; class: Anthozoa; order: Alcyonacea; family: Isididae; genus: Isis; scientificNameAuthorship: Lamouroux, 1812; **Location:** waterBody: Indian Ocean; country: Seychelles; locality: Alphonse N1, D'Arros N1; minimumDepthInMeters: 89 m; maximumDepthInMeters: 95 m; locationRemarks: First Descent: Seychelles Expedition; **Identification:** identifiedBy: Nico Fassbender, Kaveh Samimi-Namin, Paris Stefanoudis; dateIdentified: 2019, 2020; identificationRemarks: identified only from imagery; **Event:** samplingProtocol: Submersible OR Remotely Operated Vehicle OR SCUBA; **Record Level:** basisOfRecord: Human observation

##### Notes

Colonies up to 40 cm in height, can appear fan or bush-like, with thick branches covered in fuzzy-looking polyps. Colour ranges from yellow to green or brownish (Fig. 40).

#### Melithaeidae

Gray, 1870

0DF9B224-CE1C-5AC9-ACDE-BF6612A6F5E7

#### "fam. Melithaeidae" gen. indet. sp. 1

BD44E990-A40F-55F0-A48C-A8BAA4934A27

##### Materials

- **Type status:**Other material. **Taxon:** scientificName: Melithaeidae sp. 1; kingdom: Animalia; phylum: Cnidaria; class: Anthozoa; order: Alcyonacea; family: Melithaeidae; scientificNameAuthorship: Gray, 1870; **Location:** waterBody: Indian Ocean; country: Seychelles; locality: D'Arros N1, Desroches S1, Poivre E1; minimumDepthInMeters: 36.2 m; maximumDepthInMeters: 71.5 m; locationRemarks: First Descent: Seychelles Expedition; **Identification:** identifiedBy: Nico Fassbender, Kaveh Samimi-Namin, Paris Stefanoudis; dateIdentified: 2019, 2020; identificationRemarks: identified only from imagery; **Event:** samplingProtocol: Submersible OR Remotely Operated Vehicle OR SCUBA; **Record Level:** basisOfRecord: Human observation

##### Notes

Colonies up to 20 cm in height, mostly fan-shaped and uniplanar, sometimes slightly bushy. Mostly dichotomously branched, where branches originate from the nodes. Colouration rich purple-reddish colour at the base that becomes lighter towards the tips of the branches. The centre of the colony always appears darker than the edges (Fig. 41).

#### "fam. Melithaeidae" gen. indet. sp. 2

0A3D9ACA-2474-5504-A3F0-F3233096D5A0

##### Materials

- **Type status:**Other material. **Taxon:** scientificName: Melithaeidae sp. 2; kingdom: Animalia; phylum: Cnidaria; class: Anthozoa; order: Alcyonacea; family: Melithaeidae; scientificNameAuthorship: Gray, 1870; **Location:** waterBody: Indian Ocean; country: Seychelles; locality: Aldabra N1; minimumDepthInMeters: 120 m; maximumDepthInMeters: 120 m; locationRemarks: First Descent: Seychelles Expedition; **Identification:** identifiedBy: Nico Fassbender, Kaveh Samimi-Namin, Paris Stefanoudis; dateIdentified: 2019, 2020; identificationRemarks: identified only from imagery; **Event:** samplingProtocol: Submersible OR Remotely Operated Vehicle OR SCUBA; **Record Level:** basisOfRecord: Human observation

##### Notes

Colonies up to 60 cm in height, mostly fan-shaped, multiplanar with irregular branching. The periphery of the colony is rather sinuous. The main stem has a distinct orange colour with additional branches becoming successively lighter; tips of the branches appear almost white (Fig. 42).

#### "fam. Melithaeidae" gen. indet. sp. 3

F8D6379A-3D9C-5F72-96B1-CFBF611FCECB

##### Materials

- **Type status:**Other material. **Taxon:** scientificName: Melithaeidae sp. 3; kingdom: Animalia; phylum: Cnidaria; class: Anthozoa; order: Alcyonacea; family: Melithaeidae; scientificNameAuthorship: Gray, 1870; **Location:** waterBody: Indian Ocean; country: Seychelles; locality: Astove W1; minimumDepthInMeters: 49.1 m; maximumDepthInMeters: 63.4 m; locationRemarks: First Descent: Seychelles Expedition; **Identification:** identifiedBy: Nico Fassbender, Kaveh Samimi-Namin, Paris Stefanoudis; dateIdentified: 2019, 2020; identificationRemarks: identified only from imagery; **Event:** samplingProtocol: Submersible OR Remotely Operated Vehicle OR SCUBA; **Record Level:** basisOfRecord: Human observation

##### Notes

Colonies less than 20 cm in height, arborescent with dichotomous branching; sometimes appear slightly bushy when heavily branched. Colour deep purple with slightly lighter polyps (Fig. 43).

#### Nephtheidae

Gray, 1862

97D79B23-2B13-5CE3-89EC-B53499E662ED

#### Dendronephthya

Kükenthal, 1905

6423094A-4683-5912-867D-B94B5DB5B353

#### Dendronephthya sp. indet. 1

222B1ABD-12F7-5C78-9416-B2B0FA4BD929

##### Materials

- **Type status:**Other material. **Taxon:** scientificName: *Dendronephthya* sp. 1; kingdom: Animalia; phylum: Cnidaria; class: Anthozoa; order: Alcyonacea; family: Nephtheidae; genus: Dendronephthya; scientificNameAuthorship: Kükenthal, 1905; **Location:** waterBody: Indian Ocean; country: Seychelles; locality: Aldabra W1, D'Arros N1, Desroches S1, Poivre E1; minimumDepthInMeters: 85 m; maximumDepthInMeters: 123.4 m; locationRemarks: First Descent: Seychelles Expedition; **Identification:** identifiedBy: Nico Fassbender, Kaveh Samimi-Namin, Paris Stefanoudis; dateIdentified: 2019, 2020; identificationRemarks: identified only from imagery; **Event:** samplingProtocol: Submersible OR Remotely Operated Vehicle OR SCUBA; **Record Level:** basisOfRecord: Human observation

##### Notes

Bushy colonies up to 20 cm in height, with close, short branching and distinct, large, round polyp bunches at the end of each branch. Colonies can show one of three growth forms: divaricate (sparse, arborescent branching with bundled polyps), glomerate (close, short branching with polyps forming rounded bunches) or umbellate (polyps forming umbrella-like crowns that may combine to form hemispheres). White stalk with polyps of red, orange, purple, yellow, pink or white colour (Fig. 44).

#### Dendronephthya sp. indet. 2

78671234-F5BF-51A9-8283-E854333B6F01

##### Materials

- **Type status:**Other material. **Taxon:** scientificName: *Dendronephthya* sp. 2; kingdom: Animalia; phylum: Cnidaria; class: Anthozoa; order: Alcyonacea; family: Nephtheidae; genus: Dendronephthya; scientificNameAuthorship: Kükenthal, 1905; **Location:** waterBody: Indian Ocean; country: Seychelles; locality: Desroches S1, Poivre E1; minimumDepthInMeters: 20 m; maximumDepthInMeters: 120 m; locationRemarks: First Descent: Seychelles Expedition; **Identification:** identifiedBy: Nico Fassbender, Kaveh Samimi-Namin, Paris Stefanoudis; dateIdentified: 2019, 2020; identificationRemarks: identified only from imagery; **Event:** samplingProtocol: Submersible OR Remotely Operated Vehicle OR SCUBA; **Record Level:** basisOfRecord: Human observation

##### Notes

Colonies umbellate up to 20 cm in height, with polyp bunches closely arranged at the same level on the end of small branches (twigs), forming umbrella-like crowns. Creamy-white colour (Fig. 45).

#### Litophyton

Forskål, 1775

24008263-71AB-5D11-8327-CA47581B1AF1

#### Litophyton sp. indet.

90D8F957-2E27-527E-A496-25FAB1282724

##### Materials

- **Type status:**Other material. **Taxon:** scientificName: *Litophyton*; kingdom: Animalia; phylum: Cnidaria; class: Anthozoa; order: Alcyonacea; family: Nephtheidae; genus: Litophyton; scientificNameAuthorship: Forskĺl, 1775; **Location:** waterBody: Indian Ocean; country: Seychelles; locality: Aldabra N1, Aldabra W1, Alphonse N1, Astove W1, D'Arros N1, Desroches S1, Poivre E1; minimumDepthInMeters: 58.6 m; maximumDepthInMeters: 148.1 m; locationRemarks: First Descent: Seychelles Expedition; **Identification:** identifiedBy: Nico Fassbender, Kaveh Samimi-Namin, Paris Stefanoudis; dateIdentified: 2019, 2020; identificationRemarks: identified only from imagery; **Event:** samplingProtocol: Submersible OR Remotely Operated Vehicle OR SCUBA; **Record Level:** basisOfRecord: Human observation

##### Notes

Colonies are tree-like with branched polyparium, growing out from one single stem, growing up to 55 cm in height. Polyps are non-retractile and clustered at the end of the terminal branches, forming catkins. Colouration orange to yellow, cream, brown or purple. *Litophyton* can be confused with the similar-looking *Nepthea*. They can be distinguished by their general appearance, with *Litophyton* being very soft compared to the firm *Nepthea* (Fig. 46).

#### Scleronephthya

Studer, 1887

356976EF-81DE-5195-AE2B-CFE4EC2BBDAB

#### Scleronephthya sp. indet.

39A23A9B-38DB-532F-902E-A4715710A953

##### Materials

- **Type status:**Other material. **Taxon:** scientificName: *Scleronephthya* sp.; kingdom: Animalia; phylum: Cnidaria; class: Anthozoa; order: Alcyonacea; family: Nephtheidae; genus: Scleronephthya; scientificNameAuthorship: Studer, 1887; **Location:** waterBody: Indian Ocean; country: Seychelles; locality: Aldabra N1, Aldabra W1, D'Arros N1, Desroches S1; minimumDepthInMeters: 32 m; maximumDepthInMeters: 120.7 m; locationRemarks: First Descent: Seychelles Expedition; **Identification:** identifiedBy: Nico Fassbender, Kaveh Samimi-Namin, Paris Stefanoudis; dateIdentified: 2019, 2020; identificationRemarks: identified only from imagery; **Event:** samplingProtocol: Submersible OR Remotely Operated Vehicle OR SCUBA; **Record Level:** basisOfRecord: Human observation

##### Notes

Colonies up to 25 cm in height, sparsely branched and arborescent, often planar. Polyps only on the branched part of the colony and are normally expanded at night and in strong currents. Colouration translucent-white with bluish-purple polyps. Can be confused with *Dendronephthya*, which has a similar appearance, but its polyps typically form bunches that cover the entire colony surface (Fig. 47).

#### Nidaliidae

Gray, 1869

3E02287E-B157-5C47-92E3-0D247AF01A2B

#### "fam. Nidaliidae" gen. indet. sp.

6239F658-0859-5A13-94E3-45BD23D710BF

##### Materials

- **Type status:**Other material. **Taxon:** scientificName: Nidaliidae sp. 1; kingdom: Animalia; phylum: Cnidaria; class: Anthozoa; order: Alcyonacea; family: Nidaliidae; scientificNameAuthorship: Gray, 1869; **Location:** waterBody: Indian Ocean; country: Seychelles; locality: Aldabra N1, Alphonse N1, Astove W1, D'Arros N1, Desroches S1; minimumDepthInMeters: 65.6 m; maximumDepthInMeters: 148.1 m; locationRemarks: First Descent: Seychelles Expedition; **Identification:** identifiedBy: Nico Fassbender, Kaveh Samimi-Namin, Paris Stefanoudis; dateIdentified: 2019, 2020; identificationRemarks: identified only from imagery; **Event:** samplingProtocol: Submersible OR Remotely Operated Vehicle OR SCUBA; **Record Level:** basisOfRecord: Human observation

##### Notes

**Figure 48.**
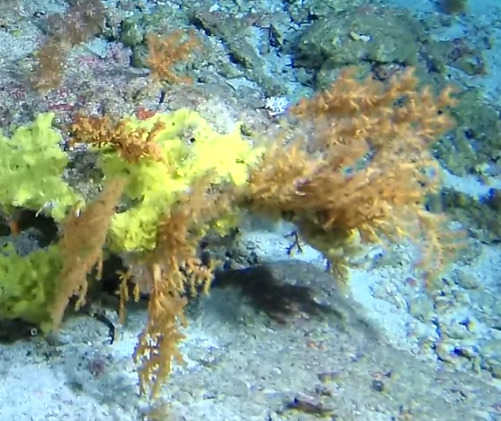
Nidaliidae gen. indet. sp. Alphonse N1, 250 m.

Colonies up to 50 cm in height, typically heavily branched with a bushy appearance. Colouration orange-brown with similar coloured polyps (Fig. 48).

#### Plexauridae

Gray, 1859

B6B54ED2-DCB2-5256-BD3D-30004649464E

#### "fam. Plexauridae" gen. indet. sp. 2

1F4C9615-C0A3-5FDA-A686-7564A27DA8D6

##### Materials

- **Type status:**Other material. **Taxon:** scientificName: Plexauridae sp. 2; kingdom: Animalia; phylum: Cnidaria; class: Anthozoa; order: Alcyonacea; family: Plexauridae; scientificNameAuthorship: Gray, 1859; **Location:** waterBody: Indian Ocean; country: Seychelles; locality: Aldabra N1, Aldabra W1, Alphonse N1, Astove W1, D'Arros N1, Desroches S1, Poivre E1; minimumDepthInMeters: 89 m; maximumDepthInMeters: 148.1 m; locationRemarks: First Descent: Seychelles Expedition; **Identification:** identifiedBy: Nico Fassbender, Kaveh Samimi-Namin, Paris Stefanoudis; dateIdentified: 2019, 2020; identificationRemarks: identified only from imagery; **Event:** samplingProtocol: Submersible OR Remotely Operated Vehicle OR SCUBA; **Record Level:** basisOfRecord: Human observation

##### Notes

Colonies fan-shaped, mostly uniplanar, with a thick main stem and several thinner branches. Overall, there is a strong tree-like resemblance with branches growing upwards. Anastomoses are never observed. Colony size typically < 50 cm in height but can occasionally reach > 1 m. The colour is bright green to yellow. Young colonies grow upright into branched stalks and do not yet have a fan morphology. Belongs to either *Paraplexaura* or *Paracis*, but positive identification requires microscopic examination. Colonies can be mistaken for *Acanthogorgia*, but the latter forms bushy colonies growing omnidirectionally (Fig. 49).

#### "fam. Plexauridae" gen. indet. sp. 4

87F15545-3C07-54FE-AF1D-71B5EBA427B3

##### Materials

- **Type status:**Other material. **Taxon:** scientificName: Plexauridae sp. 4; kingdom: Animalia; phylum: Cnidaria; class: Anthozoa; order: Alcyonacea; family: Plexauridae; scientificNameAuthorship: Gray, 1859; **Location:** waterBody: Indian Ocean; country: Seychelles; locality: Aldabra N1; minimumDepthInMeters: 100 m; maximumDepthInMeters: 100 m; locationRemarks: First Descent: Seychelles Expedition; **Identification:** identifiedBy: Nico Fassbender, Kaveh Samimi-Namin, Paris Stefanoudis; dateIdentified: 2019, 2020; identificationRemarks: identified only from imagery; **Event:** samplingProtocol: Submersible OR Remotely Operated Vehicle OR SCUBA; **Record Level:** basisOfRecord: Human observation

##### Notes

**Figure 50.**
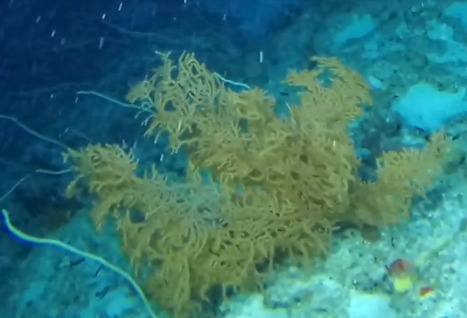
Plexauridae gen. indet. sp. 4. Aldabra N1, 100 m.

Colonies large (typically > 1 m in height), slightly bushy, with dichotomous to pinnate branching. Branches covered with small polyps and appear fuzzy. Colour shades of brown to greenish-grey (Fig. 50).

#### "fam. Plexauridae" gen. indet. sp. 5

4D3483E6-9006-5D6B-A0B9-69EDA8421F35

##### Materials

- **Type status:**Other material. **Taxon:** scientificName: Plexauridae sp. 5; kingdom: Animalia; phylum: Cnidaria; class: Anthozoa; order: Alcyonacea; family: Plexauridae; scientificNameAuthorship: Gray, 1859; **Location:** waterBody: Indian Ocean; country: Seychelles; locality: Aldabra W1, D'Arros N1, Desroches S1; minimumDepthInMeters: 58.6 m; maximumDepthInMeters: 67.9 m; locationRemarks: First Descent: Seychelles Expedition; **Identification:** identifiedBy: Nico Fassbender, Kaveh Samimi-Namin, Paris Stefanoudis; dateIdentified: 2019, 2020; identificationRemarks: identified only from imagery; **Event:** samplingProtocol: Submersible OR Remotely Operated Vehicle OR SCUBA; **Record Level:** basisOfRecord: Human observation

##### Notes

**Figure 51.**
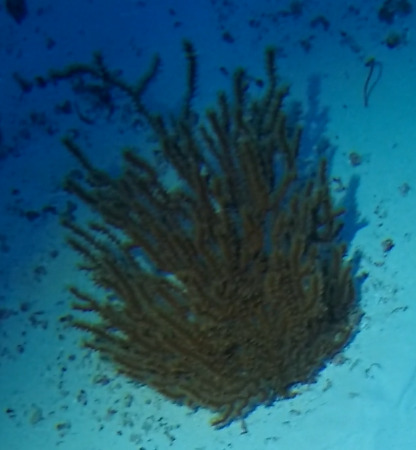
Plexauridae gen. indet. sp. 5. Desroches S1, 60 m.

Colonies up to 30 cm in height, uniplanar, branching dichotomously from the bottom of the colony with no clear mainstem visible. Conspicuously monodirectional upward branching pattern. Branches covered in small polyps and appear fuzzy. Dark brown with grey-brow polyps (Fig. 51).

#### "fam. Plexauridae" gen. indet. sp. 6

2CAD5F4C-8028-5EC2-B656-D130BA453A4D

##### Materials

- **Type status:**Other material. **Taxon:** scientificName: Plexauridae sp. 6; kingdom: Animalia; phylum: Cnidaria; class: Anthozoa; order: Alcyonacea; family: Plexauridae; scientificNameAuthorship: Gray, 1859; **Location:** waterBody: Indian Ocean; country: Seychelles; locality: Aldabra N1, Aldabra W1, Astove W1, Desroches S1; minimumDepthInMeters: 66.3 m; maximumDepthInMeters: 148.1 m; locationRemarks: First Descent: Seychelles Expedition; **Identification:** identifiedBy: Nico Fassbender, Kaveh Samimi-Namin, Paris Stefanoudis; dateIdentified: 2019, 2020; identificationRemarks: identified only from imagery; **Event:** samplingProtocol: Submersible OR Remotely Operated Vehicle OR SCUBA; **Record Level:** basisOfRecord: Human observation

##### Notes

Colonies up to 60 cm in height, fan-shaped, uniplanar with irregular branching. The stem is coloured brown with yellow to pale-grey polyps that tend to be more brightly coloured towards the end of the colony's branches (Fig. 52).

#### "fam. Plexauridae" gen. indet. sp. 7

65730FEC-EED1-57F9-80E0-786437B3A085

##### Materials

- **Type status:**Other material. **Taxon:** scientificName: Plexauridae sp. 7; kingdom: Animalia; phylum: Cnidaria; class: Anthozoa; order: Alcyonacea; family: Plexauridae; scientificNameAuthorship: Gray, 1859; **Location:** waterBody: Indian Ocean; country: Seychelles; locality: Aldabra N1, Astove W1; minimumDepthInMeters: 32 m; maximumDepthInMeters: 60 m; locationRemarks: First Descent: Seychelles Expedition; **Identification:** identifiedBy: Nico Fassbender, Kaveh Samimi-Namin, Paris Stefanoudis; dateIdentified: 2019, 2020; identificationRemarks: identified only from imagery; **Event:** samplingProtocol: Submersible OR Remotely Operated Vehicle OR SCUBA; **Record Level:** basisOfRecord: Human observation

##### Notes

Colonies up to 50 cm in height, bushy, with dense, short branches. Polyps are small, but numerous, giving the colony a fuzzy appearance. Dark brown to black with pale white to purple polyps (Fig. 53).

#### "fam. Plexauridae" gen. indet. sp. 8

29C47F72-FE10-52B4-B3B6-33193DE21352

##### Materials

- **Type status:**Other material. **Taxon:** scientificName: Plexauridae sp. 8; kingdom: Animalia; phylum: Cnidaria; class: Anthozoa; order: Alcyonacea; family: Plexauridae; scientificNameAuthorship: Gray, 1859; **Location:** waterBody: Indian Ocean; country: Seychelles; locality: Aldabra N1, Aldabra W1, D'Arros N1, Desroches S1; minimumDepthInMeters: 30 m; maximumDepthInMeters: 128 m; locationRemarks: First Descent: Seychelles Expedition; **Identification:** identifiedBy: Nico Fassbender, Kaveh Samimi-Namin, Paris Stefanoudis; dateIdentified: 2019, 2020; identificationRemarks: identified only from imagery; **Event:** samplingProtocol: Submersible OR Remotely Operated Vehicle OR SCUBA; **Record Level:** basisOfRecord: Human observation

##### Notes

Colonies small (typically ~ 10 cm in height), with sparse, dichotomous branching and a twig-like appearance. No visible polyp calices. Dark red to dark brown. *Astrogorgia* appears similar, but that genus has highly developed and conspicuous polyp calices (Fig. 54).

#### "fam. Plexauridae" gen. indet. sp. 9

5E738C66-6B5E-5D2A-8D7E-E46EF30DC6FB

##### Materials

- **Type status:**Other material. **Taxon:** scientificName: Plexauridae sp. 9; kingdom: Animalia; phylum: Cnidaria; class: Anthozoa; order: Alcyonacea; family: Plexauridae; scientificNameAuthorship: Gray, 1859; **Location:** waterBody: Indian Ocean; country: Seychelles; locality: Aldabra W1, Alphonse N1, Desroches S1; minimumDepthInMeters: 61.8 m; maximumDepthInMeters: 140.4 m; locationRemarks: First Descent: Seychelles Expedition; **Identification:** identifiedBy: Nico Fassbender, Kaveh Samimi-Namin, Paris Stefanoudis; dateIdentified: 2019, 2020; identificationRemarks: identified only from imagery; **Event:** samplingProtocol: Submersible OR Remotely Operated Vehicle OR SCUBA; **Record Level:** basisOfRecord: Human observation

##### Notes

Colonies up to 40 cm in height, fan-shaped, appearing mostly uniplanar and heavily branched. With conspicuous polyps giving the colony a fuzzy appearance. Multi-coloured with a pale white base, a dark-red to purple middle area and a bright yellow outer edge. This colouration is mostly well-developed in larger colonies (Fig. 55).

#### "fam. Plexauridae" gen. indet. sp. 11

F2AAF282-70E7-52CE-A81A-DC3CAA78605A

##### Materials

- **Type status:**Other material. **Taxon:** scientificName: Plexauridae sp. 11; kingdom: Animalia; phylum: Cnidaria; class: Anthozoa; order: Alcyonacea; family: Plexauridae; scientificNameAuthorship: Gray, 1859; **Location:** waterBody: Indian Ocean; country: Seychelles; locality: Alphonse N1; minimumDepthInMeters: 85 m; maximumDepthInMeters: 108 m; locationRemarks: First Descent: Seychelles Expedition; **Identification:** identifiedBy: Nico Fassbender, Kaveh Samimi-Namin, Paris Stefanoudis; dateIdentified: 2019, 2020; identificationRemarks: identified only from imagery; **Event:** samplingProtocol: Submersible OR Remotely Operated Vehicle OR SCUBA; **Record Level:** basisOfRecord: Human observation

##### Notes

Colonies up to 40 cm in height, fan-shaped to slightly bushy and heavily branched. Large polyps are giving the colony a fuzzy appearance. Purple to dark brown stem with yellow-orange polyps (Fig. 56).

#### "fam. Plexauridae" gen. indet. sp. 13

EA7ECBB5-AC0B-581C-BC98-2760B6152E1A

##### Materials

- **Type status:**Other material. **Taxon:** scientificName: Plexauridae sp. 13; kingdom: Animalia; phylum: Cnidaria; class: Anthozoa; order: Alcyonacea; family: Plexauridae; scientificNameAuthorship: Gray, 1859; **Location:** waterBody: Indian Ocean; country: Seychelles; locality: Aldabra W1; minimumDepthInMeters: 61.9; maximumDepthInMeters: 72 m; locationRemarks: First Descent: Seychelles Expedition; **Identification:** identifiedBy: Nico Fassbender, Kaveh Samimi-Namin, Paris Stefanoudis; dateIdentified: 2019, 2020; identificationRemarks: identified only from imagery; **Event:** samplingProtocol: Submersible OR Remotely Operated Vehicle OR SCUBA; **Record Level:** basisOfRecord: Human observation

##### Notes

**Figure 57.**
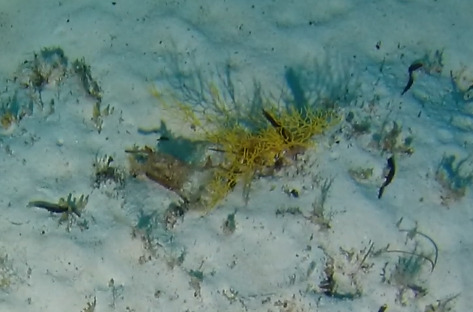
Plexauridae gen. indet. sp. 13. Aldabra W1, 72 m.

Colonies are short (~ 15 cm in height), irregularly branched, forming small bushes. Branches are thin and almost twig-like, giving an overall delicate and brittle appearance. The colouration is bright yellow. Observed to grow in sedimented habitats around 60 m. The species could be confused with *Plexauridae* sp. 2; however, the latter has thicker, fuzzier-looking branches and is found under overhangs and ledges in around 120 m of depth (Fig. 57).

#### "fam. Plexauridae" gen. indet. sp. 14

5FBC7734-1E89-5703-99E5-0F070DA237BD

##### Materials

- **Type status:**Other material. **Taxon:** scientificName: Plexauridae sp. 14; kingdom: Animalia; phylum: Cnidaria; class: Anthozoa; order: Alcyonacea; family: Plexauridae; scientificNameAuthorship: Gray, 1859; **Location:** waterBody: Indian Ocean; country: Seychelles; locality: Aldabra N1, Alphonse N1, D'Arros N1, Desroches S1; minimumDepthInMeters: 61.9 m; maximumDepthInMeters: 122 m; locationRemarks: First Descent: Seychelles Expedition; **Identification:** identifiedBy: Nico Fassbender, Kaveh Samimi-Namin, Paris Stefanoudis; dateIdentified: 2019, 2020; identificationRemarks: identified only from imagery; **Event:** samplingProtocol: Submersible OR Remotely Operated Vehicle OR SCUBA; **Record Level:** basisOfRecord: Human observation

##### Notes

Colonies up to 40 cm in height, displaying uniplanar to bushy branching. Conspicuous polyps give the colony a rather fuzzy appearance. The colouration is a pale yellow to brown, with the polyps slightly darker coloured than the colony main colour (Fig. 58).

#### Astrogorgia

Verrill, 1868

94ED3EC4-A21E-5EE7-8FFF-61CFA08C12F8

#### Astrogorgia sp. indet.

5437566D-557D-5758-8760-4A6549D44523

##### Materials

- **Type status:**Other material. **Taxon:** scientificName: *Astrogorgia*; kingdom: Animalia; phylum: Cnidaria; class: Anthozoa; order: Alcyonacea; family: Plexauridae; genus: Astrogorgia; scientificNameAuthorship: Verrill, 1868; **Location:** waterBody: Indian Ocean; country: Seychelles; locality: Aldabra N1, Aldabra W1, Astove W1, Desroches S1; minimumDepthInMeters: 30 m; maximumDepthInMeters: 148.1 m; locationRemarks: First Descent: Seychelles Expedition; **Identification:** identifiedBy: Nico Fassbender, Kaveh Samimi-Namin, Paris Stefanoudis; dateIdentified: 2019, 2020; identificationRemarks: identified only from imagery; **Event:** samplingProtocol: Submersible OR Remotely Operated Vehicle OR SCUBA; **Record Level:** basisOfRecord: Human observation

##### Notes

Colonies typically < 20 cm in height, but some up to 55 cm tall, growing as uniplanar, irregularly branched fans. Branches thin with large polyp calices giving the colony a spiky appearance. Anastomoses are never observed. Colour shades of red to brown with yellow polyps. Species of *Acanthogorgia* and *Muricella* can have similar growth forms; therefore particular attention should be paid to whether calices, the identifying feature of *Astrogorgia*, are present (Fig. 59).

#### Echinogorgia

Kölliker, 1865

4D037848-5F12-5A60-AA3E-393B58DB5EBA

#### Echinogorgia gen. inc.

7FCA77A4-2247-57A1-98FF-0DC7E9C027FD

##### Materials

- **Type status:**Other material. **Taxon:** scientificName: *Echinogorgia*; kingdom: Animalia; phylum: Cnidaria; class: Anthozoa; order: Alcyonacea; family: Plexauridae; genus: Echinogorgia; scientificNameAuthorship: Kölliker, 1865; **Location:** waterBody: Indian Ocean; country: Seychelles; locality: Aldabra N1, Aldabra W1, Alphonse N1, D;Arros N1; minimumDepthInMeters: 30 m; maximumDepthInMeters: 120 m; locationRemarks: First Descent: Seychelles Expedition; **Identification:** identifiedBy: Nico Fassbender, Kaveh Samimi-Namin, Paris Stefanoudis; dateIdentified: 2019, 2020; identificationRemarks: identified only from imagery; **Event:** samplingProtocol: Submersible OR Remotely Operated Vehicle OR SCUBA; **Record Level:** basisOfRecord: Human observation

##### Notes

Colonies up to 50 cm in height, with thin branches and uniplanar growth form, with side branches much shorter than main branches. Some degree of anastomoses should always be present. Polyp calices are conspicuous and give branches a bumpy appearance. Colonies are red-brown to grey, with one white individual recorded. The similar-looking *Muricella* may appear superficially similar in terms of colony shape, yet perpendicular branching should be visible compared to *Echinogorgia* (Fig. 60).

#### Paracis

Kükenthal, 1919

500A53C5-9637-52AE-8545-51FA24CAB073

#### Paracis gen. inc.

8BC03B5C-EE57-5F6B-BAF9-5A212FBFD4CF

##### Materials

- **Type status:**Other material. **Taxon:** scientificName: *Paracis* (cg.); kingdom: Animalia; phylum: Cnidaria; class: Anthozoa; order: Alcyonacea; family: Plexauridae; genus: Paracis; scientificNameAuthorship: Kükenthal, 1919; **Location:** waterBody: Indian Ocean; country: Seychelles; locality: Aldabra N1, Aldabra W1, Desroches S1; minimumDepthInMeters: 31.1 m; maximumDepthInMeters: 71.5 m; locationRemarks: First Descent: Seychelles Expedition; **Identification:** identifiedBy: Nico Fassbender, Kaveh Samimi-Namin, Paris Stefanoudis; dateIdentified: 2019, 2020; identificationRemarks: identified only from imagery; **Event:** samplingProtocol: Submersible OR Remotely Operated Vehicle OR SCUBA; **Record Level:** basisOfRecord: Human observation

##### Notes

**Figure 61.**
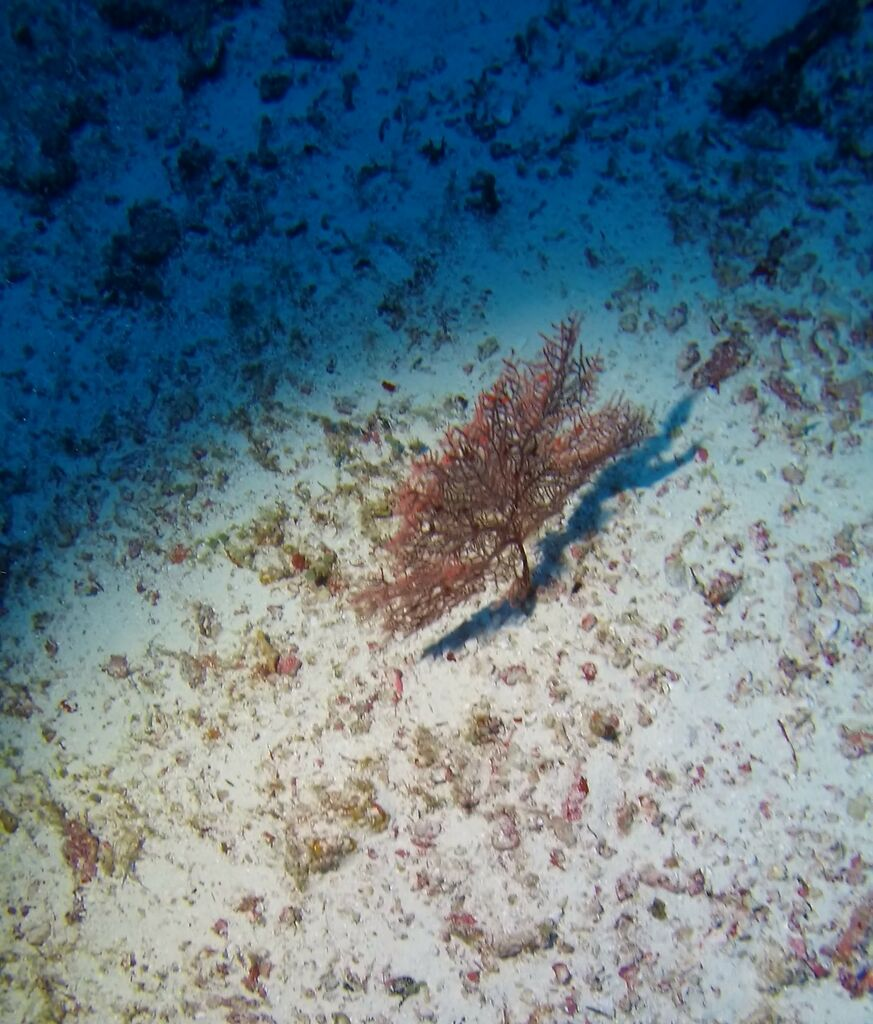
*Paracis* gen. inc. Aldabra N1, 60 m.

Colonies up to 60 cm in height, uniplanar and profusely branched. With a thick central stem and visibly thinner branches. Colour observed here was a distinctly bright red, with pink, yellow and pale-blue also common (Fig. 61).

#### Trimuricea

Gordon, 1926

0817BAB2-EF5C-5633-B121-5C4B890AB0EE

#### Trimuricea sp. indet.

45585402-230D-51C9-96EA-AF61B663539D

##### Materials

- **Type status:**Other material. **Taxon:** scientificName: *Trimuricea*; kingdom: Animalia; phylum: Cnidaria; class: Anthozoa; order: Alcyonacea; family: Plexauridae; genus: Trimuricea; scientificNameAuthorship: Gordon, 1926; **Location:** waterBody: Indian Ocean; country: Seychelles; locality: Aldabra N1, Aldabra W1, Astove W1, D'Arros N1; minimumDepthInMeters: 58.6 m; maximumDepthInMeters: 148.1 m; locationRemarks: First Descent: Seychelles Expedition; **Identification:** identifiedBy: Nico Fassbender, Kaveh Samimi-Namin, Paris Stefanoudis; dateIdentified: 2019, 2020; identificationRemarks: identified only from imagery; **Event:** samplingProtocol: Submersible OR Remotely Operated Vehicle OR SCUBA; **Record Level:** basisOfRecord: Human observation

##### Notes

**Figure 62.**
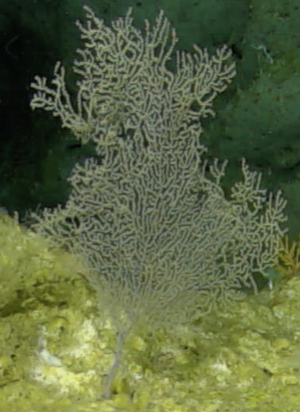
*Trimuricea* sp. indet. Aldabra N1, 120 m.

Colonies < 50 cm in height, fan-shaped and uniplanar. Dense, fine branches displaying a high degree of anastomoses, giving the colony a mesh-like appearance. Colour pale yellow-green to grey (Fig. 62).

#### Primnoidae

Milne Edwards, 1857

92C8A926-E942-5F5F-86B6-2165CC07C516

#### Primnoa

Lamouroux, 1812

92EE01C6-7ADF-5204-9F1B-65B1B26FC86D

#### Primnoa sp. indet.

8449181A-8E3C-5431-8D4D-ED95BD67D7B7

##### Materials

- **Type status:**Other material. **Taxon:** scientificName: *Primnoa*; kingdom: Animalia; phylum: Cnidaria; class: Anthozoa; order: Alcyonacea; family: Primnoidae; genus: Primnoa; scientificNameAuthorship: Lamouroux, 1812; **Location:** waterBody: Indian Ocean; country: Seychelles; locality: Aldabra W1, Alphonse N1; minimumDepthInMeters: 132 m; maximumDepthInMeters: 250 m; locationRemarks: First Descent: Seychelles Expedition; **Identification:** identifiedBy: Nico Fassbender, Kaveh Samimi-Namin, Paris Stefanoudis; dateIdentified: 2019, 2020; identificationRemarks: identified only from imagery; **Event:** samplingProtocol: Submersible OR Remotely Operated Vehicle OR SCUBA; **Record Level:** basisOfRecord: Human observation

##### Notes

Colonies typically ~ 20 cm in height, fan-shaped, densely-branched, with fine branches and a strong tree-like appearance. The colouration of the branches is a light orange that tends to have a reddish tint (Fig. 63).

#### Narella

Gray, 1870

023B76B6-E687-5C69-8338-C7618A54F7D4

#### Narella sp. indet.

DD44009F-8293-5817-941C-FBBDB3519CAB

##### Materials

- **Type status:**Other material. **Taxon:** scientificName: *Narella*; kingdom: Animalia; phylum: Cnidaria; class: Anthozoa; order: Alcyonacea; family: Primnoidae; genus: Narella; scientificNameAuthorship: Gray, 1870; **Location:** waterBody: Indian Ocean; country: Seychelles; locality: Aldabra N1, D'Arros N1; minimumDepthInMeters: 190 m; maximumDepthInMeters: 351 m; locationRemarks: First Descent: Seychelles Expedition; **Identification:** identifiedBy: Nico Fassbender, Kaveh Samimi-Namin, Paris Stefanoudis; dateIdentified: 2019, 2020; identificationRemarks: identified only from imagery; **Event:** samplingProtocol: Submersible OR Remotely Operated Vehicle OR SCUBA; **Record Level:** basisOfRecord: Human observation

##### Notes

Colonies up to 40 cm in height, sparsely branched, with thick branches starting from the base of the colony; large polyp calyces give branches a serrated appearance. The colouration of the colony is light pink (Fig. 64).

#### Subergorgiidae

Gray, 1859

C2C2943A-F2D0-5CBB-991F-89ADE596B601

#### Annella

Gray, 1858

13B7BB47-9C81-5D82-AF46-12061B36B126

#### Annella sp. indet.

55F11046-1577-5557-B7C6-6FD9A570297D

##### Materials

- **Type status:**Other material. **Taxon:** scientificName: *Annella*; kingdom: Animalia; phylum: Cnidaria; class: Anthozoa; order: Alcyonacea; family: Subergorgiidae; genus: Annella; scientificNameAuthorship: Gray, 1858; **Location:** waterBody: Indian Ocean; country: Seychelles; locality: Aldabra N1, Aldabra W1, Alphonse N1, Astove W1, D'Arros N1, Desroches S1, Poivre E1; minimumDepthInMeters: 21.7 m; maximumDepthInMeters: 148.1 m; locationRemarks: First Descent: Seychelles Expedition; **Identification:** identifiedBy: Nico Fassbender, Kaveh Samimi-Namin, Paris Stefanoudis; dateIdentified: 2019, 2020; identificationRemarks: identified only from imagery; **Event:** samplingProtocol: Submersible OR Remotely Operated Vehicle OR SCUBA; **Record Level:** basisOfRecord: Human observation

##### Notes

Colonies are fan-shaped and uniplanar. Branches display a high degree of anastomoses, forming net-like fans. Stalks are always attached to hard substrates. Colouration ranges from red to orange and yellow. Some colonies are larger than 2 m across. Often with crinoids commensals. The three known species of *Annella* are distinguished by the shape of the mesh (elongate or polygonal) (Fig. 65).

#### Tubiporidae

Ehrenberg, 1828

8EA22381-080E-5C15-AA5A-8C18F8DFECFC

#### Tubipora

Linnaeus, 1758

6B1ED38E-9641-5B98-B54C-26B761470D3D

#### Tubipora sp. indet.

04517EE0-700C-5AFE-A509-5834663F191D

##### Materials

- **Type status:**Other material. **Taxon:** scientificName: *Tubipora*; kingdom: Animalia; phylum: Cnidaria; class: Anthozoa; order: Alcyonacea; family: Tubiporidae; genus: Tubipora; scientificNameAuthorship: Linnaeus, 1758; **Location:** waterBody: Indian Ocean; country: Seychelles; locality: Alphonse N1, Desroches S1; minimumDepthInMeters: 10 m; maximumDepthInMeters: 10 m; locationRemarks: First Descent: Seychelles Expedition; **Identification:** identifiedBy: Nico Fassbender, Kaveh Samimi-Namin, Paris Stefanoudis; dateIdentified: 2019, 2020; identificationRemarks: identified only from imagery; **Event:** samplingProtocol: Submersible OR Remotely Operated Vehicle OR SCUBA; **Record Level:** basisOfRecord: Human observation

##### Notes

Colonies are thickly encrusted to massive and hemispherical, made up of upright, connected, parallel tubes that house a single polyp. Colonies can form step-like morphologies, growing on multilevel, horizontal platforms. Polyps are typically extended and visible. The skeleton of live individuals is always covered by polyps. The colouration of the skeleton is bright to dark red, with tentacles coloured in variations of pale, cream, green and white. Maximum recorded size: 55 cm across (Fig. 66).

#### Xeniidae

Ehrenberg, 1828

1F0E859B-FA16-56C9-A89C-A398086E9CA2

#### Xenia

Lamarck, 1816

36FA4912-4D9F-52D4-8FAB-85F11F4BC144

#### Xenia sp. indet.

655B1010-2ED3-5274-B1EA-F573FD6B40CA

##### Materials

- **Type status:**Other material. **Taxon:** scientificName: *Xenia*; kingdom: Animalia; phylum: Cnidaria; class: Anthozoa; order: Alcyonacea; family: Xeniidae; genus: Xenia; scientificNameAuthorship: Lamarck, 1816; **Location:** waterBody: Indian Ocean; country: Seychelles; locality: Aldabra N1, Astove W1, Poivre E1; minimumDepthInMeters: 30 m; maximumDepthInMeters: 30 m; locationRemarks: First Descent: Seychelles Expedition; **Identification:** identifiedBy: Nico Fassbender, Kaveh Samimi-Namin, Paris Stefanoudis; dateIdentified: 2019, 2020; identificationRemarks: identified only from imagery; **Event:** samplingProtocol: Submersible OR Remotely Operated Vehicle OR SCUBA; **Record Level:** basisOfRecord: Human observation

##### Notes

**Figure 67.**
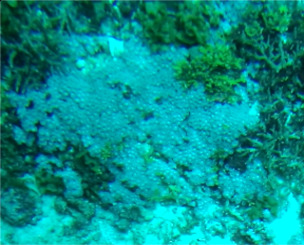
*Xenia* sp. indet. Poivre E1, 30 m.

Colonies up to 30 cm, cylindrical with dome-shaped summits. Can sometimes show branching (not observed here). Polyps found exclusively on the upper colony surface. Polyps have varying contractility, but are never fully retracted. The colouration of stalks is often similar to the polyps' colouration, ranging from white, yellow, cream, brown to dark brown (Fig. 67).

#### "ord. Alcyonacea" fam. indet. sp. 1

B61D5F11-BC19-5CF1-96F1-19F3253AC6AA

##### Materials

- **Type status:**Other material. **Taxon:** scientificName: Alcyonacea sp. 1; kingdom: Animalia; phylum: Cnidaria; class: Anthozoa; order: Alcyonacea; scientificNameAuthorship: Gray, 1859; **Location:** waterBody: Indian Ocean; country: Seychelles; locality: D'Arros N1, Desroches S1; minimumDepthInMeters: 114.5 m; maximumDepthInMeters: 120.7 m; locationRemarks: First Descent: Seychelles Expedition; **Identification:** identifiedBy: Nico Fassbender, Kaveh Samimi-Namin, Paris Stefanoudis; dateIdentified: 2019, 2020; identificationRemarks: identified only from imagery; **Event:** samplingProtocol: Submersible OR Remotely Operated Vehicle OR SCUBA; **Record Level:** basisOfRecord: Human observation

##### Notes

Colonies up to 20 cm in height, flat and fan-shaped, displaying dense, dichotomous branching. Polyps are numerous, giving the colony a fuzzy appearance. Light blue colouration with dark blue polyps (Fig. 68).

#### "ord. Alcyonacea" fam. indet. sp. 2

7BB0D0AD-CD2D-5696-A6DC-FDBA41FA39EE

##### Materials

- **Type status:**Other material. **Taxon:** scientificName: Alcyonacea sp. 2; kingdom: Animalia; phylum: Cnidaria; class: Anthozoa; order: Alcyonacea; scientificNameAuthorship: Gray, 1859; **Location:** waterBody: Indian Ocean; country: Seychelles; locality: D'Arros N1, Desroches S1; minimumDepthInMeters: 66.3 m; maximumDepthInMeters: 123.4 m; locationRemarks: First Descent: Seychelles Expedition; **Identification:** identifiedBy: Nico Fassbender, Kaveh Samimi-Namin, Paris Stefanoudis; dateIdentified: 2019, 2020; identificationRemarks: identified only from imagery; **Event:** samplingProtocol: Submersible OR Remotely Operated Vehicle OR SCUBA; **Record Level:** basisOfRecord: Human observation

##### Notes

**Figure 69.**
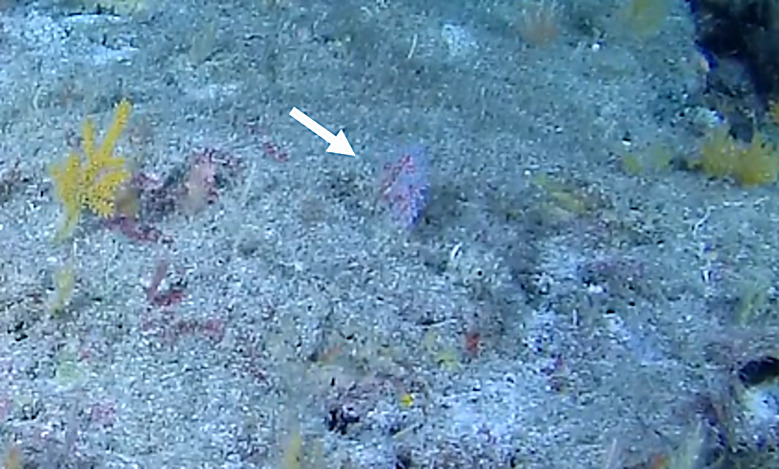
Alcyonacea fam. indet. sp. 2. D'Arros N1, 120 m.

Colonies typically ~15–20 cm in height, fan-shaped and heavily branched. Colour deep purple with lighter coloured polyps (Fig. 69).

#### "ord. Alcyonacea" fam. indet. sp. 3

6485F1CD-524D-5CCB-92CB-2AEBB3AEB5E6

##### Materials

- **Type status:**Other material. **Taxon:** scientificName: Alcyonacea sp. 3; kingdom: Animalia; phylum: Cnidaria; class: Anthozoa; order: Alcyonacea; scientificNameAuthorship: Gray, 1859; **Location:** waterBody: Indian Ocean; country: Seychelles; locality: D'Arros N1, Desroches S1; minimumDepthInMeters: 61.8 m; maximumDepthInMeters: 123.4 m; locationRemarks: First Descent: Seychelles Expedition; **Identification:** identifiedBy: Nico Fassbender, Kaveh Samimi-Namin, Paris Stefanoudis; dateIdentified: 2019, 2020; identificationRemarks: identified only from imagery; **Event:** samplingProtocol: Submersible OR Remotely Operated Vehicle OR SCUBA; **Record Level:** basisOfRecord: Human observation

##### Notes

Branching colonies up to 20 cm in height. Branching starts from the base of the stalk. Colour in shades of red to brown and orange (Fig. 70).

#### "ord. Alcyonacea" fam. indet. sp. 4

A8684F4E-1A70-5E7E-A7FC-43C6C82CA5C8

##### Materials

- **Type status:**Other material. **Taxon:** scientificName: Alcyonacea sp. 4; kingdom: Animalia; phylum: Cnidaria; class: Anthozoa; order: Alcyonacea; scientificNameAuthorship: Lamouroux, 1812; **Location:** waterBody: Indian Ocean; country: Seychelles; locality: Desroches S1; minimumDepthInMeters: 114.5 m; maximumDepthInMeters: 122.6 m; locationRemarks: First Descent: Seychelles Expedition; **Identification:** identifiedBy: Nico Fassbender, Kaveh Samimi-Namin, Paris Stefanoudis; dateIdentified: 2019, 2020; identificationRemarks: identified only from imagery; **Event:** samplingProtocol: Submersible OR Remotely Operated Vehicle OR SCUBA; **Record Level:** basisOfRecord: Human observation

##### Notes

**Figure 71.**
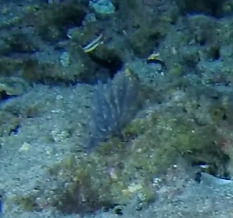
Alcyonacea fam. indet. sp. 4. Desroches S1, 120 m.

Colonies up to 30 cm in height, fan-shaped, growing uniplanar. Dichotomously branched. Dark grey to black colour (Fig. 71).

#### "ord. Alcyonacea" fam. indet. sp. 5

8828C640-2B4A-595A-981F-D8BC99924F30

##### Materials

- **Type status:**Other material. **Taxon:** scientificName: Alcyonacea sp. 5; kingdom: Animalia; phylum: Cnidaria; class: Anthozoa; order: Alcyonacea; scientificNameAuthorship: Lamouroux, 1812; **Location:** waterBody: Indian Ocean; country: Seychelles; locality: Astove W1; minimumDepthInMeters: 49.1 m; maximumDepthInMeters: 62.3 m; locationRemarks: First Descent: Seychelles Expedition; **Identification:** identifiedBy: Nico Fassbender, Kaveh Samimi-Namin, Paris Stefanoudis; dateIdentified: 2019, 2020; identificationRemarks: identified only from imagery; **Event:** samplingProtocol: Submersible OR Remotely Operated Vehicle OR SCUBA; **Record Level:** basisOfRecord: Human observation

##### Notes

**Figure 72.**
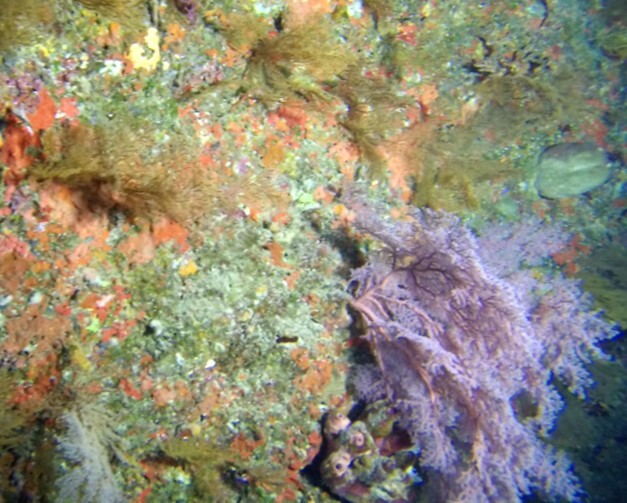
Alcyonacea fam. indet. sp. 5. Astove W1, 60 m.

Colonies up to 50 cm in height, fan-shaped, growing in multiple planes. Conspicuous central stem with many side branches. Branches appear fuzzy due to conspicuous polyps. Colour pink to fuchsia with polyps covered similar to the main colour, yet slightly lighter (Fig. 72).

#### Helioporacea

Bock, 1938

5FF7CB79-8189-5490-8524-7A036D474B57

#### Helioporidae

Moseley, 1876

88010A35-9004-5287-A2BF-1B44B32178A2

#### Heliopora

de Blainville, 1830

8396825E-AB13-5B2E-BADF-FE66D0CBA117

#### Heliopora sp. indet.

6BFB140E-BB30-5432-8979-A8A2E79267FB

##### Materials

- **Type status:**Other material. **Taxon:** scientificName: *Heliopora*; kingdom: Animalia; phylum: Cnidaria; class: Anthozoa; order: Helioporacea; family: Helioporidae; genus: Heliopora; scientificNameAuthorship: de Blainville, 1830; **Location:** waterBody: Indian Ocean; country: Seychelles; locality: Aldabra N1, Aldabra W1, Alphonse N1, Astove W1, Poivre E1; minimumDepthInMeters: 8.8 m; maximumDepthInMeters: 25.9 m; locationRemarks: First Descent: Seychelles Expedition; **Identification:** identifiedBy: Nico Fassbender, Kaveh Samimi-Namin, Paris Stefanoudis; dateIdentified: 2019, 2020; identificationRemarks: identified only from imagery; **Event:** samplingProtocol: Submersible OR Remotely Operated Vehicle OR SCUBA; **Record Level:** basisOfRecord: Human observation

##### Notes

Colonies are columnar, plating or branching. Maximum recorded size: 60 cm across. As the only octocoral genus with a massive aragonite skeleton, it is often mistaken for a scleractinian coral. However, its colonies have a unique blue to green colouration with large pore-like polyps of white colour that are often visible, both in-situ and on video footage (Fig. 73).

### Scleractinia

#### Acroporidae

Verrill, 1901

023422D1-4CC9-5D35-94FD-2B58B74C1032

#### Acropora

Oken, 1805

D06402E6-C591-5DB5-ADD9-6B0302AC4B8F

#### Acropora sp. indet.

D0E0B4AF-C995-57E0-8F9A-3010D52F696A

##### Materials

- **Type status:**Other material. **Taxon:** scientificName: *Acropora*; kingdom: Animalia; phylum: Cnidaria; class: Anthozoa; order: Scleractinia; family: Acroporidae; genus: Acropora; scientificNameAuthorship: Oken, 185; **Location:** waterBody: Indian Ocean; country: Seychelles; locality: Aldabra N1, Aldabra W1, Alphonse N1, Astove W1, Desroches S1, Poivre E1; minimumDepthInMeters: 8.8 m; maximumDepthInMeters: 36.6 m; locationRemarks: First Descent: Seychelles Expedition; **Identification:** identifiedBy: Gilberte Gendron, Nico Fassbender, Paris Stefanoudis, Rowana Walton; dateIdentified: 2019, 2020; identificationRemarks: identified only from imagery; **Event:** samplingProtocol: Submersible OR Remotely Operated Vehicle OR SCUBA; **Record Level:** basisOfRecord: Human observation

##### Notes

Wide range of morphologies; in the present survey, commonly digitate, branching or tabular. In this survey, colony size was typically < 40 cm across. Visually distinct corallites, 0.7 to 1.3 mm in diameter, cylindrical in appearance. A key feature of this taxon are its differentiated axial corallites located at the tips of branches that are often pale or white; this should not be confused with bleaching-induced colour changes. Commonly in shades of brown, although other colours can occur (Fig. 74).

#### Astreopora

Blainville, 1830

3772760A-456F-506E-826A-6FDD44FFF09E

#### Astreopora sp. indet.

79FA3E38-D155-5646-A04B-5216CD7FC8F4

##### Materials

- **Type status:**Other material. **Taxon:** scientificName: *Astreopora*; kingdom: Animalia; phylum: Cnidaria; class: Anthozoa; order: Scleractinia; family: Acroporidae; genus: Astreopora; scientificNameAuthorship: Blainville, 1830; **Location:** waterBody: Indian Ocean; country: Seychelles; locality: Aldabra N1, Alphonse N1, Astove W1, Desroches S1, Poivre E1; minimumDepthInMeters: 9.5 m; maximumDepthInMeters: 36.6 m; locationRemarks: First Descent: Seychelles Expedition; **Identification:** identifiedBy: Gilberte Gendron, Nico Fassbender, Paris Stefanoudis, Rowana Walton; dateIdentified: 2019, 2020; identificationRemarks: identified only from imagery; **Event:** samplingProtocol: Submersible OR Remotely Operated Vehicle OR SCUBA; **Record Level:** basisOfRecord: Human observation

##### Notes

Colonies are massive, plating or encrusting. Maximum recorded size: 80 cm across. Polyps are conspicuous with corallites between 1.6 to 2.2 mm (typically distinguishable on video footage); resembling jet engines, sometimes of irregular sizes, always tightly packed. Coenosteum is typically spinous or flaky, giving colonies a granular appearance. Colourations range from pale-brown tones to dark orangy-brown. Can be confused with *Turbinaria*, but the latter has a smooth coenosteum with no elaborations, which gives it a smoother appearance (Fig. 75).

#### Isopora

Studer, 1879

DF64FCFA-07EF-5E98-BC6A-D8A5ADA9332D

#### Isopora sp. indet.

EA98C7D0-AC7D-5A73-80FC-2BFF688D76A8

##### Materials

- **Type status:**Other material. **Taxon:** scientificName: *Isopora*; kingdom: Animalia; phylum: Cnidaria; class: Anthozoa; order: Scleractinia; family: Acroporidae; genus: Isopora; scientificNameAuthorship: Studer, 1879; **Location:** waterBody: Indian Ocean; country: Seychelles; locality: Aldabra W1, Alphonse N1, Astove W1; minimumDepthInMeters: 8.8 m; maximumDepthInMeters: 15 m; locationRemarks: First Descent: Seychelles Expedition; **Identification:** identifiedBy: Gilberte Gendron, Nico Fassbender, Paris Stefanoudis, Rowana Walton; dateIdentified: 2019, 2020; identificationRemarks: identified only from imagery; **Event:** samplingProtocol: Submersible OR Remotely Operated Vehicle OR SCUBA; **Record Level:** basisOfRecord: Human observation

##### Notes

Colonies are sub-massive or thickly encrusted often with thick club-like branches or robust cylindrical branches. Maximum recorded size: 1 m across. Corallites up to 4.0 mm in diameter. Colours range from cream, pale brown to green. Can be confused with *Montipora* or *Acropora* spp., with *Montipora* having much smaller corallites. *Acropora* forms thinly encrusting colonies without the robust columns that *Isopora* forms (Fig. 76).

#### Montipora

Blainville, 1830

09A5E440-2BDB-5EC1-8BE8-721BD32D6260

#### Montipora sp. indet.

37CC8404-F86A-523B-A0A9-E905C3AA440C

##### Materials

- **Type status:**Other material. **Taxon:** scientificName: *Montipora*; kingdom: Animalia; phylum: Cnidaria; class: Anthozoa; order: Scleractinia; family: Acroporidae; genus: Montipora; scientificNameAuthorship: Blainville, 1830; **Location:** waterBody: Indian Ocean; country: Seychelles; locality: Aldabra N1, Aldabra W1, Alphonse N1, Astove W1, D'Arros N1, Desroches S1, Poivre E1; minimumDepthInMeters: 8.8 m; maximumDepthInMeters: 63.1 m; locationRemarks: First Descent: Seychelles Expedition; **Identification:** identifiedBy: Gilberte Gendron, Nico Fassbender, Paris Stefanoudis, Rowana Walton; dateIdentified: 2019, 2020; identificationRemarks: identified only from imagery; **Event:** samplingProtocol: Submersible OR Remotely Operated Vehicle OR SCUBA; **Record Level:** basisOfRecord: Human observation

##### Notes

Colonies are thickly encrusting, sub-massive, plating. Maximum recorded size: 1.2 m across. Corallites are extremely small (0.25 to 1.0 mm) and thus not visible on video footage. Very rough, grainy texture, often with several bumps on colony surface. Colours ranging from beige to dark shades of brown, some species with variable additional pigmentation like purple, red and violet (Fig. 77).

#### Agariciidae

Gray, 1847

D6BDD08D-6FCF-5675-A1CD-F5E3F52AD610

#### Gardineroseris

Scheer & Pillai, 1974

414A4E8A-8C66-517C-ADF9-968CE6B780EC

#### Gardineroseris sp. indet.

B2B0C787-B131-568F-B574-2BE04B0A75A4

##### Materials

- **Type status:**Other material. **Taxon:** scientificName: *Gardineroseris*; kingdom: Animalia; phylum: Cnidaria; class: Anthozoa; order: Scleractinia; family: Agariciidae; genus: Gardineroseris; scientificNameAuthorship: Scheer & Pillai, 1974; **Location:** waterBody: Indian Ocean; country: Seychelles; locality: Aldabra N1, Aldabra W1, Alphonse N1, Astove W1, Desroches S1; minimumDepthInMeters: 8.8 m; maximumDepthInMeters: 15 m; locationRemarks: First Descent: Seychelles Expedition; **Identification:** identifiedBy: Gilberte Gendron, Nico Fassbender, Paris Stefanoudis, Rowana Walton; dateIdentified: 2019, 2020; identificationRemarks: identified only from imagerySubmersible OR Remotely Operated Vehicle OR SCUBA; **Event:** samplingProtocol: Submersible OR Remotely Operated Vehicle OR SCUBA; **Record Level:** basisOfRecord: Human observation

##### Notes

**Figure 78.**
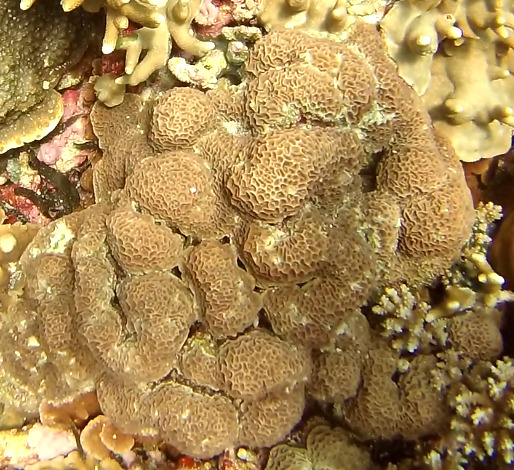
*Gardineroseris* sp. indet. Astove W1, 10 m.

Colonies are massive or encrusting with laminar margins. Maximum recorded size: 35 cm across. Corallites are immersed with distinct, steeply sloping acutely ridged walls whose ridges can appear pale, giving the colony a honeycomb-like appearance. Corallite size 3.0 mm in diameter. Colours range from yellowish to shades of light and dark brown. *Goniastrea* appears similar, but has highly visible septa that give the colony walls a serrated appearance, with colonies coloured more pale brown-whitish, rather than the uniform brown of *Gardineroseris* (Fig. 78).

#### Leptoseris

Milne Edwards & Haime, 1849

4D909046-84B4-58C8-9B4F-324B1252D380

#### Leptoseris sp. indet.

09A870BC-6CAE-5D9B-93CD-41726D1862FA

##### Materials

- **Type status:**Other material. **Taxon:** scientificName: *Leptoseris*; kingdom: Animalia; phylum: Cnidaria; class: Anthozoa; order: Scleractinia; family: Agariciidae; genus: Leptoseris; scientificNameAuthorship: Milne Edwards & Haine, 1849; **Location:** waterBody: Indian Ocean; country: Seychelles; locality: Aldabra N1, Aldabra W1, Alphonse N1, Astove W1, D'Arros N1, Desroches S1, Poivre E1; minimumDepthInMeters: 8.8 m; maximumDepthInMeters: 71.5 m; locationRemarks: First Descent: Seychelles Expedition; **Identification:** identifiedBy: Gilberte Gendron, Nico Fassbender, Paris Stefanoudis, Rowana Walton; dateIdentified: 2019, 2020; identificationRemarks: identified only from imagery; **Event:** samplingProtocol: Submersible OR Remotely Operated Vehicle OR SCUBA; **Record Level:** basisOfRecord: Human observation

##### Notes

Colonies typically small (~ 20 cm in the longest dimension), although some were up to 90 cm, encrusting or plating, unifacial and contorted. Polyps usually without distinct walls and larger than 5 mm in diameter. Growth form tends to change with depth: deeper colonies found in mesophotic depths are often laminar with a white edge. Widely spaced corallites that may be inclined to the margin, that can cluster within pockets (raised rounded walls). Appearance is wavy, granulated or waxy; deeper plating forms with small bumps on the colony surface. Colour ranges from pale to lighter shades of yellow-brown. *Pavona* looks similar, but is mostly bifacial with a rougher appearance; can be confused with plating colonies of *Pachyseris*, but the latter has clearly defined parallel ridges, lacks bumps at the surface and tends to form metre-long colonies. However, small plating colonies at depth might be difficult to separate if seen from a distance. For *Pachyseris* colonies, the corallite walls always run to the edge of the colony; however, in *Leptoseris*, these walls do not always run to the edge and are not uniform (Fig. 79).

#### Pavona

Lamarck, 1801

B010A0ED-9A93-55FD-8B08-D8DC30735856

#### Pavona sp. indet.

4925B6C9-07C8-59DB-8669-7DE5ADCA266B

##### Materials

- **Type status:**Other material. **Taxon:** scientificName: *Pavona*; kingdom: Animalia; phylum: Cnidaria; class: Anthozoa; order: Scleractinia; family: Agariciidae; genus: Pavona; scientificNameAuthorship: Lamarck, 1801; **Location:** waterBody: Indian Ocean; country: Seychelles; locality: Aldabra N1, Aldabra W1, Alphonse N1, Astove W1, Desroches S1, Poivre E1; minimumDepthInMeters: 8.8 m; maximumDepthInMeters: 33.1 m; locationRemarks: First Descent: Seychelles Expedition; **Identification:** identifiedBy: Gilberte Gendron, Nico Fassbender, Paris Stefanoudis, Rowana Walton; dateIdentified: 2019, 2020; identificationRemarks: identified only from imagery; **Event:** samplingProtocol: Submersible OR Remotely Operated Vehicle OR SCUBA; **Record Level:** basisOfRecord: Human observation

##### Notes

Colonies typically < 40 cm in the longest dimension, massive, columnar, laminar or encrusting, sometimes contorted. Laminar colonies are bifacial. Corallites between 0.5 to 3.0 mm in size, walls poorly developed or absent, centres in small shallow depressions surrounded by acute ridges. Colours range from beige to darker shades of brown. Might be confused with *Leptoseris*, but the latter has less acute ridges between corallites and colonies are unifacial. Massive and columnar colonies were not observed here (Fig. 80).

#### Dendrophyllidae

Gray, 1847

16F3CF36-FC00-5819-87CD-9F81E4D0BB34

#### Tubastraea

Lesson, 1830

E00B1390-916E-5B18-8299-8B8DCF797C42

#### Tubastraea sp. indet.

9BDCE09F-21F1-5707-BF00-69112BEED14F

##### Materials

- **Type status:**Other material. **Taxon:** scientificName: *Tubastraea*; kingdom: Animalia; phylum: Cnidaria; class: Anthozoa; order: Scleractinia; family: Dendrophyllidae; genus: Tubastraea; scientificNameAuthorship: Lesson, 1830; **Location:** waterBody: Indian Ocean; country: Seychelles; locality: Aldabra N1, Alphonse N1, Astove W1, D'Arros N1, Desroches S1, Poivre E1; minimumDepthInMeters: 9.7 m; maximumDepthInMeters: 67.9 m; locationRemarks: First Descent: Seychelles Expedition; **Identification:** identifiedBy: Gilberte Gendron, Nico Fassbender, Paris Stefanoudis, Rowana Walton; dateIdentified: Gilberte Gendron, Nico Fassbender, Paris Stefanoudis, Rowana Walton; identificationRemarks: identified only from imagery; **Event:** samplingProtocol: Submersible OR Remotely Operated Vehicle OR SCUBA; **Record Level:** basisOfRecord: Human observation

##### Notes

Small (typically < 10 cm) branching colonies. Branches ending in tubular corallites. Corallites have high, thin walls with well-defined septa. Colouration normally dark green or black, in our survey orange or grey (Fig. 81).

#### Tubastraea micranthus

(Ehrenberg, 1834)

52F14DB2-4CD7-5481-9F04-F09431F8FAC3

##### Materials

- **Type status:**Other material. **Taxon:** scientificName: *Tubastraeamicranthus*; kingdom: Animalia; phylum: Cnidaria; class: Anthozoa; order: Scleractinia; family: Dendrophyllidae; genus: Tubastraea; scientificNameAuthorship: Ehrenberg, 1834; **Location:** waterBody: Indian Ocean; country: Seychelles; locality: Aldabra N1, Aldabra W1, Alphonse N1; minimumDepthInMeters: 21 m; maximumDepthInMeters: 53 m; locationRemarks: First Descent: Seychelles Expedition; **Identification:** identifiedBy: Gilberte Gendron, Nico Fassbender, Paris Stefanoudis, Rowana Walton; dateIdentified: 2019, 2020; identificationRemarks: identified only from imagery; **Event:** samplingProtocol: Submersible OR Remotely Operated Vehicle OR SCUBA; **Record Level:** basisOfRecord: Human observation

##### Notes

**Figure 82.**
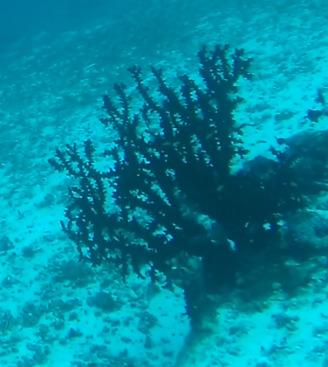
*Tubastraeamicranthus*. Alphonse N1, 60 m.

Colonies are tall with thick, erect branches. Maximum recorded size: 30 cm across. Corallites sparse, tubular and are clearly discernible both in-situ and on video footage. Colouration is dark green or black. *T.micranthus* is the only species of *Tubastraea* observed that could be continuously identified to species level (Fig. 82).

#### Turbinaria

Oken, 1815

DF502B15-BBBD-561A-BB35-09DC5A1A5647

#### Turbinaria sp. indet.

924A5EA3-8021-5FA0-9900-4E69E7611ABF

##### Materials

- **Type status:**Other material. **Taxon:** scientificName: *Turbinaria*; kingdom: Animalia; phylum: Cnidaria; class: Anthozoa; order: Scleractinia; family: Dendrophyllidae; genus: Turbinaria; scientificNameAuthorship: Oken, 1815; **Location:** waterBody: Indian Ocean; country: Seychelles; locality: Aldabra N1, Aldabra W1, Alphonse N1, Astove W1, Desroches S1, Poivre E1; minimumDepthInMeters: 8.8 m; maximumDepthInMeters: 35 m; locationRemarks: First Descent: Seychelles Expedition; **Identification:** identifiedBy: Gilberte Gendron, Nico Fassbender, Paris Stefanoudis, Rowana Walton; dateIdentified: 2019, 2020; identificationRemarks: identified only from imagery; **Event:** samplingProtocol: Submersible OR Remotely Operated Vehicle OR SCUBA; **Record Level:** basisOfRecord: Human observation

##### Notes

Colonies are laminar, columnar, dome-shaped or foliaceous. Maximum recorded size: 1.3 m across. Frequently contorted; laminar and foliaceous growth forms often with paler margins. Corallites usually only on one surface, round and well-spaced from each other; often form tubular raised mounts giving the coral a bumpy texture. Corallite size 1.5 to 4.0 mm. Smooth coenosteum. Colouration ranging from beige to shades of brown and green. *Astreopora* appears similar; however, has a granulated coenosteum (Fig. 83).

#### Euphylliidae

Milne Edwards & Haime, 1857

FD935AC3-B4F4-50CA-8F16-4002976BABE2

#### Galaxea

Oken, 1815

C9885B76-449F-57E6-AD1E-D51DF135FE14

#### Galaxea sp. indet.

937A35F8-ED54-53DD-9D69-0F03C84ED0BB

##### Materials

- **Type status:**Other material. **Taxon:** scientificName: *Galaxea*; kingdom: Animalia; phylum: Cnidaria; class: Anthozoa; order: Scleractinia; family: Euphylliidae; genus: Galaxea; scientificNameAuthorship: Oken, 1815; **Location:** waterBody: Indian Ocean; country: Seychelles; locality: Aldabra N1, Alphonse N1, Astove W1, Desroches S1; minimumDepthInMeters: 9.6 m; maximumDepthInMeters: 36.6 m; locationRemarks: First Descent: Seychelles Expedition; **Identification:** identifiedBy: Gilberte Gendron, Nico Fassbender, Paris Stefanoudis, Rowana Walton; dateIdentified: 2019, 2020; identificationRemarks: identified only from imagery; **Event:** samplingProtocol: Submersible OR Remotely Operated Vehicle OR SCUBA; **Record Level:** basisOfRecord: Human observation

##### Notes

Massive or encrusting colonies, predominantly cushion-shaped or irregularly following the substrate. Maximum recorded size: 20 cm across. Corallites are between 3.5 to 6.0 mm, plocoid, cylindrical with relatively large, visible gaps. Septa are large and form tall, sharp points that are visible underwater. Tentacles can be extended during the day. Colours can be green to brown with yellow variants observed at Desroches (Fig. 84).

#### Fungiidae

Dana, 1846

D90C2D1D-473E-5C4A-9363-1EEF426FB623

#### "fam. Fungiidae" gen. indet. sp. 1

7C27E5A4-D389-5D58-A93C-D569F0F927D2

##### Materials

- **Type status:**Other material. **Taxon:** scientificName: Fungiidae*sp. 1*; kingdom: Animalia; phylum: Cnidaria; class: Anthozoa; order: Scleractinia; family: Fungiidae; scientificNameAuthorship: Verrill, 1864 / Eschscholtz, 1825; **Location:** waterBody: Indian Ocean; country: Seychelles; locality: Aldabra W1, Desroches S1, Poivre E1; minimumDepthInMeters: 8.8 m; maximumDepthInMeters: 36.6 m; locationRemarks: First Descent: Seychelles Expedition; **Identification:** identifiedBy: Gilberte Gendron, Nico Fassbender, Paris Stefanoudis, Rowana Walton; dateIdentified: 2019, 2020; identificationRemarks: identified only from imagery; **Event:** samplingProtocol: Submersible OR Remotely Operated Vehicle OR SCUBA; **Record Level:** basisOfRecord: Human observation

##### Notes

Solitary, free-living. Forms elongated discs with an axial furrow that may extend to reach the colony edge. Septa are alternating and are non-continuous from the axial furrow to the sides. The maximum recorded size in this survey was 30 cm (length), although specimens can grow >1 m and also appear y- or x-shaped. Belongs to either *Ctenactis* or *Herpolitha*; however, these two genera are not easy to distinguish from video footage alone, as that requires a close-up examination of individuals underwater. Corallite size 5.0 to 10.0 mm (Fig. 85).

#### "fam. Fungiidae" gen. indet. sp. 2

9BEE8556-8DEF-5736-A56A-84EDD780DFAF

##### Materials

- **Type status:**Other material. **Taxon:** scientificName: Fungiidae sp. 2; kingdom: Animalia; phylum: Cnidaria; class: Anthozoa; order: Scleractinia; family: Fungiidae; scientificNameAuthorship: Milne Edwards & Haime, 1849 / Lamarck, 1801; **Location:** waterBody: Indian Ocean; country: Seychelles; locality: Aldabra N1, Aldabra W1, Alphonse N1, Astove W1, D'Arros N1, Desroches S1, Poivre E1; minimumDepthInMeters: 8.8 m; maximumDepthInMeters: 36.6 m; locationRemarks: First Descent: Seychelles Expedition; **Identification:** identifiedBy: Gilberte Gendron, Nico Fassbender, Paris Stefanoudis, Rowana Walton; dateIdentified: 2019, 2020; identificationRemarks: identified only from imagery; **Event:** samplingProtocol: Submersible OR Remotely Operated Vehicle OR SCUBA; **Record Level:** basisOfRecord: Human observation

##### Notes

Solitary, free-living, except juveniles of *Fungia* that are firmly attached to substrates. Forms oval to round discs. Maximum recorded size: 19 cm across. Septa radiating out from the slit-like central mouth. Individuals belong to either Cycloseis or Fungia; however, these two genera cannot be consistently distinguished from video footage alone. *Fungia* tends to be more prominent on reef areas and its septa are serrated. Corallite size up to 300 mm. *Cycloseris* tends to be more prominent in non-reef environments and has smooth septa. Corallite size between 40.0 and 80.0 mm (Fig. 86).

#### Halomitra

Eschscholtz, 1825

81E3D41F-8405-5BE9-A409-3DAD936D1850

#### Halomitra sp. indet.

2CFAEB14-B425-5281-8F1C-CBC69E9BD05B

##### Materials

- **Type status:**Other material. **Taxon:** scientificName: Halomitra; kingdom: Animalia; phylum: Cnidaria; class: Anthozoa; order: Scleractinia; family: Fungiidae; genus: Halomitra; scientificNameAuthorship: Dana, 1846; **Location:** waterBody: Indian Ocean; country: Seychelles; locality: Aldabra W1, Desroches S1, Poivre E1; minimumDepthInMeters: 11.3 m; maximumDepthInMeters: 36.5 m; locationRemarks: First Descent: Seychelles Expedition; **Identification:** identifiedBy: Gilberte Gendron, Nico Fassbender, Paris Stefanoudis, Rowana Walton; dateIdentified: 2019, 2020; identificationRemarks: identified only from imagery; **Event:** samplingProtocol: Submersible OR Remotely Operated Vehicle OR SCUBA; **Record Level:** basisOfRecord: Human observation

##### Notes

**Figure 87.**
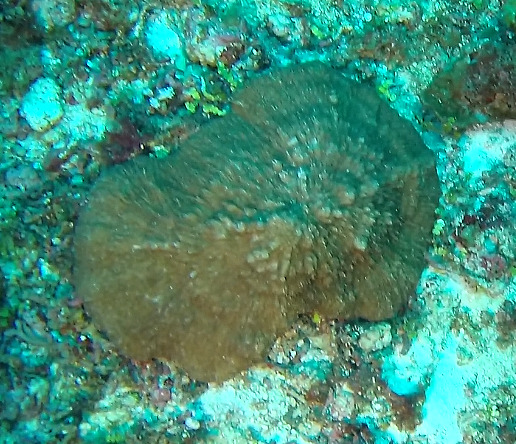
Poivre E1, 30 m.

Free-living, flat, domed or bell-shaped and oval growth forms are commonly observed. Maximum recorded size: 30 cm across. Corallites 6.0 mm. No axial furrow, but septo-costae radiate out from the centre towards the margin. Corallites are often white. Colouration pale brown with bright pink or purple margin (Fig. 87).

#### Leptastreidae

Rowlett, 2020

FC2FD6DD-1214-5BB6-BCC1-37FB881FBC17

#### Leptastrea

Milne Edwards & Haime, 1849

9ABF06E1-2BEF-51E1-8B88-2A31F7248A21

#### Leptastrea sp. indet.

51E84F45-96F3-59D0-83F6-1B8D2F81E002

##### Materials

- **Type status:**Other material. **Taxon:** scientificName: *Leptastrea*; kingdom: Animalia; phylum: Cnidaria; class: Anthozoa; order: Scleractinia; family: Leptastreidae ; genus: Leptastrea; scientificNameAuthorship: Milne Edwards & Haime, 1849; **Location:** waterBody: Indian Ocean; country: Seychelles; locality: Aldabra N1, Aldabra W1, Alphonse N1, Astove W1, Poivre E1; minimumDepthInMeters: 8.8 m; maximumDepthInMeters: 33 m; locationRemarks: First Descent: Seychelles Expedition; **Identification:** identifiedBy: Gilberte Gendron, Nico Fassbender, Paris Stefanoudis, Rowana Walton; dateIdentified: 2019, 2020; identificationRemarks: identified only from imagery; **Event:** samplingProtocol: Submersible OR Remotely Operated Vehicle OR SCUBA; **Record Level:** basisOfRecord: Human observation

##### Notes

Colonies can be massive, flat or dome-shaped or, as observed here, encrusting. Maximum recorded size: 50 cm across. Corallites raised unevenly from the coenosteum, giving the colonies a bumpy appearance. Corallites are 2.5 to 6.0 mm in diameter. Colouration shades of brown, but tends to have a white upper surface with darker corallites. *Goniastrea* looks similar, but tends to be of uniform colour (Fig. 88).

#### Lobophylliidae

Dai & Horng, 2009

30F9DCEB-855B-5791-ABB9-9F87DE4D2236

#### Echinophyllia

Klunzinger, 1879

CF5ED182-E590-55D3-A570-7FEF9DA51564

#### Echinophyllia sp. indet.

BEDA7068-AE6C-5DBB-BD66-892440EE5B63

##### Materials

- **Type status:**Other material. **Taxon:** scientificName: *Echinophyllia*; kingdom: Animalia; phylum: Cnidaria; class: Anthozoa; order: Scleractinia; family: Lobophylliidae; genus: Echinophyllia; scientificNameAuthorship: Klunzinger, 1879; **Location:** waterBody: Indian Ocean; country: Seychelles; locality: Aldabra N1, Astove W1, D'Arros N1, Desroches S1, Poivre E1; minimumDepthInMeters: 9.7 m; maximumDepthInMeters: 71.5 m; locationRemarks: First Descent: Seychelles Expedition; **Identification:** identifiedBy: Gilberte Gendron, Nico Fassbender, Paris Stefanoudis, Rowana Walton; dateIdentified: 2019, 2020; identificationRemarks: identified only from imagery; **Event:** samplingProtocol: Submersible OR Remotely Operated Vehicle OR SCUBA; **Record Level:** basisOfRecord: Human observation

##### Notes

Colonies are thickly encrusting or laminar. Maximum recorded size: 1 m across. Large, round or oval protuberant corallites that are visible underwater, 4.0 to 15.0 mm in diameter. Septa are numerous and form visible ridges running along the surface towards the edge of the colony; the colony edge appears serrated. Septa resemble dripping candle wax. Corallites are scattered and often separated by a gap of several mm. Colouration brown, commonly with pale or whitish scalloped edges. *Mycedium* looks similar, but can be distinguished by its larger corallites that are facing towards the edges of the colonies and are shaped like noses (Fig. 89).

#### Lobophyllia

Blainville, 1830

8A59FA27-BD07-55BA-ADA9-7CECF0FB7F4A

#### Lobophyllia sp. indet.

9DAE8EF0-D676-5034-80EA-606C9583EF74

##### Materials

- **Type status:**Other material. **Taxon:** scientificName: *Lobophyllia*; kingdom: Animalia; phylum: Cnidaria; class: Anthozoa; order: Scleractinia; family: Lobophylliidae; genus: Lobophyllia; scientificNameAuthorship: Blainville, 1830; **Location:** waterBody: Indian Ocean; country: Seychelles; locality: Aldabra N1, Aldabra W1, Alphonse N1, Astove W1, D'Arros N1, Desroches S1, Poivre E1; minimumDepthInMeters: 8.8 m; maximumDepthInMeters: 39.4 m; locationRemarks: First Descent: Seychelles Expedition; **Identification:** identifiedBy: Gilberte Gendron, Nico Fassbender, Paris Stefanoudis, Rowana Walton; dateIdentified: 2019, 2020; identificationRemarks: identified only from imagery; **Event:** samplingProtocol: Submersible OR Remotely Operated Vehicle OR SCUBA; **Record Level:** basisOfRecord: Human observation

##### Notes

Colonies are massive, sub-massive or flat-topped. Maximum recorded size: 50 cm across. Most colonies in this survey with corallites forming valleys of varying length, resembling ear-lobes or meandering ridges. Often with spiky appearance due to large skeletal teeth. Large corallites that range in size from 13.0 to 35.0 mm. Colours range from brown and greyish-green to bright orange and shades of purple. *Platygyra* looks similar, but has narrower valleys deepening towards the colony centres, whereas *Lobophyllia* has evenly deep ridges (Fig. 90).

#### Merulinidae

Verrill, 1865

229540E5-673E-5E80-9379-D3E8AD166D39

#### Dipsastraea

Blainville, 183

1A3FA7B7-1D84-5189-8917-23859BD55F24

#### Dipsastraea sp. indet.

353ADA33-3D1D-5BE5-BDB9-F77F8953D291

##### Materials

- **Type status:**Other material. **Taxon:** scientificName: *Dipsastraea*; kingdom: Animalia; phylum: Cnidaria; class: Anthozoa; order: Scleractinia; family: Merulinidae; genus: Dipsastraea; scientificNameAuthorship: Blainvile, 1830; **Location:** waterBody: Indian Ocean; country: Seychelles; locality: Aldabra N1, Aldabra W1, Alphonse N1, Astove W1, D'Arros N1, Desroches S1, Poivre E1; minimumDepthInMeters: 8.8 m; maximumDepthInMeters: 39.4 m; locationRemarks: First Descent: Seychelles Expedition; **Identification:** identifiedBy: Gilberte Gendron, Nico Fassbender, Paris Stefanoudis, Rowana Walton; dateIdentified: 2019, 2020; identificationRemarks: identified only from imagery; **Event:** samplingProtocol: Submersible OR Remotely Operated Vehicle OR SCUBA; **Record Level:** basisOfRecord: Human observation

##### Notes

Massive, sub-massive or encrusting colonies. Maximum recorded size: 50 cm across. Corallites are highly plocoid to almost-cerioid, roughly equal in size and do not share walls. Corallite size can range from 3.0 to 25.0 mm. Colouration shades of beige, brown or green. Sometimes confused with *Montastrea* whose corallites are squeezed into more irregular shapes. *Favites* colonies look similar, but their corallites share walls (Fig. 91).

#### Echinopora

Lamarck, 1816

A3D7BB88-E1C4-560E-BBC4-A8862DCC7BE1

#### Echinopora sp. indet.

E1B5004E-A50F-5F7B-945C-C1BBE3DCAFF7

##### Materials

- **Type status:**Other material. **Taxon:** scientificName: *Echinopora*; kingdom: Animalia; phylum: Cnidaria; class: Anthozoa; order: Scleractinia; family: Merulinidae; genus: Echinopora; scientificNameAuthorship: Lamarck, 1816; **Location:** waterBody: Indian Ocean; country: Seychelles; locality: Aldabra N1, Aldabra W1, Alphonse N1, Astove W1, Desroches S1, Poivre E1; minimumDepthInMeters: 8.8 m; maximumDepthInMeters: 52 m; locationRemarks: First Descent: Seychelles Expedition; **Identification:** identifiedBy: Gilberte Gendron, Nico Fassbender, Paris Stefanoudis, Rowana Walton; dateIdentified: 2019, 2020; identificationRemarks: identified only from imagery; **Event:** samplingProtocol: Submersible OR Remotely Operated Vehicle OR SCUBA; **Record Level:** basisOfRecord: Human observation

##### Notes

Colonies encrusting, laminar or branching. Maximum recorded size: 1 m across. Corallites are plocoid, uniformly shaped and elevated from the colony surface, 3.5 to 4.5 mm; they are often visible on video footage. The surface of the colony appears rough due to irregularly shaped septo-costae with spines. Colour shades of brown to grey with brightly coloured corallite centres (green) and white growing edges. *Echinophyllia* looks similar, but has larger corallites with spines that are arranged in distinct rows. *Astreopora* has smaller corallites (Fig. 92).

#### Favites

Link, 1807

52EEE4AC-9567-560B-B227-5DF83375BB0B

#### Favites sp. indet.

8B774A20-AD53-5946-9F1F-E90DD8F1AED5

##### Materials

- **Type status:**Other material. **Taxon:** scientificName: *Favites*; kingdom: Animalia; phylum: Cnidaria; class: Anthozoa; order: Scleractinia; family: Merulinidae; genus: Favites; scientificNameAuthorship: Link, 1807; **Location:** waterBody: Indian Ocean; country: Seychelles; locality: Aldabra N1, Aldabra W1, Alphonse N1, Astove W1, D'Arros N1, Desroches S1, Poivre E1; minimumDepthInMeters: 8.8 m; maximumDepthInMeters: 52 m; locationRemarks: First Descent: Seychelles Expedition; **Identification:** identifiedBy: Gilberte Gendron, Nico Fassbender, Paris Stefanoudis, Rowana Walton; dateIdentified: 2019, 2020; identificationRemarks: identified only from imagery; **Event:** samplingProtocol: Submersible OR Remotely Operated Vehicle OR SCUBA; **Record Level:** basisOfRecord: Human observation

##### Notes

Colonies massive, sub-massive or encrusting. Maximum recorded size: 60 cm across. Corallites are cerioid, with oblong or polygonal calyces of even size, between 3.0 to 25.0 mm across; they share walls that can be smooth or serrated-looking. Colours vary from brown to yellow and sometimes orange or green. *Dipsastraea* appears superficially similar, but corallites do not share walls (Fig. 93).

#### Goniastrea

Milne Edwards & Haime, 1848

B627CBBF-0C24-58CA-9D26-F3A25927BAEC

#### Goniastrea sp. indet.

609A44D0-A3FC-5F1C-BF99-7BBCE8E942CB

##### Materials

- **Type status:**Other material. **Taxon:** scientificName: *Goniastrea*; kingdom: Animalia; phylum: Cnidaria; class: Anthozoa; order: Scleractinia; family: Merulinidae; genus: Goniastrea; scientificNameAuthorship: Milne Edwards & Haime, 1848; **Location:** waterBody: Indian Ocean; country: Seychelles; locality: Aldabra W1, Alphonse N1, Astove W1, Desroches S1, Poivre E1; minimumDepthInMeters: 8.8 m; maximumDepthInMeters: 35.8 m; locationRemarks: First Descent: Seychelles Expedition; **Identification:** identifiedBy: Gilberte Gendron, Nico Fassbender, Paris Stefanoudis, Rowana Walton; dateIdentified: 2019, 2020; identificationRemarks: identified only from imagery; **Event:** samplingProtocol: Submersible OR Remotely Operated Vehicle OR SCUBA; **Record Level:** basisOfRecord: Human observation

##### Notes

Colonies massive, sub-massive, plate-like or encrusting. Maximum recorded size: 80 cm across. Corallites are cerioid, uniformly shaped and 3.5 to 14 mm in diameter. The septo-costae are neatly arranged around the corallite chalices, giving them a serrated appearance. Colour shades of brown. *Gardineroseris* looks similar, but has corallites with smooth walls. *Favites* superficially similar, but has less regular septa (Fig. 94).

#### Hydnophora

Fischer von Waldheim, 1807

1B951765-4920-5743-BC01-8BCD4884F207

#### Hydnophora sp. indet.

3A9C97B1-3B95-56BC-B970-DCB4D9EC9649

##### Materials

- **Type status:**Other material. **Taxon:** scientificName: *Hydnophora*; kingdom: Animalia; phylum: Cnidaria; class: Anthozoa; order: Scleractinia; family: Merulinidae; genus: Hydnophora; scientificNameAuthorship: Fischer von Waldheim, 1807; **Location:** waterBody: Indian Ocean; country: Seychelles; locality: Alphonse N1, Desroches S1; minimumDepthInMeters: 10 m; maximumDepthInMeters: 10 m; locationRemarks: First Descent: Seychelles Expedition; **Identification:** identifiedBy: Gilberte Gendron, Nico Fassbender, Paris Stefanoudis, Rowana Walton; dateIdentified: 2019, 2020; identificationRemarks: identified only from imagery; **Event:** samplingProtocol: Submersible OR Remotely Operated Vehicle OR SCUBA; **Record Level:** basisOfRecord: Human observation

##### Notes

**Figure 95.**
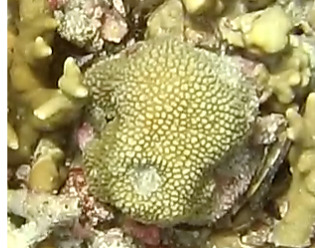
*Hydnophora* sp. indet. Massive colony. Alphonse N1, 10 m.

Colonies massive, encrusting or branching. Maximum recorded size: 30 cm across. The colony surface is covered in skeletal bumps (‘hydnophores’) that appear as lemon-squeezers or juicers, with small corallites, 3.0 to 4.0 mm in size, clustered in between. Hydnophores are often brighter than the corallites, with the latter being commonly brown to grey-green (Fig. 95).

#### Pectinia

Blainville, 1825

436DA054-61CD-5B91-9BE5-13D6BFE0A48C

#### Pectinia sp. indet.

71E0C49D-2983-5C7D-AEA5-D10AF69C4340

##### Materials

- **Type status:**Other material. **Taxon:** scientificName: *Pectinia*; kingdom: Animalia; phylum: Cnidaria; class: Anthozoa; order: Scleractinia; family: Merulinidae; genus: Pectinia; scientificNameAuthorship: Blainvile, 1825; **Location:** waterBody: Indian Ocean; country: Seychelles; locality: Aldabra W1, Astove W1, Desroches S1; minimumDepthInMeters: 11.3 m; maximumDepthInMeters: 15 m; locationRemarks: First Descent: Seychelles Expedition; **Identification:** identifiedBy: Gilberte Gendron, Nico Fassbender, Paris Stefanoudis, Rowana Walton; dateIdentified: 2019, 2020; identificationRemarks: identified only from imagery; **Event:** samplingProtocol: Submersible OR Remotely Operated Vehicle OR SCUBA; **Record Level:** basisOfRecord: Human observation

##### Notes

**Figure 96.**
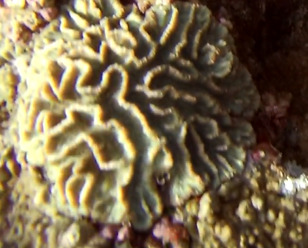
*Pectinia* sp. indet. Astove W1, 10 m.

Dome-shaped or laminar colonies, forming thick plates. Maximum recorded size: 30 cm across. Corallites 4.5 to 24.0 mm. With thin-walled, meandering valleys that increase in depth towards the centre of the colony. Some colonies branch towards the centre. Colour ranges from light brown to grey or green. *Platygyra* can appear similar from afar; however, has valleys of uniform depth. Its valleys tend to have a rougher texture due to its highly developed septa, often with a flattened top and green mouths (*Fig. 96*).

#### Platygyra

Ehrenberg, 1834

39F971F5-04C2-54E1-9D79-77A8412EEAD1

#### Platygyra sp. indet.

DCE5F1C4-E69A-50D6-A002-FB52A2614A03

##### Materials

- **Type status:**Other material. **Taxon:** scientificName: *Platygyra*; kingdom: Animalia; phylum: Cnidaria; class: Anthozoa; order: Scleractinia; family: Merulinidae; genus: Platygyra; scientificNameAuthorship: Ehrenberg, 1834; **Location:** waterBody: Indian Ocean; country: Seychelles; locality: Aldabra N1, Aldabra W1, Alphonse N1, Astove W1, D'Arros N1, Desroches S1, Poivre E1; minimumDepthInMeters: 10 m; maximumDepthInMeters: 250 m; locationRemarks: First Descent: Seychelles Expedition; **Identification:** identifiedBy: Gilberte Gendron, Nico Fassbender, Paris Stefanoudis, Rowana Walton; dateIdentified: 2019, 2020; identificationRemarks: identified only from imagery; **Event:** samplingProtocol: Submersible OR Remotely Operated Vehicle OR SCUBA; **Record Level:** basisOfRecord: Human observation

##### Notes

Colonies massive, dome-shaped or encrusting. Corallites forming dense meandroid valleys, giving the colony a maze-like appearance. Maximum recorded size: 40 cm across. Corallite size between 4.0 to 6.0 mm. Colour typically shades of brown or green. Can look similar to some meandering colonies of *Lobophyllia*, but the latter has typically wider ridges that are often serrated. Some colonies look also similar to *Leptoria*; however, the latter has much thinner and more meandroid walls with more uniform valleys (Fig. 97).

#### Oulophyllia

Milne Edwards & Haime, 1848

51A653A6-6458-5187-9339-93BFC6A100E2

#### Oulophyllia sp. indet.

C66D843F-B31D-50D7-BC66-823E4B4EADC6

##### Materials

- **Type status:**Other material. **Taxon:** scientificName: *Oulophyllia*; kingdom: Animalia; phylum: Cnidaria; class: Anthozoa; order: Scleractinia; family: Merulinidae; genus: Oulophyllia; scientificNameAuthorship: Milne Edwards & Haime, 1848; **Location:** waterBody: Indian Ocean; country: Seychelles; locality: Aldabra W1, Alphonse N1, Astove W1, Poivre E1; minimumDepthInMeters: 10 m; maximumDepthInMeters: 250 m; locationRemarks: First Descent: Seychelles Expedition; **Identification:** identifiedBy: Gilberte Gendron, Nico Fassbender, Paris Stefanoudis, Rowana Walton; dateIdentified: 2019, 2020; identificationRemarks: identified only from imagery; **Event:** samplingProtocol: Submersible OR Remotely Operated Vehicle OR SCUBA; **Record Level:** basisOfRecord: Human observation

##### Notes

Colonies massive appearing thickly encrusting. Maximum recorded size: 20 cm across. The colony surface consists of monocentric to meandroid, thin and ragged walls forming large valleys. Paliform lobes are commonly observed. Corallites between 12.0 to 15.0 mm in diameter. Colouration shades of brown. *Platygyra* and *Favites* appear similar, but both possess finer skeletal features (Fig. 98).

#### Plerogyridae

Rowlett, 2020

B4850A21-71AF-548B-8D90-86732FEF7A96

#### Physogyra

Quelch, 1884

F99B4066-E683-55EE-BFF2-5D885B368F24

#### Physogyra sp. indet.

E44C91F6-9D53-515A-A4B5-F65AD1DD68CB

##### Materials

- **Type status:**Other material. **Taxon:** scientificName: *Physogyra*; kingdom: Animalia; phylum: Cnidaria; class: Anthozoa; order: Scleractinia; family: Plerogyridae ; genus: Physogyra; scientificNameAuthorship: Quelch, 1884; **Location:** waterBody: Indian Ocean; country: Seychelles; locality: Aldabra N1, Aldabra W1, Alphonse N1, Desroches S1; minimumDepthInMeters: 8.8 m; maximumDepthInMeters: 53 m; locationRemarks: First Descent: Seychelles Expedition; **Identification:** identifiedBy: Gilberte Gendron, Nico Fassbender, Paris Stefanoudis, Rowana Walton; dateIdentified: 2019, 2020; identificationRemarks: identified only from imagery; **Event:** samplingProtocol: Submersible OR Remotely Operated Vehicle OR SCUBA; **Record Level:** basisOfRecord: Human observation

##### Notes

Colonies are massive or form thick plates. Colony surface entirely covered in bubble-like, teardrop-shaped vesicles. Meandroid with short widely spaced valleys that are only visible when vesicles retract. Maximum recorded size: 1.5 m across. Corallites 18.0 mm in diameter. The colouration of the vesicles is always pale-whitish (Fig. 99).

#### Pocilloporidae

Gray, 1840

6CFF8F39-DD4F-5BC6-9E7D-76075754609C

#### Pocillopora

Lamarck, 1816

67D16AF0-43CF-59E4-B165-79116BC1A60E

#### Pocillopora sp. indet.

E7E23432-3DBB-5ACE-BE8B-5ED82890A6DC

##### Materials

- **Type status:**Other material. **Taxon:** scientificName: *Pocillopora*; kingdom: Animalia; phylum: Cnidaria; class: Anthozoa; order: Scleractinia; family: Pocilloporidae; genus: Pocillopora; scientificNameAuthorship: Lamrack, 1816; **Location:** waterBody: Indian Ocean; country: Seychelles; locality: Aldabra N1, Aldabra W1, Alphonse N1, Astove W1, Poivre E1; minimumDepthInMeters: 8.8 m; maximumDepthInMeters: 23.9 m; locationRemarks: First Descent: Seychelles Expedition; **Identification:** identifiedBy: Gilberte Gendron, Nico Fassbender, Paris Stefanoudis, Rowana Walton; dateIdentified: 2019, 2020; identificationRemarks: identified only from imagery; **Event:** samplingProtocol: Submersible OR Remotely Operated Vehicle OR SCUBA; **Record Level:** basisOfRecord: Human observation

##### Notes

Colonies branching. Morphology depends on the environment, with thicker, stubby branches common in high-energy environments. Maximum recorded size: 30 cm across. Corallite size is 1.1 mm. In deeper, more sheltered waters, branches are thinner and more open. Colony surface covered in skeletal bumps (‘verrucae’) giving a rather spiky/rough appearance. The corallites’ darker colouration gives the coral a “black peppered” appearance (Fig. 100).

#### Pocillopora damicornis

(Linnaeus, 1758)

E9FD4DA6-3BB1-5519-BC3C-B843352635A6

##### Materials

- **Type status:**Other material. **Taxon:** scientificName: *Pocilloporadamicornis*; kingdom: Animalia; phylum: Cnidaria; class: Anthozoa; order: Scleractinia; family: Pocilloporidae; genus: Pocillopora; scientificNameAuthorship: Linnaeus, 1758; **Location:** waterBody: Indian Ocean; country: Seychelles; locality: Aldabra N1, Aldabra W1, Alphonse N1, Astove W1, Poivre E1; minimumDepthInMeters: 8.8 m; maximumDepthInMeters: 32 m; locationRemarks: First Descent: Seychelles Expedition; **Identification:** identifiedBy: Gilberte Gendron, Nico Fassbender, Paris Stefanoudis, Rowana Walton; dateIdentified: 2019, 2020; identificationRemarks: identified only from imagery; **Event:** samplingProtocol: Submersible OR Remotely Operated Vehicle OR SCUBA; **Record Level:** basisOfRecord: Human observation

##### Notes

Colonies are branching with branches shorter than those of other species of its genus. Morphology correlated to environmental parameters with colonies in calmer waters being more open and branched and colonies in high-energy environments appearing more compact. Maximum recorded size: 35 cm across. Colouration normally pale-brown, but can appear greenish and pink. *P.damicornis* is the only species of *Pocillopora* that we saw during our dives that could be continuously identified to species level (Fig. 101).

#### Stylophora

Schweigger, 1820

BAA9EA60-BCA3-5735-B47D-0018E91185A5

#### Stylophora sp. indet.

DE8744DA-43D1-551C-B108-688F85EC8F16

##### Materials

- **Type status:**Other material. **Taxon:** scientificName: *Stylophora*; kingdom: Animalia; phylum: Cnidaria; class: Anthozoa; order: Scleractinia; family: Pocilloporidae; genus: Stylophora; scientificNameAuthorship: Schweigger, 1820; **Location:** waterBody: Indian Ocean; country: Seychelles; locality: Aldabra N1, Alphonse N1, Astove W1, Poivre E1; minimumDepthInMeters: 9.6 m; maximumDepthInMeters: 10 m; locationRemarks: First Descent: Seychelles Expedition; **Identification:** identifiedBy: Gilberte Gendron, Nico Fassbender, Paris Stefanoudis, Rowana Walton; dateIdentified: 2019, 2020; identificationRemarks: identified only from imagery; **Event:** samplingProtocol: Submersible OR Remotely Operated Vehicle OR SCUBA; **Record Level:** basisOfRecord: Human observation

##### Notes

**Figure 102.**
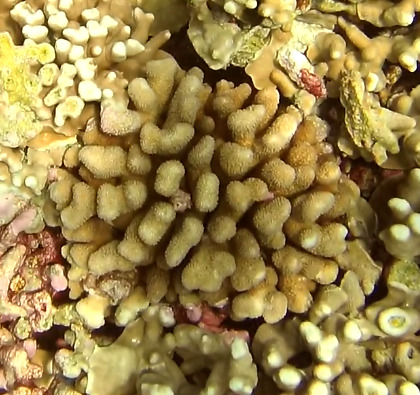
*Stylophora* sp. indet. Branching colony. Astove W1, 10 m.

Colonies branching or encrusting. Morphology is correlated to wave action energy levels of the surrounding environment - higher wave action leads to a more compact and dense growth of the colony. Branches have blunt ends and can be very thick. The surface appears rough and the corallites are very small, 1.0 mm in diameter and hooded towards the end of each branch. No verrucae present. Branches resemble teddy bear legs due to their thick appearance. Maximum recorded size: 30 cm across. Colouration pale brown. *Pocillopora* appears similar, but has verrucae on its surface and black corallites (Fig. 102).

#### Poritidae

Gray, 1840

A2570B9B-FBAF-5160-84C7-AA05EAE17CA7

#### Porites

Link, 1807

A58D236C-C93E-5767-9FE5-73EC068392DE

#### Porites sp. indet.

77E45DB0-ECBF-566C-B67C-975C46F480CE

##### Materials

- **Type status:**Other material. **Taxon:** scientificName: *Porites*; kingdom: Animalia; phylum: Cnidaria; class: Anthozoa; order: Scleractinia; family: Poritidae; genus: Porites; scientificNameAuthorship: Link, 1807; **Location:** waterBody: Indian Ocean; country: Seychelles; locality: Aldabra N1, Aldabra W1, Alphonse N1, Astove W1, D'Arros N1, Desroches S1, Poivre E1; minimumDepthInMeters: 8.8 m; maximumDepthInMeters: 63.4 m; locationRemarks: First Descent: Seychelles Expedition; **Identification:** identifiedBy: Gilberte Gendron, Nico Fassbender, Paris Stefanoudis, Rowana Walton; dateIdentified: 2019, 2020; identificationRemarks: identified only from imagery; **Event:** samplingProtocol: Submersible OR Remotely Operated Vehicle OR SCUBA; **Record Level:** basisOfRecord: Human observation

##### Notes

Massive, sub-massive, encrusting or branching colonies. Maximum recorded size up to 1 m across, except for some colonies that become hemispherical to helmed-shaped and can be several metres across. Branching colonies have stubby branches with pale tips. Corallites are very small, 0.6 to 1.3 mm and a close-up or a high-resolution camera is needed to distinguish them underwater; the surface of the colony is smooth, giving the coral an almost rock-like appearance. Colours include shades of brown or green (Fig. 103).

#### Goniopora

de Blainville, 1830

B810DDD0-7822-5C54-B367-4277138161E9

#### Goniopora sp. indet.

CDD6466C-A889-5568-BA6A-820A4B8AA506

##### Materials

- **Type status:**Other material. **Taxon:** scientificName: *Goniopora*; kingdom: Animalia; phylum: Cnidaria; class: Anthozoa; order: Scleractinia; family: Poritidae; genus: Goniopora; scientificNameAuthorship: de Blainville, 1830; **Location:** waterBody: Indian Ocean; country: Seychelles; locality: Aldabra N1, Aldabra W1, D'Arros N1, Desroches S1, Poivre E1; minimumDepthInMeters: 8.8 m; maximumDepthInMeters: 36.3 m; locationRemarks: First Descent: Seychelles Expedition; **Identification:** identifiedBy: Gilberte Gendron, Nico Fassbender, Paris Stefanoudis, Rowana Walton; dateIdentified: 2019, 2020; identificationRemarks: identified only from imagery; **Event:** samplingProtocol: Submersible OR Remotely Operated Vehicle OR SCUBA; **Record Level:** basisOfRecord: Human observation

##### Notes

**Figure 104.**
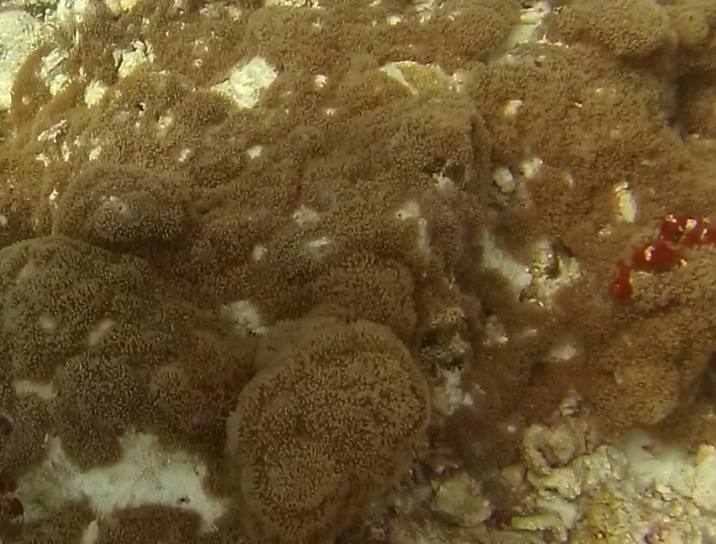
*Goniopora* sp. indet. Massive colony. Aldabra W1, 10 m.

Colonies with a range of growth forms; in this survey, mostly massive. This genus is easily recognised by the long, fleshy polyps that are extended day and night. Maximum recorded size: 1 m across. Corallites between 2.2 and 5.0 mm in size. *Alveopora* appears very similar and, if in close view, can be distinguished by the number of polyp tentacles (*Goniopora*: 24, *Alveopora*: 12) (Fig. 104).

#### Scleractinia

E3F23A8C-35C5-502D-A93F-E21C6F4B55CD

#### Pachyseris

Milne Edwards & Haime, 1849

229ABBF4-074A-5D90-B4FA-C786C3BCED4F

#### Pachyseris sp. indet.

BC02EE50-03C3-50FD-9F4C-945889D77AC9

##### Materials

- **Type status:**Other material. **Taxon:** scientificName: *Pachyseris*; kingdom: Animalia; phylum: Cnidaria; class: Anthozoa; order: Scleractinia; family: Scleractinia incertae sedis; genus: Pachyseris; scientificNameAuthorship: Milne Edwards & Haime, 1849; **Location:** waterBody: Indian Ocean; country: Seychelles; locality: Aldabra N1, Aldabra W1, Alphonse N1, Astove W1, D'Arros N1, Desroches S1, Poivre E1; minimumDepthInMeters: 8.8 m; maximumDepthInMeters: 63.4 m; locationRemarks: First Descent: Seychelles Expedition; **Identification:** identifiedBy: Gilberte Gendron, Nico Fassbender, Paris Stefanoudis, Rowana Walton; dateIdentified: 2019, 2020; identificationRemarks: identified only from imagery; **Event:** samplingProtocol: Submersible OR Remotely Operated Vehicle OR SCUBA; **Record Level:** basisOfRecord: Human observation

##### Notes

Colonies laminar and unifacial to branched and bifacial; also encrusting. Surface covered in a series of concentric ridges that are either parallel to colony margins or contorted. Corallites 0.5 mm in diameter. Most abundant in mesophotic waters, where it forms plating colonies several metres long. Colouration shades of brown with white laminar margins. *Pavona* looks similar, but possesses thicker septo-costae. Small plates can sometimes be confused with *Leptoseris*; however, the latter lacks the parallel ridges and has a bumpier, uneven surface (Fig. 105).

### Zoantharia

#### Zoantharia

Gray, 1832

B18AB8E0-6D58-5323-A98F-FF90A0F155C3

#### Zoantharia stet.

58736E85-E1CB-56B1-BFC6-CE4EC2D204ED

##### Materials

- **Type status:**Other material. **Taxon:** scientificName: Zoantharia; kingdom: Animalia; phylum: Cnidaria; class: Anthozoa; order: Zoantharia; scientificNameAuthorship: Gray, 1832; **Location:** waterBody: Indian Ocean; country: Seychelles; locality: Aldabra N1, Aldabra W1, Alphonse N1, Astove W1, Desroches S1, Poivre E1; minimumDepthInMeters: 8.8 m; maximumDepthInMeters: 31.5 m; locationRemarks: First Descent: Seychelles Expedition; **Identification:** identifiedBy: Nico Fassbender, Paris Stefanoudis; dateIdentified: 2019, 2020; identificationRemarks: identified only from imagery; **Event:** samplingProtocol: Submersible OR Remotely Operated Vehicle OR SCUBA; **Record Level:** basisOfRecord: Human observation

##### Notes

Colonies thickly encrusting to massive. Conspicuous, elliptical to round polyps, typically discernible underwater. Colour shades of creamy-white. Maximum recorded size: 50 cm across. This group likely contains a variety of species that are difficult to identify from video footage; hence, no attempt was made to identify them at a lower taxonomic level. Zoanthids can be distinguished from other Anthozoans by their tendency to incorporate sand and small pieces of the substrate into their tissue (Fig. 106).

### Hydrozoa

#### Hydrozoa

Owen, 1843

3C2CEA50-C1CA-5B4F-9B52-7CC742FDD224

#### "class Hydrozoa" stet.

0C098E50-8E3D-5218-B766-C476E2AEF19B

##### Materials

- **Type status:**Other material. **Taxon:** scientificName: Hydrozoa; kingdom: Animalia; phylum: Cnidaria; class: Hydrozoa; scientificNameAuthorship: Owen, 1843; **Location:** waterBody: Indian Ocean; country: Seychelles; locality: Aldabra N1, Aldabra W1, Alphonse N1, Astove W1, D'Arros N1, Desroches S1; minimumDepthInMeters: 15.5 m; maximumDepthInMeters: 138.5 m; locationRemarks: First Descent: Seychelles Expedition; **Identification:** identifiedBy: Nico Fassbender, Carlos Moura, Paris Stefanoudis; dateIdentified: 2019, 2020; identificationRemarks: identified only from imagery; **Event:** samplingProtocol: Submersible OR Remotely Operated Vehicle OR SCUBA; **Record Level:** basisOfRecord: Human observation

##### Notes

Often colonial, with varied branching forms and dimensions (few millimetres to few tenths of centimetres). Colouration variable. Aggregations of hydroid colonies commonly overgrow rocks and portions of dead corals. Some hydrozoans bear resemblance to octocorals, but the latter have a much more solid structure and tend to be larger. This group likely contains a variety of species that are difficult to identify from video footage; hence, no attempt was made to identify them at a lower taxonomic level. Members of the Milleporidae, Stylasteridae and Thyroscyphidae families may be more easily identifiable from video footage (see below). Commonly known as hydroids (Fig. 107).

#### Anthoathecata

Cornelius, 1992

00E93387-5977-5EA6-A9CD-017422236282

#### Milleporidae

Fleming, 1828

EFB3A700-8B67-53E9-B1A7-88F682A0DC2D

#### Millepora

Linnaeus, 1758

CCA84EE1-7DE4-5FA2-B2FC-ACE801E4727A

#### Millepora sp. indet.

492EC3C8-1948-529E-BD81-A82E7C09A484

##### Materials

- **Type status:**Other material. **Taxon:** scientificName: *Millepora* sp.; kingdom: Animalia; phylum: Cnidaria; class: Hydrozoa; order: Anthoathecata; family: Milleporidae; genus: Millepora; scientificNameAuthorship: Linnaeus, 1758; **Location:** waterBody: Indian Ocean; country: Seychelles; locality: Aldabra N1, Aldabra W1, Alphonse N1, Astove W1 Desroches S1; minimumDepthInMeters: 8.8 m; maximumDepthInMeters: 32 m; locationRemarks: First Descent: Seychelles Expedition; **Identification:** identifiedBy: Nico Fassbender, Carlos Moura, Paris Stefanoudis; dateIdentified: 2019, 2020; identificationRemarks: identified only from imagery; **Event:** samplingProtocol: Submersible OR Remotely Operated Vehicle OR SCUBA; **Record Level:** basisOfRecord: Human observation

##### Notes

Colonies encrusting or branching, either thick and heavy with lobed projections or cylindrical branches. Bumpy texture with almost no gaps between bumps, but smooth surface. Generally following the substrate, colonies can appear massive or look like large plates. Colouration pale brown to yellow or whitish. Branched colonies normally have pale to whitish tips. Maximum recorded size: 1 m across. In general, *Millepora* has a very smooth surface when compared to scleractinian corals. Encrusting colonies look similar to some species of *Sinularia*; however, the latter has wider ridges between bumps. *Millepora* is commonly known as fire coral. However, it was not possible to distinguish between distinct species from underwater images alone (Fig. 108).

#### Solanderiidae

Marshall, 1892

2EE5B768-FA72-541A-B3EF-BD14DA606548

#### Solanderia

Duchassaing & Michelin, 1846

718E1C0F-1135-5720-895A-49BFFBCC41FD

#### Solanderia sp. indet.

64A99DA6-44A2-5B0C-A309-0B810F93BFB3

##### Materials

- **Type status:**Other material. **Taxon:** scientificName: *Solanderia*; kingdom: Animalia; phylum: Cnidaria; class: Hydrozoa; order: Anthoathecata; family: Solanderiidae; genus: Solanderia; scientificNameAuthorship: Duchassaing & Michelin, 1846; **Location:** waterBody: Indian Ocean; country: Seychelles; locality: Aldabra N1; minimumDepthInMeters: 10 m; maximumDepthInMeters: 10 m; locationRemarks: First Descent: Seychelles Expedition; **Identification:** identifiedBy: Nico Fassbender, Carlos Moura, Paris Stefanoudis; dateIdentified: 2019, 2020; identificationRemarks: identified only from imagery; **Event:** samplingProtocol: Submersible OR Remotely Operated Vehicle OR SCUBA; **Record Level:** basisOfRecord: Human observation

##### Notes

**Figure 109.**
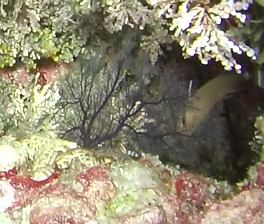
*Solanderia* sp. indet. Aldabra N1, 10 m.

Colonies are fan-shaped with fine branches typically growing in one plane. Colouration dark brown to black. Resemble sea fans, but can be distinguished by their extremely fine branches and very dark colouration. Commonly known as tree or sea fan hydroids (Fig. 109).

#### Stylasteridae

Gray, 1847

497AB533-CE86-57DD-A1A4-7A988E2D4644

#### "fam. Stylasteridae" gen. indet. sp. 1 f. sp. 1 var. sp. 1

2936CCC2-257E-54A4-A380-38DB9981DFC7

##### Materials

- **Type status:**Other material. **Taxon:** scientificName: Stylasteridae sp. 1; kingdom: Animalia; phylum: Cnidaria; class: Hydrozoa; order: Leptolida; family: Stylasteridae; scientificNameAuthorship: Gray, 1847; **Location:** waterBody: Indian Ocean; country: Seychelles; locality: Aldabra N1, Aldabra W1, Alphonse N1, Astove W1, D'Arros N1, Desroches S1; minimumDepthInMeters: 52.7 m; maximumDepthInMeters: 255.5 m; locationRemarks: First Descent: Seychelles Expedition; **Identification:** identifiedBy: Nico Fassbender, Carlos Moura, Paris Stefanoudis; dateIdentified: 2019, 2020; identificationRemarks: identified only from imagery; **Event:** samplingProtocol: Submersible OR Remotely Operated Vehicle OR SCUBA; **Record Level:** basisOfRecord: Human observation

##### Notes

Colonies delicately branched with branches ending in sympodial fashion. Maximum recorded size: 60 cm tall. Commonly growing in caves or underneath ledges. Branch surfaces are covered by dactylophores. Colouration observed was pale white, with orange, red, pink, purple and yellow possible (see the colour change in the collected specimen in Fig. 110b, SEY1_213). Commonly known as lace coral (Fig. 110).

#### "fam. Stylasteridae" gen. indet. sp. 2 cf.

6D71F002-9388-59E1-B42F-9FC48AB6B00B

##### Materials

- **Type status:**Other material. **Taxon:** scientificName: Stylasteridae sp. 2; kingdom: Animalia; phylum: Cnidaria; class: Hydrozoa; order: Leptolida; family: Stylasteridae; scientificNameAuthorship: Gray, 1847; **Location:** waterBody: Indian Ocean; country: Seychelles; locality: Aldabra W1; minimumDepthInMeters: 249.3 m; maximumDepthInMeters: 251.9 m; locationRemarks: First Descent: Seychelles Expedition; **Identification:** identifiedBy: Nico Fassbender, Carlos Moura, Paris Stefanoudis; dateIdentified: 2019, 2020; identificationRemarks: identified only from imagery; **Event:** samplingProtocol: Submersible OR Remotely Operated Vehicle OR SCUBA; **Record Level:** basisOfRecord: Human observation

##### Notes

Colonies form thin, unbranched stalks. The surface is covered in dactylopores. Colouration observed here was pale white. Maximum recorded size: 10 cm in height. Commonly known as lace coral (Fig. 111).

#### Leptothecata

Cornelius, 1992

BF6D87A7-F583-5058-9B6C-C350CEA60F39

#### Thyroscyphidae

Stechow, 1920

8AA39AE7-51C3-5C5E-8049-02F5DD132CE9

#### Thyroscyphus

Allman, 1877

8A828DE2-8341-537E-A120-9AA80A1D7E8D

#### Thyroscyphus sp. indet.

02428ECE-1795-5B05-AB8D-4A425F11E816

##### Materials

- **Type status:**Other material. **Taxon:** scientificName: *Thyroscyphus*; kingdom: Animalia; phylum: Cnidaria; class: Hydrozoa; order: Macrocolonia; family: Thyroscyphidae; genus: Thyroscyphus; scientificNameAuthorship: Allman, 1877; **Location:** waterBody: Indian Ocean; country: Seychelles; locality: Aldabra N1, Aldabra W1, Alphonse N1, Astove W1, D'Arros N1, Desroches S1; minimumDepthInMeters: 15.5 m; maximumDepthInMeters: 138.5 m; locationRemarks: First Descent: Seychelles Expedition; **Identification:** identifiedBy: Nico Fassbender, Carlos Moura, Paris Stefanoudis; dateIdentified: 2019, 2020; identificationRemarks: identified only from imagery; **Event:** samplingProtocol: Submersible OR Remotely Operated Vehicle OR SCUBA; **Record Level:** basisOfRecord: Human observation

##### Notes

Colonies irregularly branched in one plane, with stem stiff and unfascicled (i.e. with a single perisarc tube). Hydrothecae relatively prominent, pedicellated, not annulated and without nematothecae. Colouration yellowish to reddish (Fig. 112).

### Ctenophora

#### Tentaculata

Eschscholtz, 1825

240168E8-13B8-5EE0-A273-2EB69E809BC5

#### Platyctenida

Bourne, 1900

04D39AB0-DF84-5EE9-B36D-C53BCA4310A9

#### Lyroctenidae

Komai, 1942

F37ACAC8-2BE7-573C-BE5A-4B1A8C7939C9

#### Lyrocteis

Komai, 1941

72CB77A4-AE2E-598F-A79F-12B33988E1B2

#### Lyrocteis sp. indet.

73311CDB-27A1-57F2-A02A-916791383386

##### Materials

- **Type status:**Other material. **Taxon:** scientificName: *Lyrocteis*; kingdom: Animalia; phylum: Ctenophora; class: Tentaculata; order: Platyctenida; family: Lyroctenidae; genus: Lyrocteis; scientificNameAuthorship: Komai, 1941; **Location:** waterBody: Indian Ocean; country: Seychelles; locality: Aldabra N1, Aldabra W1; minimumDepthInMeters: 120 m; maximumDepthInMeters: 250.8 m; locationRemarks: First Descent: Seychelles Expedition; **Identification:** identifiedBy: Nico Fassbender, Carlos Moura, Paris Stefanoudis; dateIdentified: 2019, 2020; identificationRemarks: identified only from imagery; **Event:** samplingProtocol: Submersible OR Remotely Operated Vehicle OR SCUBA; **Record Level:** basisOfRecord: Human observation

##### Notes

While most ctenophores are present in the water column, ctenophores belonging to the order Platyctenida spend their lives on the seafloor. Those of the genus *Lyrocteis* are typically lyre-shaped and may present a great array of colours in a single species. Currently, this genus only comprises two species: *Lyrocteisflavopallidus* Robilliard & Dayton, 1972 and *Lyrocteisimperatoris* Komai, 1941, the first originally described from the Antarctic, the latter described and observed multiple times in the Pacific Ocean. Recently, an unknown species of *Lyrocteis* that resembles the present observations from Seychelles was recorded in canyons off northern KwaZulu Natal, in the Western Indian Ocean (Gibbons et al. 2021). However, it was not possible to identify these ctenophores to species level from underwater images alone without further microscopic examination (Fig. 113).

### Asteroidea

#### Asteroidea

de Blainville, 1830

ABF1309B-14B0-5164-9BD8-24E9287DCA8C

#### "class Asteroidea" ord. indet. sp. 1

927D43CD-BD5F-59DF-A5FF-FEF626C05DE4

##### Materials

- **Type status:**Other material. **Taxon:** scientificName: Asteroidea sp. 1; kingdom: Animalia; phylum: Echinodermata; class: Asteroidea; scientificNameAuthorship: de Blainville, 1830; **Location:** waterBody: Indian Ocean; country: Seychelles; locality: Astove W1, Desroches S1; minimumDepthInMeters: 114.5 m; maximumDepthInMeters: 269.5 m; locationRemarks: First Descent: Seychelles Expedition; **Identification:** identifiedBy: Nico Fassbender, Christopher Mah, Paris Stefanoudis; dateIdentified: 2019, 2020; identificationRemarks: identified only from imagery; **Event:** samplingProtocol: Submersible OR Remotely Operated Vehicle OR SCUBA; **Record Level:** basisOfRecord: Human observation

##### Notes

**Figure 114.**
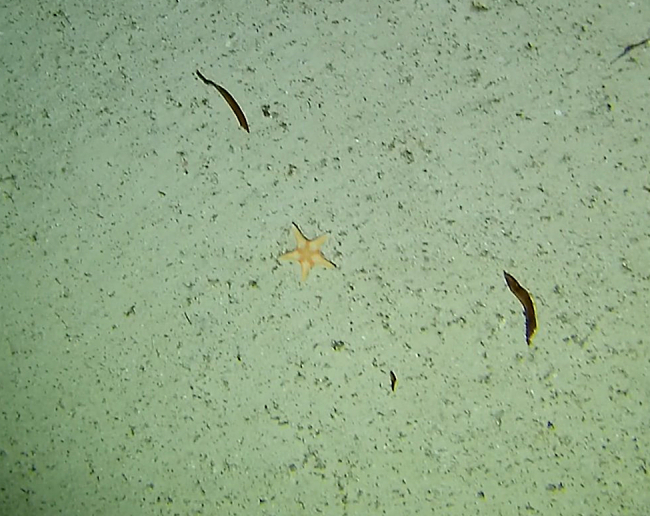
Asteroidea ord. indet. sp. 1. Desroches S1, 250 m.

Five tapered, triangular arms merging into a conspicuous central disc. Smooth body surface. Arms are light orange to yellowish with the body darker. These specimens are showing a reverse colour pattern, resembling a miniature sea star in a darker orange on the central disc between the arms. Maximum recorded size: 18 cm across (Fig. 114).

#### "class Asteroidea" ord. indet. sp. 2

97B337A2-DD5C-576D-A3D0-9DFFA02462EC

##### Materials

- **Type status:**Other material. **Taxon:** scientificName: Asteroidea ord. indet. sp. 2; kingdom: Animalia; phylum: Echinodermata; class: Asteroidea; scientificNameAuthorship: Perrier, 1884; **Location:** waterBody: Indian Ocean; country: Seychelles; locality: Desroches S1, Poivre E1; minimumDepthInMeters: 33.4 m; maximumDepthInMeters: 350 m; locationRemarks: First Descent: Seychelles Expedition; **Identification:** identifiedBy: Nico Fassbender, Christopher Mah, Paris Stefanoudis; dateIdentified: 2019, 2020; identificationRemarks: identified only from imagery; **Event:** samplingProtocol: Submersible OR Remotely Operated Vehicle OR SCUBA; **Record Level:** basisOfRecord: Human observation

##### Notes

**Figure 115.**
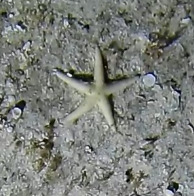
Asteroidea ord. indet. sp. 2. Desroches S1, 250 m.

Four to five slightly tubular, thick arms with rounded tips. The central disc is inconspicuous and the arms appear to almost seamlessly merge into one another at the base. The surface appears smooth. Dark blue colour, sometimes purple or light orange. Maximum recorded size: 11 cm across. The captured individuals could belong to the Ophidiasteridae or Echinasteridae families, but it was impossible to identify them to a lower taxonomic level from video footage alone (Fig. 115).

#### Forcipulatida

Perrier, 1884

CAD1E83F-0650-5110-8B6D-B23D383CB148

#### Asteriidae

Gray, 1840

D7B743AE-5405-52D4-BA5B-E98F1A1610F6

#### Coronaster

Perrier, 1885

248446E6-D6FE-514D-A2D3-A57FA793466C

#### Coronaster volsellatus

(Sladen, 1889)

927ECCB1-9974-5FDE-B209-20628F1A2362

##### Materials

- **Type status:**Other material. **Taxon:** scientificName: *Coronastervolsellatus*; kingdom: Animalia; phylum: Echinodermata; class: Asteroidea; order: Forcipulatida; family: Asteriidae; genus: Coronaster; scientificNameAuthorship: Sladen, 1889; **Location:** waterBody: Indian Ocean; country: Seychelles; locality: Aldabra N1, Alphonse N1; minimumDepthInMeters: 175 m; maximumDepthInMeters: 250 m; locationRemarks: First Descent: Seychelles Expedition; **Identification:** identifiedBy: Nico Fassbender, Christopher Mah, Paris Stefanoudis; dateIdentified: 2019, 2020; identificationRemarks: identified only from imagery; **Event:** samplingProtocol: Submersible OR Remotely Operated Vehicle OR SCUBA; **Record Level:** basisOfRecord: Human observation

##### Notes

**Figure 116.**
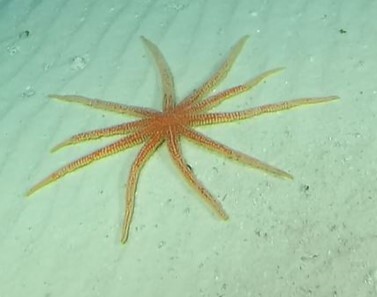
*Coronastervolsellatus*. Aldabra N1, 250 m.

Ten long and tapered arms merging into a small central disc. The surface is covered in small bumps and appears rough. Colouration is bright orange on the dorsal surface, with a lighter coloured edge around the sides of the organism (Fig. 116).

#### Coronaster sp. indet.

2E85E49D-6409-5D94-BC16-115797B0E97A

##### Materials

- **Type status:**Other material. **Taxon:** scientificName: *Coronaster* sp.; kingdom: Animalia; phylum: Echinodermata; class: Asteroidea; order: Forcipulatida; family: Asteriidae; genus: Coronaster; scientificNameAuthorship: Perrier, 1885; **Location:** waterBody: Indian Ocean; country: Seychelles; locality: Alphonse N1; minimumDepthInMeters: 150 m; maximumDepthInMeters: 172 m; locationRemarks: First Descent: Seychelles Expedition; **Identification:** identifiedBy: Nico Fassbender, Christopher Mah, Paris Stefanoudis; dateIdentified: 2019, 2020; identificationRemarks: identified only from imagery; **Event:** samplingProtocol: Submersible OR Remotely Operated Vehicle OR SCUBA; **Record Level:** basisOfRecord: Human observation

##### Notes

**Figure 117.**
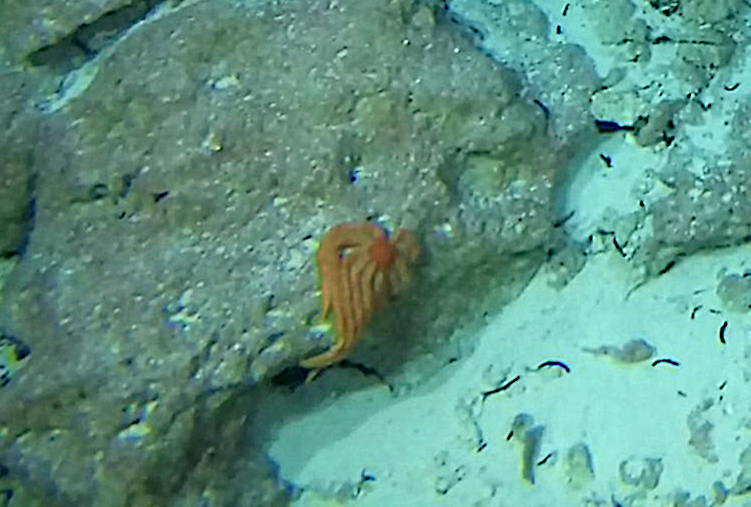
*Coronaster* sp. indet. Alphonse N1, 150 m.

Multiple long heavily bent arms merge into a conspicuously rounded central disc. The overall surface appears smooth. Arms are of light orange colour with the central disc coloured in dark orange to red (Fig. 117).

#### Sclerasterias

Perrier, 1891

572CC18B-6D7A-5F55-87F4-C3F4982AEDF5

#### Sclerasterias sp. indet.

EADC1B0E-CF2C-581C-A943-82032DAA4F48

##### Materials

- **Type status:**Other material. **Taxon:** scientificName: *Sclerasterias* sp.; kingdom: Animalia; phylum: Echinodermata; class: Asteroidea; order: Forcipulatida; family: Asteriidae; genus: Sclerasterias; scientificNameAuthorship: Perrier, 1891; **Location:** waterBody: Indian Ocean; country: Seychelles; locality: Astove W1; minimumDepthInMeters: 250 m; maximumDepthInMeters: 250 m; locationRemarks: First Descent: Seychelles Expedition; **Identification:** identifiedBy: Nico Fassbender, Christopher Mah, Paris Stefanoudis; dateIdentified: 2019, 2020; identificationRemarks: identified only from imagery; **Event:** samplingProtocol: Submersible OR Remotely Operated Vehicle OR SCUBA; **Record Level:** basisOfRecord: Human observation

##### Notes

**Figure 118.**
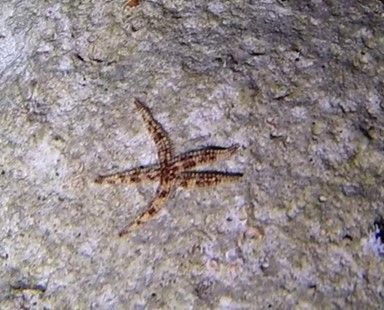
*Sclerasterias* sp. indet. Astove W1, 250 m.

Five tapered arms and a rough, spiny surface. Inconspicuous central disc. Colour of the body is beige with brown patches covering the disc and arms (Fig. 118).

#### Paxillosida

Perrier, 1884

66D97D1A-EFA4-5DC4-9414-C0C246C56230

#### Astropectinidae

Gray, 1840

69BF260B-C466-56A1-A9A8-103C855B55F4

#### "fam. Astropectinidae" gen. indet. sp.

1D242BDF-1123-5093-BEB4-D083B3AAC38C

##### Materials

- **Type status:**Other material. **Taxon:** scientificName: Astropectinidae sp.; kingdom: Animalia; phylum: Echinodermata; class: Asteroidea; order: Paxillosida; family: Astropectinidae; scientificNameAuthorship: Gray, 1840; **Location:** waterBody: Indian Ocean; country: Seychelles; locality: Alphonse N1, D'Arros N1, Desroches S1; minimumDepthInMeters: 114.5 m; maximumDepthInMeters: 269.5 m; locationRemarks: First Descent: Seychelles Expedition; **Identification:** identifiedBy: Nico Fassbender, Christopher Mah, Paris Stefanoudis; dateIdentified: 2019, 2020; identificationRemarks: identified only from imagery; **Event:** samplingProtocol: Submersible OR Remotely Operated Vehicle OR SCUBA; **Record Level:** basisOfRecord: Human observation

##### Notes

Five tapered arms merging into the central disc. Appearance varies and individuals can be slender to thick-bodied with stubby and rounded or slim and highly tapered arms. Smooth body surface. The main body is pale whitish or yellowish-orange. Approximately 7 cm across.

**Figure 119.**
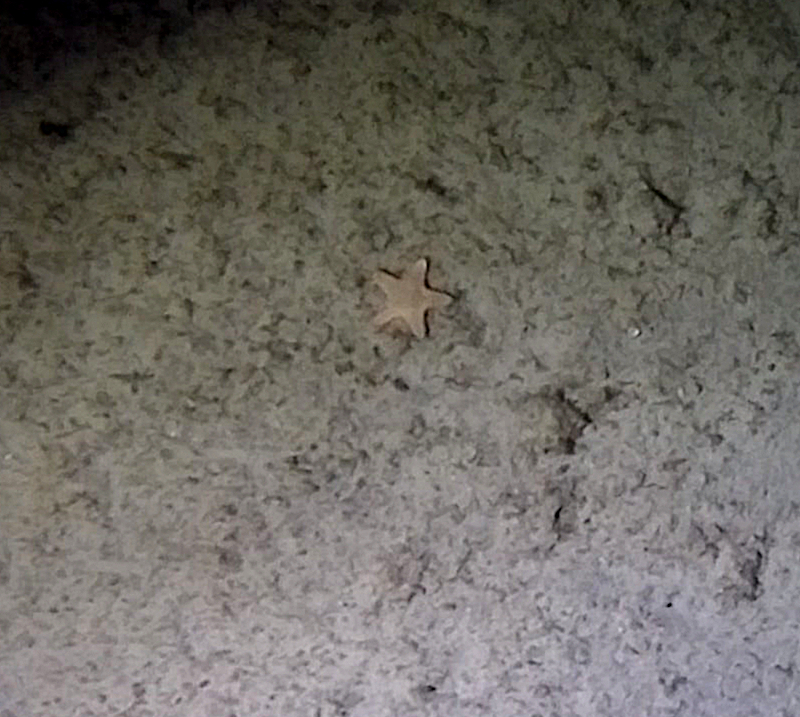
*Astropectinidae gen. indet. sp*. D'Arros N1, 350 m.

The individual might belong to *Dipsacaster* or *Leptychaster*; however, further microscopic examination is necessary for positive identification (Fig. 119).

#### Valvatida

Perrier, 1884

572762D7-70DF-5E11-8331-2BD79E06E97F

#### Asterinidae

Gray, 1840

58E4D004-18E9-5829-87A3-4CFF577AB65F

#### Nepanthia

Gray, 1840

48F38F83-0187-527C-A8A0-024FEC48B0A2

#### Nepanthia sp. indet.

8954DE2A-6568-52AC-AB2C-8939957D4A31

##### Materials

- **Type status:**Other material. **Taxon:** scientificName: *Nepanthia*; kingdom: Animalia; phylum: Echinodermata; class: Asteroidea; order: Valvatida; family: Asterinidae; genus: Nepanthia; scientificNameAuthorship: Gray, 1840; **Location:** waterBody: Indian Ocean; country: Seychelles; locality: Alphonse N1, Astove W1, D'Arros N1; minimumDepthInMeters: 244.1 m; maximumDepthInMeters: 351 m; locationRemarks: First Descent: Seychelles Expedition; **Identification:** identifiedBy: Nico Fassbender, Christopher Mah, Paris Stefanoudis; dateIdentified: 2019, 2020; identificationRemarks: identified only from imagery; **Event:** samplingProtocol: Submersible OR Remotely Operated Vehicle OR SCUBA; **Record Level:** basisOfRecord: Human observation

##### Notes

Five short tapered arms merge into a conspicuous central disc. Maximum recorded size: 15 cm across. The overall surface appears smooth, but the collected specimen displayed some very small bumpy projections. Orange colour (Fig. 120).

#### Asterodiscididae

Rowe, 1977

6C8C67EF-D998-5028-99E2-B408E2A5F1C0

#### Asterodiscides

A. M. Clark, 1974

31FC5E06-CFDC-50C8-827B-F9FD52666E0B

#### Asterodiscides sp. indet.

0DA336B7-8C36-5EF6-93A3-48F1ADB63B79

##### Materials

- **Type status:**Other material. **Taxon:** scientificName: *Asterodiscides* sp.; kingdom: Animalia; phylum: Echinodermata; class: Asteroidea; order: Valvatida; family: Asterodiscididae; genus: Asterodiscides; scientificNameAuthorship: A. M. Clark, 1974; **Location:** waterBody: Indian Ocean; country: Seychelles; locality: Alphonse N1, Poivre E1; minimumDepthInMeters: 10 m; maximumDepthInMeters: 128.3 m; locationRemarks: First Descent: Seychelles Expedition; **Identification:** identifiedBy: Nico Fassbender, Christopher Mah, Paris Stefanoudis; dateIdentified: 2019, 2020; identificationRemarks: identified only from imagery; **Event:** samplingProtocol: Submersible OR Remotely Operated Vehicle OR SCUBA; **Record Level:** basisOfRecord: Human observation

##### Notes

Five short triangular arms and a wide central disc, ~ 10 cm across. Rough surface with small bumpy projections. Colouration is a light orange mottled in darker orange patches with a conspicuous seastar-shaped darker patch in the centre (Fig. 121).

#### Goniasteridae

Forbes, 1841

C392B2EC-9D30-553B-8F62-56CE36B17A7F

#### Astroceramus

Fisher, 1906

76305A06-3C75-5961-B96B-38FD33CB417C

#### Astroceramus sp. indet.

959104F6-2D63-51AB-902D-1E93FF2ED682

##### Materials

- **Type status:**Other material. **Taxon:** scientificName: *Astroceramus* sp.; kingdom: Animalia; phylum: Echinodermata; class: Asteroidea; order: Valvatida; family: Goniasteridae; genus: Astroceramus; scientificNameAuthorship: Fisher, 1906; **Location:** waterBody: Indian Ocean; country: Seychelles; locality: Alphonse N1; minimumDepthInMeters: 250 m; maximumDepthInMeters: 250 m; locationRemarks: First Descent: Seychelles Expedition; **Identification:** identifiedBy: Nico Fassbender, Christopher Mah, Paris Stefanoudis; dateIdentified: 2019, 2020; identificationRemarks: identified only from imagery; **Event:** samplingProtocol: Submersible OR Remotely Operated Vehicle OR SCUBA; **Record Level:** basisOfRecord: Human observation

##### Notes

Five short, tapered arms and large central disc. Maximum recorded size: 10 cm across. The Latin name of this sea star translates to "star-shaped tile", describing tile-shaped plates covering the entire dorsal body surface. The main body is darker orange or yellow with slightly lighter coloured arm tips (Fig. 122).

#### Calliaster

Gray, 1840

B8990BA2-F9F8-50D9-9244-AC45B47EF861

#### Calliaster chaos

Mah, 2018

1E5D2D7A-6716-525F-9F83-B8E274EEFF45

##### Materials

- **Type status:**Other material. **Taxon:** scientificName: *Calliasterchaos*; kingdom: Animalia; phylum: Echinodermata; class: Asteroidea; order: Valvatida; family: Goniasteridae; genus: Calliaster; scientificNameAuthorship: Gray, 1840; **Location:** waterBody: Indian Ocean; country: Seychelles; locality: Desroches S1, Poivre E1; minimumDepthInMeters: 114.5 m; maximumDepthInMeters: 122.6 m; locationRemarks: First Descent: Seychelles Expedition; **Identification:** identifiedBy: Nico Fassbender, Christopher Mah, Paris Stefanoudis; dateIdentified: 2019, 2020; identificationRemarks: identified only from imagery; **Event:** samplingProtocol: Submersible OR Remotely Operated Vehicle OR SCUBA; **Record Level:** basisOfRecord: Human observation

##### Notes

**Figure 123.**
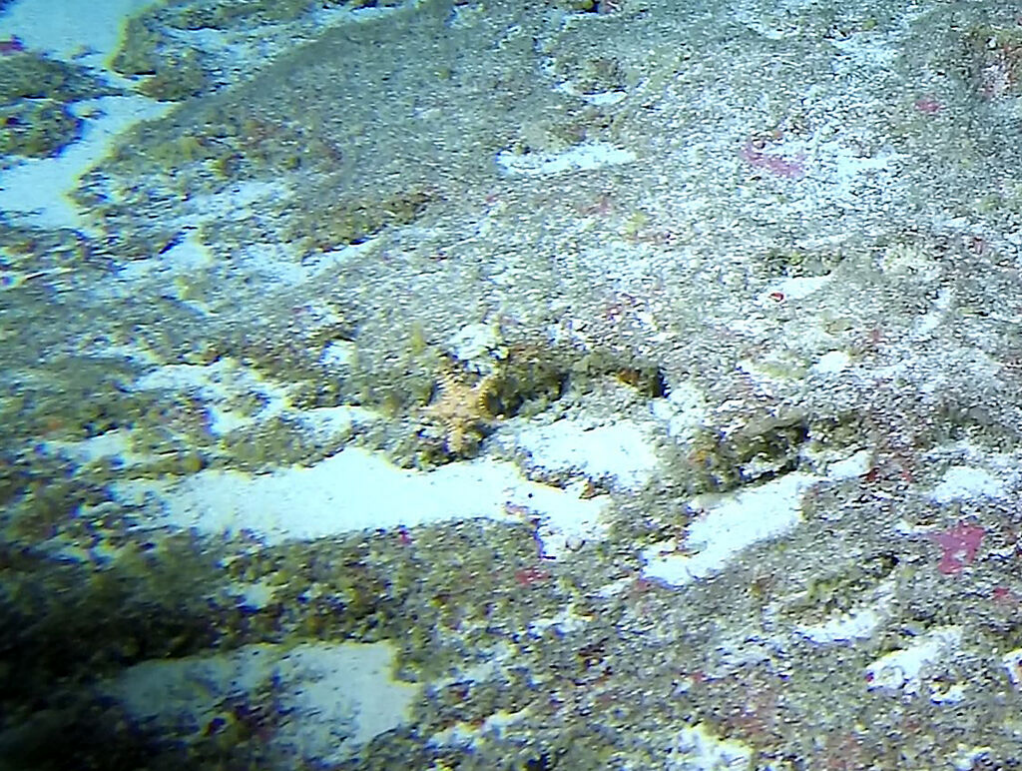
*Calliasterchaos*. Poivre E1, 120 m.

Five tapered spiny arms and a large central disc. Body surface covered in short spine-like projections and bumps. The main body is pale brown with lighter brown to pale whitish spines (Fig. 123).

#### Fromia

Gray, 1840

BC184C97-A4EF-58B7-9D9C-D4276FEC15EF

#### Fromia nodosa

A. M. Clark, 1967

8B87AE28-A769-56B6-8426-EAA7E8A3D8C6

##### Materials

- **Type status:**Other material. **Taxon:** scientificName: *Fromianodosa*; kingdom: Animalia; phylum: Echinodermata; class: Asteroidea; order: Valvatida; family: Goniasteridae; genus: Fromia; scientificNameAuthorship: A. M. Clark, 1967; **Location:** waterBody: Indian Ocean; country: Seychelles; locality: Alphonse N1, D'Arros N1, Poivre E1; minimumDepthInMeters: 33.5 m; maximumDepthInMeters: 71.4 m; locationRemarks: First Descent: Seychelles Expedition; **Identification:** identifiedBy: Nico Fassbender, Christopher Mah, Paris Stefanoudis; dateIdentified: 2019, 2020; identificationRemarks: identified only from imagery; **Event:** samplingProtocol: Submersible OR Remotely Operated Vehicle OR SCUBA; **Record Level:** basisOfRecord: Human observation

##### Notes

Five tapered arms and conspicuous central disc. The main body is dark red and the plates are creamy light brown to orange. Sometimes whitish. The central disc and the tips of the arms are darker. Maximum recorded size: 10 cm across. The collected specimen was *F.nodosa*; however, we want to mention that *Fromiamonilis* has a very similar appearance. They can be distinguished by looking at the red-tipped arms - the arms of *F.nodosa* have red tips only, whilst the arms of *F.monilis* are coloured red halfway (Fig. 124).

#### Peltaster

Verrill, 1899

3B27F660-B774-5251-A02B-F7355D7C1D6D

#### Peltaster cycloplax

Fisher, 1913

FBB23686-761A-56DC-93CF-715CCC819ADF

##### Materials

- **Type status:**Other material. **Taxon:** scientificName: *Peltastercycloplax*; kingdom: Animalia; phylum: Echinodermata; class: Asteroidea; order: Valvatida; family: Goniasteridae; genus: Peltaster; scientificNameAuthorship: Fisher, 1913; **Location:** waterBody: Indian Ocean; country: Seychelles; locality: Alphonse N1, Astove W1; minimumDepthInMeters: 250 m; maximumDepthInMeters: 250 m; locationRemarks: First Descent: Seychelles Expedition; **Identification:** identifiedBy: Nico Fassbender, Christopher Mah, Paris Stefanoudis; dateIdentified: 2019, 2020; identificationRemarks: identified only from imagery; **Event:** samplingProtocol: Submersible OR Remotely Operated Vehicle OR SCUBA; **Record Level:** basisOfRecord: Human observation

##### Notes

Five short, stubby arms and a large central disc. The edge of the starfish is slightly ridged. Maximum recorded size: 10 cm across. Darker main body with lighter edge and arm tips. Orange colour (Fig. 125).

#### Sphaeriodiscus

Fisher, 1910

4C6D33D4-22D4-591F-941D-9446A8068ED1

#### Sphaeriodiscus sp. indet.

100C855D-781B-5270-803D-18B3E9DF2C00

##### Materials

- **Type status:**Other material. **Taxon:** scientificName: *Sphaeriodiscus* sp.; kingdom: Animalia; phylum: Echinodermata; class: Asteroidea; order: Valvatida; family: Goniasteridae; genus: Sphaeriodiscus; scientificNameAuthorship: Fisher, 1910; **Location:** waterBody: Indian Ocean; country: Seychelles; locality: Alphonse N1, D'Arros N1; minimumDepthInMeters: 230 m; maximumDepthInMeters: 350 m; locationRemarks: First Descent: Seychelles Expedition; **Identification:** identifiedBy: Nico Fassbender, Christopher Mah, Paris Stefanoudis; dateIdentified: 2019, 2020; identificationRemarks: identified only from imagery; **Event:** samplingProtocol: Submersible OR Remotely Operated Vehicle OR SCUBA; **Record Level:** basisOfRecord: Human observation

##### Notes

Five short, triangular and stubby arms with a large central disc. Maximum recorded size: 8 cm across. Dark orange with arm tips appearing lighter orange to yellow (Fig. 126).

#### Ophidiasteridae

Verrill, 1870

27961A4F-6406-53AF-979D-349DA2892772

#### "fam. Ophidiasteridae" gen. indet. sp.

B5551542-9883-5BC5-971B-7CFDB44AB301

##### Materials

- **Type status:**Other material. **Taxon:** scientificName: Ophidiasteridae; kingdom: Animalia; phylum: Echinodermata; class: Asteroidea; order: Valvatida; family: Ophidiasteridae; scientificNameAuthorship: Verrill, 1870; **Location:** waterBody: Indian Ocean; country: Seychelles; locality: Alphonse N1, Astove W1, D'Arros N1, Poivre E1; minimumDepthInMeters: 89 m; maximumDepthInMeters: 251.3 m; locationRemarks: First Descent: Seychelles Expedition; **Identification:** identifiedBy: Nico Fassbender, Christopher Mah, Paris Stefanoudis; dateIdentified: 2019, 2020; identificationRemarks: identified only from imagery; **Event:** samplingProtocol: Submersible OR Remotely Operated Vehicle OR SCUBA; **Record Level:** basisOfRecord: Human observation

##### Notes

Five tapered, slender arms and inconspicuous central disc. Maximum recorded size: 19 cm across. Rough body surface covered in small bumps. The main body is pale brown to yellow, displaying cryptic mottled colour patterns that make them blend in with the substrate (Fig. 127).

#### Heteronardoa

Hayashi, 1973

A72AD371-FF00-5695-AD61-127D3E256D5B

#### Heteronardoa diamantinae

Rowe, 1976

D1C79198-00E9-5041-AE6E-48EF1890B5E4

##### Materials

- **Type status:**Other material. **Taxon:** scientificName: *Heteronardoadiamantinae*; kingdom: Animalia; phylum: Echinodermata; class: Asteroidea; order: Valvatida; family: Ophidiasteridae; genus: Heteronardoa; scientificNameAuthorship: Rowe, 1976; **Location:** waterBody: Indian Ocean; country: Seychelles; locality: D'Arros N1, Desroches S1, Poivre E1; minimumDepthInMeters: 110.7 m; maximumDepthInMeters: 351 m; locationRemarks: First Descent: Seychelles Expedition; **Identification:** identifiedBy: Nico Fassbender, Christopher Mah, Paris Stefanoudis; dateIdentified: 2019, 2020; identificationRemarks: identified only from imagery; **Event:** samplingProtocol: Submersible OR Remotely Operated Vehicle OR SCUBA; **Record Level:** basisOfRecord: Human observation

##### Notes

Five tapered slender arms merging into an inconspicuous central disc. Maximum recorded size: 18 cm across. Smooth body surface. The main body is pale orange to yellow (Fig. 128).

#### Leiaster

Peters, 1852

4470E267-D62A-5883-8F34-3773604188CA

#### Leiaster sp. indet.

B530CEBC-0045-5459-B9FA-D7C01025B541

##### Materials

- **Type status:**Other material. **Taxon:** scientificName: *Leiaster* sp.; kingdom: Animalia; phylum: Echinodermata; class: Asteroidea; order: Valvatida; family: Ophidiasteridae; genus: Leiaster; scientificNameAuthorship: Peters, 1852; **Location:** waterBody: Indian Ocean; country: Seychelles; locality: Desroches S1, Poivre E1; minimumDepthInMeters: 9.5 m; maximumDepthInMeters: 10.1 m; locationRemarks: First Descent: Seychelles Expedition; **Identification:** identifiedBy: Nico Fassbender, Christopher Mah, Paris Stefanoudis; dateIdentified: 2019, 2020; identificationRemarks: identified only from imagery; **Event:** samplingProtocol: Submersible OR Remotely Operated Vehicle OR SCUBA; **Record Level:** basisOfRecord: Human observation

##### Notes

Five tapered and slender arms and smooth body surface. The body colour is dark green (Fig. 129).

#### Oreasteridae

Fisher, 1908

88E48B86-54B6-51A2-B3A6-AE760EA6CB40

#### "fam. Oreasteridae" sp. indet.

EB094CB8-BCC6-5104-A9B7-DA6639ECE5BC

##### Materials

- **Type status:**Other material. **Taxon:** scientificName: Oreasteridae sp.; kingdom: Animalia; phylum: Echinodermata; class: Asteroidea; order: Valvatida; family: Oreasteridae; scientificNameAuthorship: Fisher, 1908; **Location:** waterBody: Indian Ocean; country: Seychelles; locality: Poivre E1; minimumDepthInMeters: 33.4 m; maximumDepthInMeters: 35 m; locationRemarks: First Descent: Seychelles Expedition; **Identification:** identifiedBy: Nico Fassbender, Christopher Mah, Paris Stefanoudis; dateIdentified: 2019, 2020; identificationRemarks: identified only from imagery; **Event:** samplingProtocol: Submersible OR Remotely Operated Vehicle OR SCUBA; **Record Level:** basisOfRecord: Human observation

##### Notes

**Figure 130.**
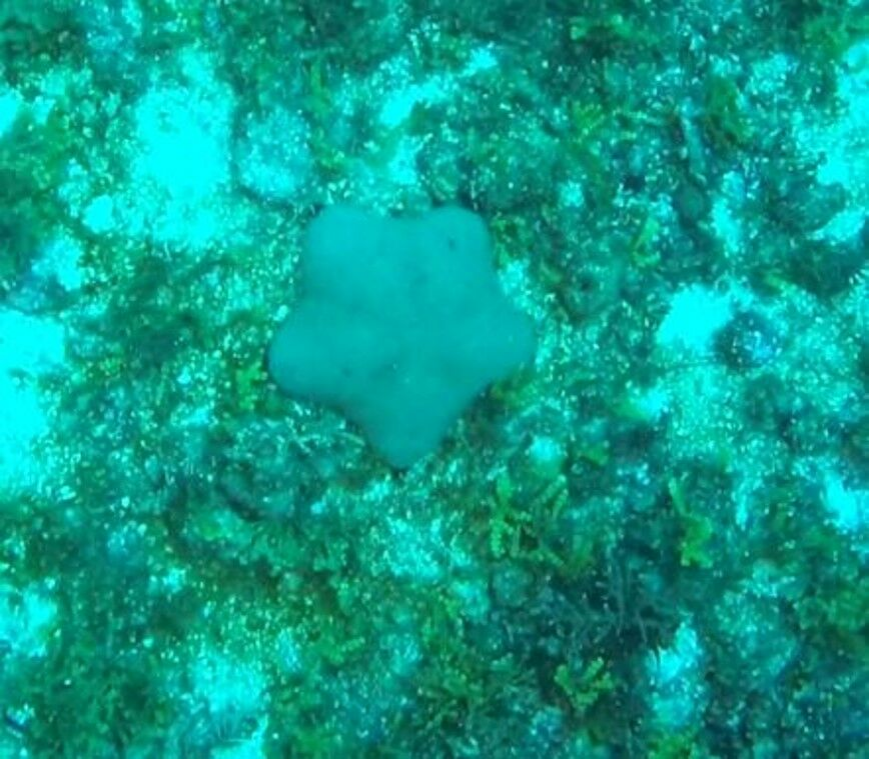
Oreasteridae sp. indet. Poivre E1, 30 m.

Five short stubby arms that rarely protrude from the wide central disc. Maximum recorded size: 23 cm across. Smooth surface. Appears roughly pentagonal in shape. Colouration is a uniform light grey. Possible genera could be *Halityle* or *Astrosarkus* (Fig. 130).

#### Culcita

Agassiz, 1836

1A93E778-0816-5391-A00E-8FFD6F918646

#### Culcita schmideliana

(Bruzelius, 1805)

8BF0B429-912C-53E2-AE44-D4A7A05A3D36

##### Materials

- **Type status:**Other material. **Taxon:** scientificName: *Culcitaschmideliana*; kingdom: Animalia; phylum: Echinodermata; class: Asteroidea; order: Valvatida; family: Oreasteridae; genus: Culcita; scientificNameAuthorship: Bruzelius, 1805; **Location:** waterBody: Indian Ocean; country: Seychelles; locality: Desroches S1, Poivre E1; minimumDepthInMeters: 33.9 m; maximumDepthInMeters: 35 m; locationRemarks: First Descent: Seychelles Expedition; **Identification:** identifiedBy: Nico Fassbender, Christopher Mah, Paris Stefanoudis; dateIdentified: 2019, 2020; identificationRemarks: identified only from imagery; **Event:** samplingProtocol: Submersible OR Remotely Operated Vehicle OR SCUBA; **Record Level:** basisOfRecord: Human observation

##### Notes

**Figure 131.**
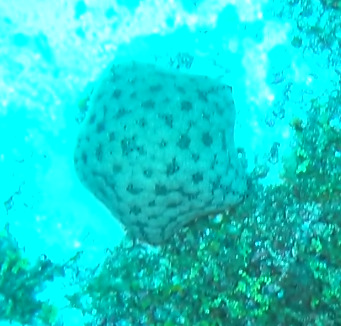
*Culcitaschmideliana*. Poivre E1, 30 m.

Five stubby, triangular arms merging into a cushion-like central disc. Maximum recorded size: 16 cm across. Roughly pentagonal appearance with a leathery surface. The aboral surface is covered in small conical spines. Colouration can vary, but most commonly a light greyish base colour with small pink patches adjacent to black tubercules (Fig. 131).

#### Halityle

Fisher, 1913

9AD282D6-5495-546E-A1F0-83540A36E911

#### Halityle regularis

Fisher, 1913

0DF8447D-BCCC-55D9-B4F4-8557B812F8E7

##### Materials

- **Type status:**Other material. **Taxon:** scientificName: *Halityleregularis*; kingdom: Animalia; phylum: Echinodermata; class: Asteroidea; order: Valvatida; family: Oreasteridae; genus: Halityle; scientificNameAuthorship: Fisher, 1913; **Location:** waterBody: Indian Ocean; country: Seychelles; locality: Poivre E1; minimumDepthInMeters: 60 m; maximumDepthInMeters: 60 m; locationRemarks: First Descent: Seychelles Expedition; **Identification:** identifiedBy: Nico Fassbender, Christopher Mah, Paris Stefanoudis; dateIdentified: 2019, 2020; identificationRemarks: identified only from imagery; **Event:** samplingProtocol: Submersible OR Remotely Operated Vehicle OR SCUBA; **Record Level:** basisOfRecord: Human observation

##### Notes

**Figure 132.**
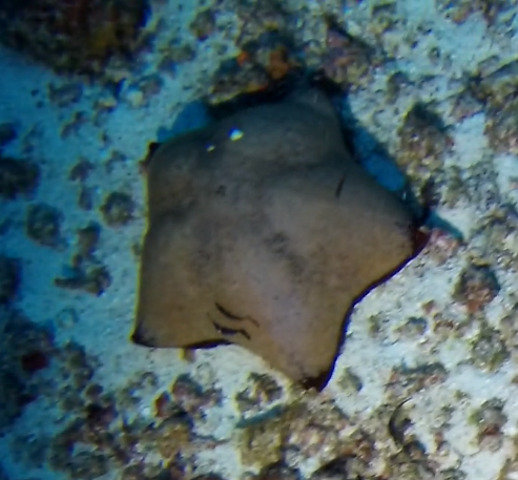
*Halityleregularis*. Poivre E1, 60 m.

Five stubby, triangular arms merging into a cushion-like central disc. Approximately 15 cm across. The body appears inflated with a smooth surface. Colour is a light brownish-grey with a dark brown edge and arm tips (Fig. 132).

### Crinoidea

#### Crinoidea

Miller, 1821

9DB12B14-9AD5-5D8C-8EF7-717666B458DC

#### "class. Crinoidea" stet.

49FE616D-0E82-552D-8F38-E1CAD49B6EF4

##### Materials

- **Type status:**Other material. **Taxon:** scientificName: Crinoidea; kingdom: Animalia; phylum: Echinodermata; class: Crinoidea; scientificNameAuthorship: Miller, 1821; **Location:** waterBody: Indian Ocean; country: Seychelles; locality: Aldabra N1, Aldabra W1, D'Arros N1, Poivre E1; minimumDepthInMeters: 30 m; maximumDepthInMeters: 350 m; locationRemarks: First Descent: Seychelles Expedition; **Identification:** identifiedBy: Nico Fassbender, Christopher Mah, Paris Stefanoudis; dateIdentified: 2019, 2020; identificationRemarks: identified only from imagery; **Event:** samplingProtocol: Submersible OR Remotely Operated Vehicle OR SCUBA; **Record Level:** basisOfRecord: Human observation

##### Notes

Can be free-swimming or anchored to the substrate by a stalk. The mouth is located on the upper surface surrounded by a crown of feeding arms. Appendages displaying pentameral symmetry are often subdivided into ten or more arms and covered in feather-like pinnules. Colours can vary, in our survey mostly dark black and white, brown, pink and yellow. Stripes commonly observed. This group likely contains a variety of species that are difficult to identify from video footage; hence, no attempt was made to identify them at a lower taxonomic level (Fig. 133).

### Echinoidea

#### Arbacioida

Gregory, 1900

E5495AC3-3F80-5889-AA28-8AFEF96D9C64

#### Arbaciidae

Gray, 1855

E19BC991-B982-5649-8B7A-B9F6B409B873

#### Coelopleurus

L. Agassiz, 1840

72510B8B-9D39-50DA-95D7-A84B4B26261C

#### Coelopleurus sp. indet.

EEF48B4E-25DA-5D0E-8636-C59065B38DD2

##### Materials

- **Type status:**Other material. **Taxon:** scientificName: *Coelepleurus* sp.; kingdom: Animalia; phylum: Echinodermata; class: Echinoidea; order: Arbacioida; family: Arbaciidae; genus: Coelopleurus; scientificNameAuthorship: L. Agassiz, 1840; **Location:** waterBody: Indian Ocean; country: Seychelles; locality: Desroches S1; minimumDepthInMeters: 230 m; maximumDepthInMeters: 230 m; locationRemarks: First Descent: Seychelles Expedition; **Identification:** identifiedBy: Nico Fassbender, Zoleka Filander, Paris Stefanoudis; dateIdentified: 2019, 2020; identificationRemarks: identified only from imagery; **Event:** samplingProtocol: Submersible OR Remotely Operated Vehicle OR SCUBA; **Record Level:** basisOfRecord: Human observation

##### Notes

Thick spines of varying lengths. The longest spines are curved, whilst shorter ones are straight. The body surface is almost entirely covered in spines, with naked and vertical spaces alternating along the body. The main body appears in a dark brown with spines being bright red and white, some with white bands towards the tips, some coloured half and half and others uniformly red. Maximum recorded size: 30 cm across. Positive species identification requires microscopic examination. Collected specimens belonged to *Coelopleurusmaillardi*.

(Fig. 134)

#### Aspidodiadematoida

Kroh & Smith, 2010

BAFA7844-9753-59ED-9BF6-AD5BB84D7A04

#### Aspidodiadematidae

Duncan, 1889

F3CD29F5-6EB9-5850-B258-2C08580E061E

#### "fam. Aspidodiadematidae" gen. indet. sp.

12A83E26-2FE7-5C40-B6DB-90067EBB582C

##### Materials

- **Type status:**Other material. **Taxon:** scientificName: Aspidodiadematidae sp.; kingdom: Animalia; phylum: Echinodermata; class: Echinoidea; order: Aspidodiadematoida; family: Aspidodiadematidae; scientificNameAuthorship: Duncan, 1889; **Location:** waterBody: Indian Ocean; country: Seychelles; locality: D'Arros N1, Poivre E1; minimumDepthInMeters: 122.4 m; maximumDepthInMeters: 251.3 m; locationRemarks: First Descent: Seychelles Expedition; **Identification:** identifiedBy: Nico Fassbender, Zoleka Filander, Paris Stefanoudis; dateIdentified: 2019, 2020; identificationRemarks: identified only from imagery; **Event:** samplingProtocol: Submersible OR Remotely Operated Vehicle OR SCUBA; **Record Level:** basisOfRecord: Human observation

##### Notes

**Figure 135.**
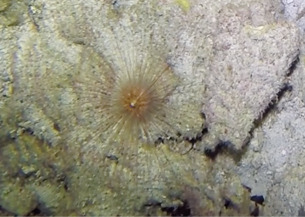
Aspidodiadematidae gen. indet. sp. D'Arros N1, 250 m.

The body surface is entirely covered in fine needle-like spines that are longer than the body is wide. Maximum recorded size: 15 cm across. The main body is appearing globular, orange-brown in colour with similar coloured spines and a conspicuous predominantly white anal cone (Fig. 135).

#### Cidaroida

Claus, 1880

4CE9D25A-568F-5CE7-96F7-ADF6EA0DC3CF

#### "ord. Cidaroida" fam. indet. sp. 1

98B1A18C-1567-514D-9BFF-52153C6F3E64

##### Materials

- **Type status:**Other material. **Taxon:** scientificName: Cidaroida sp. 1; kingdom: Animalia; phylum: Echinodermata; class: Echinoidea; order: Cidaroida; scientificNameAuthorship: Claus, 1880; **Location:** waterBody: Indian Ocean; country: Seychelles; locality: Aldabra N1, Aldabra W1, Alphonse N1, Astove W1, D'Arros N1, Desroches S1, Poivre E1; minimumDepthInMeters: 111.2 m; maximumDepthInMeters: 351 m; locationRemarks: First Descent: Seychelles Expedition; **Identification:** identifiedBy: Nico Fassbender, Zoleka Filander, Paris Stefanoudis; dateIdentified: 2019, 2020; identificationRemarks: identified only from imagery; **Event:** samplingProtocol: Submersible OR Remotely Operated Vehicle OR SCUBA; **Record Level:** basisOfRecord: Human observation

##### Notes

Thick spines pointed and longer than body width. The body surface appears smooth, but is covered with shorter spines that are encircling the attachment areas of the longer spines. Maximum recorded size: 25 cm across. Colour a pale white to cream brown. Positive species identification requires microscopic examination. Collected specimens belonged to *Histocidaris* sp. (Fig. 136).

#### "ord. Cidaroida" fam. indet. sp. 2

0A2F788F-A555-5583-91EF-25959C108E8B

##### Materials

- **Type status:**Other material. **Taxon:** scientificName: Cidaroida sp. 2; kingdom: Animalia; phylum: Echinodermata; class: Echinoidea; order: Cidaroida; scientificNameAuthorship: Claus, 1880; **Location:** waterBody: Indian Ocean; country: Seychelles; locality: Aldabra W1, Astove W1, Desroches S1; minimumDepthInMeters: 132 m; maximumDepthInMeters: 269.5 m; locationRemarks: First Descent: Seychelles Expedition; **Identification:** identifiedBy: Nico Fassbender, Zoleka Filander, Paris Stefanoudis; dateIdentified: 2019, 2020; identificationRemarks: identified only from imagery; **Event:** samplingProtocol: Submersible OR Remotely Operated Vehicle OR SCUBA; **Record Level:** basisOfRecord: Human observation

##### Notes

**Figure 137.**
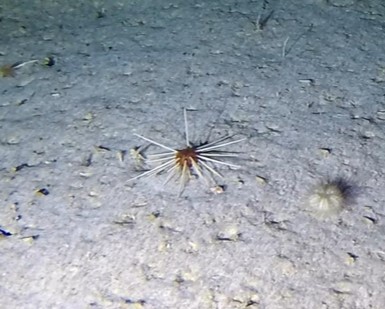
Cidaroida fam. indet. sp. 2. Astove W1, 250 m.

Thick spines longer than body width. Length of spines varies, with ventral ones shorter and dorsal ones longer. The body surface is covered in short inconspicuous spines in between larger, more prominent ones. Maximum recorded size: 10 cm across. Colour a dark red with spines pale white (Fig. 137).

#### Cidaridae

Gray, 1825

23A47E91-45B5-57AF-AB5D-E4D0AAC83F54

#### Acanthocidaris

Mortensen, 1903

51D19B5A-B0D3-5444-9252-24BB600A3CA6

#### Acanthocidaris sp. indet.

14A4768D-19DE-5723-A660-B72C9C5A425F

##### Materials

- **Type status:**Other material. **Taxon:** scientificName: *Acanthocidaris* sp.; kingdom: Animalia; phylum: Echinodermata; class: Echinoidea; order: Cidaroida; family: Cidaridae; genus: Acanthocidaris; scientificNameAuthorship: Mortensen, 1903; **Location:** waterBody: Indian Ocean; country: Seychelles; locality: Aldabra N1, Poivre E1; minimumDepthInMeters: 120 m; maximumDepthInMeters: 128.2 m; locationRemarks: First Descent: Seychelles Expedition; **Identification:** identifiedBy: Nico Fassbender, Zoleka Filander, Paris Stefanoudis; dateIdentified: 2019, 2020; identificationRemarks: identified only from imagery; **Event:** samplingProtocol: Submersible OR Remotely Operated Vehicle OR SCUBA; **Record Level:** basisOfRecord: Human observation

##### Notes

Thick, somewhat pointed spines longer than body width. The main body is very small in comparison to the spines and the body surface is almost entirely covered in spines. Maximum recorded size: 15 cm across. Colour a dark brown with the base of spines pale brown and dark brown to dark red towards the tips (Fig. 138).

#### Clypeasteroida

A. Agassiz, 1872

40ABA583-638C-526A-A280-0D22487C4710

#### Clypeasteridae

L. Agassiz, 1835

5817FFB7-59A2-5964-805B-7B268F83CAE0

#### Clypeaster

Lamarck, 1801

BF10E0F2-5BDA-57F7-8C65-BF797C50E72D

#### Clypeaster sp. indet.

73490C99-1CA3-5366-BACA-5BDB49A883C4

##### Materials

- **Type status:**Other material. **Taxon:** scientificName: *Clypeaster* sp.; kingdom: Animalia; phylum: Echinodermata; class: Echinoidea; order: Clypeasteroida; family: Clypeasteridae; genus: Clypeaster; scientificNameAuthorship: Lamarck, 1801; **Location:** waterBody: Indian Ocean; country: Seychelles; locality: Alphonse N1, Astove W1, D'Arros N1, Desroches S1, Poivre E1; minimumDepthInMeters: 124.8 m; maximumDepthInMeters: 269.5 m; locationRemarks: First Descent: Seychelles Expedition; **Identification:** identifiedBy: Nico Fassbender, Zoleka Filander, Paris Stefanoudis; dateIdentified: 2019, 2020; identificationRemarks: identified only from imagery; **Event:** samplingProtocol: Submersible OR Remotely Operated Vehicle OR SCUBA; **Record Level:** basisOfRecord: Human observation

##### Notes

The body is dome-shaped and covered in short and fine spines, giving a smooth appearance from a distance. Maximum recorded size: 15 cm across. The main body is of light brown colour with darker pores that form a pattern on the dorsal side that resembles the outline of opened petals. Found on sand or in seagrass habitats, in deeper water often covered in seagrass fragments. Positive species identification requires microscopic examination. Collected specimens belonged to *Clypeasterfervens* (Fig. 139).

#### Diadematoida

Duncan, 1889

71E97953-3AAE-5577-80CA-8687175D98C2

#### Diadematidae

Gray, 1855

005C0940-2DC3-5649-9BC2-A8C62EF0B505

#### Echinothrix

Peters, 1853

968495FC-5E28-5469-9EBE-F8D8352B96F5

#### Echinothrix diadema

(Linnaeus, 1758)

E9AFAC71-A8FD-5065-BCB4-DED5AFAC3573

##### Materials

- **Type status:**Other material. **Taxon:** scientificName: *Echinothrixdiadema*; kingdom: Animalia; phylum: Echinodermata; class: Echinoidea; order: Diadematoida; family: Diadematidae; genus: Echinothrix; scientificNameAuthorship: Linnaeus, 1758; **Location:** waterBody: Indian Ocean; country: Seychelles; locality: Poivre E1; minimumDepthInMeters: 33.5 m; maximumDepthInMeters: 36.5 m; locationRemarks: First Descent: Seychelles Expedition; **Identification:** identifiedBy: Nico Fassbender, Zoleka Filander, Paris Stefanoudis; dateIdentified: 2019, 2020; identificationRemarks: identified only from imagery; **Event:** samplingProtocol: Submersible OR Remotely Operated Vehicle OR SCUBA; **Record Level:** basisOfRecord: Human observation

##### Notes

**Figure 140.**
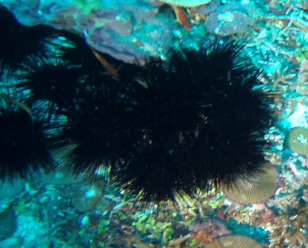
*Echinothrixdiadema*. Poivre E1, 30 m.

Spines are roughly equal to the diameter of the body. Maximum recorded size: 14 cm across. Primarily found in crevices, but can form aggregations in the open. Colour a uniform black, juveniles can have banded spines. Commonly known as Short Spine Urchin. Spines are noticeably shorter than those of *Diadema* sp. (Long Spine Urchin) (Fig. 140).

#### Micropygoida

Kroh & Smith, 2010

2C84447B-B9BF-51D4-889D-8F5C3DFE3D80

#### Micropygidae

Mortensen, 1903

2B792867-5E7A-5B6D-ACF1-BDE9123C0373

#### Micropyga

A. Agassiz, 1879

A7E9626B-23D8-50BF-B2EA-28EF35D76396

#### Micropyga sp. indet.

9DDFD043-8D15-573D-B0BB-F6A75752C516

##### Materials

- **Type status:**Other material. **Taxon:** scientificName: Micropyga sp.; kingdom: Animalia; phylum: Echinodermata; class: Echinoidea; order: Micropygoida; family: Micropygidae; genus: Micropyga; scientificNameAuthorship: A. Agassiz, 1879; **Location:** waterBody: Indian Ocean; country: Seychelles; locality: Aldabra N1, Alphonse N1, Astove W1, D'Arros N1, Desroches S1, Poivre E1; minimumDepthInMeters: 115.4 m; maximumDepthInMeters: 350 m; locationRemarks: First Descent: Seychelles Expedition; **Identification:** identifiedBy: Nico Fassbender, Zoleka Filander, Paris Stefanoudis; dateIdentified: 2019, 2020; identificationRemarks: identified only from imagery; **Event:** samplingProtocol: Submersible OR Remotely Operated Vehicle OR SCUBA; **Record Level:** basisOfRecord: Human observation

##### Notes

Spines shorter than body width and of uniform length. Maximum recorded size: 20 cm across. Colouration variable, from dark red to pale orange with distinct white bands or a pale white with distinct red bands. Positive species identification requires microscopic examination. Collected specimens belonged to Mircpoygacf.tuberculata. (Fig. 141).

#### Pedinoida

Mortensen, 1939

55259FAD-C17C-5EE7-90E4-CCBD741B5BCD

#### Pedinidae

Pomel, 1883

FB9E3EEC-FA60-55F6-BEBE-AA0A85FE4898

#### Caenopedina

A. Agassiz, 1869

1C9F0C11-2AD9-5E79-BE63-8544A3FA82AD

#### Caenopedina sp. indet.

70FC0C2D-24CE-5B45-8784-A9C0BFC09265

##### Materials

- **Type status:**Other material. **Taxon:** scientificName: *Caenopedina* sp.; kingdom: Animalia; phylum: Echinodermata; class: Echinoidea; order: Pedinoida; family: Pedinidae; genus: Caenopedina; scientificNameAuthorship: Leske, 1778; **Location:** waterBody: Indian Ocean; country: Seychelles; locality: Alphonse N1; minimumDepthInMeters: 250 m; maximumDepthInMeters: 250 m; locationRemarks: First Descent: Seychelles Expedition; **Identification:** identifiedBy: Nico Fassbender, Zoleka Filander, Paris Stefanoudis; dateIdentified: 2019, 2020; identificationRemarks: identified only from imagery; **Event:** samplingProtocol: Submersible OR Remotely Operated Vehicle OR SCUBA; **Record Level:** basisOfRecord: Human observation

##### Notes

**Figure 142.**
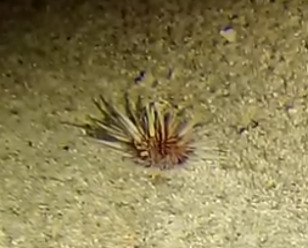
*Caenopedina* sp. indet. Alphonse N1, 250 m.

The body surface is almost entirely covered in spines of varying lengths that tend to be thicker and longer towards the dorsal surface of the main body. The main body appears in a dark red with white spines. Maximum recorded size: 6 cm across. Cidaroida fam. indet. sp. 2. appears similar, but that species has spines longer than the width of the body (Fig. 142).

#### Spatangoida

L. Agassiz, 1840

726CD6D0-AA3E-5074-B166-5C72B140A815

#### "ord. Spatangoida" fam. indet. sp.

4B23596C-63DD-5A11-8401-8FAB5E36B417

##### Materials

- **Type status:**Other material. **Taxon:** scientificName: Spatangoida sp. 1; kingdom: Animalia; phylum: Echinodermata; class: Echinoidea; order: Spatangoida; scientificNameAuthorship: L. Agassiz, 1840; **Location:** waterBody: Indian Ocean; country: Seychelles; locality: Astove W1, Poivre E1; minimumDepthInMeters: 124.8 m; maximumDepthInMeters: 350 m; locationRemarks: First Descent: Seychelles Expedition; **Identification:** identifiedBy: Nico Fassbender, Zoleka Filander, Paris Stefanoudis; dateIdentified: 2019, 2020; identificationRemarks: identified only from imagery; **Event:** samplingProtocol: Submersible OR Remotely Operated Vehicle OR SCUBA; **Record Level:** basisOfRecord: Human observation

##### Notes

Spines shorter than body width and of uniform length. The body appears egg-shaped and not as globular as other urchins observed here. Maximum recorded size: 12 cm across. Colour a dark red with distinct white patches. Spines all coloured dark red (Fig. 143).

### Holothuroidea

#### Holothuriida

Miller, Kerr, Paulay, Reich, Wilson, Carvajal & Rouse, 2017

969F9773-8A48-5BF8-B35D-28224E8154E4

#### Holothuriidae

Burmeister, 1837

FAF3206A-6C64-534B-8AAF-FE9E7F6D7061

#### Bohadschia

Jaeger, 1833

F7B42ADA-F8DE-5992-841B-DB1A65232A0E

#### Bohadschia sp. indet.

F5D54039-FEBC-50D8-8A3E-C069C1DA5B97

##### Materials

- **Type status:**Other material. **Taxon:** scientificName: *Bohadschia* sp.; kingdom: Animalia; phylum: Echinodermata; class: Holothuroidea; order: Holothuriida; family: Holothuriidae; genus: Bohadschia; scientificNameAuthorship: Jaeger, 1833; **Location:** waterBody: Indian Ocean; country: Seychelles; locality: Desroches S1; minimumDepthInMeters: 65.6 m; maximumDepthInMeters: 67.9 m; locationRemarks: First Descent: Seychelles Expedition; **Identification:** identifiedBy: Nico Fassbender, Paris Stefanoudis; dateIdentified: 2019, 2020; identificationRemarks: identified only from imagery; **Event:** samplingProtocol: Submersible OR Remotely Operated Vehicle OR SCUBA; **Record Level:** basisOfRecord: Human observation

##### Notes

**Figure 144.**
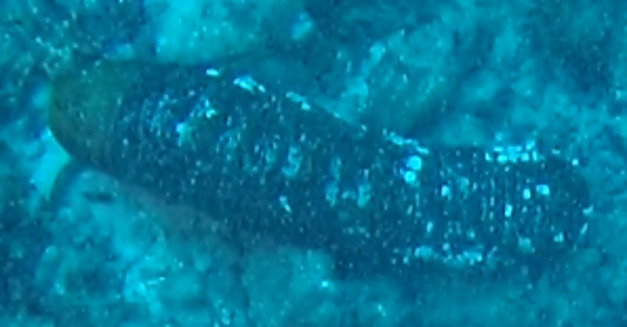
*Bohadschia* sp. indet. Desroches S1, 60 m.

Two possible species, *Bohadschiaatra* and *Bohadschiasubrubra*. *B.atra* has an elongated oblong body of dark brown to black colour, covered in conspicuous red-orange spots. *B.subrubra* is of similar body shape as the former, but has a highly variable body colour, from orange-brown to golden-white or black. Often covered in seagrass or shell fragments as seen here. Maximum recorded size: 15 cm long (Fig. 144).

#### Holothuria

Linnaeus, 1767

50F1D0A3-7950-509C-B2F8-9245C419D05A

#### Holothuria (Halodeima) atra

Jaeger, 1833

2AC53B06-E5C2-5A3E-A320-EF57B18D3EC9

##### Materials

- **Type status:**Other material. **Taxon:** scientificName: Holothuria (Halodeima) atra; kingdom: Animalia; phylum: Echinodermata; class: Holothuroidea; order: Holothuriida; family: Holothuriidae; genus: Holothuria; scientificNameAuthorship: Jaeger, 1833; **Location:** waterBody: Indian Ocean; country: Seychelles; locality: D'Arros N1, Desroches S1, Poivre E1; minimumDepthInMeters: 9.6 m; maximumDepthInMeters: 71.5 m; locationRemarks: First Descent: Seychelles Expedition; **Identification:** identifiedBy: Nico Fassbender, Paris Stefanoudis; dateIdentified: 2019, 2020; identificationRemarks: identified only from imagery; **Event:** samplingProtocol: Submersible OR Remotely Operated Vehicle OR SCUBA; **Record Level:** basisOfRecord: Human observation

##### Notes

Commonly known as "Lollyfish", this holothurian has a smooth, slender body that slightly tapers towards the ends. The body is often covered in sand, only leaving out small dotted areas on its dorsal surface. Body colour is black (Fig. 145).

#### Holothuria (Halodeima) edulis

Lesson, 1830

BD593567-01AD-5045-A891-9E8342B0B889

##### Materials

- **Type status:**Other material. **Taxon:** scientificName: Holothuria (Halodeima) edulis; kingdom: Animalia; phylum: Echinodermata; class: Holothuroidea; order: Holothuriida; family: Holothuriidae; genus: Holothuria; scientificNameAuthorship: Lesson, 1830; **Location:** waterBody: Indian Ocean; country: Seychelles; locality: Astove W1, D'Arros N1, Desroches S1; minimumDepthInMeters: 34.2 m; maximumDepthInMeters: 120 m; locationRemarks: First Descent: Seychelles Expedition; **Identification:** identifiedBy: Nico Fassbender, Paris Stefanoudis; dateIdentified: 2019, 2020; identificationRemarks: identified only from imagery; **Event:** samplingProtocol: Submersible OR Remotely Operated Vehicle OR SCUBA; **Record Level:** basisOfRecord: Human observation

##### Notes

Commonly known as "edible sea cucumber", this holothurian has a smooth body that slightly tapers towards the ends. Darker, blackish dorsal surface and reddish or beige underside. Maximum recorded size: 35 cm long (Fig. 146).

#### Synallactida

1EB791AA-2CF9-5295-ACDA-F9771BE41352

#### Stichopodidae

Haeckel, 1896

C63C02A1-A2A1-5E46-9B3F-6B1A0654C7FB

#### Stichopus

Brandt, 1835

03B6FE37-ED55-5251-9B6E-0BB6EBB67058

#### Stichopus sp. indet.

9E1A41D2-3E52-552C-BE8E-8B21B5F232F9

##### Materials

- **Type status:**Other material. **Taxon:** scientificName: *Stichopus* sp.; kingdom: Animalia; phylum: Echinodermata; class: Holothuroidea; order: Synallactida; family: Stichopodidae; genus: Stichopus; scientificNameAuthorship: Brandt, 1835; **Location:** waterBody: Indian Ocean; country: Seychelles; locality: Desroches S1; minimumDepthInMeters: 250 m; maximumDepthInMeters: 250 m; locationRemarks: First Descent: Seychelles Expedition; **Identification:** identifiedBy: Nico Fassbender, Paris Stefanoudis; dateIdentified: 2019, 2020; identificationRemarks: identified only from imagery; **Event:** samplingProtocol: Submersible OR Remotely Operated Vehicle OR SCUBA; **Record Level:** basisOfRecord: Human observation

##### Notes

**Figure 147.**
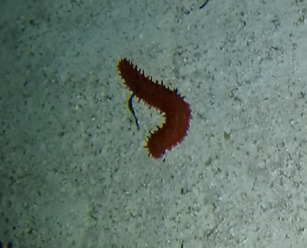
*Stichopus* sp. indet. Desroches S1, 250 m.

Body surface covered in spike-like papillae, but otherwise smooth. Square body cross-section. Approximately 10 cm long. Colouration dark red (Fig. 147).

#### Thelenota

Brandt, 1835

D977CE34-A675-5964-9FF7-5AF5AA81F4BD

#### Thelenota ananas

(Jaeger, 1833)

76D0BB3A-F042-51CD-983A-9875743254BC

##### Materials

- **Type status:**Other material. **Taxon:** scientificName: *Thelenotaananas*; kingdom: Animalia; phylum: Echinodermata; class: Holothuroidea; order: Synallactida; family: Stichopodidae; genus: Thelenota; scientificNameAuthorship: Jaeger, 1833; **Location:** waterBody: Indian Ocean; country: Seychelles; locality: Aldabra N1; minimumDepthInMeters: 10 m; maximumDepthInMeters: 10 m; locationRemarks: First Descent: Seychelles Expedition; **Identification:** identifiedBy: Nico Fassbender, Paris Stefanoudis; dateIdentified: 2019, 2020; identificationRemarks: identified only from imagery; **Event:** samplingProtocol: Submersible OR Remotely Operated Vehicle OR SCUBA; **Record Level:** basisOfRecord: Human observation

##### Notes

**Figure 148.**
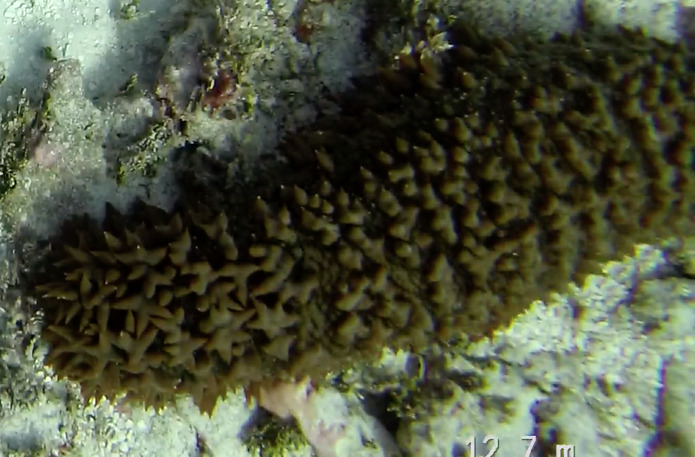
*Thelenotaananas*. Aldabra N1, 10 m.

Commonly known as "Prickly Redfish", the body surface of this sea cucumber is almost entirely covered in spike-like papillae. Large (up to 50 cm long in this survey) and heavy-bodied with a square body cross-section. Colouration bright orange to greenish in deeper waters, with pink, red and brown common in shallow water (Fig. 148).

### Annelida

#### Polychaeta

Grube, 1850

73290A37-BD8F-59AB-9990-E741C21AEA7C

#### Sabellida

Levinsen, 1883

F351D87B-7AC3-595F-858E-AA67DE5A4CDE

#### Sabellidae

Latreille, 1825

396B9B09-5E56-5813-9A83-277FE120F195

#### "fam. Sabellidae" stet.

4D6A1DFF-82B2-50F5-A66F-BB58F8AD579A

##### Materials

- **Type status:**Other material. **Taxon:** scientificName: Sabellidae sp.; kingdom: Animalia; phylum: Annelida; class: Polychaeta; order: Sabellida; family: Sabellidae; scientificNameAuthorship: Latreille, 1825; **Location:** waterBody: Indian Ocean; country: Seychelles; locality: Aldabra N1, Alphonse N1, Astove W1, D'Arros N1, Poivre E1; minimumDepthInMeters: 120 m; maximumDepthInMeters: 250 m; locationRemarks: First Descent: Seychelles Expedition; **Identification:** identifiedBy: Nico Fassbender, Paris Stefanoudis; dateIdentified: 2019, 2020; identificationRemarks: identified only from imagery; **Event:** samplingProtocol: Submersible OR Remotely Operated Vehicle OR SCUBA; **Record Level:** basisOfRecord: Human observation

##### Notes

**Figure 149.**
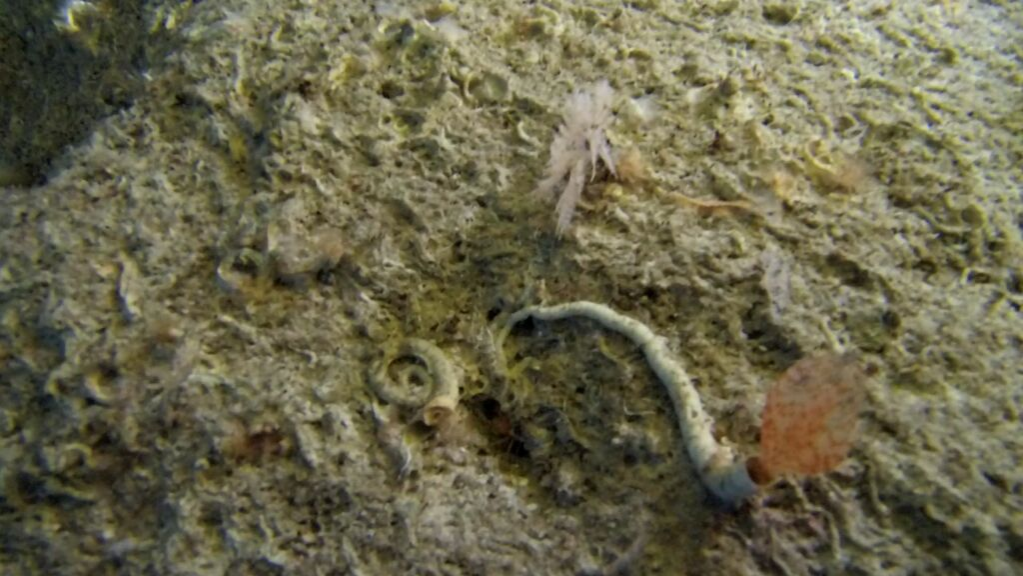
*Sabellidaestet*. Alphonse N1, 250 m.

Tube-dwelling worms with highly modified branchial crowns that form large fans. Crowns appear feathery and are separated into two clusters. Colours can vary and range from white to brown. Some species brightly coloured in pink, blue or green. Here orange. This group likely contains a variety of species that are difficult to identify from images; hence, no attempt was made to identify them at a lower taxonomic level (Fig. 149).

### Mollusca

#### Bivalvia

Linnaeus, 1758

B8AB22C7-D177-5D92-8B6B-A8717E78CE2E

#### Cardiida

Ferussac, 1822

A5A149EB-CCF8-5B88-A6BA-968F13CEEA8B

#### Cardiidae

Lamarck, 1809

80D4E38B-F9BD-5227-B611-ADA2A6F5F011

#### Tridacna

Bruguière, 1797

98DD9F79-6C59-59B7-9DEF-EA4DDB41FD9B

#### Tridacna sp. indet.

91A6ED9D-7EE5-5351-A5AB-22E5E122797B

##### Materials

- **Type status:**Other material. **Taxon:** scientificName: *Tridacna* sp.; kingdom: Animalia; phylum: Mollusca; class: Bivalvia; order: Cardiida; family: Cardiidae; genus: Tridacna; scientificNameAuthorship: Bruguière, 1797; **Location:** waterBody: Indian Ocean; country: Seychelles; locality: Aldabra W1, Alphonse N1, Desroches S1; minimumDepthInMeters: 10 m; maximumDepthInMeters: 52 m; locationRemarks: First Descent: Seychelles Expedition; **Identification:** identifiedBy: Nico Fassbender, Paris Stefanoudis; dateIdentified: 2019, 2020; identificationRemarks: identified only from imagery; **Event:** samplingProtocol: Submersible OR Remotely Operated Vehicle OR SCUBA; **Record Level:** basisOfRecord: Human observation

##### Notes

**Figure 150.**
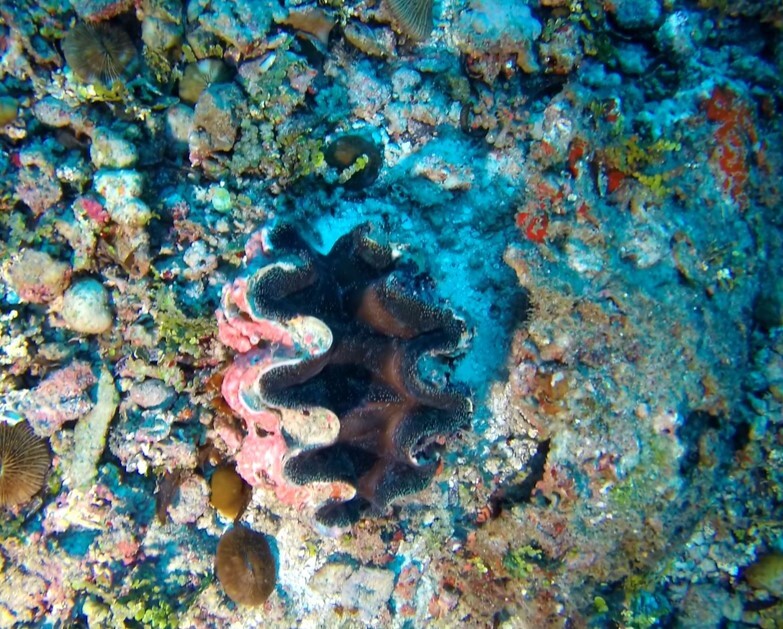
*Tridacna* sp. indet. Desroches S1, 30 m.

Extremely large-bodied clams with four to five distinct folds in its shell. The mantle is always visible, even when the shell is closed and covered in hundreds of small spots. Shell colour can vary and is often determined by organisms growing on it (such as algae or CCA), the mantle is normally of a dark brown to bluish-purple colour (Fig. 150).

### Porifera

#### Calcarea

Bowerbank, 1862

5219AD49-7510-596A-BD90-1CD620F1FCFA

#### Clathrinida

Hartman, 1958

89D43F64-3570-5385-80F7-D3E8AAF6D28A

#### Leucettidae

Laubenfels, 1936

A61BD6FF-1E02-5A56-9C73-AD8A6BE0CFFE

#### Leucetta

Haeckel, 1872

537A9D2A-3A1E-57B3-BAEE-AC8E1B8E3118

#### Leucetta chagosensis sp. inc.

Dendy, 1913

E7860684-87AD-5309-AA8C-C18FC5EEE84A

##### Materials

- **Type status:**Other material. **Taxon:** scientificName: *Leucettachagosensis*; kingdom: Animalia; phylum: Porifera; class: Calcarea; order: Clathrinida; family: Leucettidae; genus: Leucetta; scientificNameAuthorship: Dendy, 1913; **Location:** waterBody: Indian Ocean; country: Seychelles; locality: Astove W1; minimumDepthInMeters: 250 m; maximumDepthInMeters: 250 m; locationRemarks: First Descent: Seychelles Expedition; **Identification:** identifiedBy: Nico Fassbender, Toufiek Samaai, Paris Stefanoudis; dateIdentified: 2019, 2020; identificationRemarks: identified only from imagery; **Event:** samplingProtocol: Submersible OR Remotely Operated Vehicle OR SCUBA; **Record Level:** basisOfRecord: Human observation

##### Notes

**Figure 151.**
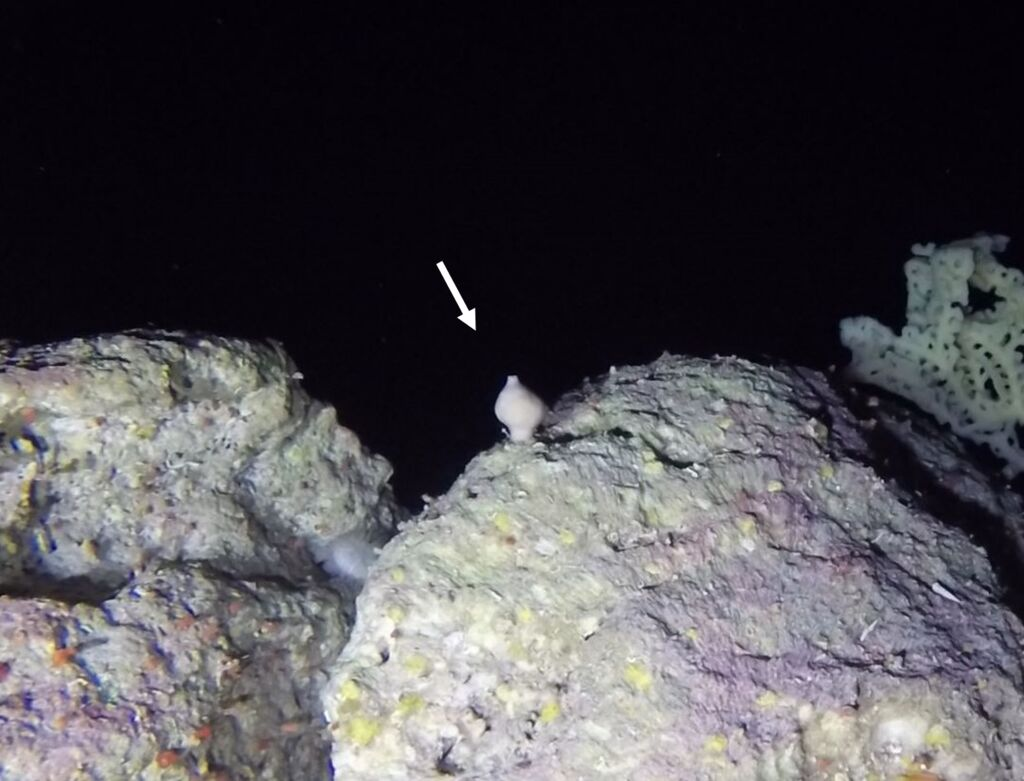
*Leucettachagosensis* sp. inc. Astove W1, 250 m.

A spherical sponge that resembles an upside-down teardrop with a tapered, singular oscule. Maximum recorded size: 5 cm long. Individuals emerge from a stubby conspicuous stem. Pale-whitish colouration (Fig. 151).

#### Demospongiae

Sollas, 1885

C38654C7-215E-5CA6-87ED-ACCB1A564CD4

#### Axinellida

Lévi, 1953

4483CB25-00F2-546C-B719-2A25A08FBF1B

#### Axinellidae

Carter, 1875

6E9267A1-4961-57D2-BB81-CA6F2B8C275C

#### Axinella

Schmidt, 1862

31082A4F-3970-53E4-B677-D7863D03BBE4

#### Axinella weltnerii

(Lendenfeld, 1897)

36A267F0-7CC7-5012-B216-E8C449080ADB

##### Materials

- **Type status:**Other material. **Taxon:** scientificName: *Axinellaweltnerii*; kingdom: Animalia; phylum: Porifera; class: Demospongiae; order: Axinellida; family: Axinellidae; genus: Axinella; scientificNameAuthorship: Lendenfeld, 1897; **Location:** waterBody: Indian Ocean; country: Seychelles; locality: Aldabra N1, Aldabra W1; minimumDepthInMeters: 30 m; maximumDepthInMeters: 30 m; locationRemarks: First Descent: Seychelles Expedition; **Identification:** identifiedBy: Nico Fassbender, Toufiek Samaai, Paris Stefanoudis; dateIdentified: 2019, 2020; identificationRemarks: identified only from imagery; **Event:** samplingProtocol: Submersible OR Remotely Operated Vehicle OR SCUBA; **Record Level:** basisOfRecord: Human observation

##### Notes

Erect flabellate sometimes fan-shaped with a short stalk. Maximum recorded size: 30 cm long. The surface appears rough, convoluted and crinkly with shallow ridges. Colour dark red (Fig. 152).

#### Clionaida

Morrow & Cárdenas, 2015

447BBBE0-7A50-5428-A509-535D0981999F

#### Clionaidae

d'Orbigny, 1851

D7CE784C-5777-5420-8D0A-FF85EE59A725

#### Spheciospongia

Marshall, 1892

C63DD089-6305-5852-81AA-EABD9280A0C7

#### Spheciospongia sp. indet. 1

5A75D60C-49A5-5883-ACE5-9FAB3C847EB0

##### Materials

- **Type status:**Other material. **Taxon:** scientificName: *Spheciospongia* sp. 1; kingdom: Animalia; phylum: Porifera; class: Demospongiae; order: Clionaida; family: Clionaidae; genus: Spheciospongia; scientificNameAuthorship: Marshall, 1892; **Location:** waterBody: Indian Ocean; country: Seychelles; locality: Aldabra N1, Aldabra W1, Astove W1, D'Arros N1, Desroches S1; minimumDepthInMeters: 10 m; maximumDepthInMeters: 72 m; locationRemarks: First Descent: Seychelles Expedition; **Identification:** identifiedBy: Nico Fassbender, Toufiek Samaai, Paris Stefanoudis; dateIdentified: 2019, 2020; identificationRemarks: identified only from imagery; **Event:** samplingProtocol: Submersible OR Remotely Operated Vehicle OR SCUBA; **Record Level:** basisOfRecord: Human observation

##### Notes

Thickly encrusting, plate-like sponges; buried in sand, with groups of tubes exposed with conspicuous terminal oscules. Maximum recorded size: 1 m across. Tubes growing more towards the centre of the individual sponge tend to be volcano-shaped and larger with multiple oscules. Colour is dark green to black (Fig. 153).

#### Spheciospongia sp. indet. 2

7E88B0EB-3EAB-5135-985F-F6766BF8C21C

##### Materials

- **Type status:**Other material. **Taxon:** scientificName: *Spheciospongia* sp. 2; kingdom: Animalia; phylum: Porifera; class: Demospongiae; order: Clionaida; family: Clionaidae; genus: Spheciospongia; scientificNameAuthorship: Marshall, 1892; **Location:** waterBody: Indian Ocean; country: Seychelles; locality: Aldabra N1, Aldabra W1, D'Arros N1; minimumDepthInMeters: 10.2 m; maximumDepthInMeters: 72 m; locationRemarks: First Descent: Seychelles Expedition; **Identification:** identifiedBy: Nico Fassbender, Toufiek Samaai, Paris Stefanoudis; dateIdentified: Nico Fassbender, Toufiek Samaai, Paris Stefanoudis; identificationRemarks: 2019, 2020; **Event:** samplingProtocol: Submersible OR Remotely Operated Vehicle OR SCUBA; **Record Level:** basisOfRecord: Human observation

##### Notes

Thickly encrusting sponges with groups of tubes clustered around one or two central, conspicuous, volcano-shaped main tubes with multiple oscules. Maximum recorded size: 50 cm across. Surface undulating and texture tough. From the top, the sponge looks like a volcano. Colour is green (Fig. 154).

#### Spheciospongia sp. indet. 3

FE2205D4-B737-59FC-BE99-214506520444

##### Materials

- **Type status:**Other material. **Taxon:** scientificName: *Spheciospongia* sp. 3; kingdom: Animalia; phylum: Porifera; class: Demospongiae; order: Clionaida; family: Clionaidae; genus: Spheciospongia; scientificNameAuthorship: Marshall, 1892; **Location:** waterBody: Indian Ocean; country: Seychelles; locality: Astove W1, D'Arros N1, Desroches S1; minimumDepthInMeters: 49.1 m; maximumDepthInMeters: 71.5 m; locationRemarks: First Descent: Seychelles Expedition; **Identification:** identifiedBy: Nico Fassbender, Toufiek Samaai, Paris Stefanoudis; dateIdentified: 2019, 2020; identificationRemarks: identified only from imagery; **Event:** samplingProtocol: Submersible OR Remotely Operated Vehicle OR SCUBA; **Record Level:** basisOfRecord: Human observation

##### Notes

Barrel sponge with a smooth surface. Maximum recorded size: 20 cm across. Singular, very conspicuous terminal oscule. Can be covered in epifauna. Colouration creamy-brown (Fig. 155).

#### Dendroceratida

Minchin, 1900

C155804B-6A09-546E-8B54-A9C4ECAFE446

#### Darwinellidae

Merejkowsky, 1879

F129AE9F-5561-5FF4-B2BA-1827B00EF337

#### Aplysilla

Schulze, 1878

EB5D5DCC-406E-55D2-8E66-AFCEA13D53D3

#### Aplysilla sp. indet.

5C7141A3-7A08-5F9F-8838-7C5806998B41

##### Materials

- **Type status:**Other material. **Taxon:** scientificName: *Aplysilla* sp.; kingdom: Animalia; phylum: Porifera; class: Demospongiae; order: Dendroceratida; family: Darwinellidae; genus: Aplysilla; scientificNameAuthorship: Schulze 1878; **Location:** waterBody: Indian Ocean; country: Seychelles; locality: Aldabra N1, Aldabra W1; minimumDepthInMeters: 10.2 m; maximumDepthInMeters: 60 m; locationRemarks: First Descent: Seychelles Expedition; **Identification:** identifiedBy: Nico Fassbender, Toufiek Samaai, Paris Stefanoudis; dateIdentified: 2019, 2020; identificationRemarks: identified only from imagery; **Event:** samplingProtocol: Submersible OR Remotely Operated Vehicle OR SCUBA; **Record Level:** basisOfRecord: Human observation

##### Notes

Thin, soft, encrusting sponge with surface uplifted into low conules, otherwise smooth. Oscules mounted at the end of oscular chimneys. Maximum recorded size: 40 cm across. Red or green colouration (Fig. 156).

#### Haplosclerida

Topsent, 1928

3715E7F6-DF90-587B-AEE4-86ABDE97234E

#### Callyspongiidae

Laubenfels, 1936

F7BFCC14-10ED-566F-AA41-375E6A6B21E2

#### Callyspongia

Duchassaing & Michelotti, 1864

02B25E0C-8C2C-5679-ACAB-D7F7EF0CECFE

#### Callyspongia sp. indet.

692ACF9D-EC67-5FFB-9011-C52CBEACFDC8

##### Materials

- **Type status:**Other material. **Taxon:** scientificName: *Callyspongia* sp.; kingdom: Animalia; phylum: Porifera; class: Demospongiae; order: Haplosclerida; family: Callyspongiidae; genus: Callyspongia; scientificNameAuthorship: Duchassing & Michelotti, 1864; **Location:** waterBody: Indian Ocean; country: Seychelles; locality: Aldabra W1, Desroches S1; minimumDepthInMeters: 20 m; maximumDepthInMeters: 64.5 m; locationRemarks: First Descent: Seychelles Expedition; **Identification:** identifiedBy: Nico Fassbender, Toufiek Samaai, Paris Stefanoudis; dateIdentified: 2019, 2020; identificationRemarks: identified only from imagery; **Event:** samplingProtocol: Submersible OR Remotely Operated Vehicle OR SCUBA; **Record Level:** basisOfRecord: Human observation

##### Notes

This sponge forms groups or chains of tubes with conspicuous terminal oscules. Tubes can become very elongated, almost fan-like in some cases. Branching originates at the base. Maximum recorded size: 40 cm across. The surface is covered with small spiny projections, giving the sponge a rough appearance. Colour ash-grey (Fig. 157).

#### Chalinidae

Gray, 1867

E3B655CB-A72F-5E5A-832F-44932776335B

#### Haliclona

Grant, 1841

E73DD69D-06EC-5451-95F4-166DC25D1783

#### Haliclona sp. indet. 1

8219B261-1EDE-5600-A458-05D356069F17

##### Materials

- **Type status:**Other material. **Taxon:** scientificName: *Haliclona* sp. 1; kingdom: Animalia; phylum: Porifera; class: Demospongiae; order: Haplosclerida; family: Chalinidae; genus: Haliclona; scientificNameAuthorship: Grant, 1841; **Location:** waterBody: Indian Ocean; country: Seychelles; locality: Alphonse N1, Poivre E1; minimumDepthInMeters: 9.7 m; maximumDepthInMeters: 53 m; locationRemarks: First Descent: Seychelles Expedition; **Identification:** identifiedBy: Nico Fassbender, Toufiek Samaai, Paris Stefanoudis; dateIdentified: 2019, 2020; identificationRemarks: identified only from imagery; **Event:** samplingProtocol: Submersible OR Remotely Operated Vehicle OR SCUBA; **Record Level:** basisOfRecord: Human observation

##### Notes

Sponges form encrusting to laterally spreading masses of branches and protrusions, shaped somewhat finger-like. Maximum recorded size ~ 30 cm. Inconspicuous oscules. Surface smooth with a velvety touch. Texture very soft. Colour greenish to grey. Other members of this group have large, irregularly spaced oscules that lack raised edges (Fig. 158).

#### Haliclona sp. indet. 2

2AA59154-E4BA-52B0-8C14-EEF2C8935A18

##### Materials

- **Type status:**Other material. **Taxon:** scientificName: *Haliclona* sp. 2; kingdom: Animalia; phylum: Porifera; class: Demospongiae; order: Haplosclerida; family: Chalinidae; genus: Haliclona; scientificNameAuthorship: Grant, 1841; **Location:** waterBody: Indian Ocean; country: Seychelles; locality: Aldabra N1, Aldabra W1, Alphonse N1, Astove W1, D'Arros N1, Poivre E1; minimumDepthInMeters: 10 m; maximumDepthInMeters: 72 m; locationRemarks: First Descent: Seychelles Expedition; **Identification:** identifiedBy: Nico Fassbender, Toufiek Samaai, Paris Stefanoudis; dateIdentified: 2019, 2020; identificationRemarks: identified only from imagery; **Event:** samplingProtocol: Submersible OR Remotely Operated Vehicle OR SCUBA; **Record Level:** basisOfRecord: Human observation

##### Notes

Morphology can vary from thin encrusting to laterally spreading masses of branches and protrusions. Maximum recorded size: 50 cm across. Can form tubes as seen above. Oscules either spread across the surface or at the top of tubes. Colour purple to grey (Fig. 159).

#### Haliclona sp. indet. 3

2B8B413A-53BA-5675-B6D7-7C6DF2DD4C9B

##### Materials

- **Type status:**Other material. **Taxon:** scientificName: *Haliclona* sp. 3; kingdom: Animalia; phylum: Porifera; class: Demospongiae; order: Haplosclerida; family: Chalinidae; genus: Haliclona; scientificNameAuthorship: Grant, 1841; **Location:** waterBody: Indian Ocean; country: Seychelles; locality: Aldabra N1, Aldabra W1, Alphonse N1; minimumDepthInMeters: 21.7 m; maximumDepthInMeters: 128 m; locationRemarks: First Descent: Seychelles Expedition; **Identification:** identifiedBy: Nico Fassbender, Toufiek Samaai, Paris Stefanoudis; dateIdentified: 2019, 2020; identificationRemarks: identified only from imagery; **Event:** samplingProtocol: Submersible OR Remotely Operated Vehicle OR SCUBA; **Record Level:** basisOfRecord: Human observation

##### Notes

Thin encrusting sponges that can have singular, chimney-like protrusions. Maximum recorded size: 15 cm across. Oscules either spread across the surface or at the top of tubes. Colour greenish to pale-grey (Fig. 160).

#### Petrosiidae

van Soest, 1980

549C770D-FA7E-5C22-B89D-22B2F6F250FE

#### "fam. Petrosiidae" gen. indet. sp. 1

A5251DEE-0435-5EC1-AB9E-F3DD9DCC78C5

##### Materials

- **Type status:**Other material. **Taxon:** scientificName: Petrosiidae sp. 1; kingdom: Animalia; phylum: Porifera; class: Demospongiae; order: Haplosclerida; family: Petrosiidae; scientificNameAuthorship: van Soest, 1980; **Location:** waterBody: Indian Ocean; country: Seychelles; locality: Aldabra N1, Aldabra W1, Alphonse N1; minimumDepthInMeters: 51 m; maximumDepthInMeters: 128 m; locationRemarks: First Descent: Seychelles Expedition; **Identification:** identifiedBy: Nico Fassbender, Toufiek Samaai, Paris Stefanoudis; dateIdentified: 2019, 2020; identificationRemarks: identified only from imagery; **Event:** samplingProtocol: Submersible OR Remotely Operated Vehicle OR SCUBA; **Record Level:** basisOfRecord: Human observation

##### Notes

**Figure 161.**
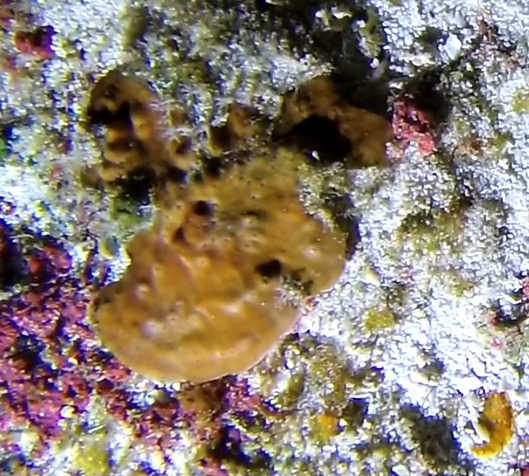
*Petrosiidae* gen. indet. sp. 1. Alphonse N1, 96 m.

Encrusting sponge with multiple small oscules prominent. Maximum recorded size: 20 cm across. The surface appears smooth. Irregular knobs or lobes are present. Might be covered in epifauna. Colour cream brown to greenish. Specimens belong to either *Petrosia* or *Neopetrosi*a; however, it is not possible to distinguish them from video footage alone without further microscopic examination (Fig. 161).

#### "fam. Petrosiidae" gen. indet. sp. 2

9EB90E72-041A-5E70-99AA-0561874CE0C0

##### Materials

- **Type status:**Other material. **Taxon:** scientificName: Petrosiidae sp. 2; kingdom: Animalia; phylum: Porifera; class: Demospongiae; order: Haplosclerida; family: Petrosiidae; scientificNameAuthorship: van Soest, 1980; **Location:** waterBody: Indian Ocean; country: Seychelles; locality: Aldabra N1, Aldabra W1, Astove W1, Desroches S1; minimumDepthInMeters: 10 m; maximumDepthInMeters: 52 m; locationRemarks: First Descent: Seychelles Expedition; **Identification:** identifiedBy: Nico Fassbender, Toufiek Samaai, Paris Stefanoudis; dateIdentified: 2019, 2020; identificationRemarks: identified only from imagery; **Event:** samplingProtocol: Submersible OR Remotely Operated Vehicle OR SCUBA; **Record Level:** basisOfRecord: Human observation

##### Notes

Encrusting sponge with multiple oscules on the surface; surface smooth with a stony appearance. Maximum recorded size: 10 cm across. Sometimes irregular knobs or lobes present. Might be covered in epifauna. Colour cream brown to orange or red. Specimens belong to either *Petrosia* or *Neopetrosi*a; however, it is not possible to distinguish them from video footage alone without further microscopic examination. It differs from Petrosiidae sp. 1 by having more prominent and conspicuous oscules (Fig. 162).

#### Petrosia

Dendy, 1905

341EAFED-EE96-570C-BA4B-E15211315FDF

#### Petrosia (Strongylophora) sp. indet.

F667AC3D-A954-5D66-8A48-54202A0E6C69

##### Materials

- **Type status:**Other material. **Taxon:** scientificName: Petrosia (Strongylophora) sp.; kingdom: Animalia; phylum: Porifera; class: Demospongiae; order: Haplosclerida; family: Petrosiidae; genus: Petrosia; subgenus: Strongylophora; scientificNameAuthorship: Dendy, 1905; **Location:** waterBody: Indian Ocean; country: Seychelles; locality: Astove W1; minimumDepthInMeters: 60 m; maximumDepthInMeters: 60 m; locationRemarks: First Descent: Seychelles Expedition; **Identification:** identifiedBy: Nico Fassbender, Toufiek Samaai, Paris Stefanoudis; dateIdentified: 2019, 2020; identificationRemarks: identified only from imagery; **Event:** samplingProtocol: Submersible OR Remotely Operated Vehicle OR SCUBA; **Record Level:** basisOfRecord: Human observation

##### Notes

A massive sponge (up to 50 cm across) that appears smooth with a stony, velvety texture. Forms irregularly shaped masses that resemble rocks. Colouration mottled with yellow, greenish and brown patches (Fig. 163).

#### Xestospongia

Laubenfels, 1932

DED89B26-FBD3-5A0B-A76B-1FB9B4DB556D

#### Xestospongia sp. indet.

2BB3FA95-BCB8-5E9C-9FDA-A7A74C7963C2

##### Materials

- **Type status:**Other material. **Taxon:** scientificName: *Xestospongia* sp. 1; kingdom: Animalia; phylum: Porifera; class: Demospongiae; order: Haplosclerida; family: Petrosiidae; genus: Xestospongia; scientificNameAuthorship: Laubenfels, 1932; **Location:** waterBody: Indian Ocean; country: Seychelles; locality: Aldabra W1; minimumDepthInMeters: 32.4 m; maximumDepthInMeters: 35.4 m; locationRemarks: First Descent: Seychelles Expedition; **Identification:** identifiedBy: Nico Fassbender, Toufiek Samaai, Paris Stefanoudis; dateIdentified: 2019, 2020; identificationRemarks: identified only from imagery; **Event:** samplingProtocol: Submersible OR Remotely Operated Vehicle OR SCUBA; **Record Level:** basisOfRecord: Human observation

##### Notes

A massive sponge that forms large barrels. The maximum recorded length was 32 cm, although they can often reach > 1 m. While the outside surface is heavily ridged, the inside appears smooth with a stony, velvety texture. Dark brown colouration (Fig. 164).

#### Phloeodictyidae

Carter, 1882

647F5AEA-FCFC-524A-ABC8-7CF8F81734B3

#### Oceanapia

Norman, 1869

96EB5F7B-108B-5647-9D79-7ABFC3603B61

#### Oceanapia sp. indet.

1B9EA5D4-118C-5600-AF5A-2E1D462A9BF2

##### Materials

- **Type status:**Other material. **Taxon:** scientificName: *Oceanapia* sp. 1; kingdom: Animalia; phylum: Porifera; class: Demospongiae; order: Haplosclerida; family: Phloeodictyidae; genus: Oceanapia; scientificNameAuthorship: Norman, 1869; **Location:** waterBody: Indian Ocean; country: Seychelles; locality: Astove W1, D'Arros N1, Desroches S1, Poivre E1; minimumDepthInMeters: 110.7 m; maximumDepthInMeters: 148.1 m; locationRemarks: First Descent: Seychelles Expedition; **Identification:** identifiedBy: Submersible OR Remotely Operated Vehicle OR SCUBA; dateIdentified: Nico Fassbender, Toufiek Samaai, Paris Stefanoudis; identificationRemarks: 2019, 2020; **Event:** samplingProtocol: identified only from imagery; **Record Level:** basisOfRecord: Human observation

##### Notes

Thick encrusting to massive sponges with a rough surface dominated by prominent oscules. Maximum recorded size: 25 cm across. Grey-whitish colouration (Fig. 165).

#### Poecilosclerida

Topsent, 1928

C1CF6236-8BFF-5868-BB63-D54B35F86116

#### Iotrochotidae

Dendy, 1922

ECD12264-99AF-5867-890C-46834D72D69F

#### Iotrochota

Ridley, 1884

73D450B9-C363-529C-B534-7E79A34609C1

#### Iotrochota nigra

(Baer, 1906)

22839BDA-9F01-572C-A09E-1290139C5089

##### Materials

- **Type status:**Other material. **Taxon:** scientificName: *Iotrochotanigra*; kingdom: Animalia; phylum: Porifera; class: Demospongiae; order: Poecilosclerida; family: Iotrochotidae; genus: Iotrochota; scientificNameAuthorship: Baer, 1906; **Location:** waterBody: Indian Ocean; country: Seychelles; locality: Aldabra N1, Aldabra W1, Desroches S1; minimumDepthInMeters: 38.4 m; maximumDepthInMeters: 120 m; locationRemarks: First Descent: Seychelles Expedition; **Identification:** identifiedBy: Nico Fassbender, Toufiek Samaai, Paris Stefanoudis; dateIdentified: 2019, 2020; identificationRemarks: identified only from imagery; **Event:** samplingProtocol: Submersible OR Remotely Operated Vehicle OR SCUBA; **Record Level:** basisOfRecord: Human observation

##### Notes

Encrusting or massive sponge up to 1 m long, with a smooth surface. Surface area covered in inconspicuous oscules across. Can be observed as a singular encrusting mass or as a group of multiple small sponges. Colouration black (Fig. 166).

#### Iotrochota sinki

Samaai, Pillay & Janson, 2019

DC52E735-F823-5D53-AF05-3CBC8017DC64

##### Materials

- **Type status:**Other material. **Taxon:** scientificName: *Iotrochotasinki*; kingdom: Animalia; phylum: Porifera; class: Demospongiae; order: Poecilosclerida; family: Iotrochotidae; genus: Iotrochota; scientificNameAuthorship: Samaai, Pillay & Janson, 2019; **Location:** waterBody: Indian Ocean; country: Seychelles; locality: Aldabra W1; minimumDepthInMeters: 58.6 m; maximumDepthInMeters: 64.5 m; locationRemarks: First Descent: Seychelles Expedition; **Identification:** identifiedBy: Nico Fassbender, Toufiek Samaai, Paris Stefanoudis; dateIdentified: 2019, 2020; identificationRemarks: identified only from imagery; **Event:** samplingProtocol: Submersible OR Remotely Operated Vehicle OR SCUBA; **Record Level:** basisOfRecord: Human observation

##### Notes

Branching sponge with thick, ramose branches and a rough surface. Maximum recorded size: 35 cm across. Colouration dark brown, but mottled throughout with bright yellow patches (Fig. 167).

#### Microcionidae

Carter, 1875

AB1B8F27-676B-5329-9C53-2D2C38BEC146

#### Calthria

Schmidt, 1862

7E4C310B-2FDD-566E-9073-C31CC59387F0

#### Clathria sp. indet.

5672D011-4493-5C2C-AC2C-A17194C8EB07

##### Materials

- **Type status:**Other material. **Taxon:** scientificName: *Clathria* sp. 1; kingdom: Animalia; phylum: Porifera; class: Demospongiae; order: Poecilosclerida; family: Microcionidae; genus: Clathria; scientificNameAuthorship: Schmidt, 1862; **Location:** waterBody: Indian Ocean; country: Seychelles; locality: Aldabra N1, Aldabra W1, Alphonse N1, Astove W1, Desroches S1, Poivre E1; minimumDepthInMeters: 9.7 m; maximumDepthInMeters: 72 m; locationRemarks: First Descent: Seychelles Expedition; **Identification:** identifiedBy: Nico Fassbender, Toufiek Samaai, Paris Stefanoudis; dateIdentified: 2019, 2020; identificationRemarks: identified only from imagery; **Event:** samplingProtocol: Submersible OR Remotely Operated Vehicle OR SCUBA; **Record Level:** basisOfRecord: Human observation

##### Notes

Thickly encrusting, sub-massive to fan-shaped morphology. Short stalk present in the fan-shaped form with thick, fleshy lobes. Maximum recorded size: 30 cm across. The surface appears rough, crinkly and conulose, corrugated longitudinally with shallow ridges. Oscules are inconspicuous and spread throughout the surface. Colour dark orange to orange-yellow (Fig. 168).

#### Scopalinida

Morrow & Cárdenas, 2015

57344643-2CD7-504E-8946-3F80DE054D9B

#### Scopalinidae

Morrow, Picton, Erpenbeck, Boury-Esnault, Maggs & Allcock, 2012

A167DAE0-3542-599F-ADC4-68F929628B04

#### Stylissa

Hallmann, 1914

154724BB-4BDF-5277-984A-44B3747875BA

#### Stylissa carteri

(Dendy, 1889)

4AA9C2FB-21E9-56B3-8943-9944EF03CF57

##### Materials

- **Type status:**Other material. **Taxon:** scientificName: *Stylissacarteri*; kingdom: Animalia; phylum: Porifera; class: Demospongiae; order: Scopalinida; family: Scopalinidae; genus: Stylissa; scientificNameAuthorship: Dendy, 1889; **Location:** waterBody: Indian Ocean; country: Seychelles; locality: Aldabra N1, Aldabra W1, Alphonse N1, Astove W1, D'Arros N1, Desroches S1; minimumDepthInMeters: 10 m; maximumDepthInMeters: 72 m; locationRemarks: First Descent: Seychelles Expedition; **Identification:** identifiedBy: Nico Fassbender, Toufiek Samaai, Paris Stefanoudis; dateIdentified: 2019, 2020; identificationRemarks: identified only from imagery; **Event:** samplingProtocol: Submersible OR Remotely Operated Vehicle OR SCUBA; **Record Level:** basisOfRecord: Human observation

##### Notes

Flabellate: either fan-shaped or branching. Forms short stalk with fleshy, blade-like lobes or erect, expanded leaves. Maximum recorded size: 31 cm across. The surface appears rough and crinkly, corrugated longitudinally with shallow ridges. Colour orange to pink.

Clathria (Thalysias) vulpine is a closely related species that cannot be distinguished from *S.carteri* without examining spicules under the microscope; however, since it has its type locality in Australia, we are considering most specimens observed to belong to *S.carteri* (Fig. 169).

#### Tetractinellida

Marshall, 1876

CEAA9724-F7DA-5FAB-A120-4D5F91C1BE8F

#### Ancorinidae

Schmidt, 1870

C213F4DF-84B0-569F-A749-F14063CA4CAD

#### Stelletta

Schmidt, 1862

6945E71B-CEB3-5DE1-BBD2-A6D90E3D33C4

#### Stelletta sp. indet.

02AB2B61-BC1F-50E5-B070-963DB7882108

##### Materials

- **Type status:**Other material. **Taxon:** scientificName: *Stelletta* sp.; kingdom: Animalia; phylum: Porifera; class: Demospongiae; order: Tetractinellida; family: Ancorinidae; genus: Stelletta; scientificNameAuthorship: Schmidt, 1862; **Location:** waterBody: Indian Ocean; country: Seychelles; locality: Aldabra W1, Astove W1; minimumDepthInMeters: 49.1 m; maximumDepthInMeters: 148.1 m; locationRemarks: First Descent: Seychelles Expedition; **Identification:** identifiedBy: Nico Fassbender, Toufiek Samaai, Paris Stefanoudis; dateIdentified: 2019, 2020; identificationRemarks: identified only from imagery; **Event:** samplingProtocol: Submersible OR Remotely Operated Vehicle OR SCUBA; **Record Level:** basisOfRecord: Human observation

##### Notes

Globular sponge with a smooth surface. Maximum recorded size: 17 cm across. Singular terminal oscule very conspicuous in the darker coloured morphotype. Colouration pale grey to a dark brown (Fig. 170).

#### Corallistidae

Sollas, 1888

2BC5C7BE-F2C9-5F7F-9524-ED76EFC2BC6B

#### Corallistes

Schmidt, 1870

C7D59BDA-5FD0-5D92-8101-3C8DD71D6AA9

#### Corallistes sp. indet.

909260D3-3D88-5127-AF05-65952A26B38A

##### Materials

- **Type status:**Other material. **Taxon:** scientificName: *Corallistes* sp.; kingdom: Animalia; phylum: Porifera; class: Demospongiae; order: Tetractinellida; family: Corallistidae; genus: Corallistes; scientificNameAuthorship: Schmidt, 1870; **Location:** waterBody: Indian Ocean; country: Seychelles; locality: Aldabra N1, Aldabra W1, Astove W1, Desroches S1; minimumDepthInMeters: 111.2 m; maximumDepthInMeters: 148.1 m; locationRemarks: First Descent: Seychelles Expedition; **Identification:** identifiedBy: Nico Fassbender, Toufiek Samaai, Paris Stefanoudis; dateIdentified: 2019, 2020; identificationRemarks: identified only from imagery; **Event:** samplingProtocol: Submersible OR Remotely Operated Vehicle OR SCUBA; **Record Level:** basisOfRecord: Human observation

##### Notes

Cup-shaped sponge with a smooth, velvety surface. Up to 22 cm across; however, the majority ~ 10 cm. The funnel-shaped cup is anchored to the substrate with a short, stubby stem. Texture hard. Colour whitish to pale yellow (Fig. 171).

#### Pachastrellidae

Carter, 1875

D8CF30FB-D8DC-573D-A1AE-B534083ED08C

#### Pachastrella

Schmidt, 1868

0A068A25-84E0-5BAB-A579-501BCA62DA88

#### Pachastrella sp. indet.

AB4C33B8-4C83-590A-B3AE-91C8B6C0EFDB

##### Materials

- **Type status:**Other material. **Taxon:** scientificName: *Pachastrella* sp.; kingdom: Animalia; phylum: Porifera; class: Demospongiae; order: Tetractinellida; family: Pachastrellidae; genus: Pachastrella; scientificNameAuthorship: Schmidt, 1868; **Location:** waterBody: Indian Ocean; country: Seychelles; locality: Aldabra N1, Aldabra W1, Alphonse N1, Astove W1, D'Arros N1, Desroches S1; minimumDepthInMeters: 51 m; maximumDepthInMeters: 252.3 m; locationRemarks: First Descent: Seychelles Expedition; **Identification:** identifiedBy: Nico Fassbender, Toufiek Samaai, Paris Stefanoudis; dateIdentified: 2019, 2020; identificationRemarks: identified only from imagery; **Event:** samplingProtocol: Submersible OR Remotely Operated Vehicle OR SCUBA; **Record Level:** basisOfRecord: Human observation

##### Notes

Massive or thickly foliose sponge with very rough, hispid surface. Maximum recorded size: 50 cm across. Rock hard texture. Colouration whitish with yellow to greenish patches (Fig. 172).

#### Scleritodermidae

Sollas, 1888

367B8D45-10AA-5F22-B4CE-E03945985E95

#### Scleritoderma

Schmidt, 1879

CC88F985-A177-5896-8FC4-4ACE1B5DC48D

#### Scleritoderma sp. indet.

1E5CF6EA-2639-5D86-BF8C-186F882FD6B7

##### Materials

- **Type status:**Other material. **Taxon:** scientificName: *Scleritoderma* sp.; kingdom: Animalia; phylum: Porifera; class: Demospongiae; order: Tetractinellida; family: Scleritodermidae; genus: Scleritoderma; scientificNameAuthorship: Schmidt, 1879; **Location:** waterBody: Indian Ocean; country: Seychelles; locality: Aldabra W1, Alphonse N1, D'Arros N1, Desroches S1; minimumDepthInMeters: 51 m; maximumDepthInMeters: 128 m; locationRemarks: First Descent: Seychelles Expedition; **Identification:** identifiedBy: Nico Fassbender, Toufiek Samaai, Paris Stefanoudis; dateIdentified: 2019, 2020; identificationRemarks: identified only from imagery; **Event:** samplingProtocol: Submersible OR Remotely Operated Vehicle OR SCUBA; **Record Level:** basisOfRecord: Human observation

##### Notes

**Figure 173.**
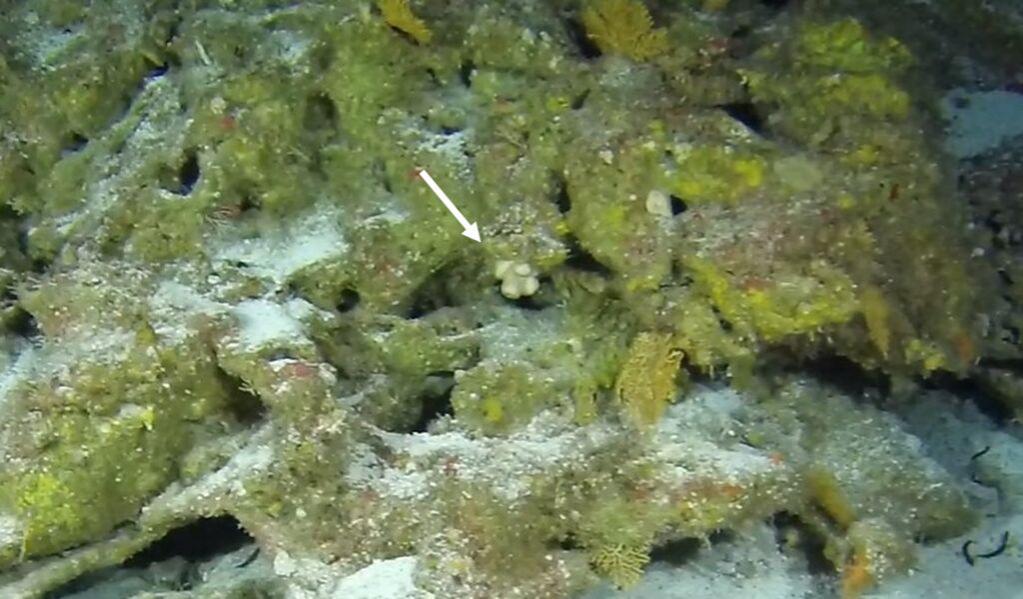
*Scleritoderma* sp. indet. Desroches S1, 120 m.

Encrusting morphology, sometimes forming shallow tubes. Maximum recorded size: 10 cm across. Large oscules at the end of tubes. Colourations pale whitish to beige (Fig. 173).

#### Tetillidae

Sollas, 1886

7CC153E1-7763-5E34-813D-2CA904C837B9

#### Tetilla

Schmidt, 1868

9CF26FA4-49CA-56F2-8A5D-1A0C7F4A3CB4

#### Tetilla sp. indet.

F741307E-F00A-550E-A5FD-8B07624F4736

##### Materials

- **Type status:**Other material. **Taxon:** scientificName: *Tetilla* sp.; kingdom: Animalia; phylum: Porifera; class: Demospongiae; order: Tetractinellida; family: Tetillidae; genus: Tetilla; scientificNameAuthorship: Schmidt, 1868; **Location:** waterBody: Indian Ocean; country: Seychelles; locality: Aldabra W1, D'Arros N1, Desroches S1, Poivre E1; minimumDepthInMeters: 58.6 m; maximumDepthInMeters: 138.5 m; locationRemarks: First Descent: Seychelles Expedition; **Identification:** identifiedBy: Nico Fassbender, Toufiek Samaai, Paris Stefanoudis; dateIdentified: 2019, 2020; identificationRemarks: identified only from imagery; **Event:** samplingProtocol: Submersible OR Remotely Operated Vehicle OR SCUBA; **Record Level:** basisOfRecord: Human observation

##### Notes

**Figure 174.**
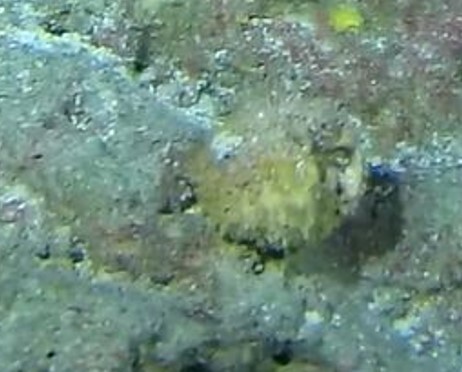
*Tetilla* sp. indet. D'Arros N1, 120 m.

Globular sponge with a warty surface; inconspicuous singular terminal oscule. Maximum recorded size: 20 cm across. Can be covered in epifauna or sediment. Colouration yellow (Fig. 174).

#### Theonellidae

Lendenfeld, 1903

F4AEA063-D631-55D5-A65B-1089E6E62504

#### Theonella

Gray, 1868

1D158A3A-24CA-564B-AA34-67D0CEE456A3

#### Theonella cf. swinhoei

Gray, 1868

D7B0A40A-E7A6-5815-9951-5FD7983D0E02

##### Materials

- **Type status:**Other material. **Taxon:** scientificName: Theonellacf.swinhoei; kingdom: Animalia; phylum: Porifera; class: Demospongiae; order: Tetractinellida; family: Theonellidae; genus: Theonella; scientificNameAuthorship: Gray, 1868; **Location:** waterBody: Indian Ocean; country: Seychelles; locality: Aldabra N1, Aldabra W1, Alphonse N1, Astove W1, Desroches S1; minimumDepthInMeters: 10 m; maximumDepthInMeters: 72 m; locationRemarks: First Descent: Seychelles Expedition; **Identification:** identifiedBy: Nico Fassbender, Toufiek Samaai, Paris Stefanoudis; dateIdentified: 2019, 2020; identificationRemarks: identified only from imagery; **Event:** samplingProtocol: Submersible OR Remotely Operated Vehicle OR SCUBA; **Record Level:** basisOfRecord: Human observation

##### Notes

Tube-shaped sponge with tubes growing in a cluster that originates from a broad base. Maximum recorded size: 40 cm across. Conspicuous oscules at the terminal end of slightly tapered tubes. Smooth, velvety surface. Maroon-brown to dark brown colouration. Can be confused with *Theonella* sp. 2, which lacks prominent tubes, appears more encrusting and is brick-red (Fig. 175).

#### Theonella sp. indet.

D1AFD68C-0554-5EFB-8099-142091B99558

##### Materials

- **Type status:**Other material. **Taxon:** scientificName: *Thonella* sp. 1; kingdom: Animalia; phylum: Porifera; class: Demospongiae; order: Tetractinellida; family: Theonellidae; genus: Theonella; scientificNameAuthorship: Gray, 1868; **Location:** waterBody: Indian Ocean; country: Seychelles; locality: Astove W1; minimumDepthInMeters: 10 m; maximumDepthInMeters: 10 m; locationRemarks: First Descent: Seychelles Expedition; **Identification:** identifiedBy: Nico Fassbender, Toufiek Samaai, Paris Stefanoudis; dateIdentified: 2019, 2020; identificationRemarks: identified only from imagery; **Event:** samplingProtocol: Submersible OR Remotely Operated Vehicle OR SCUBA; **Record Level:** basisOfRecord: Human observation

##### Notes

Thickly encrusting sponge, sometimes with shallow, bumpy tubes. Maximum recorded size: 15 cm across. Conspicuous, terminal oscules that can appear clustered on the surface, giving it a mammiform appearance. Smooth, velvety surface. Dark brick-red colouration (Fig. 176).

#### "class Demospongiae" order indet. sp. 1 f. Creamy brown encrusting sponges

7D277E8A-7EB0-5488-831F-789BC5F3787F

##### Materials

- **Type status:**Other material. **Taxon:** scientificName: Demospongiae; kingdom: Animalia; phylum: Porifera; class: Demospongiae; **Location:** waterBody: Indian Ocean; country: Seychelles; locality: Aldabra W1, Alphonse N1, Astove W1, D'Arros N1, Desroches S1, Poivre E1; minimumDepthInMeters: 9.5 m; maximumDepthInMeters: 128.3 m; locationRemarks: First Descent: Seychelles Expedition; **Identification:** identifiedBy: Nico Fassbender, Toufiek Samaai, Paris Stefanoudis; dateIdentified: 2019, 2020; identificationRemarks: identified only from imagery; **Event:** samplingProtocol: Submersible OR Remotely Operated Vehicle OR SCUBA; **Record Level:** basisOfRecord: Human observation

##### Notes

Encrusting sponge with a velvety smooth surface texture. Colouration creamy brown, sometimes mottled with yellow patches. This group likely contains a variety of species belonging to different families; however, consistent positive identification was not always possible (e.g. due to distance from the camera) and, in most cases, requires microscopic examination. Observed genera included *Aaptos* sp. (Fig. 177).

#### "class Demospongiae" order indet. sp. 2 f. Orange encrusting sponges

CC211E9F-FF59-5D06-A237-04EE7363F1D8

##### Materials

- **Type status:**Other material. **Taxon:** scientificName: Demospongiae; kingdom: Animalia; phylum: Porifera; class: Demospongiae; **Location:** waterBody: Indian Ocean; country: Seychelles; locality: Aldabra N1, Aldabra W1, Alphonse N1, Astove W1, D'Arros N1, Desroches S1, Poivre E1; minimumDepthInMeters: 8.8 m; maximumDepthInMeters: 148.1 m; locationRemarks: First Descent: Seychelles Expedition; **Identification:** identifiedBy: Nico Fassbender, Toufiek Samaai, Paris Stefanoudis; dateIdentified: 2019, 2020; identificationRemarks: identified only from imagery; **Event:** samplingProtocol: Submersible OR Remotely Operated Vehicle OR SCUBA; **Record Level:** basisOfRecord: Human observation

##### Notes

Encrusting sponges of variable thickness and surface texture. Colouration shades of orange. This group likely contains a variety of species belonging to different families; however, consistent positive identification was not always possible (e.g. due to distance from the camera) and, in most cases, requires microscopic examination. Observed genera included *Biemna* sp., *Cliona* sp. and *Petrosia* sp. (Fig. 178).

#### "class Demospongiae" order indet. sp. 3 f. Red encrusting sponges

E1B5BA20-3435-54E7-9BF2-146776B4BE87

##### Materials

- **Type status:**Other material. **Taxon:** scientificName: Demospongiae; kingdom: Animalia; phylum: Porifera; class: Demospongiae; **Location:** waterBody: Indian Ocean; country: Seychelles; locality: Aldabra N1, Aldabra W1, Alphonse N1, Astove W1, D'Arros N1, Desroches S1, Poivre E1; minimumDepthInMeters: 8.8 m; maximumDepthInMeters: 148.1 m; locationRemarks: First Descent: Seychelles Expedition; **Identification:** identifiedBy: Nico Fassbender, Toufiek Samaai, Paris Stefanoudis; dateIdentified: 2019, 2020; identificationRemarks: identified only from imagery; **Event:** samplingProtocol: Submersible OR Remotely Operated Vehicle OR SCUBA; **Record Level:** basisOfRecord: Human observation

##### Notes

Encrusting sponges of variable thickness and surface texture. Colouration bright red to dark red and red-brown. This group likely contains a variety of species belonging to different families; however, consistent positive identification was not always possible (e.g. due to distance from the camera) and, in most cases, requires microscopic examination. Observed genera included *Cliona* sp., *Raspailia* sp. and *Spirastrella* sp. (Fig. 179).

#### "class Demospongiae" order indet. sp. 4 f. Yellow encrusting sponges

465CD469-6446-5A68-B25F-DAE5555BA81E

##### Materials

- **Type status:**Other material. **Taxon:** scientificName: Demospongiae; kingdom: Animalia; phylum: Porifera; **Location:** waterBody: Indian Ocean; country: Seychelles; locality: Aldabra N1, Aldabra W1, Alphonse N1, Astove W1, D'Arros N1, Desroches S1, Poivre E1; minimumDepthInMeters: 8.8 m; maximumDepthInMeters: 249.9 m; locationRemarks: First Descent: Seychelles Expedition; **Identification:** identifiedBy: Nico Fassbender, Toufiek Samaai, Paris Stefanoudis; dateIdentified: 2019, 2020; identificationRemarks: identified only from imagery; **Event:** samplingProtocol: Submersible OR Remotely Operated Vehicle OR SCUBA; **Record Level:** basisOfRecord: Human observation

##### Notes

Encrusting sponge with a bumpy or rough surface texture. Bright yellow to pale yellow. This group likely contains a variety of species belonging to different families; however, consistent positive identification was not always possible (e.g. due to distance from the camera) and, in most cases, requires microscopic examination. Observed genera included *Cliona* sp. and *Haliclona* sp. (Fig. 180).

#### Hexactinellida

Schmidt, 1870

2357C364-8FA6-555E-A8A0-9FCF03654A1D

#### Amphidiscosida

Schrammen, 1924

6E106087-7031-585B-87C6-3AEC9DD82E06

#### Hyalonematidae

Gray, 1857

83A48154-6523-55F6-B71F-BC1EB03E462C

#### Hyalonema

Gray, 1832

0EFF91DE-3E5D-5CF2-AE18-1655BA0283A1

#### Hyalonema sp. indet.

01C24489-042E-52E9-8C22-269725087801

##### Materials

- **Type status:**Other material. **Taxon:** scientificName: *Hyalonema* sp. 1; kingdom: Animalia; phylum: Porifera; class: Hexactinellida; order: Amphidiscosida; family: Hyalonematidae; genus: Hyalonema; scientificNameAuthorship: Gray, 1832; **Location:** waterBody: Indian Ocean; country: Seychelles; locality: Aldabra W1; minimumDepthInMeters: 250 m; maximumDepthInMeters: 250 m; locationRemarks: First Descent: Seychelles Expedition; **Identification:** identifiedBy: Nico Fassbender, Toufiek Samaai, Paris Stefanoudis; dateIdentified: 2019, 2020; identificationRemarks: identified only from imagery; **Event:** samplingProtocol: Submersible OR Remotely Operated Vehicle OR SCUBA; **Record Level:** basisOfRecord: Human observation

##### Notes

**Figure 181.**
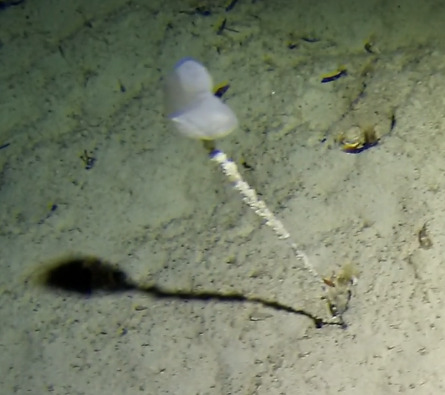
*Hyalonema* sp. indet. Aldabra W1, 250 m.

A massive-globular sponge, less than 10 cm across, that creates a single giant basal spicule to anchor itself in the sediment. Smooth surface with rough-looking “stalk”. Colouration pale white (Fig. 181).

#### Lyssacinosida

Zittel, 1877

727D5055-6C7F-5A4A-9B9C-1DD4C8B32493

#### Euplectellidae

Gray, 1867

05B0C262-C8C6-566B-A24B-CA58F708BB50

#### Heterotella

Gray, 1867

4D6FF0EF-E02B-5615-B3B8-6513D6BD2CC3

#### Heterotella corbicula

(Bowerbank, 1862)

25C44DC5-EA33-5235-B7B0-49F99A67BD34

##### Materials

- **Type status:**Other material. **Taxon:** scientificName: *Heterotellacorbicula*; kingdom: Animalia; phylum: Porifera; class: Hexactinellida; order: Lyssacinosida; family: Euplectellidae; genus: Heterotella; scientificNameAuthorship: Bowerbank, 1862; **Location:** waterBody: Indian Ocean; country: Seychelles; locality: Aldabra N1, Aldabra W1, Astove W1, D'Arros N1, Desroches S1; minimumDepthInMeters: 130.3 m; maximumDepthInMeters: 248.9 m; locationRemarks: First Descent: Seychelles Expedition; **Identification:** identifiedBy: Nico Fassbender, Toufiek Samaai, Paris Stefanoudis; dateIdentified: 2019, 2020; identificationRemarks: identified only from imagery; **Event:** samplingProtocol: Submersible OR Remotely Operated Vehicle OR SCUBA; **Record Level:** basisOfRecord: Human observation

##### Notes

Cup-shaped sponge with a smooth surface and serrated edge. Typical recorded size: 10 cm across. Individuals appear rather flat and form roughly oval masses. Pale-whitish colouration (Fig. 182).

#### Sceptrulophora

Mehl, 1992

656053B0-4FA3-56CA-A882-92E2B1B2F7EE

#### Tretodictyidae

Schulze, 1886

79E9043E-4726-59EA-AB57-3ECAF2D90C48

#### Sclerothamnus

Marshall, 1875

64A4E94A-7D76-5C66-8CCF-A51408DE6A69

#### Sclerothamnus sp. indet.

33A581A4-C5F8-5CF1-9A67-30252975E70E

##### Materials

- **Type status:**Other material. **Taxon:** scientificName: *Sclerothamnus* sp.; kingdom: Animalia; phylum: Porifera; class: Hexactinellida; order: Sceptrulophora; family: Tretodictyidae; genus: Sclerothamnus; scientificNameAuthorship: Marshall, 1875; **Location:** waterBody: Indian Ocean; country: Seychelles; locality: Aldabra N1, Aldabra W1, Astove W1, Desroches S1; minimumDepthInMeters: 130.3 m; maximumDepthInMeters: 250 m; locationRemarks: First Descent: Seychelles Expedition; **Identification:** identifiedBy: Nico Fassbender, Toufiek Samaai, Paris Stefanoudis; dateIdentified: 2019, 2020; identificationRemarks: identified only from imagery; **Event:** samplingProtocol: Submersible OR Remotely Operated Vehicle OR SCUBA; **Record Level:** basisOfRecord: Human observation

##### Notes

Branching sponge that can appear twig-like or fan-like in larger individuals due to its heavily branched appearance. Typically between 25 to 30 cm in height, although individuals can grow > 50 cm. Raised oscules that resemble corallites in hard corals. Colour light yellow. Somewhat resembles hard corals if not for the depth where it is found. (Fig. 183).

#### Homoscleromorpha

Bergquist, 1978

B9245006-C722-518B-B785-A56F431A0457

#### Homosclerophorida

Dendy, 1905

D07576EF-2CDF-503B-B0F3-BCF3F3D2066D

#### Plakinidae

Schulze, 1880

9B039302-491D-5279-89C1-FA3D16AC344E

#### Plakortis

Schulze, 1880

788F08E7-5283-5D68-B049-A051170E1C1E

#### Plakortis sp. indet.

CEE3B042-D424-5B61-A2FE-A505A913DA3A

##### Materials

- **Type status:**Other material. **Taxon:** scientificName: *Plakortis* sp.; kingdom: Animalia; phylum: Porifera; class: Homoscleromorpha; order: Homosclerophorida; family: Plakinidae; genus: Plakortis; scientificNameAuthorship: Schulze 1880; **Location:** waterBody: Indian Ocean; country: Seychelles; locality: Aldabra W1, Alphonse N1, Astove W1; minimumDepthInMeters: 10 m; maximumDepthInMeters: 72 m; locationRemarks: First Descent: Seychelles Expedition; **Identification:** identifiedBy: Nico Fassbender, Toufiek Samaai, Paris Stefanoudis; dateIdentified: 2019, 2020; identificationRemarks: identified only from imagery; **Event:** samplingProtocol: Submersible OR Remotely Operated Vehicle OR SCUBA; **Record Level:** basisOfRecord: Human observation

##### Notes

Encrusting sponge with multiple small oscules; surface smooth, but irregular, uneven with a soft texture. Maximum recorded size: 30 cm across. Colour pale brown to yellow, orange and purple. Commonly known as “chicken liver” sponges because of their fleshy texture. These sponges are soft. It is typical of the group in that the internal colour is virtually identical to the external colour (Fig. 184).

#### Unknown lettuce-like green sponge

EF73FB1B-F7F5-5576-9862-523474AB16B2

##### Materials

- **Type status:**Other material. **Taxon:** scientificName: Lettuce-like green sponge; kingdom: Animalia; phylum: Porifera; **Location:** waterBody: Indian Ocean; country: Seychelles; locality: Aldabra N1; minimumDepthInMeters: 120 m; maximumDepthInMeters: 120 m; locationRemarks: First Descent: Seychelles Expedition; **Identification:** identifiedBy: Nico Fassbender, Toufiek Samaai, Paris Stefanoudis; dateIdentified: 2019, 2020; identificationRemarks: identified only from imagery; **Event:** samplingProtocol: Submersible OR Remotely Operated Vehicle OR SCUBA; **Record Level:** basisOfRecord: Human observation

##### Notes

A foliose sponge that resembles lettuce. Maximum recorded size: 28 cm across. Flower-like appearance with irregular edges. Dark green colouration (Fig. 185).

## Figures and Tables

**Figure 2a.**
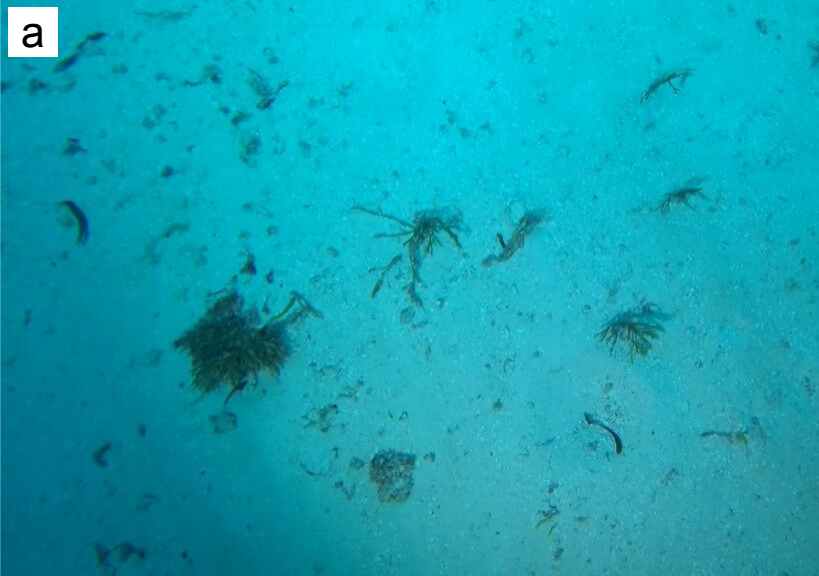
D'Arros N1, 60 m.

**Figure 2b.**
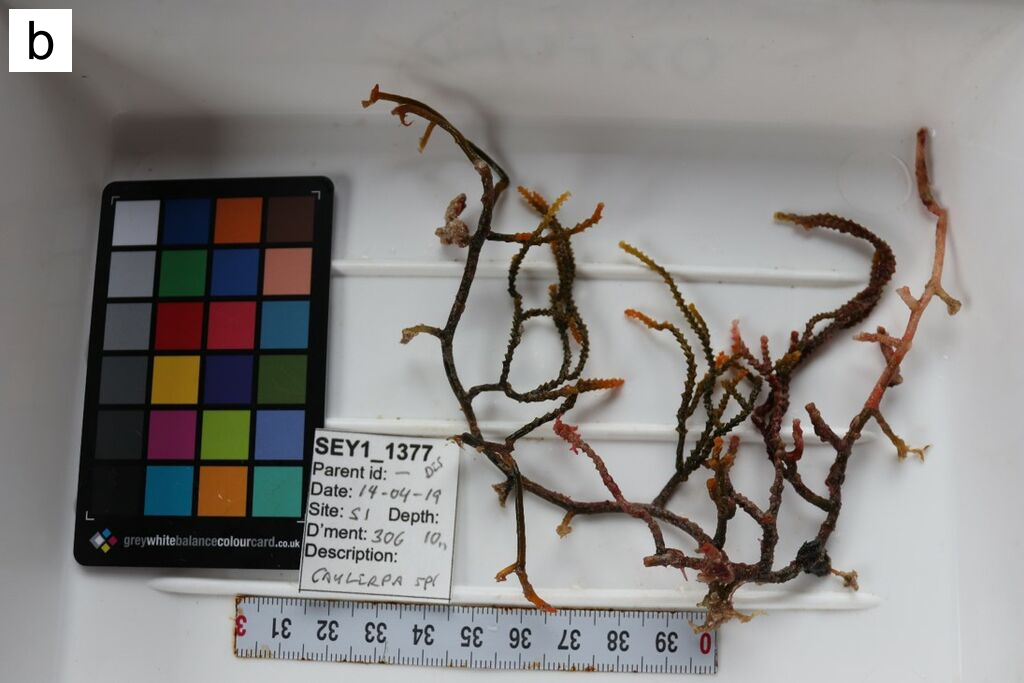
Desroches S1, 10 m, collected specimen (SEY1_1377).

**Figure 5a.**
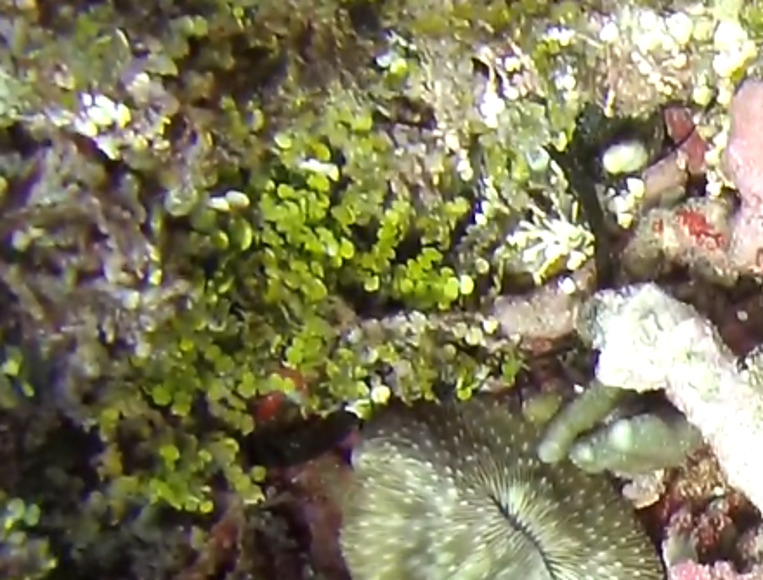
Aldabra N1, 10 m.

**Figure 5b.**
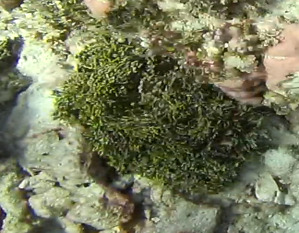
Aldabra N1, 10 m.

**Figure 5c.**
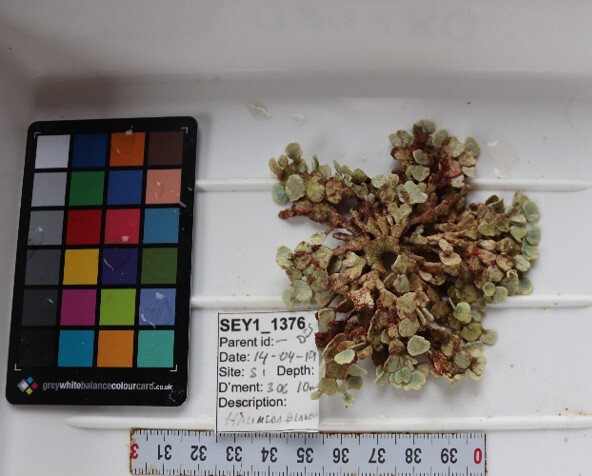
*Halimeda* sp. indet. Desroches S1, 10 m, collected specimen (SEY1_1376).

**Figure 5d.**
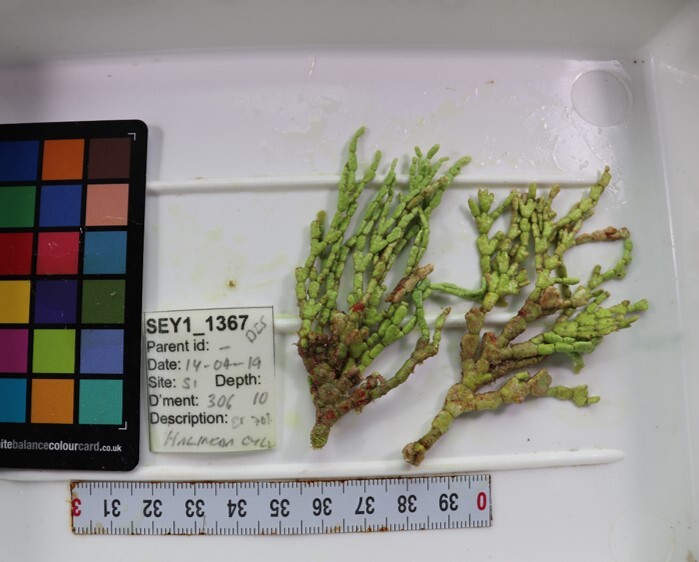
*Halimedacylindracea*. Desroches S1, 10 m, collected specimen (SEY1_1367).

**Figure 6a.**
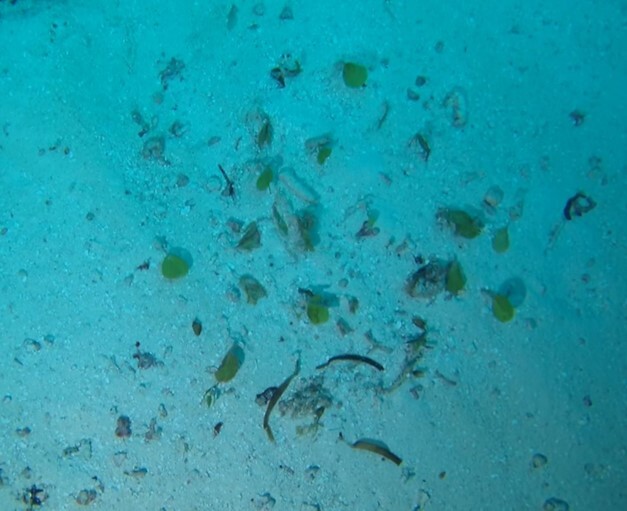
*Udotea* sp. indet. 1. D'Arros N1, 30 m.

**Figure 6b.**
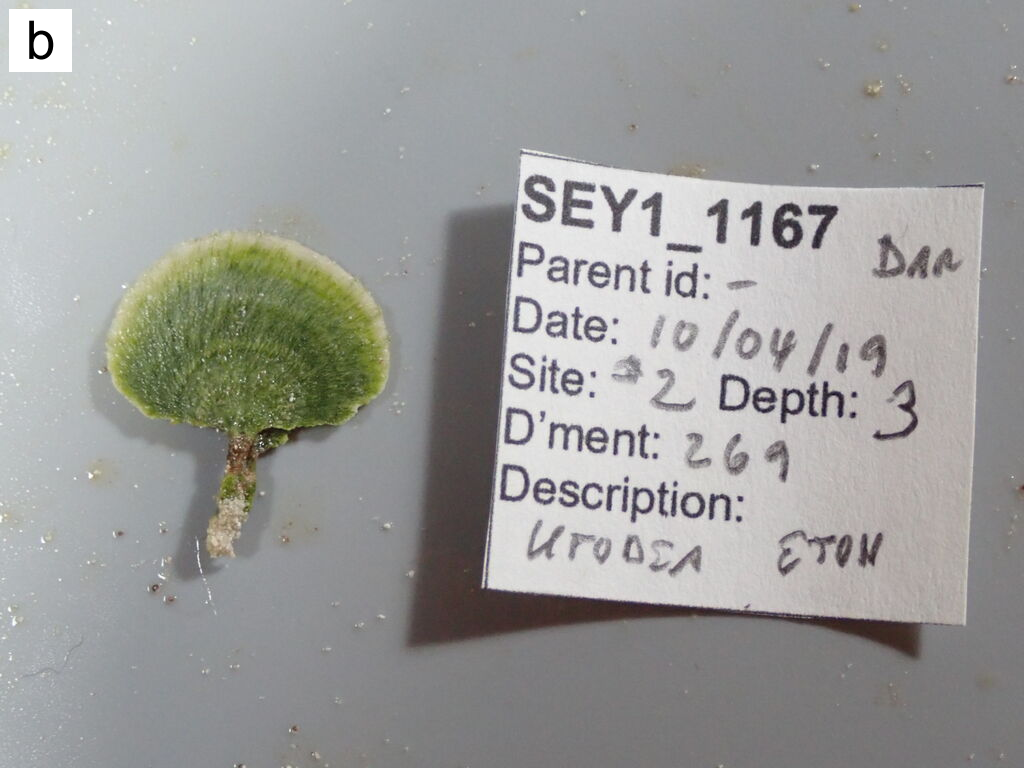
*Udotea* sp. indet. 2. D'Arros N1, 3 m, collected specimen (SEY1_1167).

**Figure 7a.**
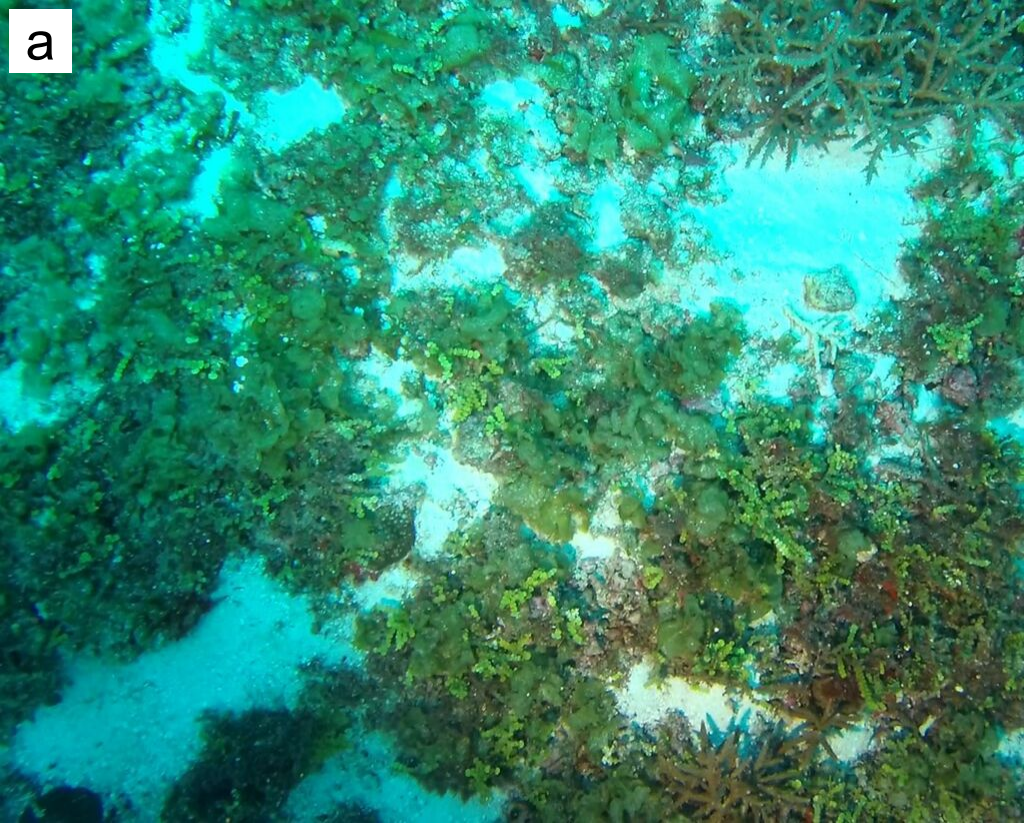
Poivre E1, 30 m.

**Figure 7b.**
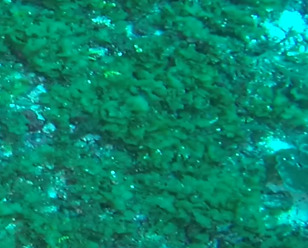
Poivre E1, 30 m.

**Figure 10a.**
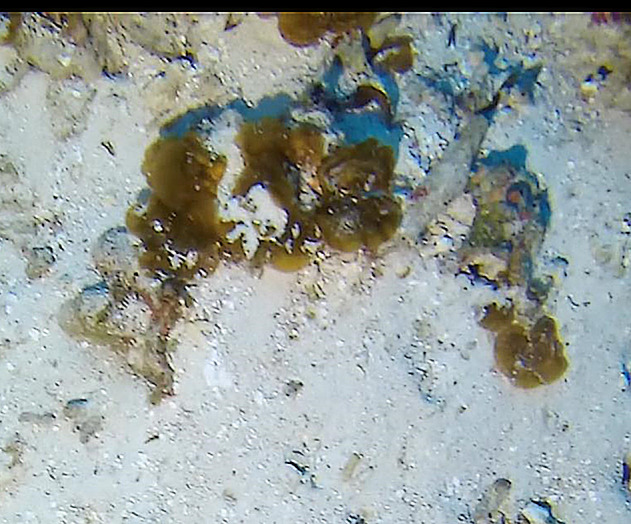
Astove W1, 60 m.

**Figure 10b.**
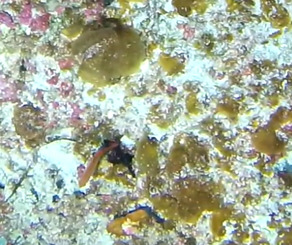
Alphonse N1, 60 m.

**Figure 11a.**
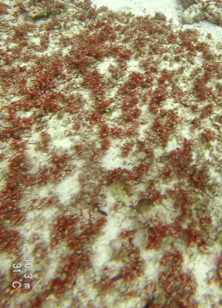
Desroches S1, 10 m.

**Figure 11b.**
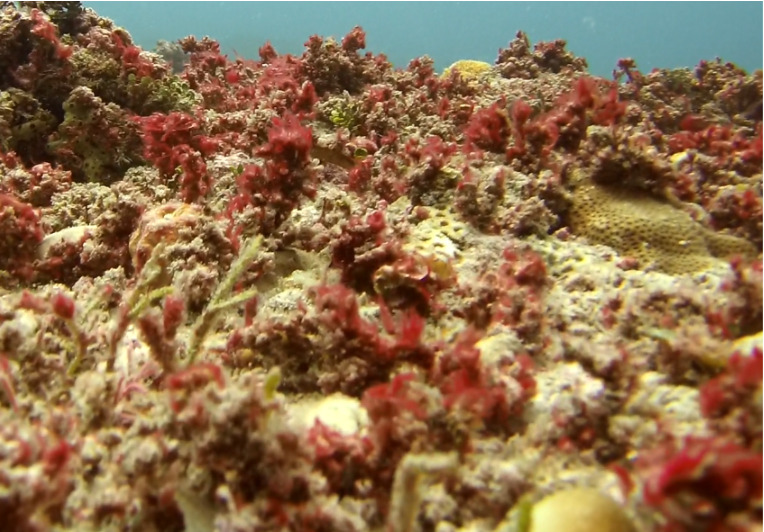
Desroches S1, 10 m.

**Figure 12a.**
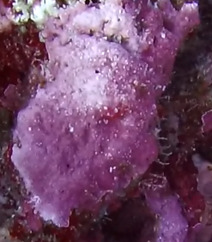
Astove W1, 10 m.

**Figure 12b.**
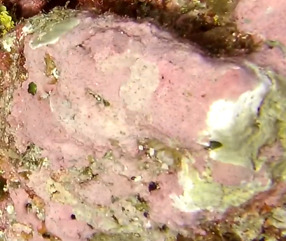
Astove W1, 10 m.

**Figure 20a.**
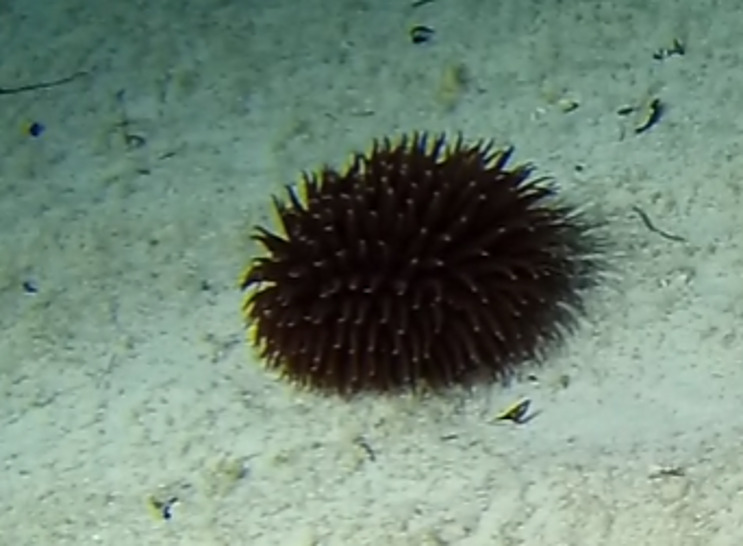
Aldabra W1, 250 m.

**Figure 20b.**
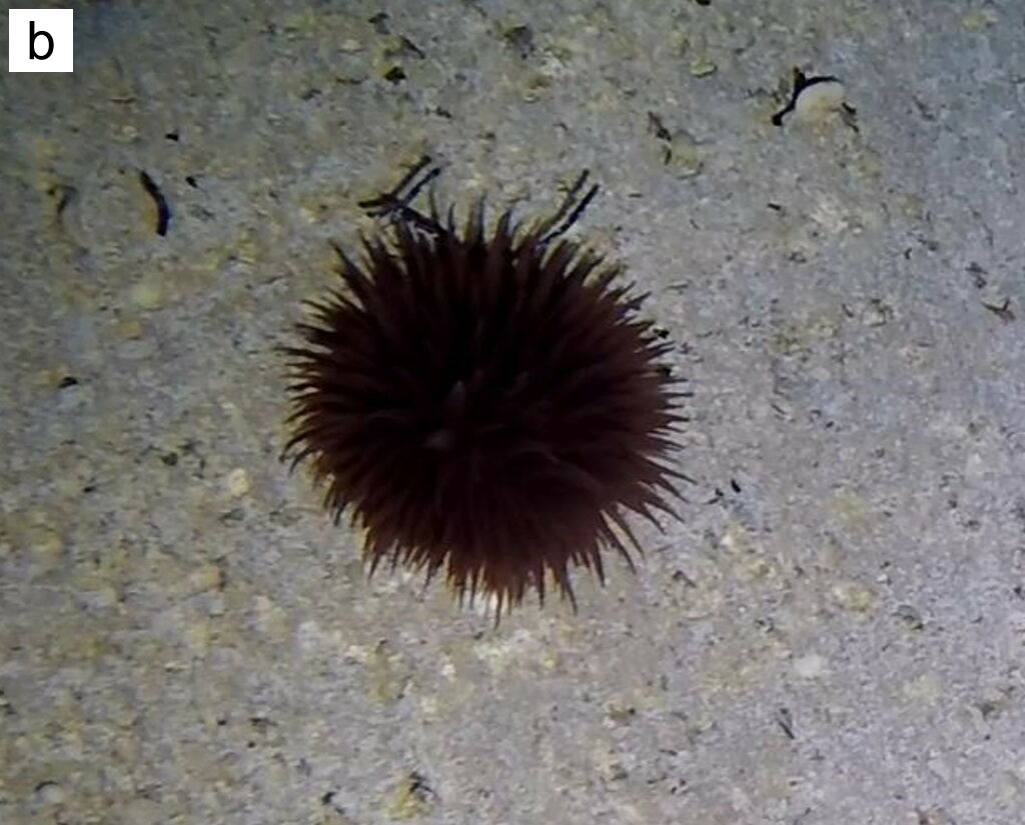
Astove W1, 250 m.

**Figure 21a.**
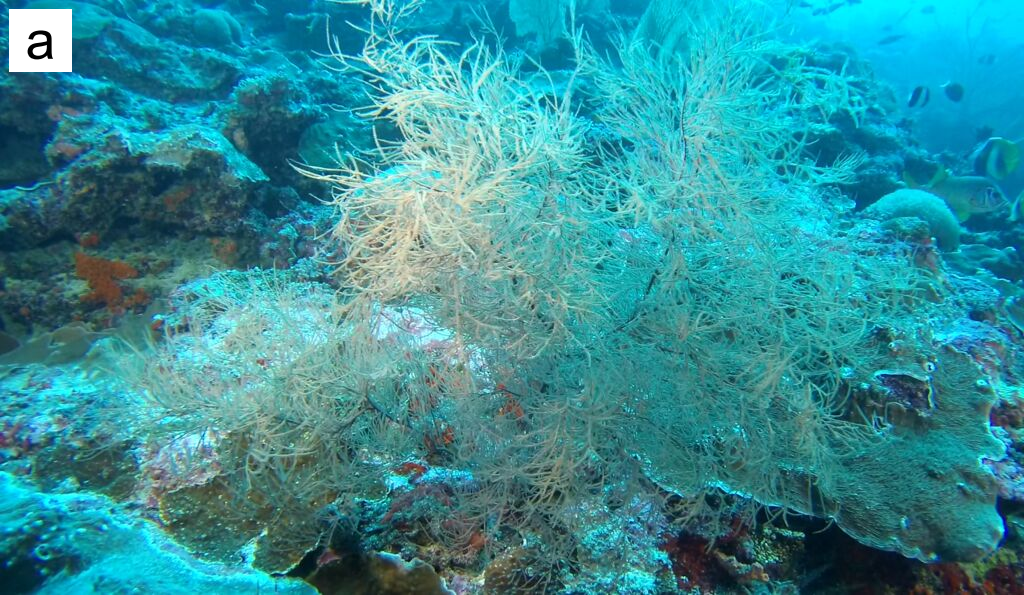
Aldabra N1, 30 m.

**Figure 21b.**
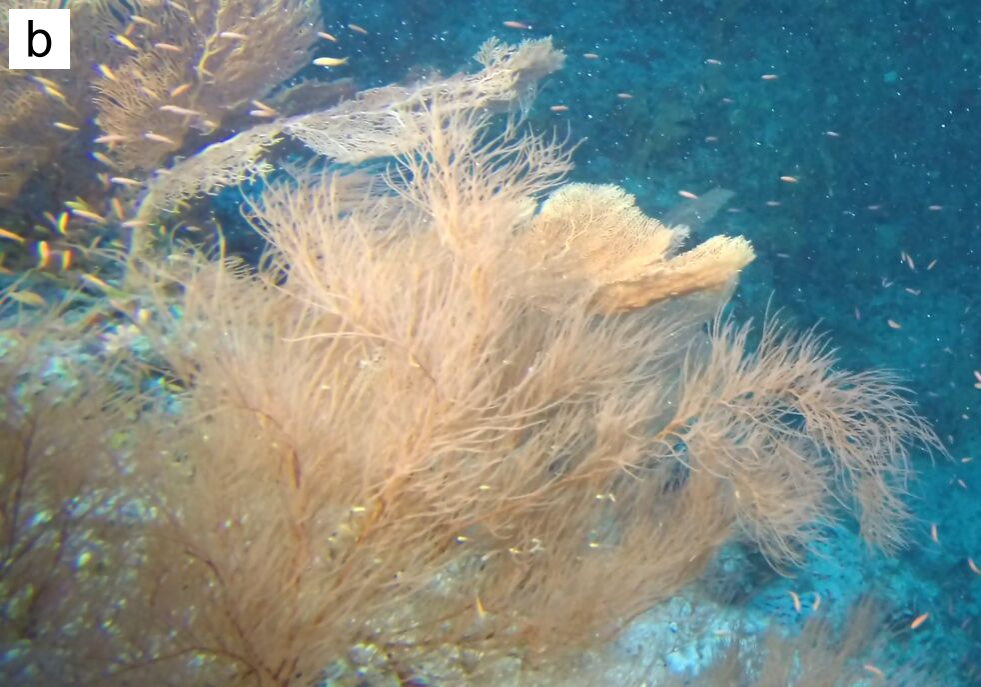
Aldabra N1, 60 m.

**Figure 22a.**
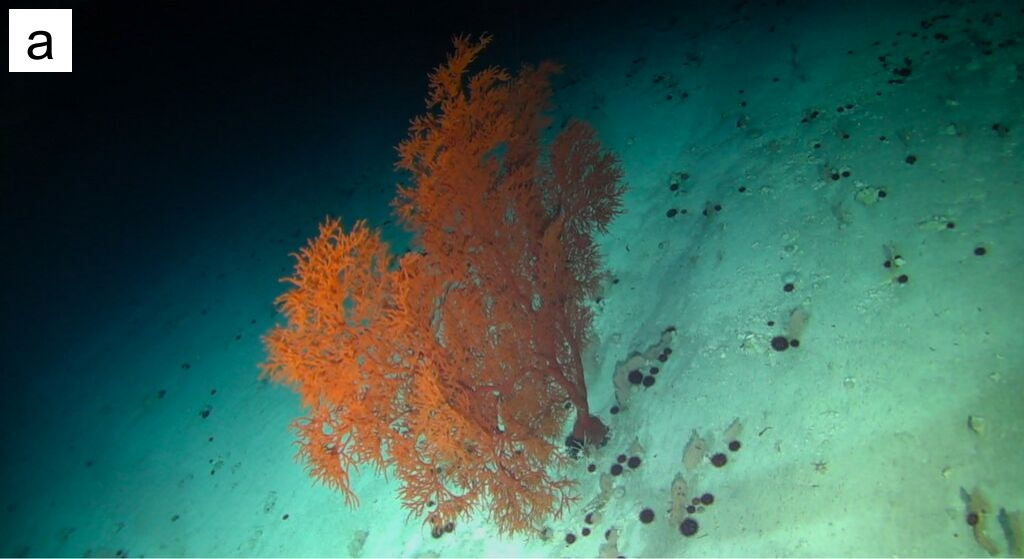
Alphonse N1, 250 m.

**Figure 22b.**
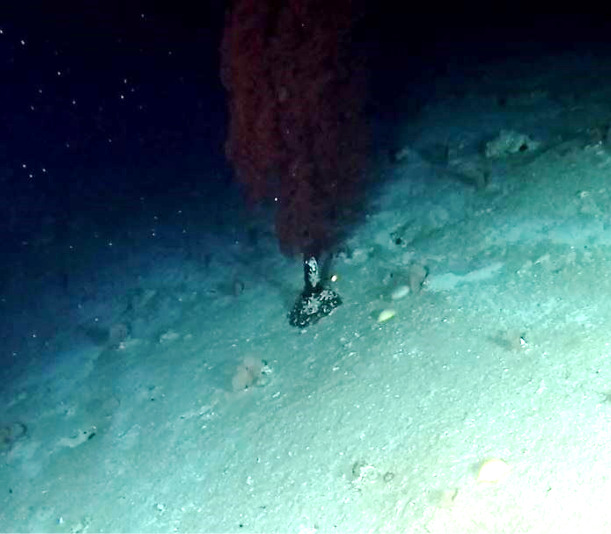
Alphonse N1, 250 m.

**Figure 23a.**
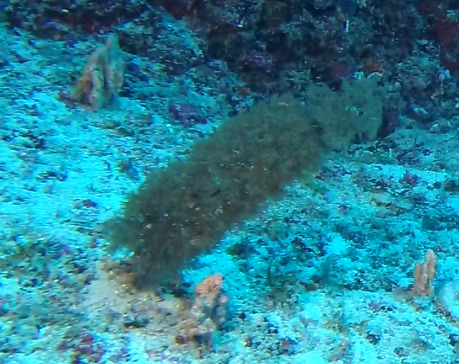
Desroches S1, 30 m.

**Figure 23b.**
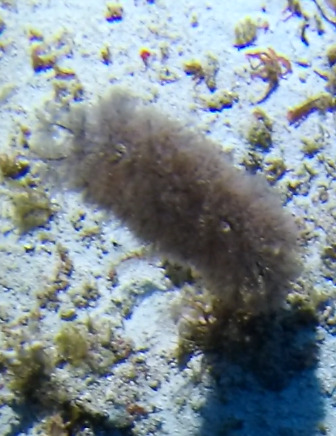
D'Arros N1, 60 m.

**Figure 23c.**
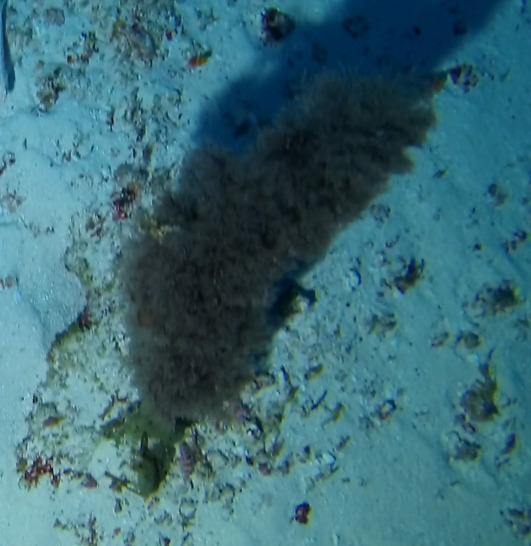
Desroches S1, 60 m.

**Figure 24a.**
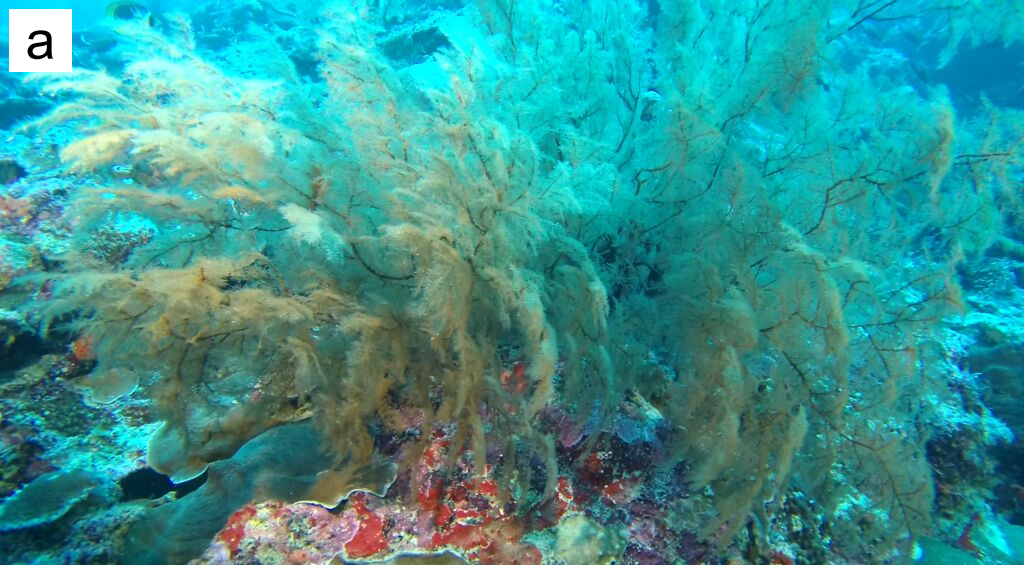
Aldabra N1, 30 m.

**Figure 24b.**
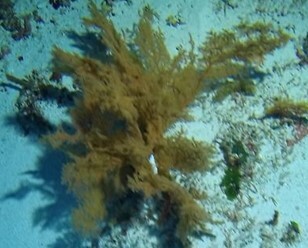
Desroches S1, 60 m.

**Figure 24c.**
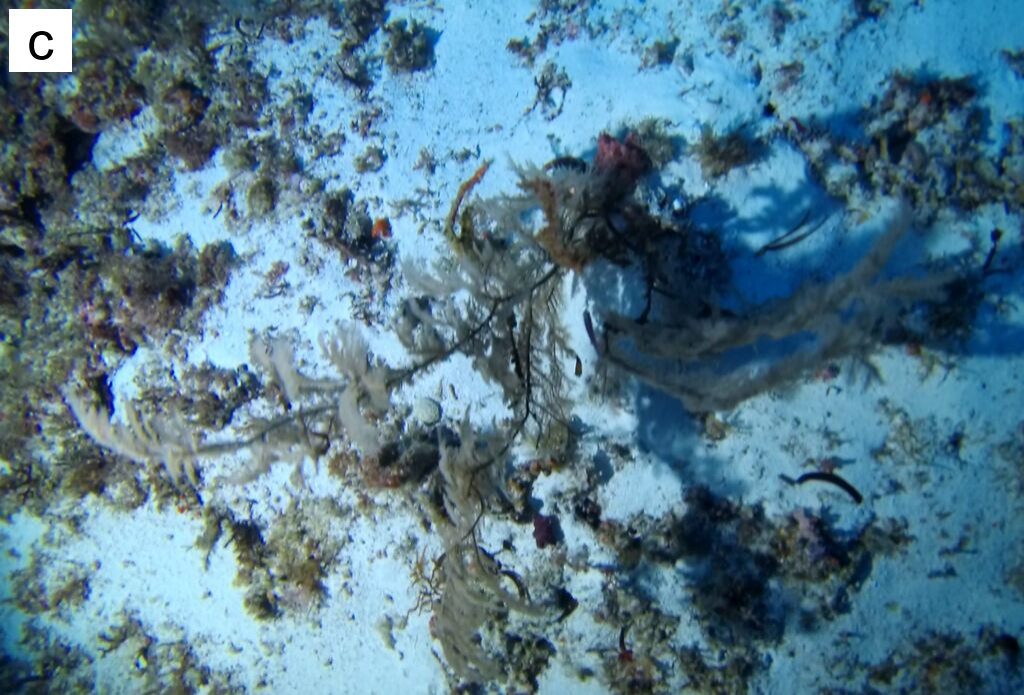
D'Arros N1, 60 m.

**Figure 25a.**
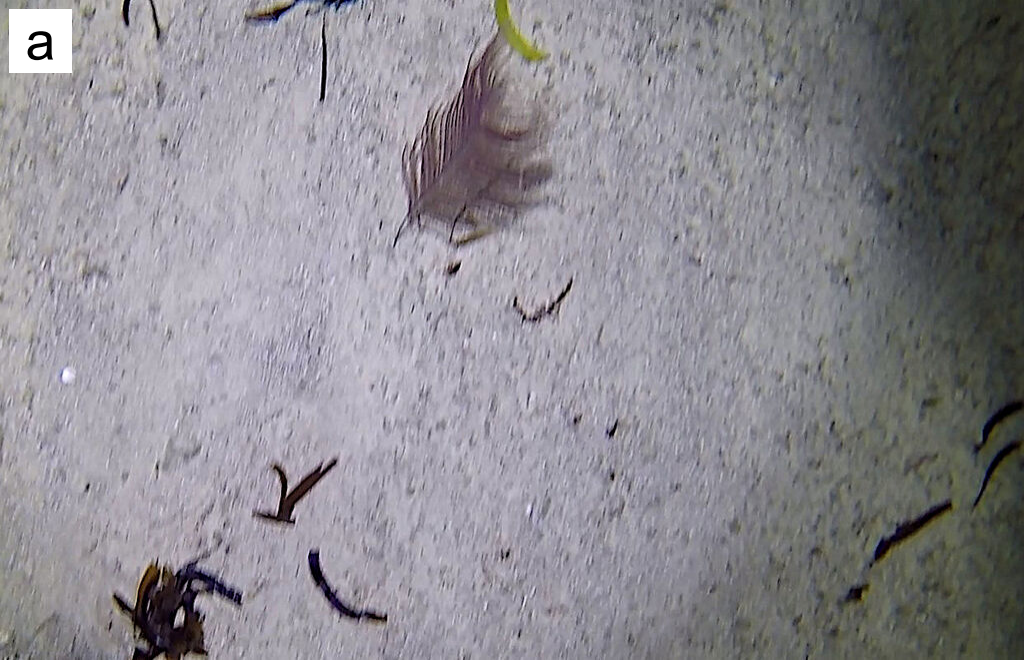
D'Arros N1, 350 m.

**Figure 25b.**
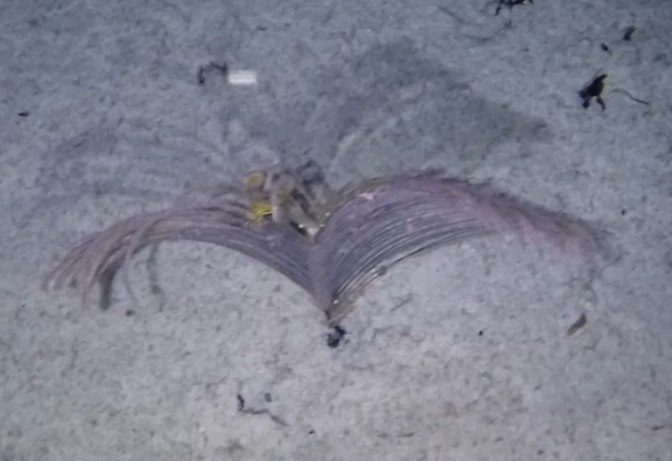
D'Arros N1, 350 m.

**Figure 27a.**
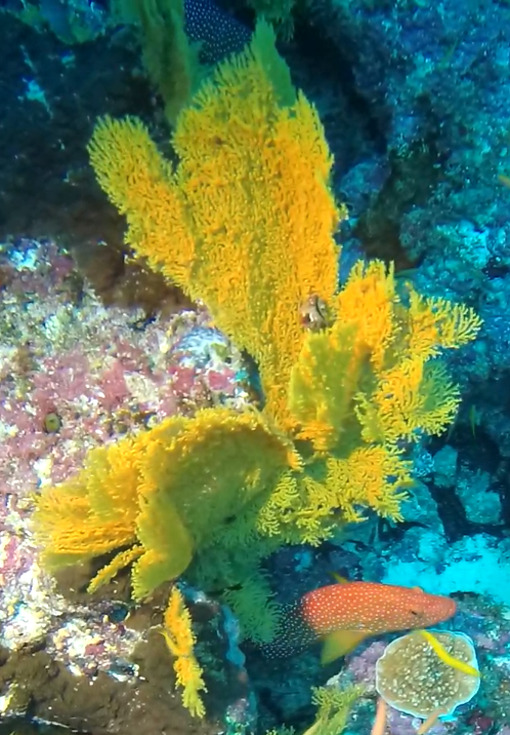
Alphonse N1, 60 m.

**Figure 27b.**
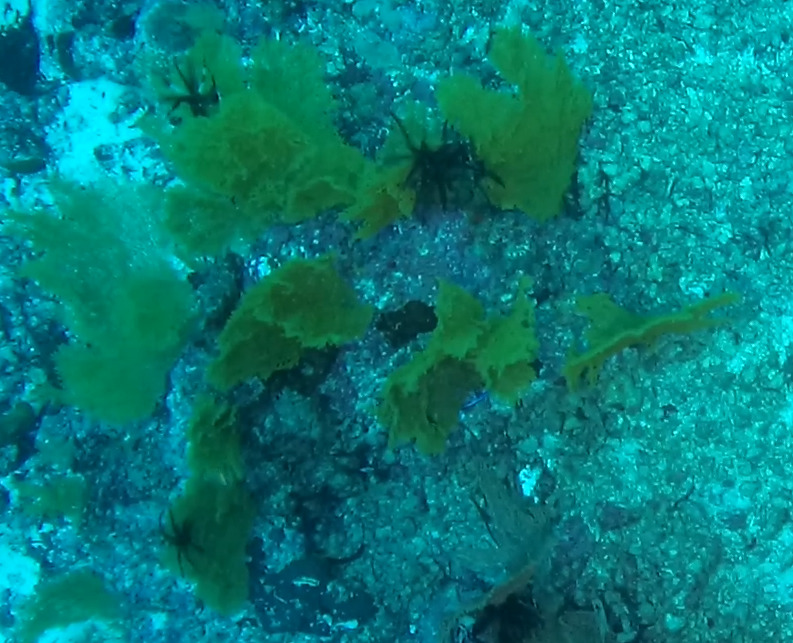
Alphonse N1, 60 m.

**Figure 28a.**
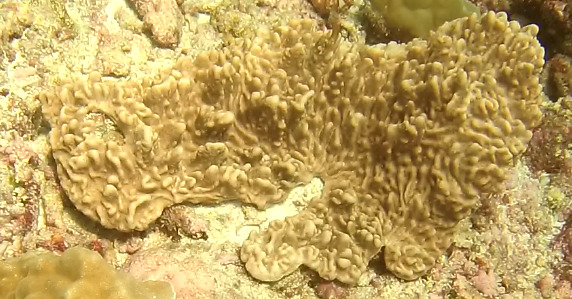
Poivre E1, 10 m.

**Figure 28b.**
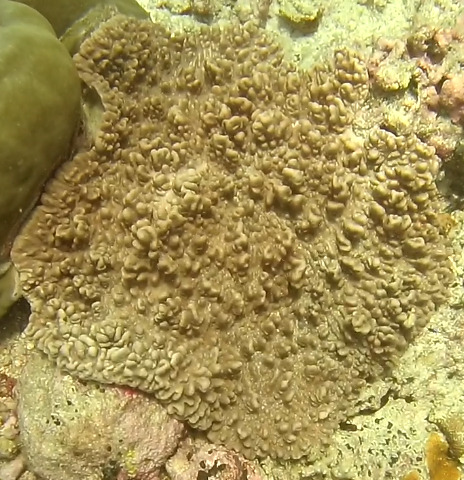
Poivre E1, 10 m.

**Figure 28c.**
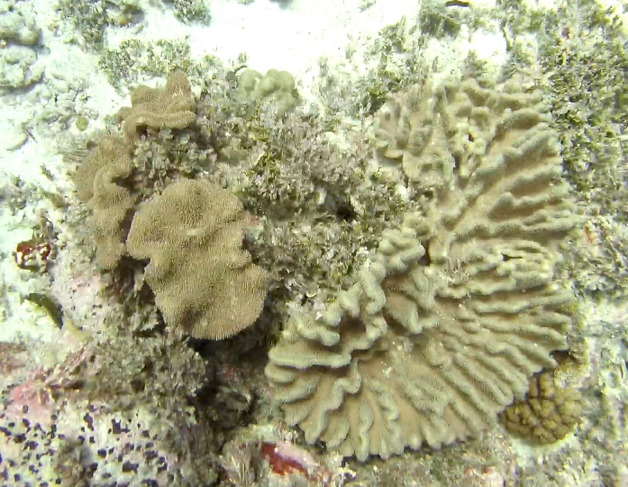
Aldabra N1, 10 m.

**Figure 29a.**
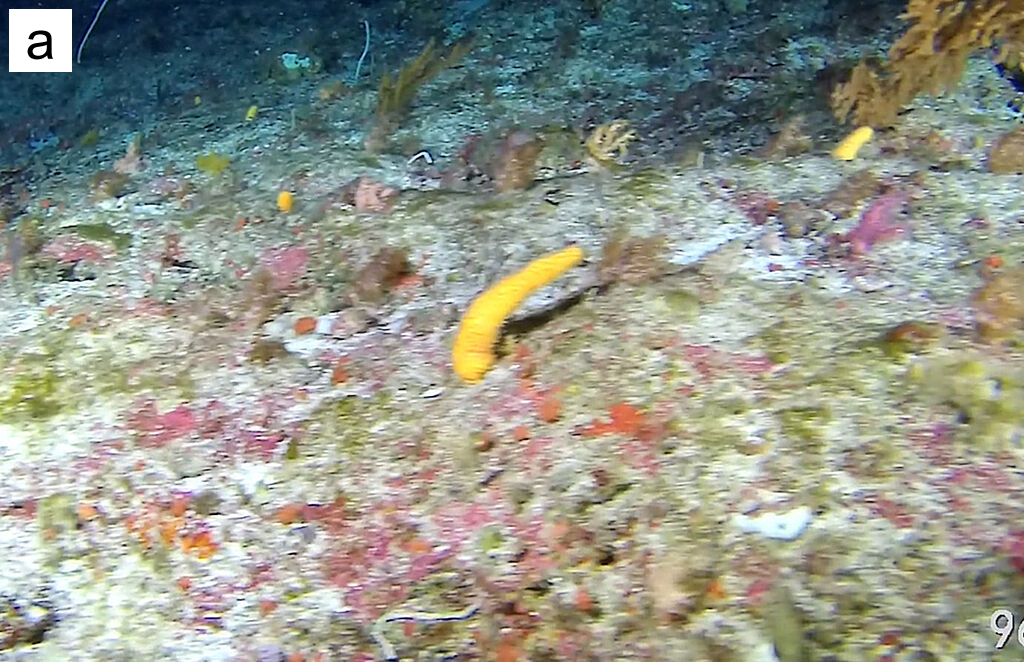
Alphonse N1, 97 m.

**Figure 29b.**
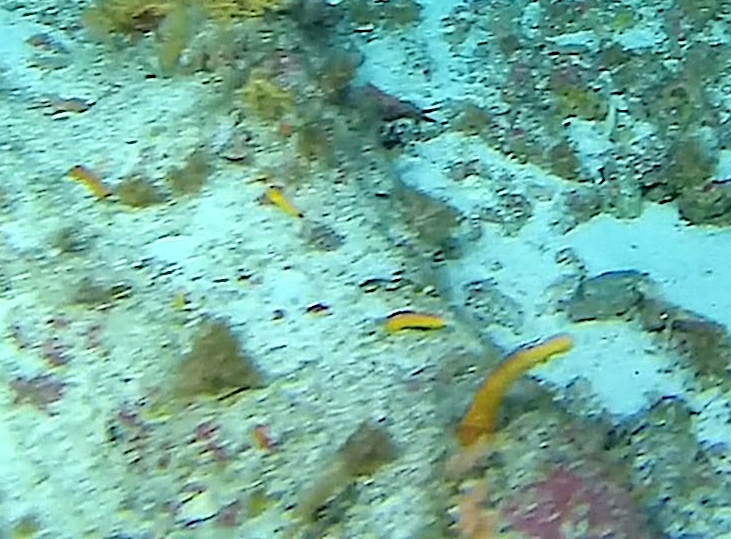
Alphonse N1, 97 m.

**Figure 30a.**
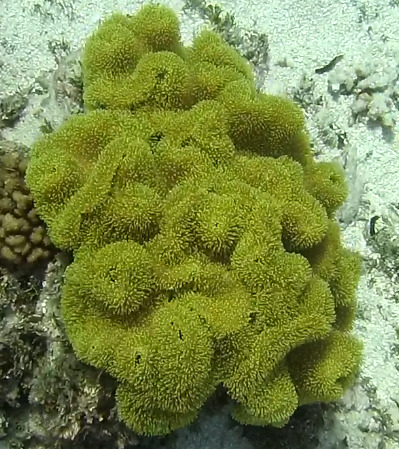
Aldabra N1, 10 m.

**Figure 30b.**
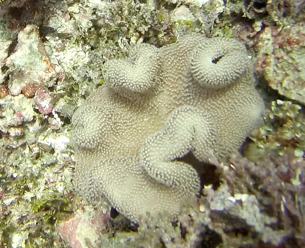
Aldabra N1, 10 m.

**Figure 30c.**
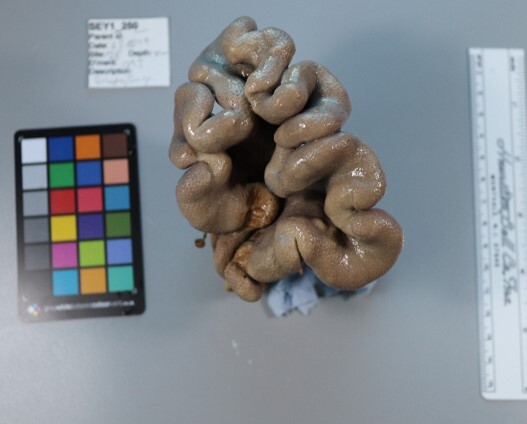
Aldabra N1, 10 m, collected specimen (SEY1_250)

**Figure 30d.**
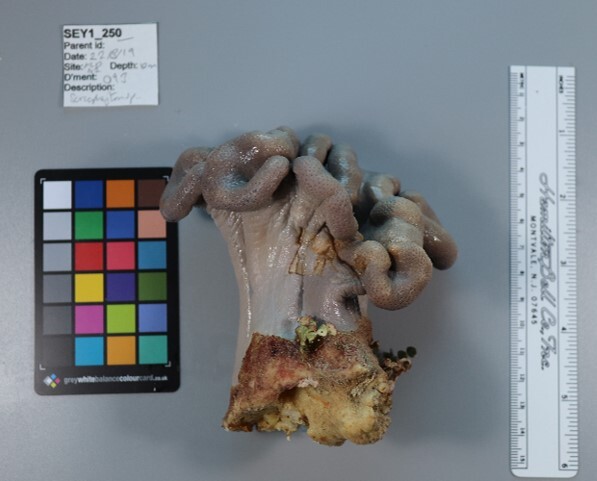
Aldabra N1, 10 m, collected specimen (SEY1_250)

**Figure 31a.**
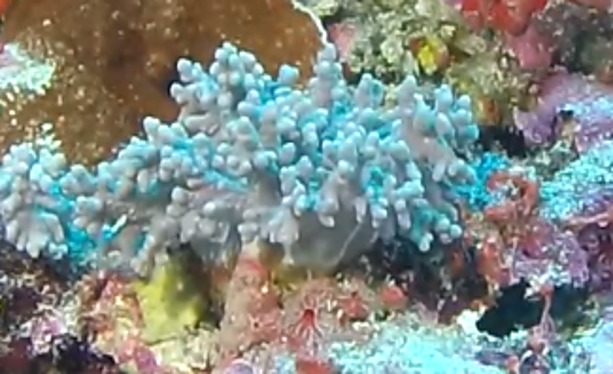
Aldabra N1, 30 m.

**Figure 31b.**
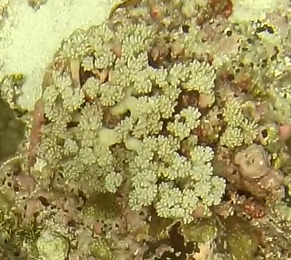
Poivre E1, 10 m.

**Figure 31c.**
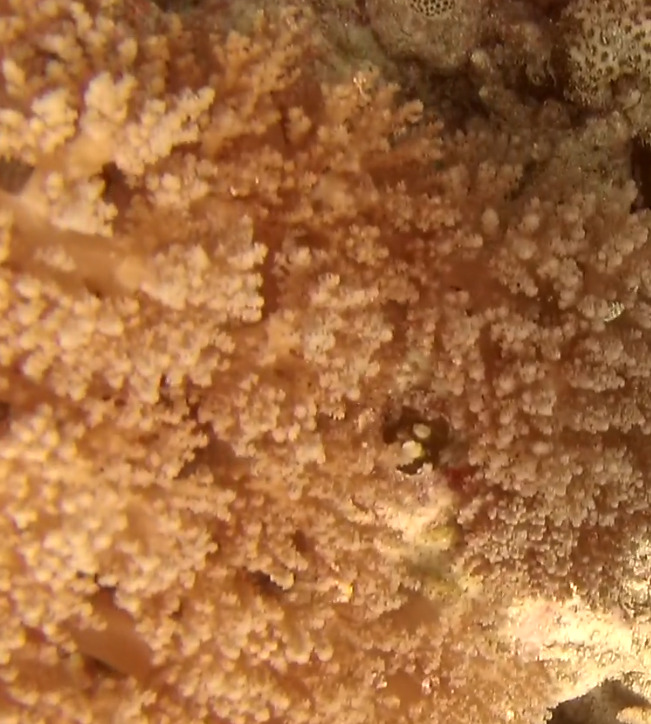
Aldabra W1, 10 m.

**Figure 32a.**
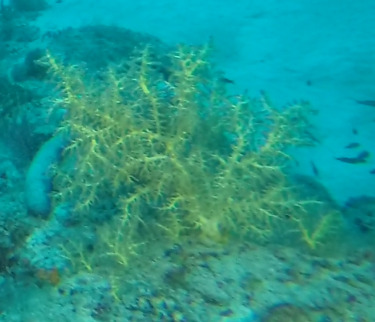
Aldabra W1, 60 m.

**Figure 32b.**
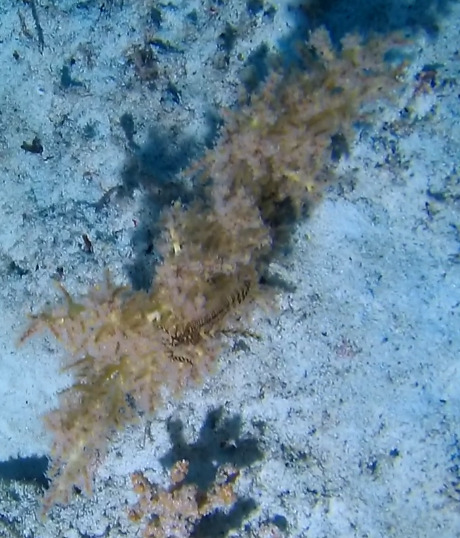
Aldabra W1, 60 m.

**Figure 32c.**
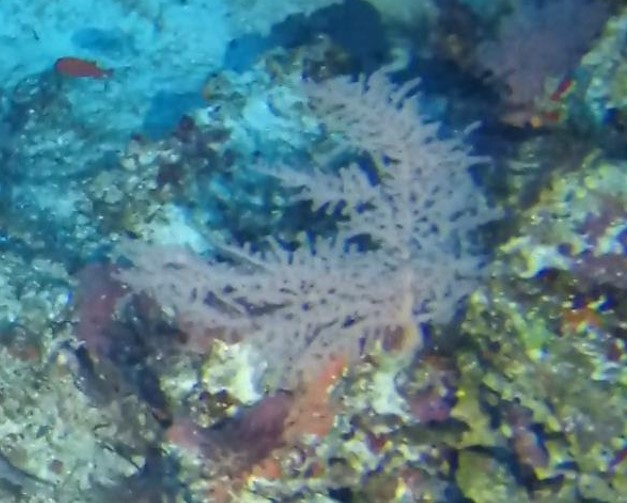
Astove W1, 60 m.

**Figure 34a.**
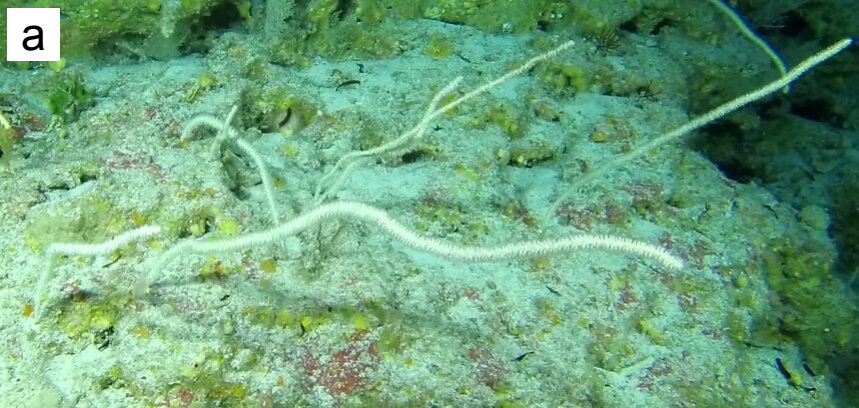
Aldabra N1, 120 m.

**Figure 34b.**
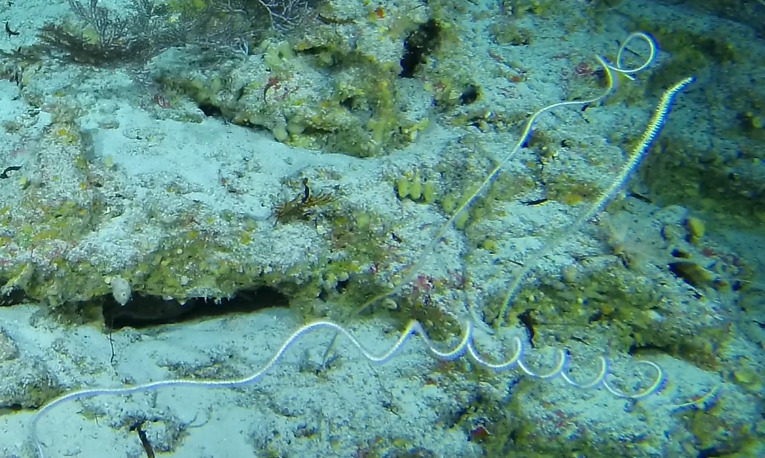
Aldabra N1, 120 m.

**Figure 34c.**
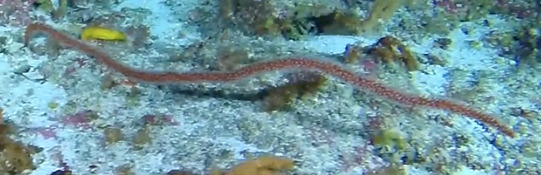
Alphonse N1, 120 m.

**Figure 36a.**
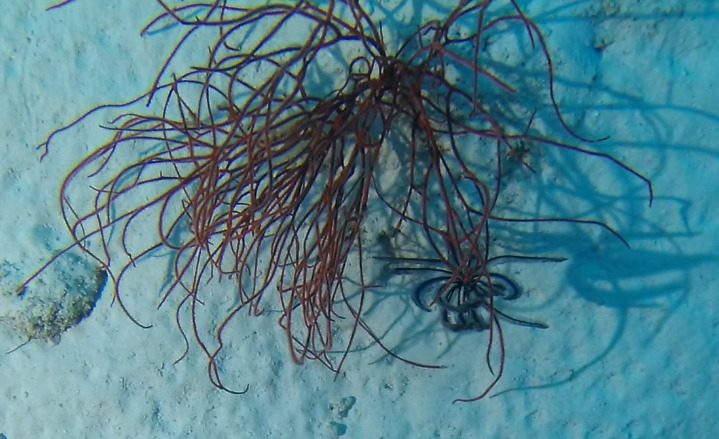
Aldabra W1, 30 m.

**Figure 36b.**
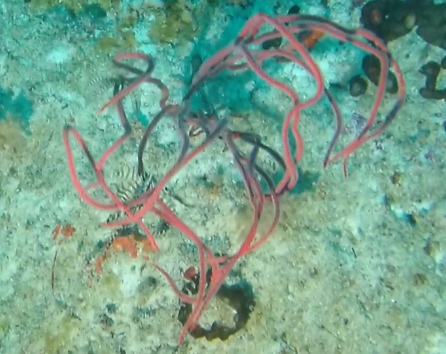
Aldabra W1, 60 m.

**Figure 37a.**
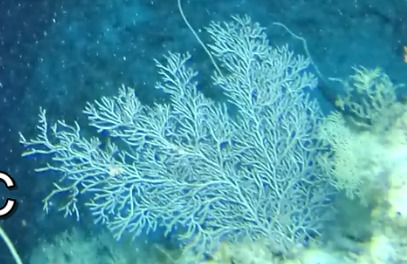
Aldabra N1, 100 m.

**Figure 37b.**
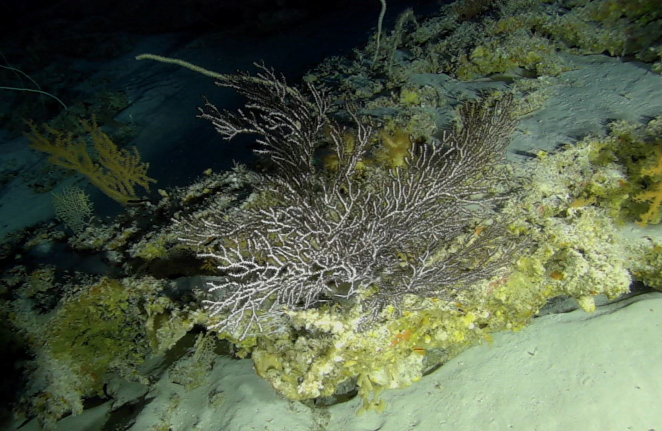
Aldabra N1, 100 m.

**Figure 38a.**
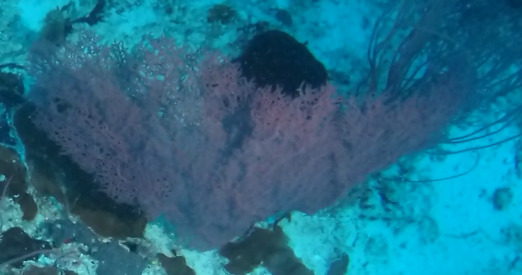
Aldabra N1, 30 m.

**Figure 38b.**
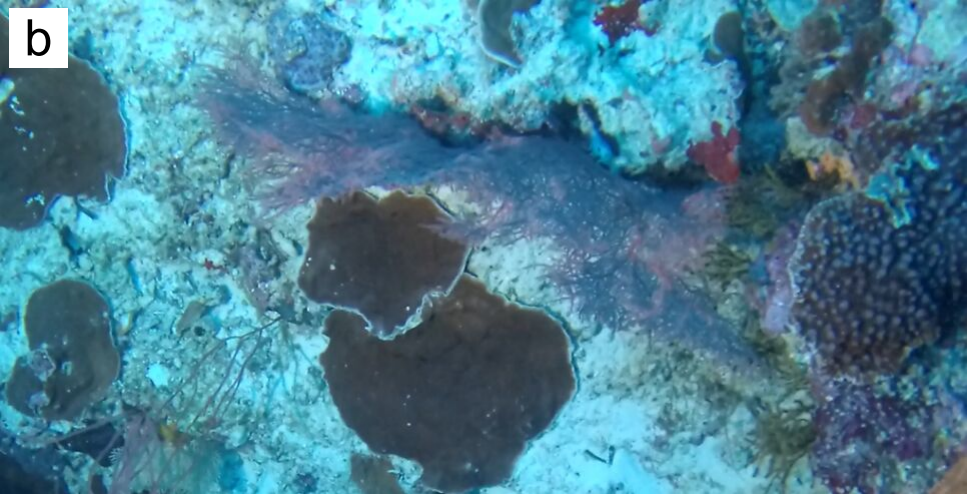
Aldabra N1, 30 m.

**Figure 40a.**
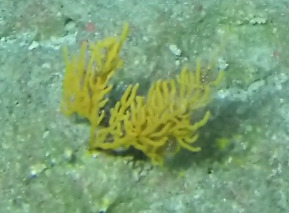
D'Arros N1, 120 m.

**Figure 40b.**
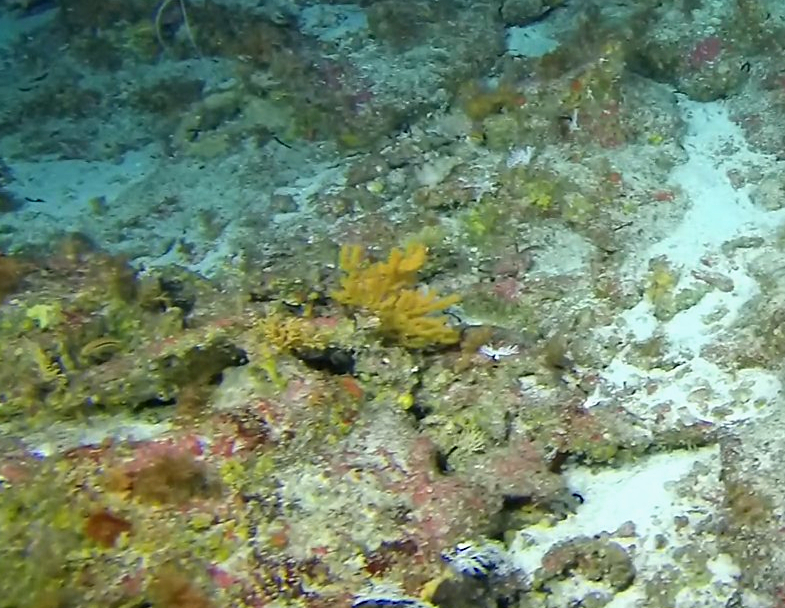
Alphonse N1, 103 m.

**Figure 41a.**
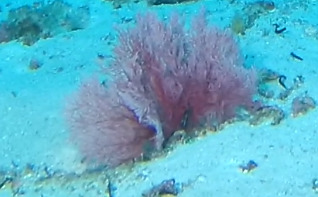
Poivre E1, 60 m.

**Figure 41b.**
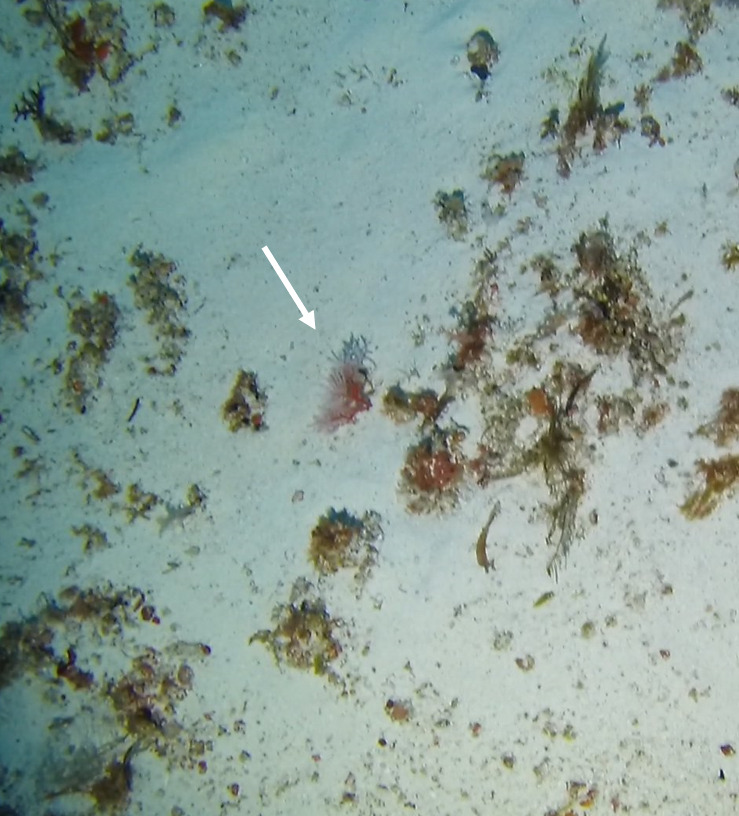
D'Arros N1, 60 m.

**Figure 41c.**
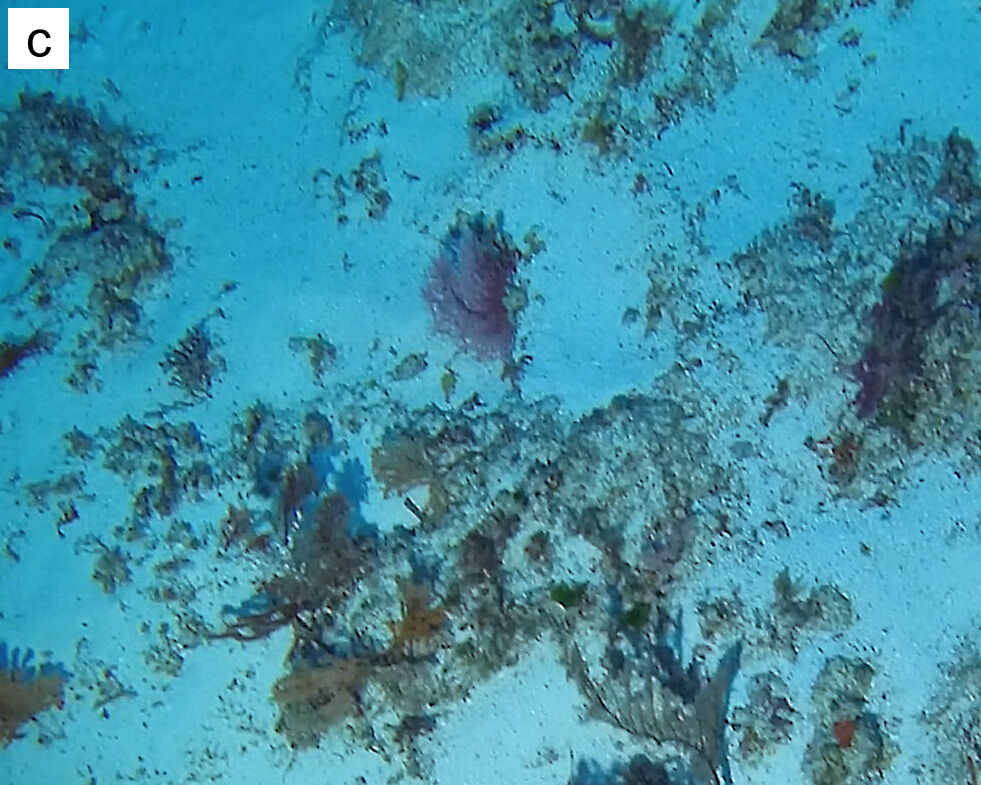
Desroches S1, 60 m.

**Figure 42a.**
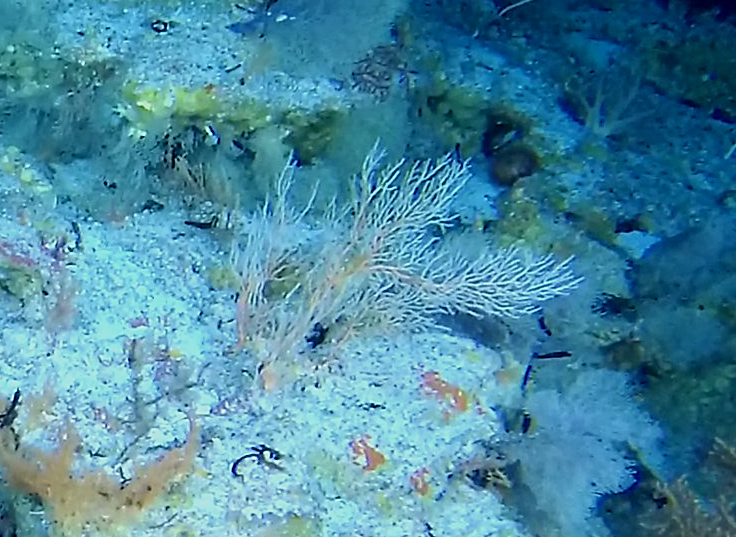
Aldabra N1, 120 m.

**Figure 42b.**
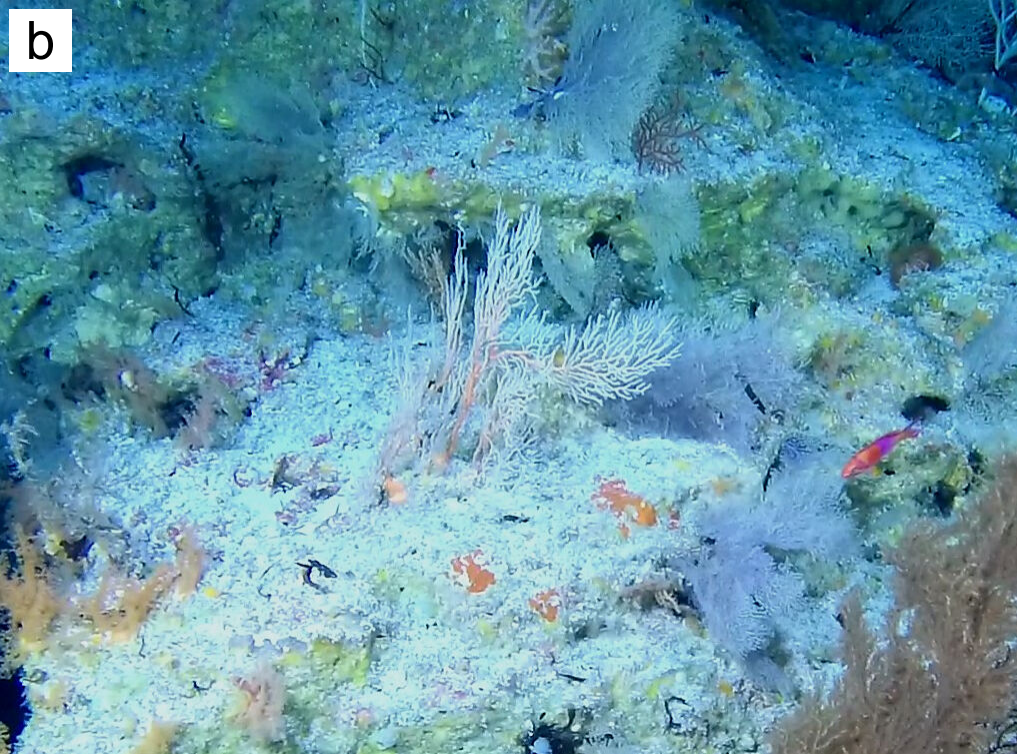
Aldabra N1, 120 m.

**Figure 43a.**
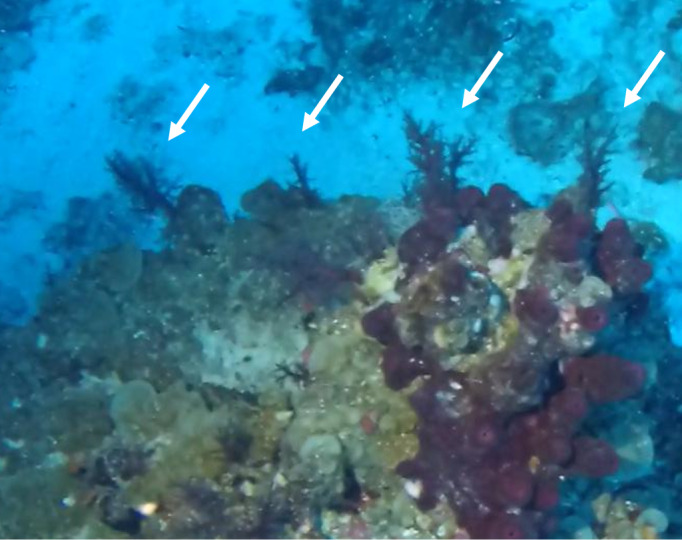
Astove W1, 60 m.

**Figure 43b.**
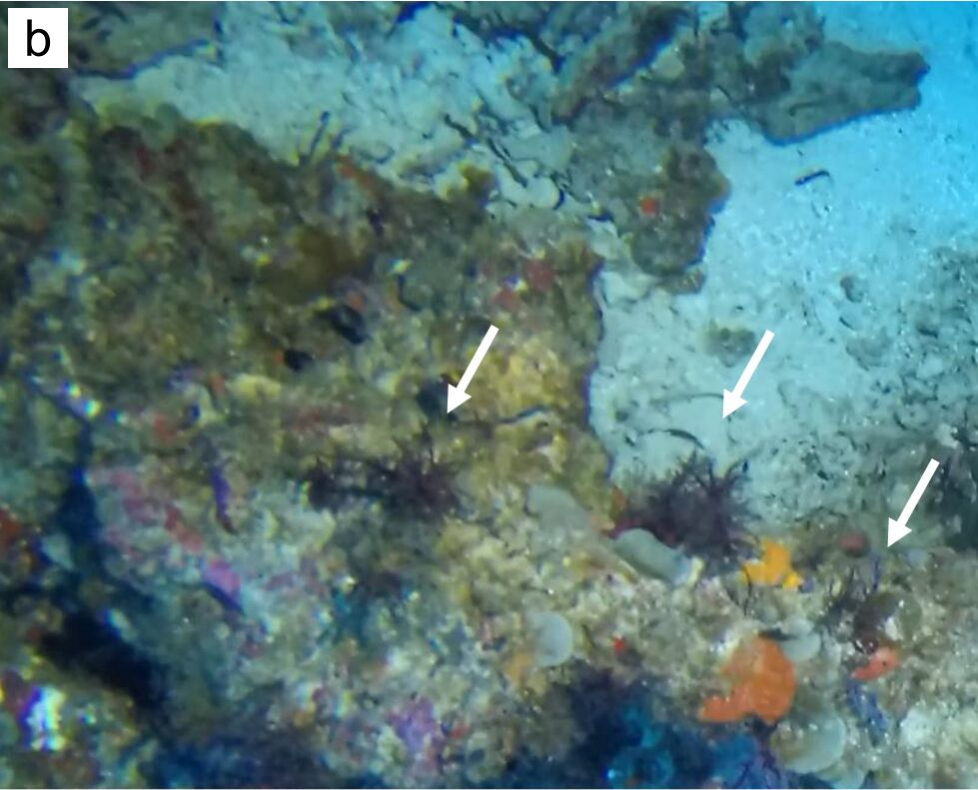
Astove W1, 60 m.

**Figure 44a.**
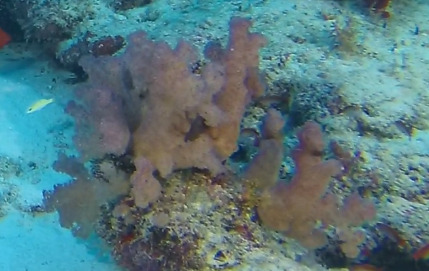
Aldabra W1, 30 m.

**Figure 44b.**
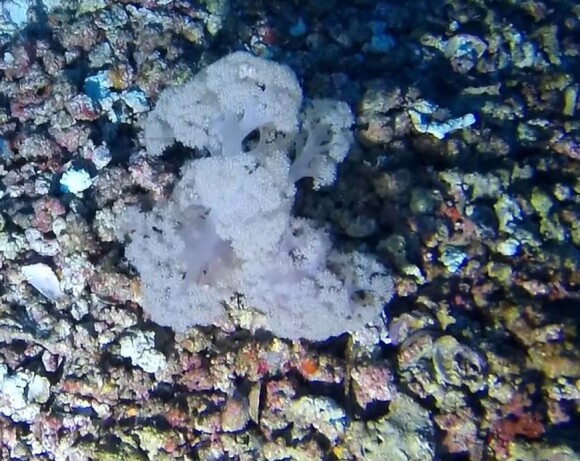
D'Arros N1, 60 m.

**Figure 44c.**
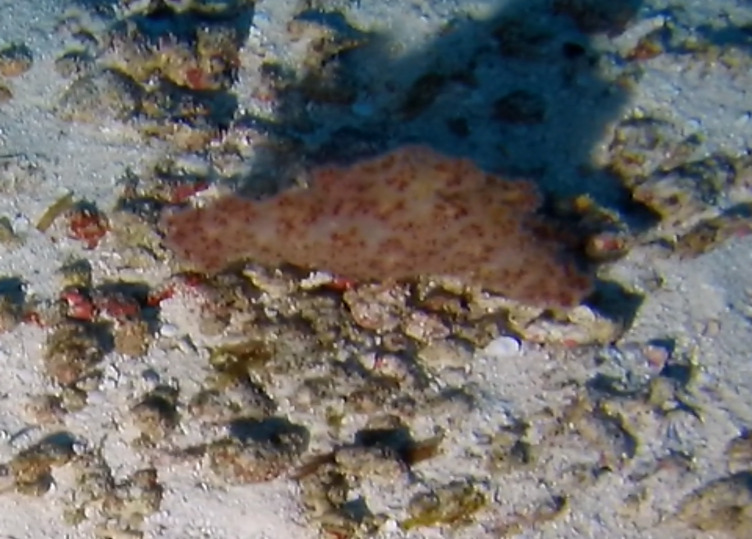
D'Arros N1, 60 m.

**Figure 45a.**
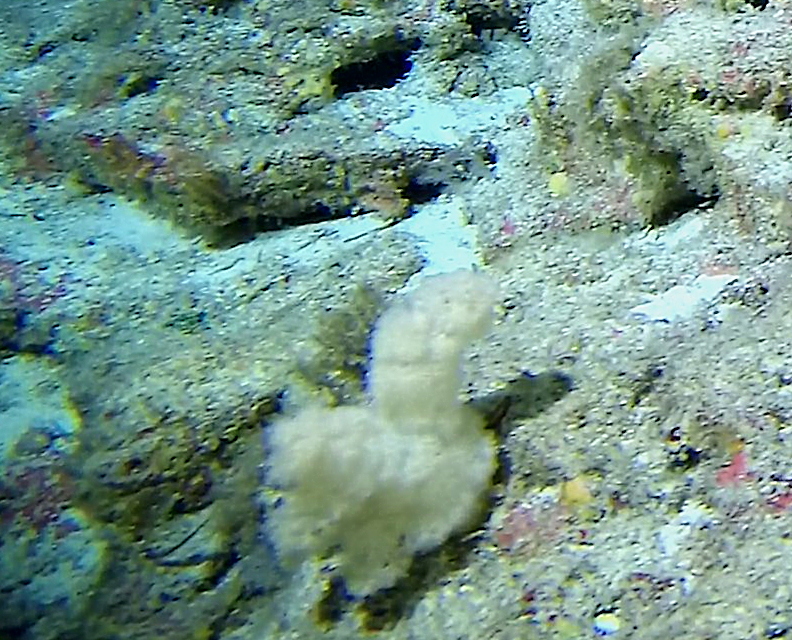
Desroches S1, 120 m.

**Figure 45b.**
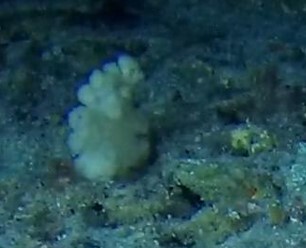
Desroches S1, 120 m.

**Figure 46a.**
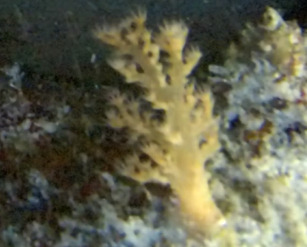
Aldabra N1, 160 m.

**Figure 46b.**
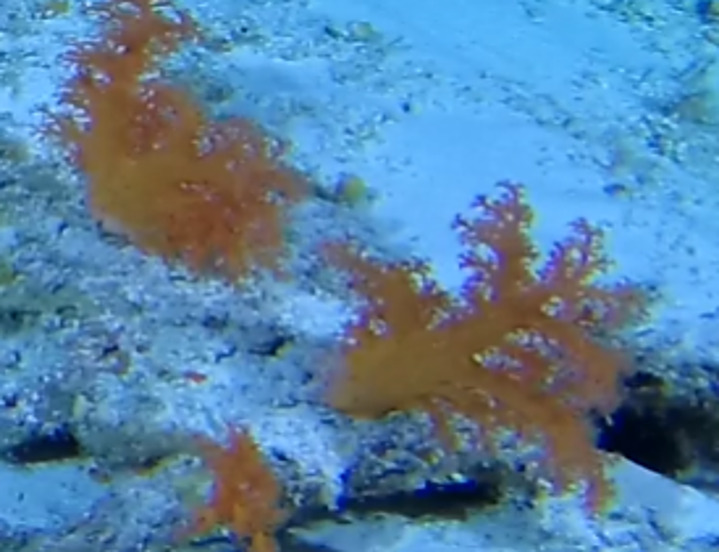
Aldabra W1, 120 m.

**Figure 47a.**
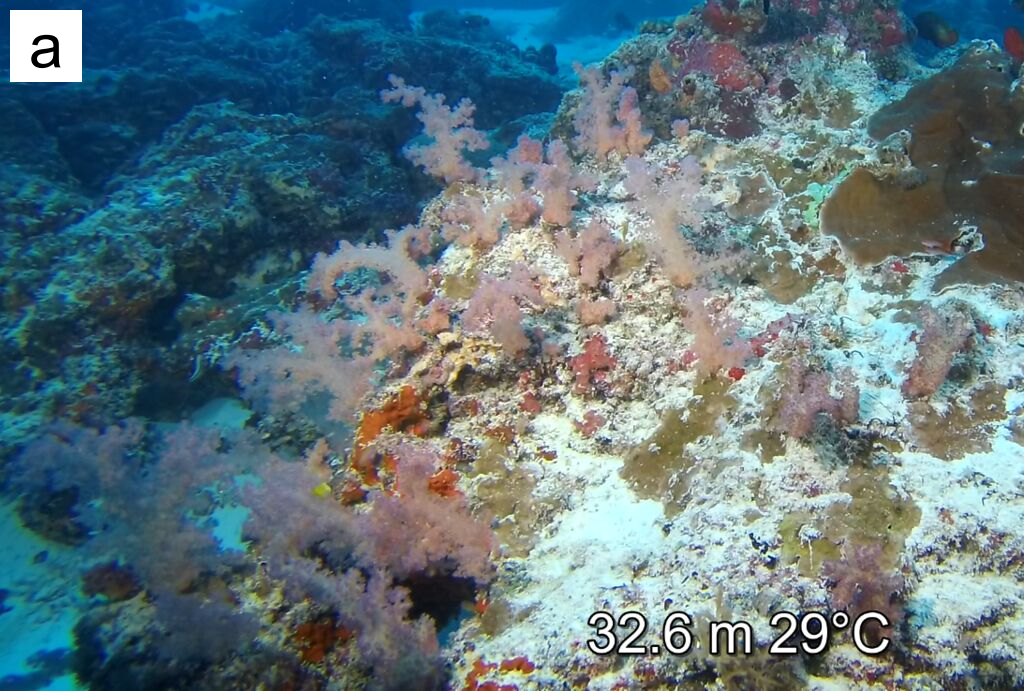
Aldabra W1, 32 m.

**Figure 47b.**
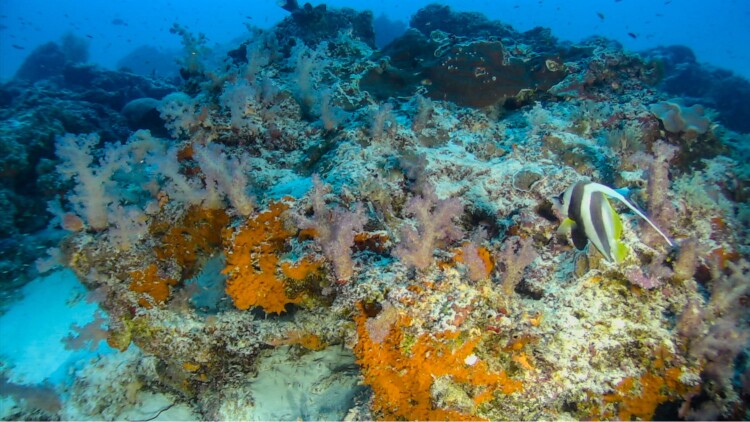
Aldabra W1, 30 m.

**Figure 47c.**
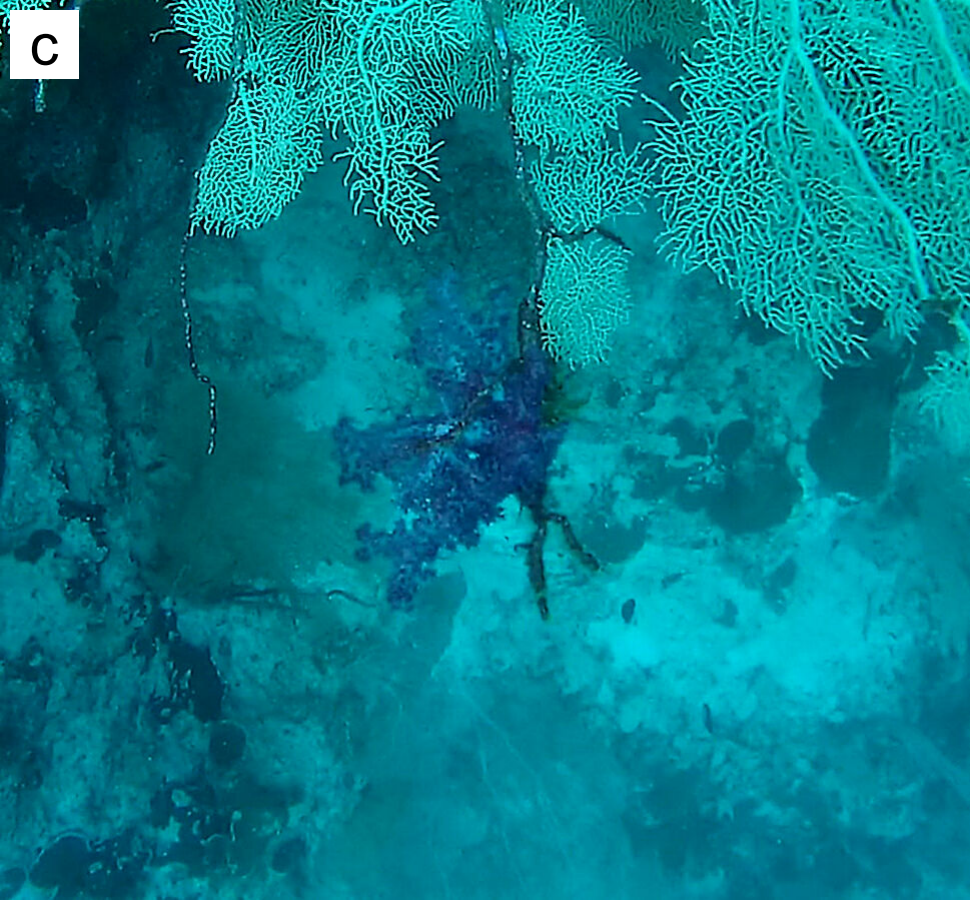
Aldabra N1, 30 m.

**Figure 49a.**
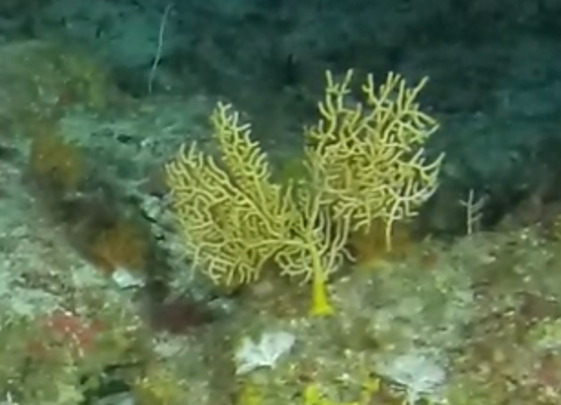
Alphonse N1, 104 m.

**Figure 49b.**
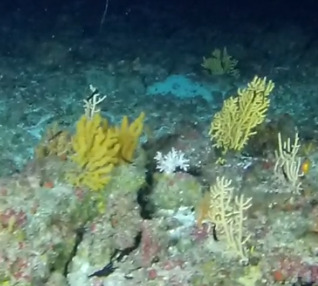
Alphonse N1, 97 m.

**Figure 49c.**
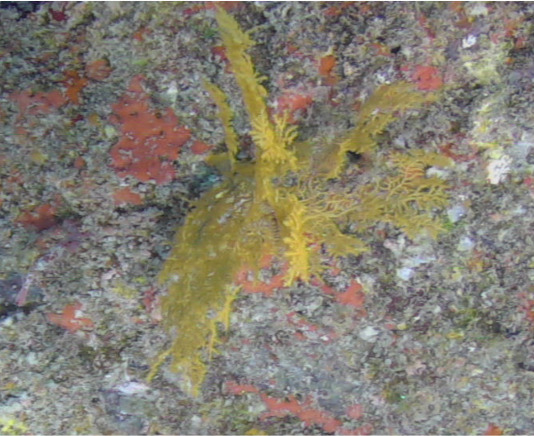
Aldabra N1, 91 m.

**Figure 49d.**
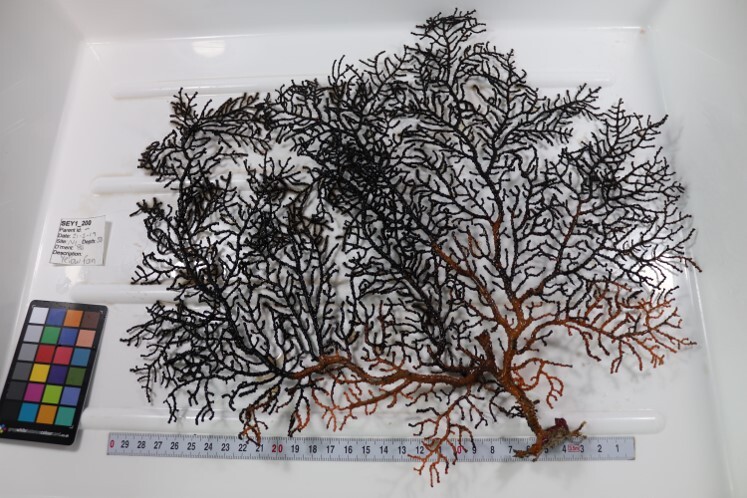
Aldabra N1, 91 m, collected specimen (SEY1_200) corresponding to the in-situ colony of Fig. 61c.

**Figure 52a.**
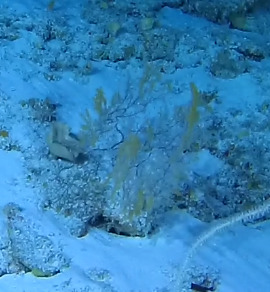
Aldabra W1, 120 m.

**Figure 52b.**
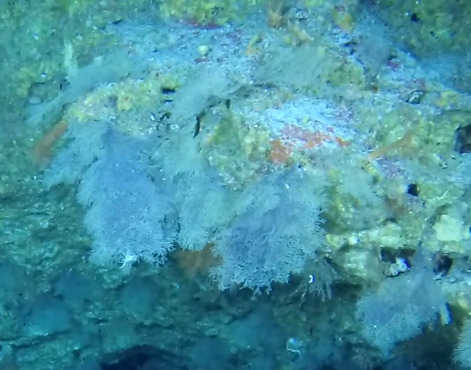
Aldabra N1, 140 m.

**Figure 52c.**
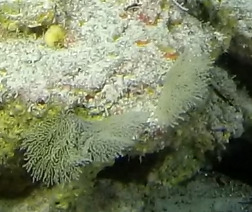
Aldabra N1, 148 m.

**Figure 53a.**
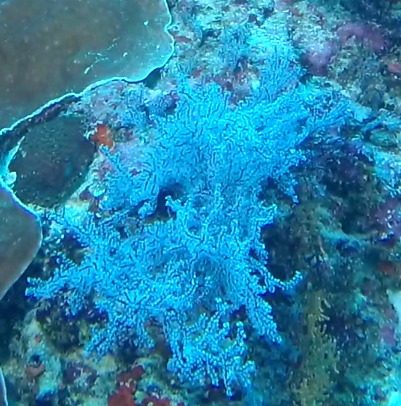
Aldabra N1, 30 m.

**Figure 53b.**
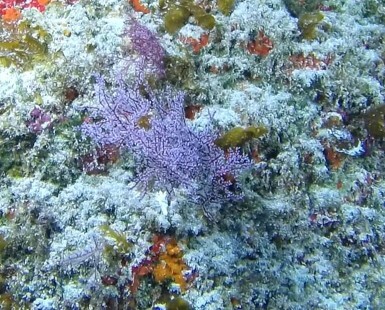
Astove W1, 60 m.

**Figure 54a.**
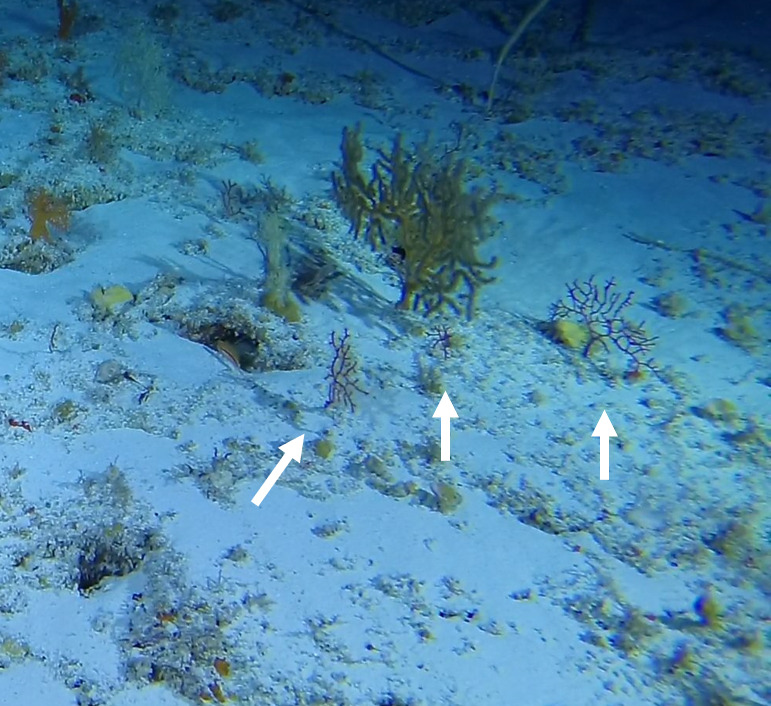
Aldabra W1, 120 m.

**Figure 54b.**
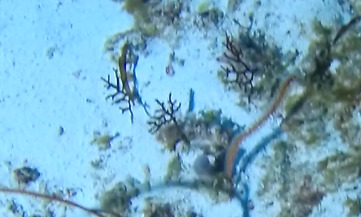
D'Arros N1, 120 m.

**Figure 55a.**
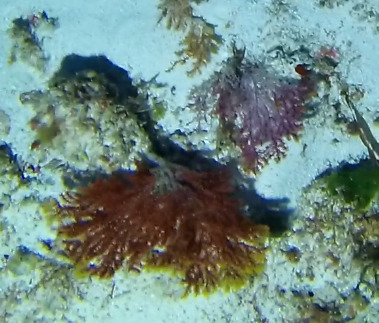
Desroches S1, 60 m.

**Figure 55b.**
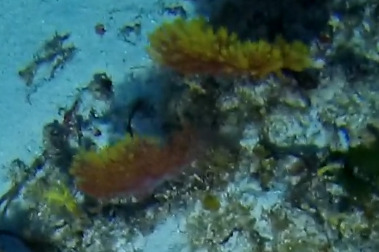
Desroches S1, 60 m.

**Figure 56a.**
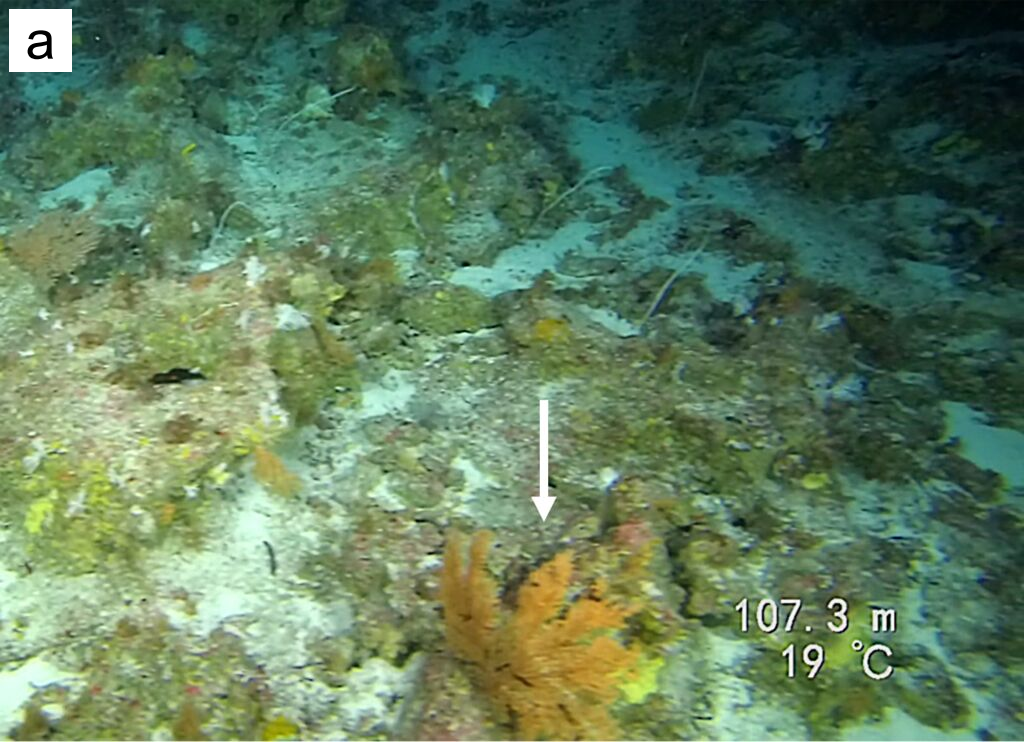
Alphonse N1, 108 m.

**Figure 56b.**
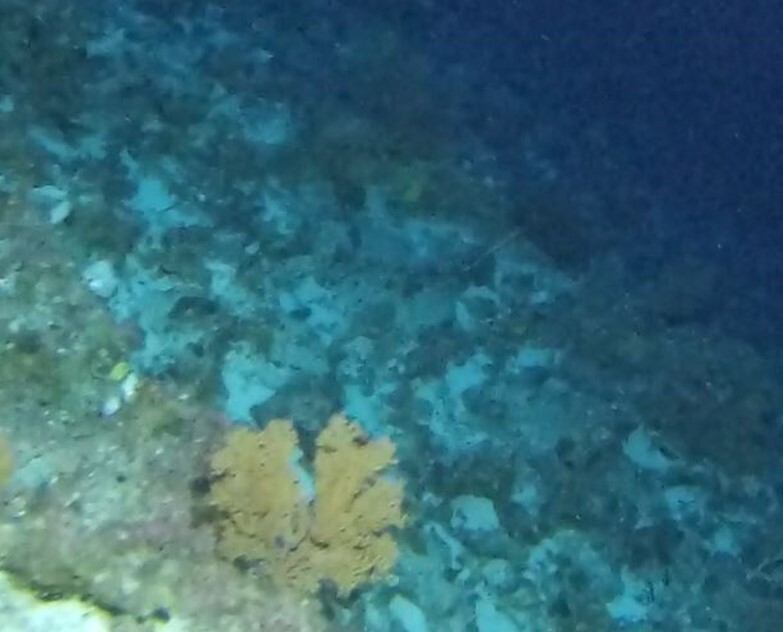
Alphonse N1, 100 m.

**Figure 58a.**
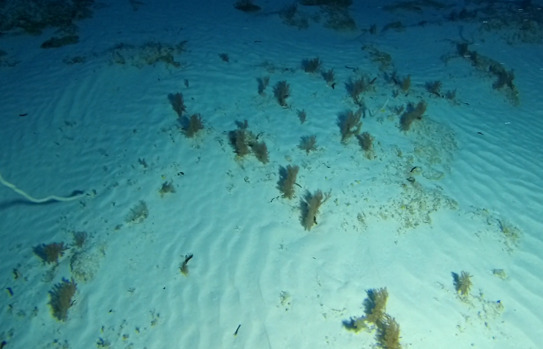
Aldabra N1, 120 m.

**Figure 58b.**
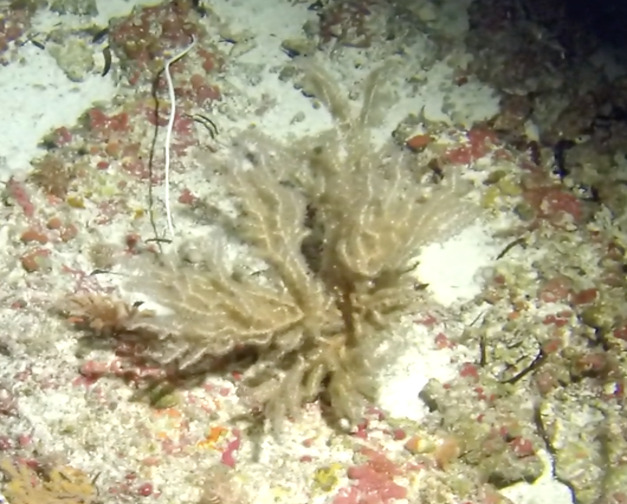
Alphonse N1, 107 m.

**Figure 59a.**
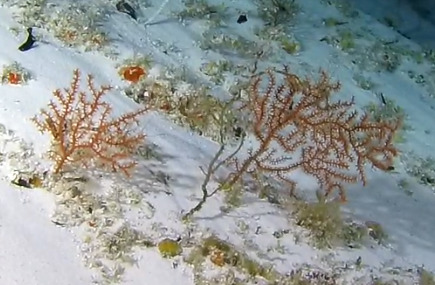
Aldabra N1, 100 m.

**Figure 59b.**
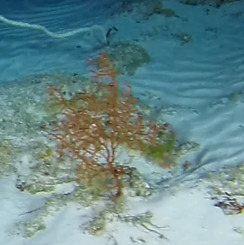
Aldabra N1, 100 m.

**Figure 59c.**
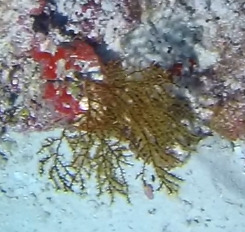
Aldabra N1, 60 m.

**Figure 59d.**
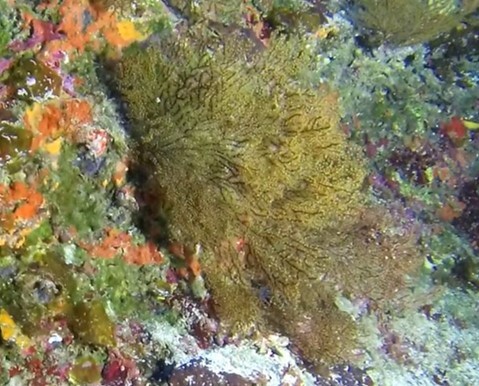
Astove W1, 60 m.

**Figure 60a.**
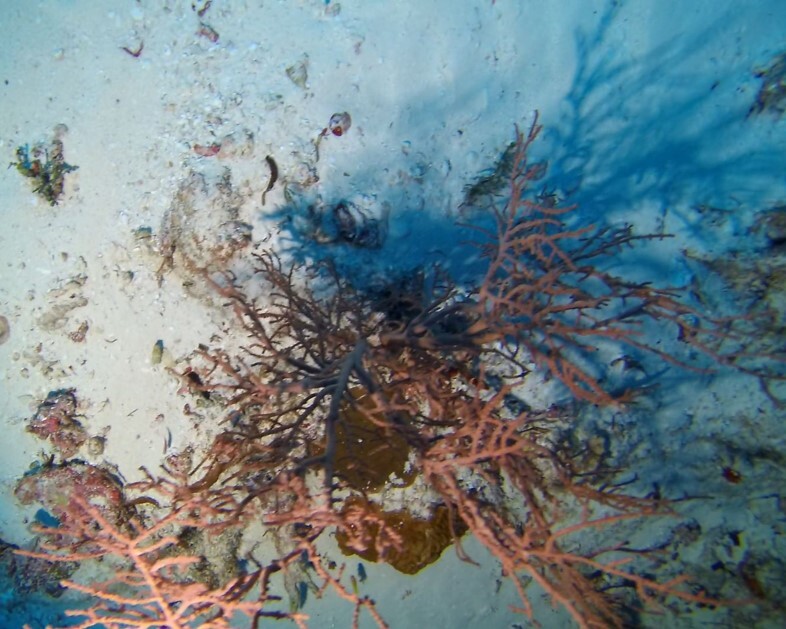
Aldabra N1, 60 m.

**Figure 60b.**
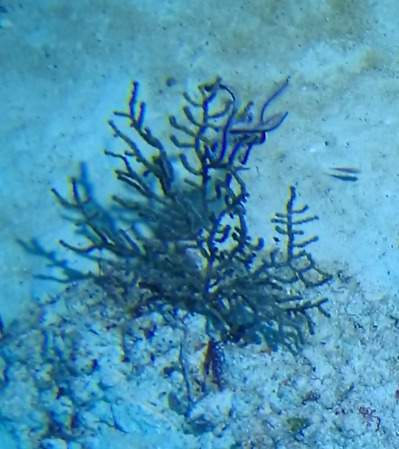
Aldabra W1, 60 m.

**Figure 60c.**
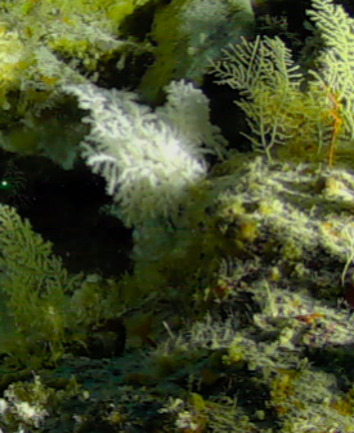
Aldabra N1, 120 m.

**Figure 60d.**
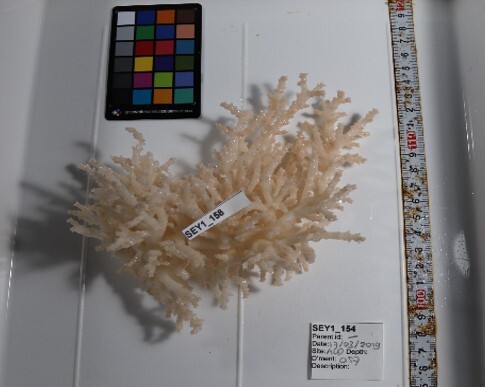
Aldabra N1, 120 m, collected specimen (SEY1_158) corresponding to the in-situ colony of Fig. 63c.

**Figure 63a.**
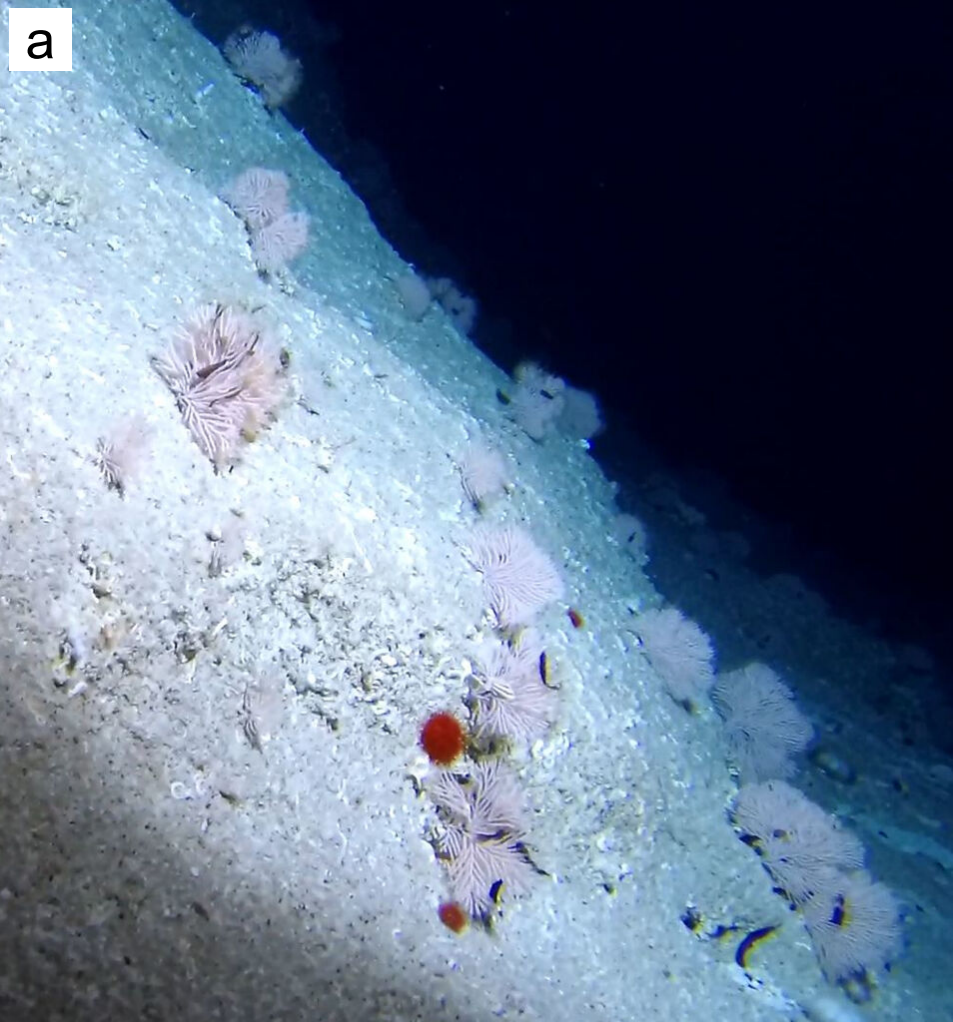
Alphonse N1, 250 m.

**Figure 63b.**
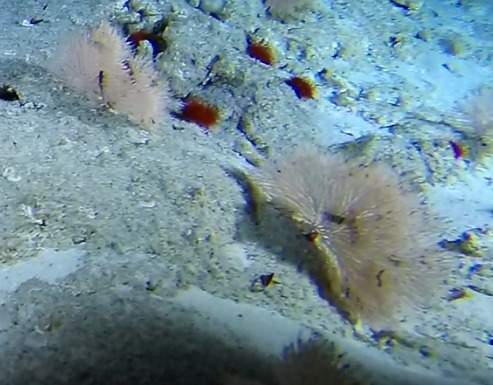
Alphonse N1, 242 m.

**Figure 64a.**
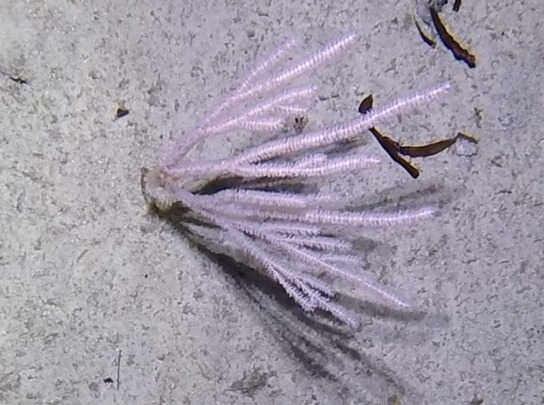
D'Arros N1, 350 m.

**Figure 64b.**
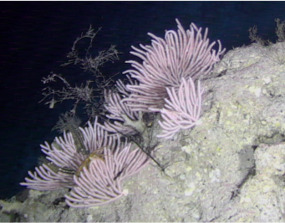
Aldabra N1, 190 m.

**Figure 64c.**
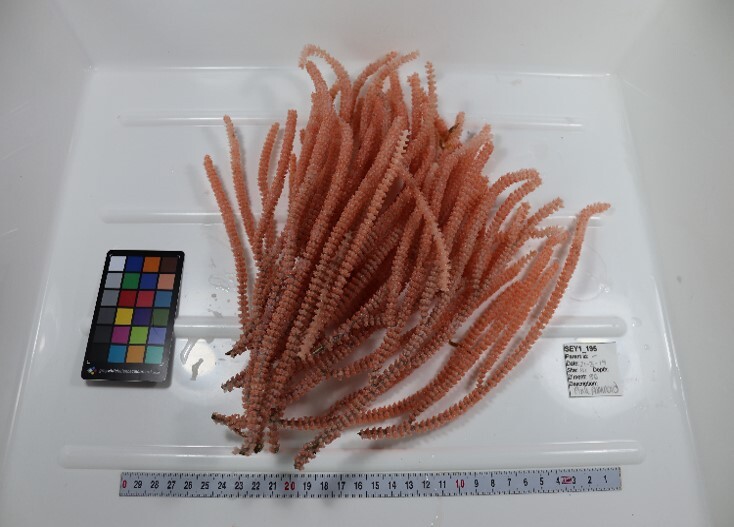
Aldabra N1, 190 m, collected specimen (SEY1_195) corresponding to the in-situ colony of Fig. 67b.

**Figure 65a.**
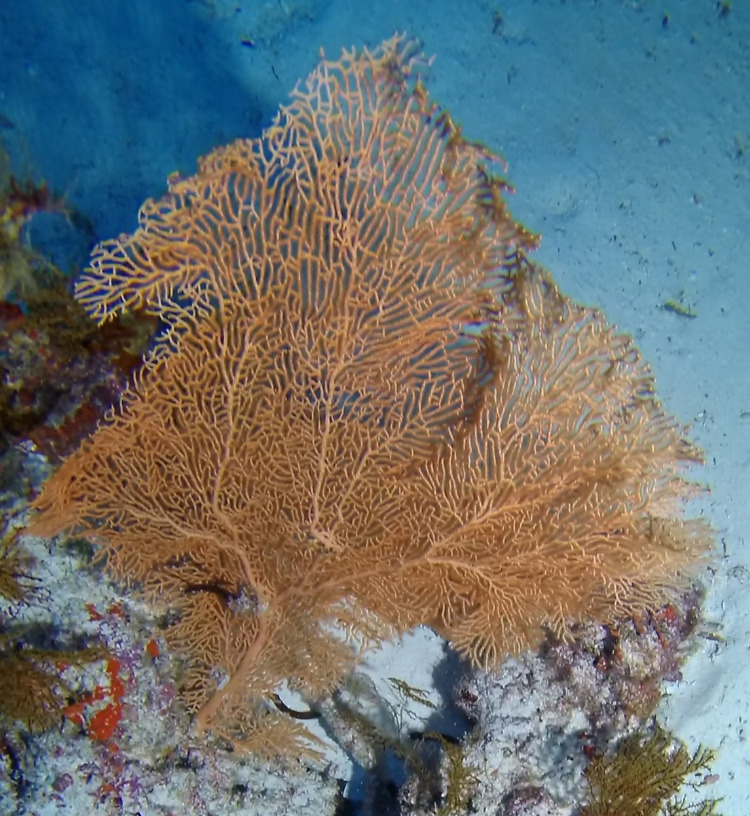
Aldabra N1, 60 m.

**Figure 65b.**
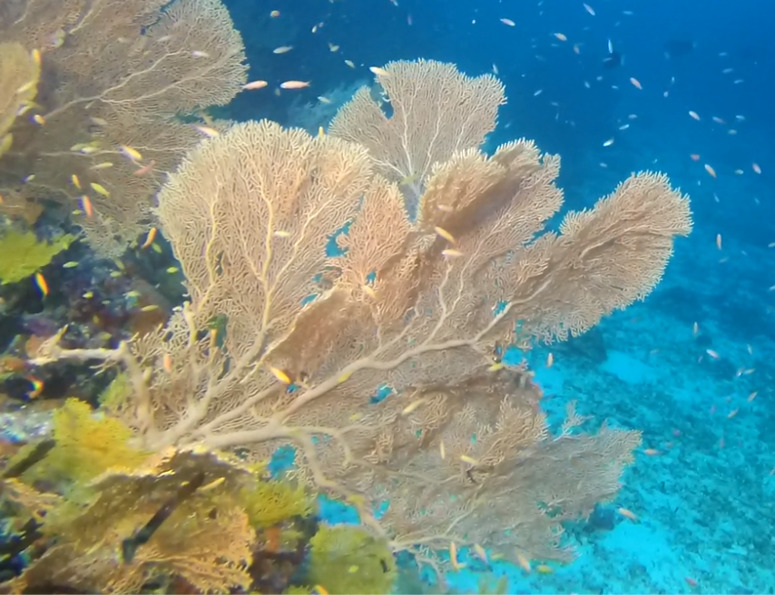
Alphonse N1, 60 m.

**Figure 65c.**
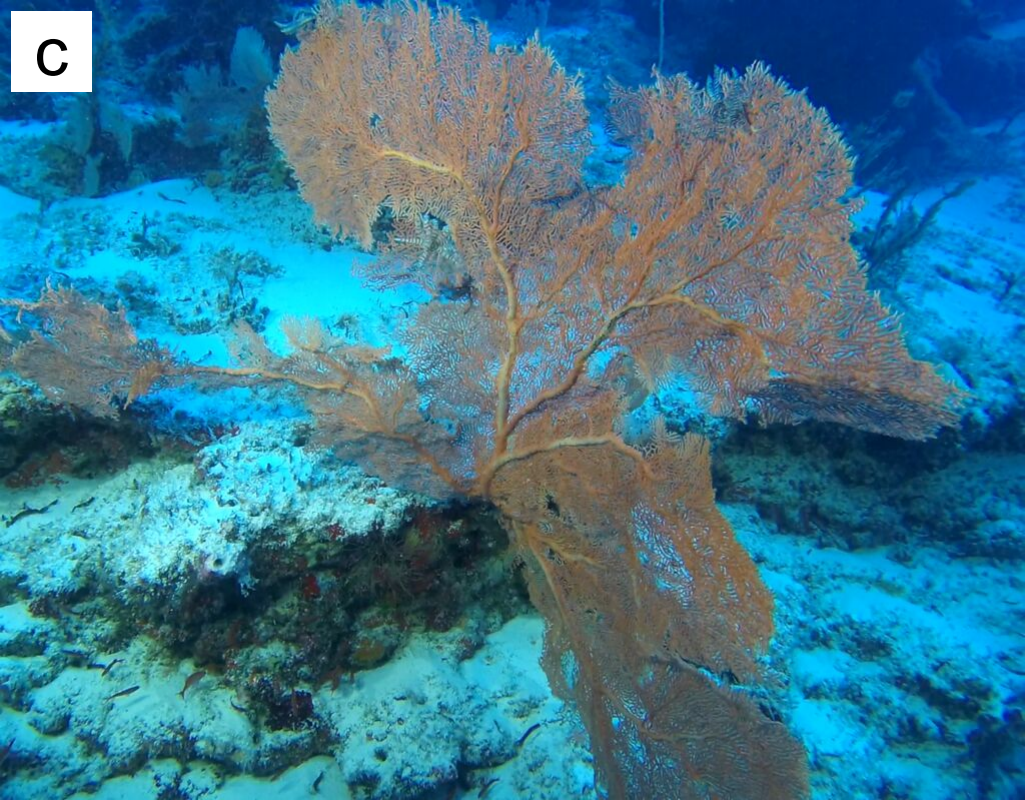
Aldabra N1, 60 m.

**Figure 65d.**
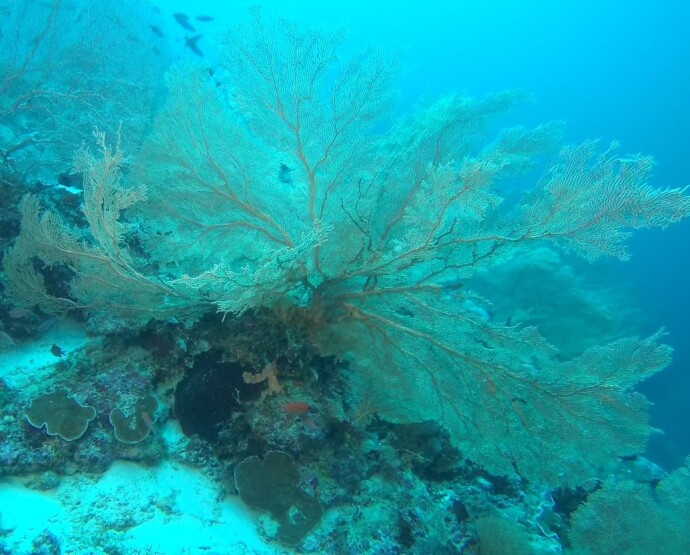
Aldabra N1, 30 m.

**Figure 65e.**
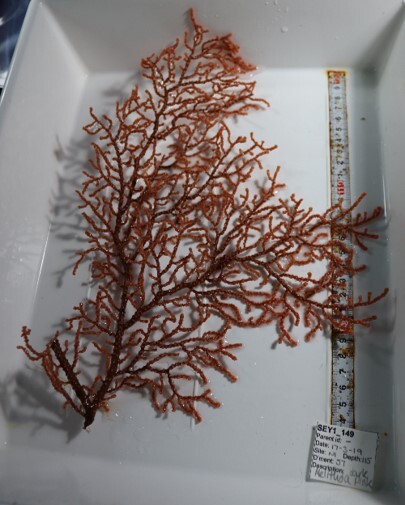
Aldabra N1, 30 m, collected specimen (SEY1_149).

**Figure 66a.**
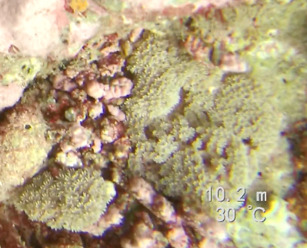
Alphonse N1, 10 m.

**Figure 66b.**
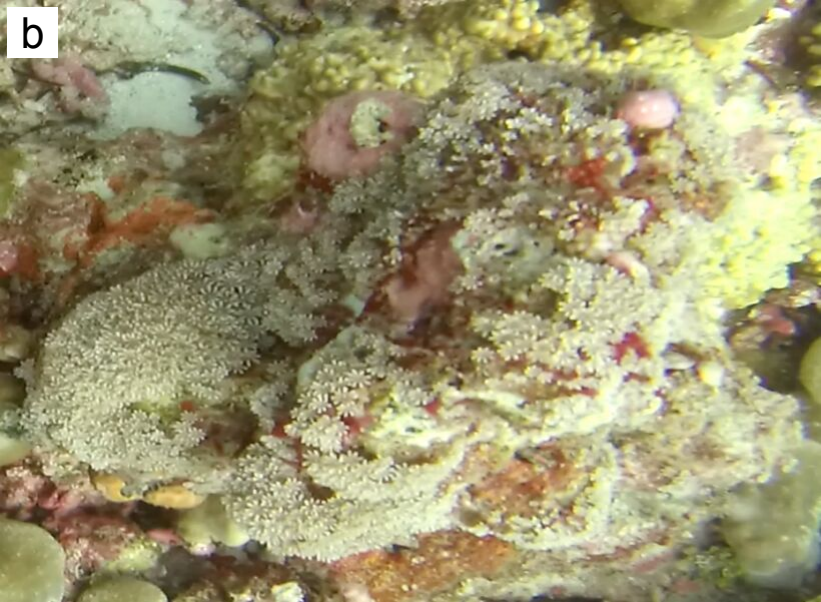
Alphonse N1, 10 m.

**Figure 68a.**
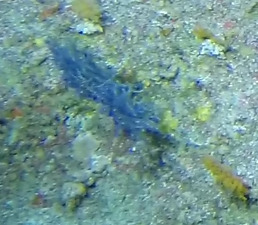
D'Arros N1, 120 m.

**Figure 68b.**
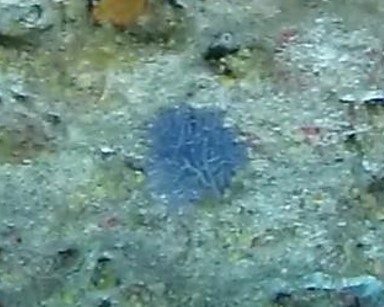
D'Arros N1, 120 m.

**Figure 70a.**
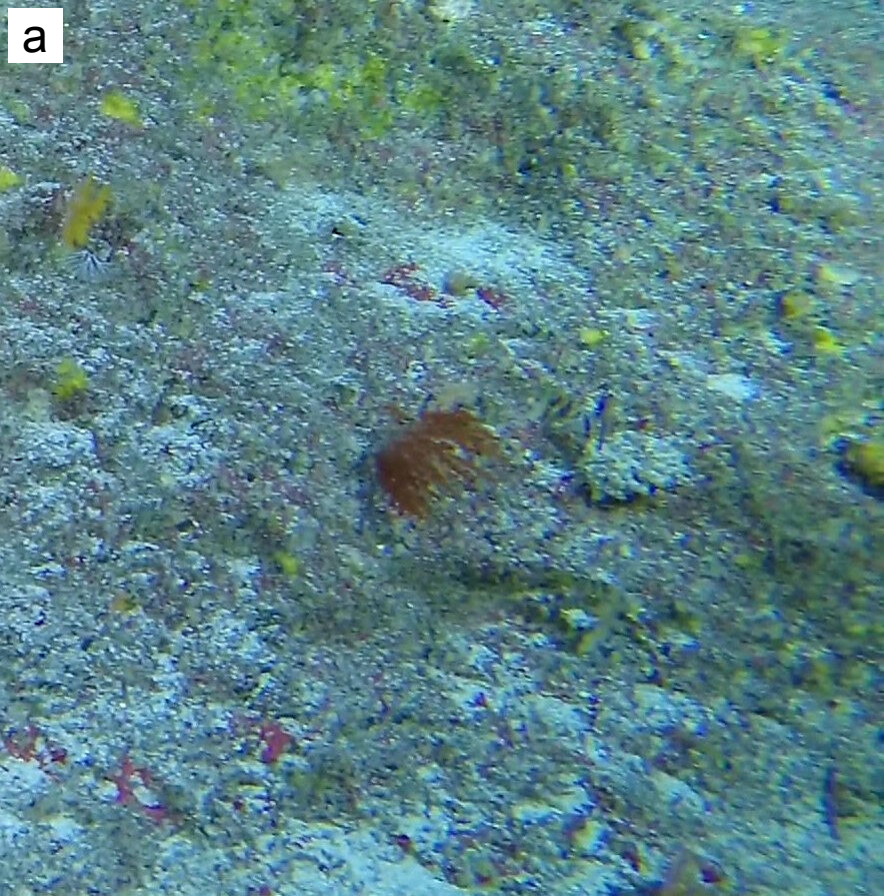
D'Arros N1, 120 m.

**Figure 70b.**
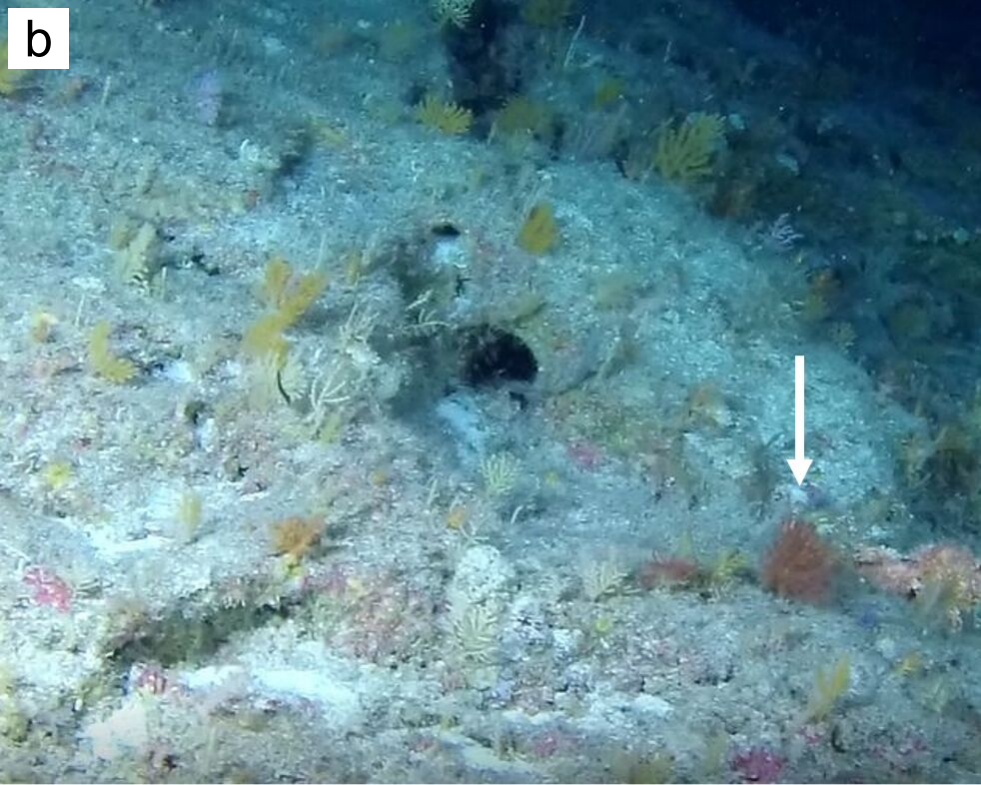
D'Arros N1, 120 m.

**Figure 73a.**
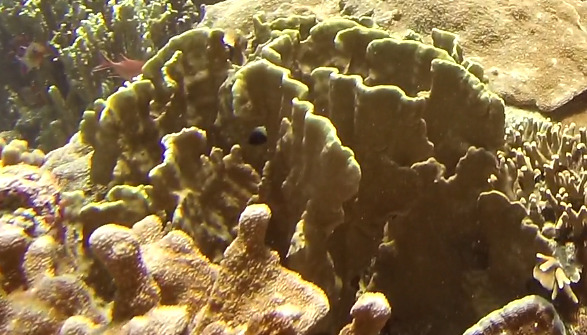
Astove W1, 10 m.

**Figure 73b.**
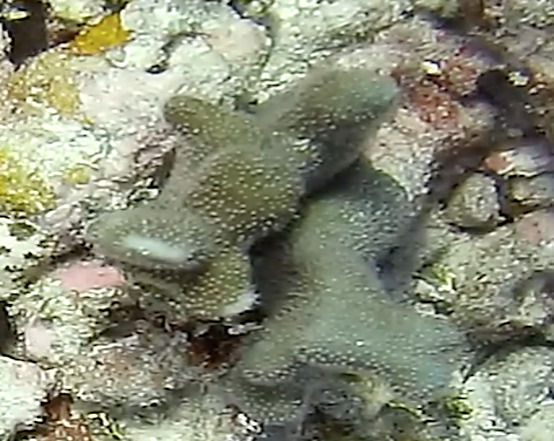
Aldabra N1, 10 m.

**Figure 74a.**
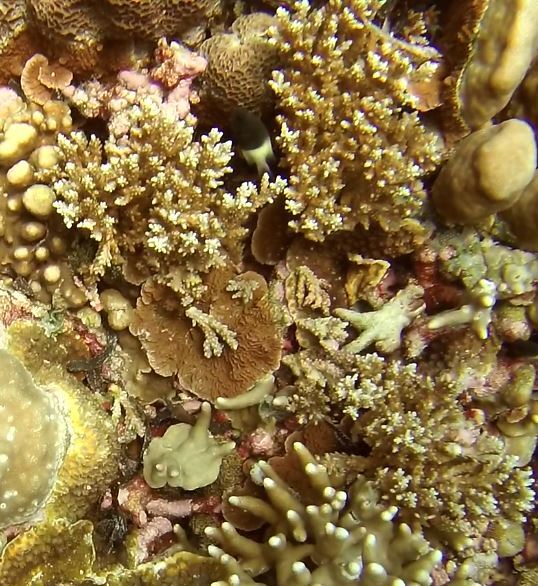
Branching colony. Astove W1, 10 m.

**Figure 74b.**
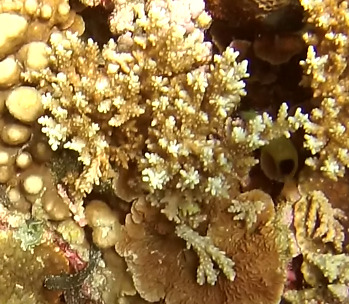
Branching colony. Astove W1, 10 m.

**Figure 74c.**
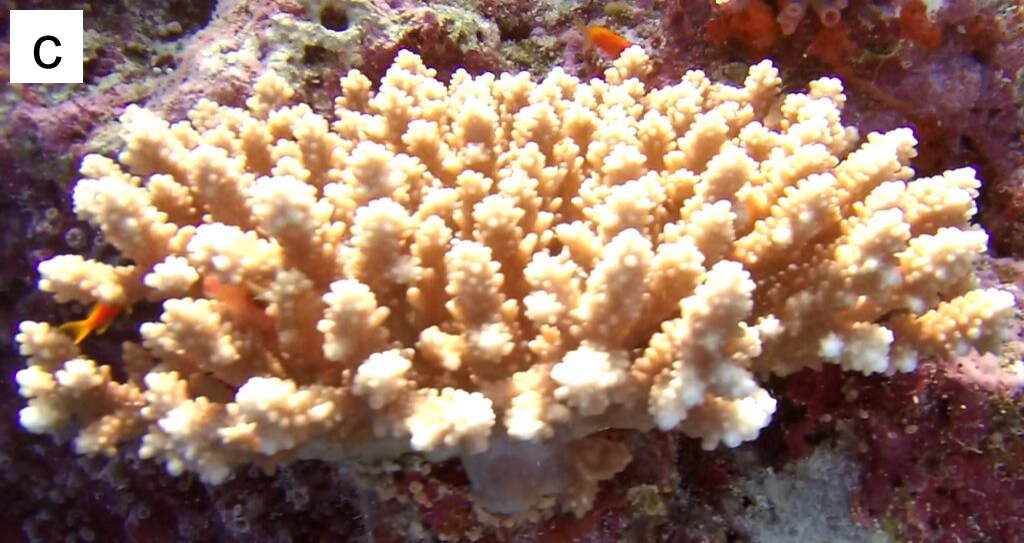
Tabulate colony. Astove W1, 10 m.

**Figure 74d.**
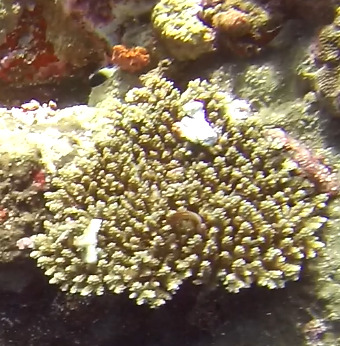
Tabulate colony. Astove W1, 10 m.

**Figure 74e.**
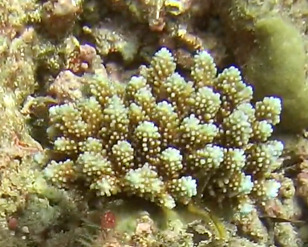
Digitate colony. Alphonse N1, 10 m.

**Figure 75a.**
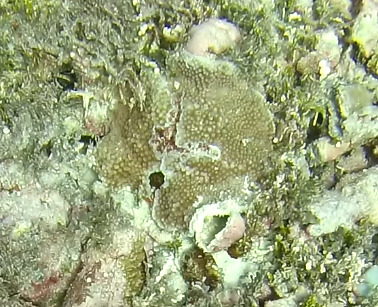
Encrusting colony. Aldabra N1, 10 m.

**Figure 75b.**
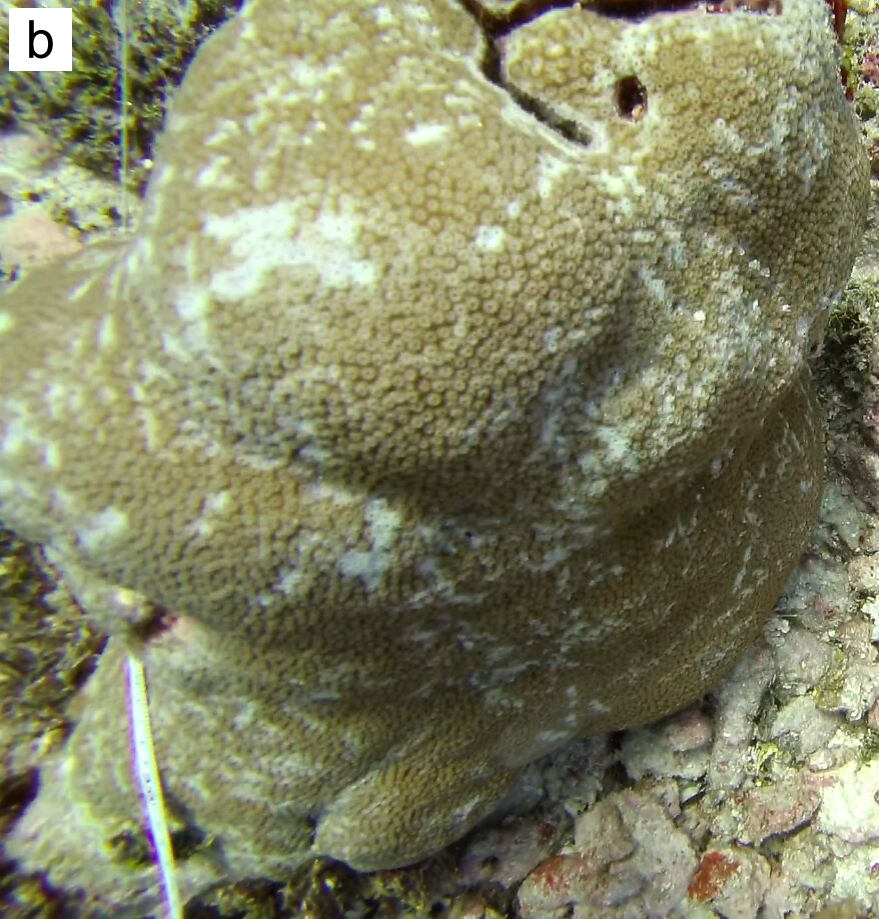
Massive colony. Aldabra N1, 10 m.

**Figure 76a.**
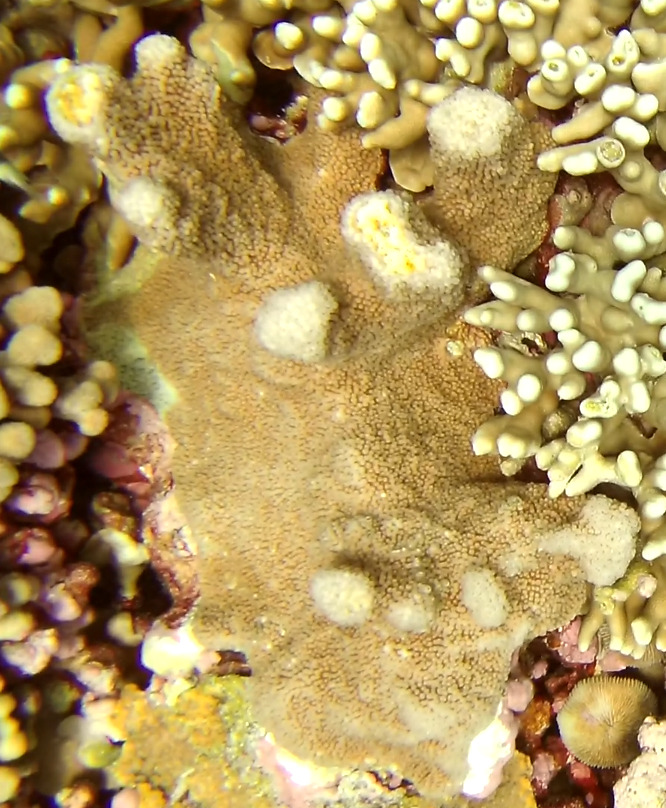
Encrusting-columnar colony. Astove W1, 10 m.

**Figure 76b.**
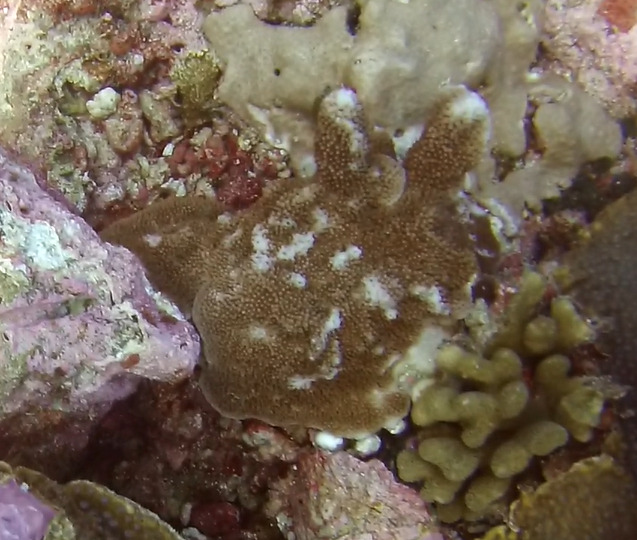
Encrusting-columnar colony. Astove W1, 10 m.

**Figure 76c.**
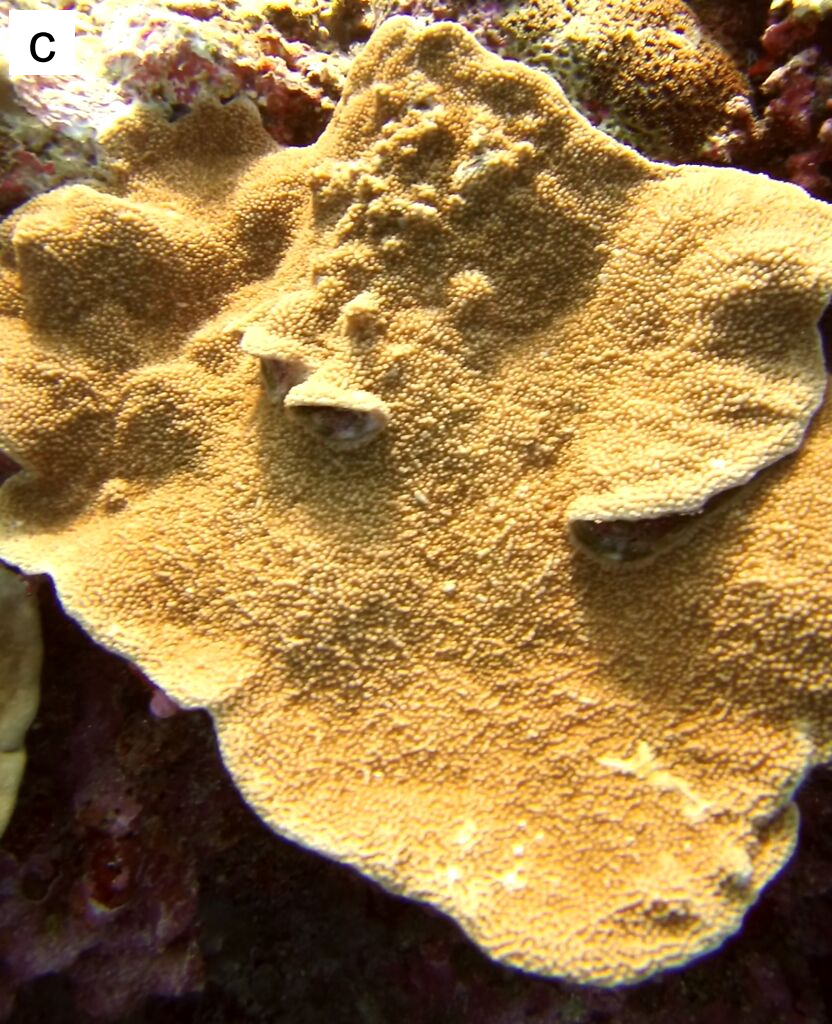
Encrusting colony. Astove W1, 10 m.

**Figure 76d.**
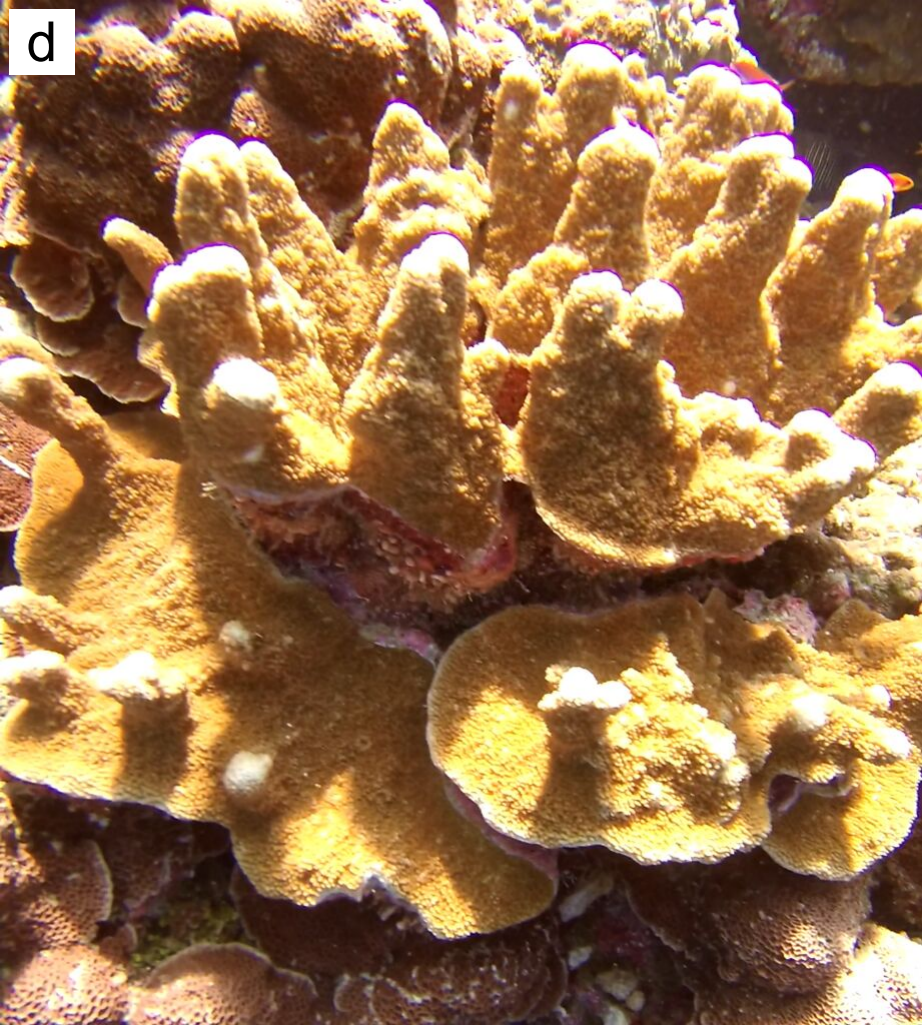
Columnar colony. Astove W1, 10 m.

**Figure 77a.**
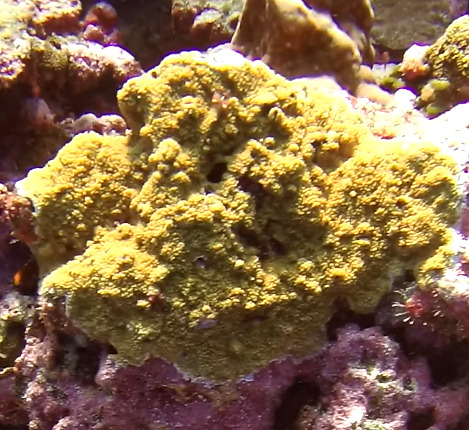
Encrusting colony. Astove W1, 10 m.

**Figure 77b.**
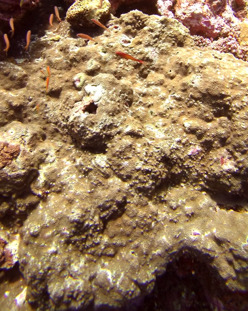
Encrusting colony. Astove W1, 10 m.

**Figure 79a.**
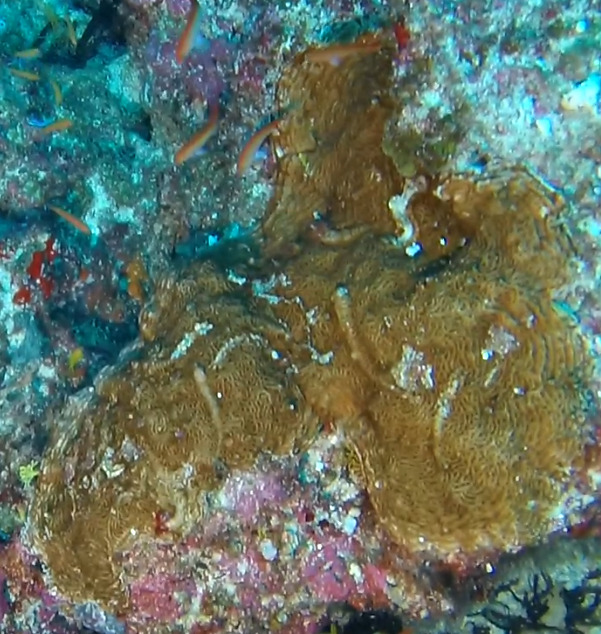
Encrusting colony. Alphonse N1, 60 m.

**Figure 79b.**
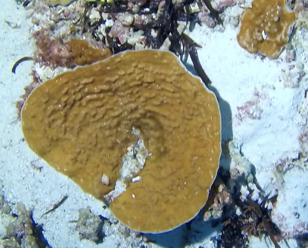
Plating colony. Alphonse N1, 60 m.

**Figure 80a.**
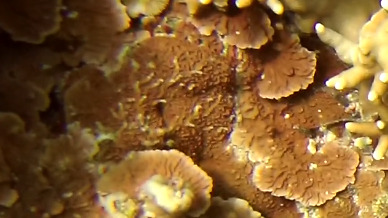
Laminar colony. Astove W1, 10 m.

**Figure 80b.**
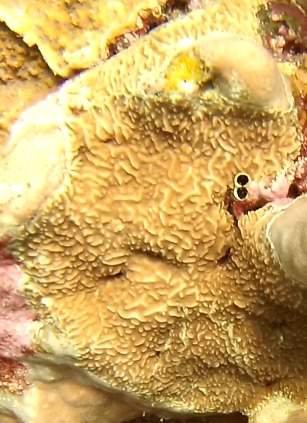
Encrusting colony. Astove W1, 10 m.

**Figure 81a.**
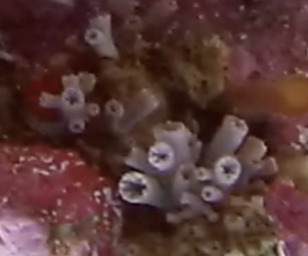
Astove W1, 10 m.

**Figure 81b.**
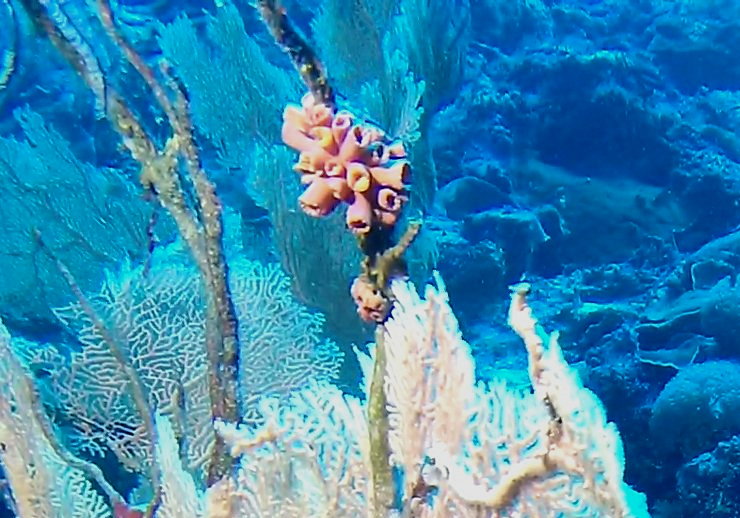
Aldabra N1, 30 m.

**Figure 83a.**
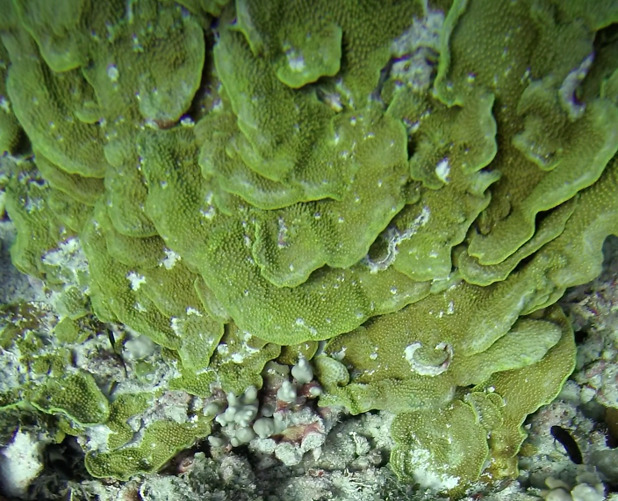
Laminar colony. Aldabra N1, 10 m.

**Figure 83b.**
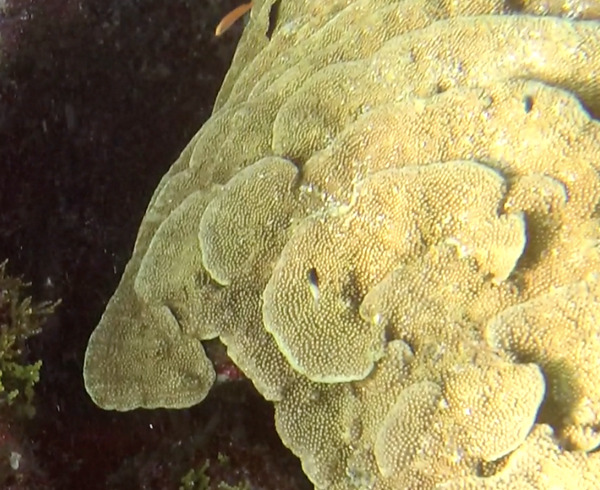
Laminar colony. Astove W1, 10 m.

**Figure 84a.**
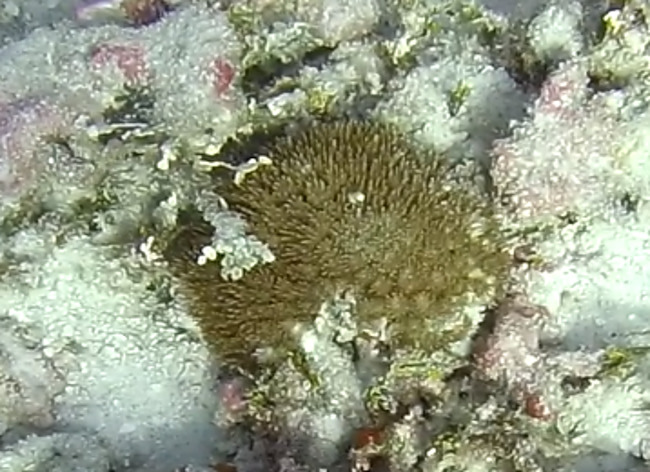
Massive colony. Aldabra N1, 10 m.

**Figure 84b.**
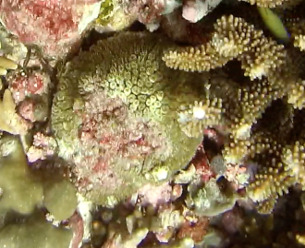
Massive colony. Alphonse N1, 10 m.

**Figure 85a.**
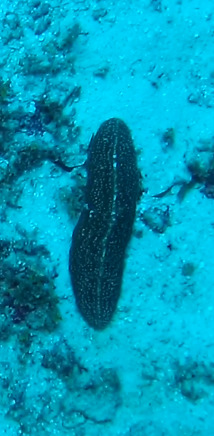
Desroches S1, 30 m.

**Figure 85b.**
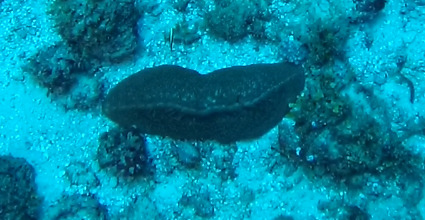
Desroches S1, 30 m.

**Figure 86a.**
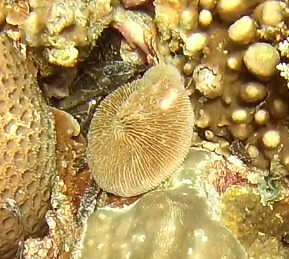
Astove W1, 10 m.

**Figure 86b.**
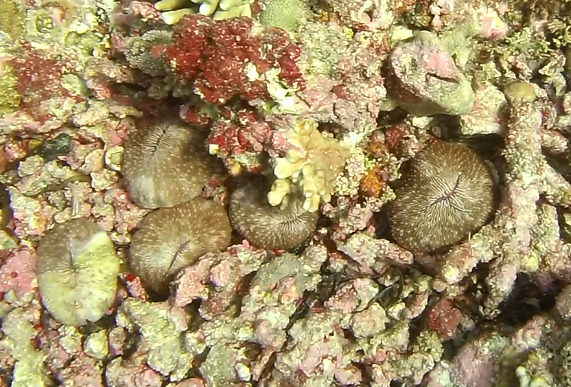
Aldabra N1, 10 m.

**Figure 88a.**
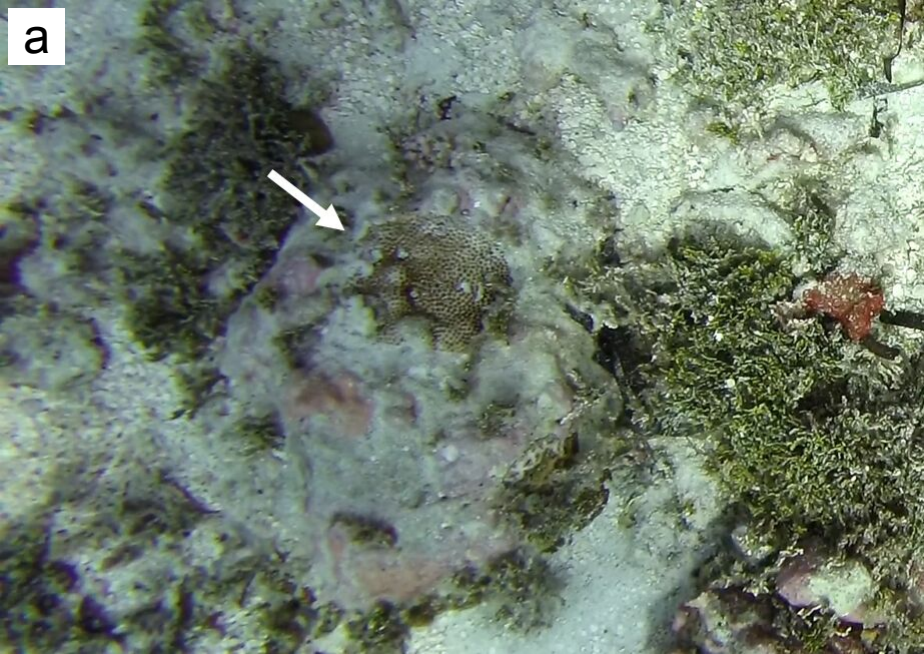
Encrusting colony. Aldabra W1, 10 m.

**Figure 88b.**
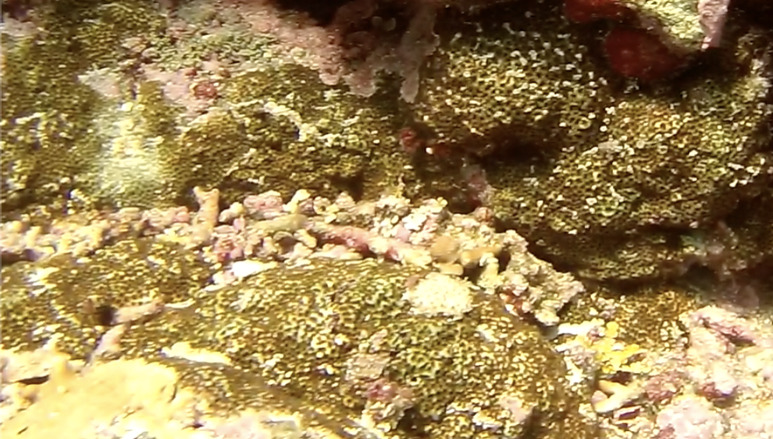
Encrusting colony. Astove W1, 10 m.

**Figure 89a.**
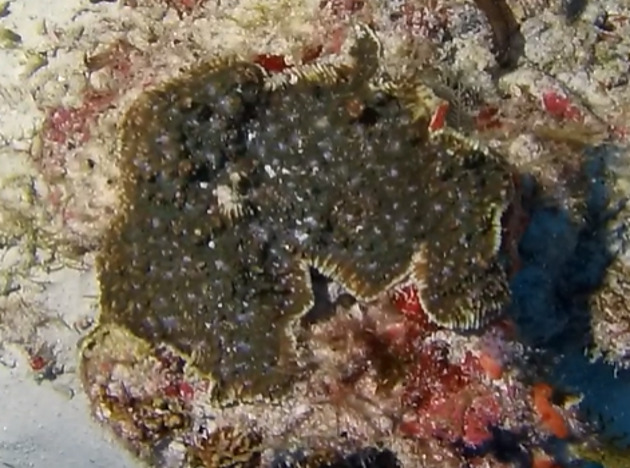
Encrusting colony. Aldabra N1, 60 m.

**Figure 89b.**
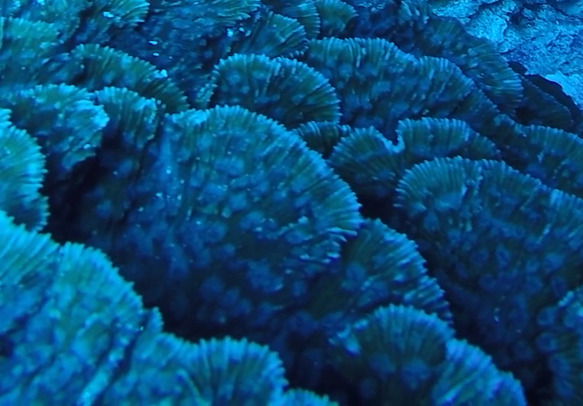
Laminar colony. Astove W1, 30 m.

**Figure 90a.**
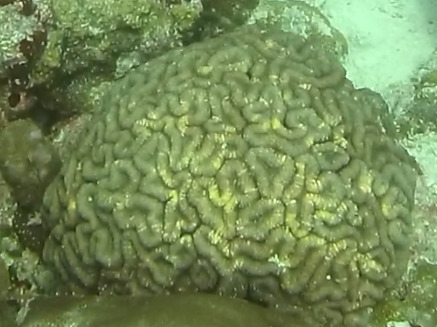
Massive colony. Aldabra W1, 10 m.

**Figure 90b.**
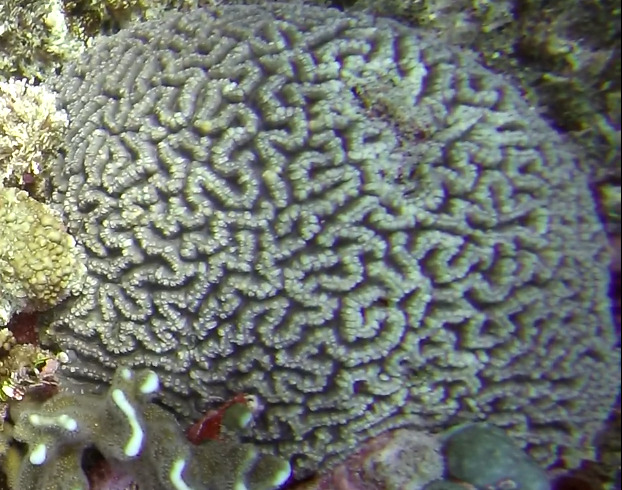
Massive colony. Aldabra N1, 10 m.

**Figure 91a.**
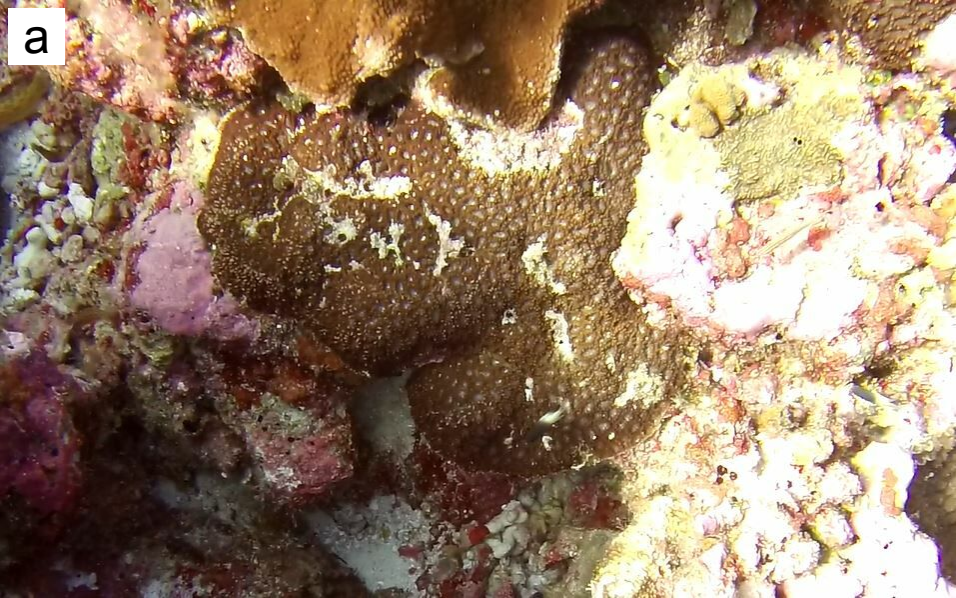
Encrusting colony. Astove W1, 10 m.

**Figure 91b.**
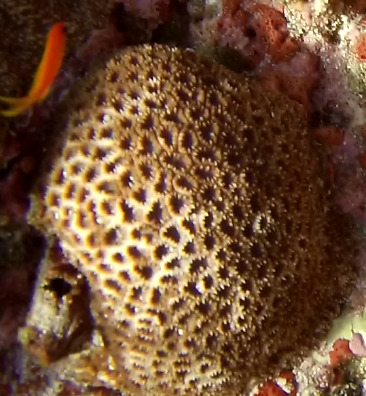
Massive colony. Aldabra N1, 10 m.

**Figure 92a.**
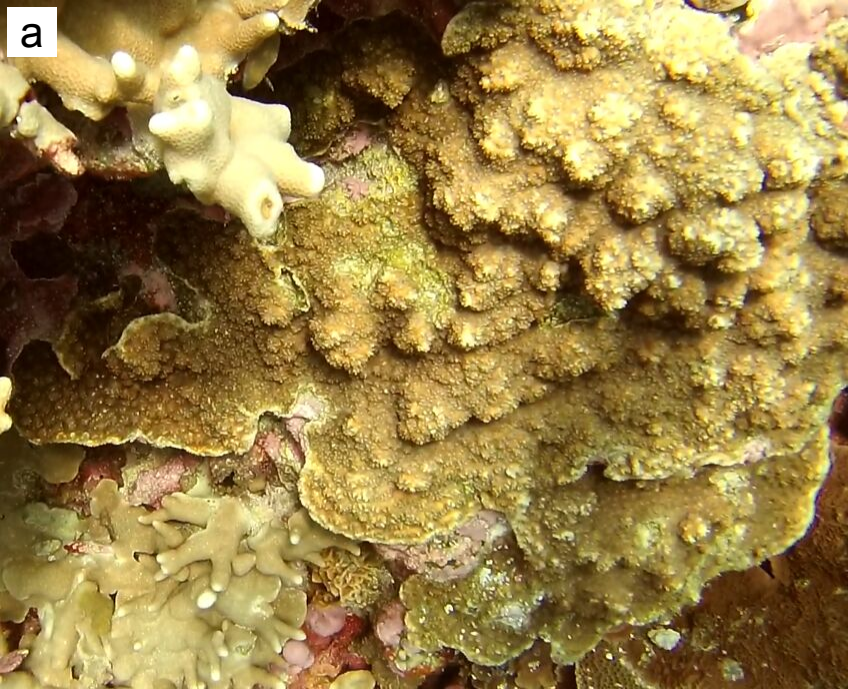
Encrusting colony. Astove W1, 10 m.

**Figure 92b.**
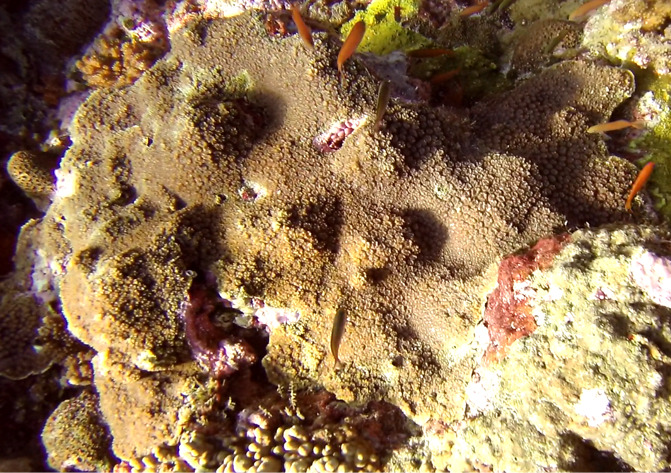
Encrusting colony. Astove W1, 10 m.

**Figure 92c.**
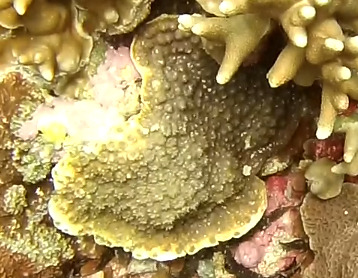
Laminar colony. Astove W1, 10 m.

**Figure 93a.**
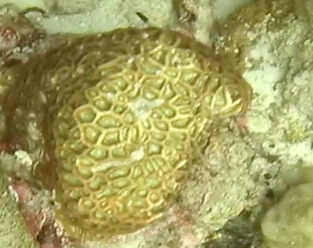
Massive colony. Alphonse N1, 10 m.

**Figure 93b.**
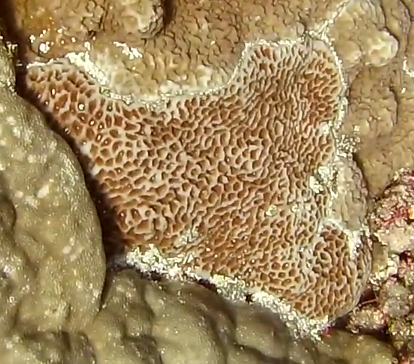
Encrusting colony. Astove W1, 10 m.

**Figure 94a.**
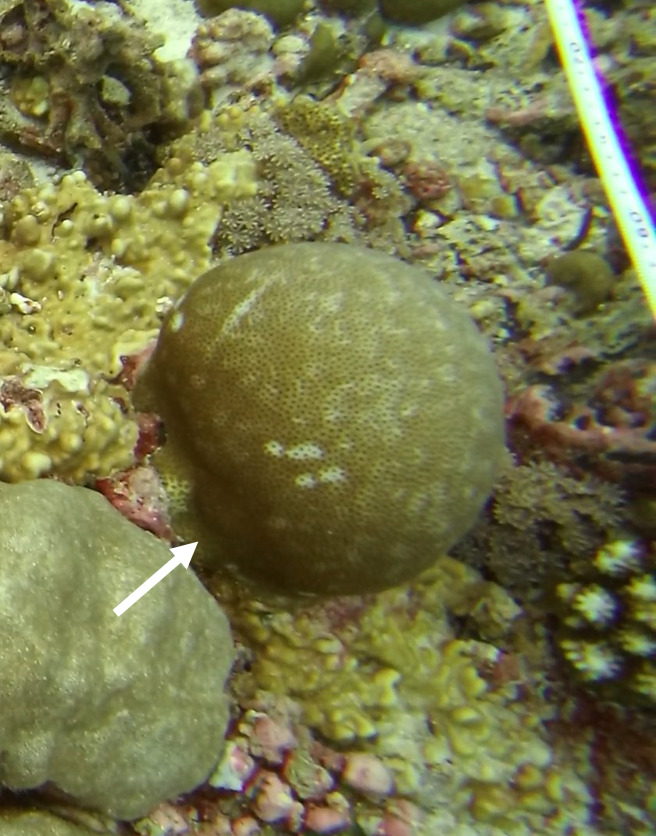
Massive colony. Alphonse N1, 10 m.

**Figure 94b.**
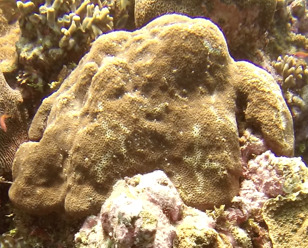
Massive colony. Astove W1, 10 m.

**Figure 94c.**
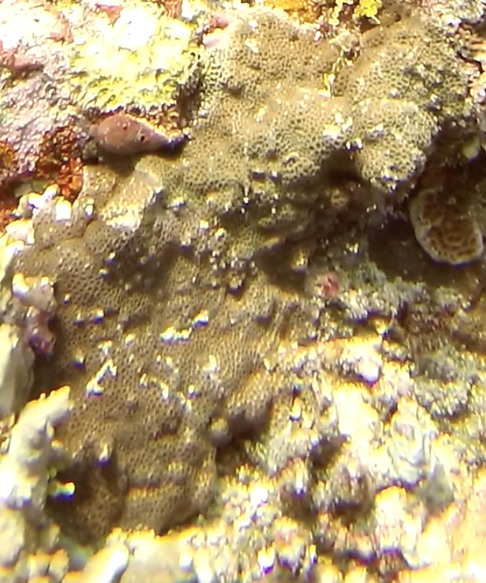
Encrusting colony. Astove W1, 10 m.

**Figure 97a.**
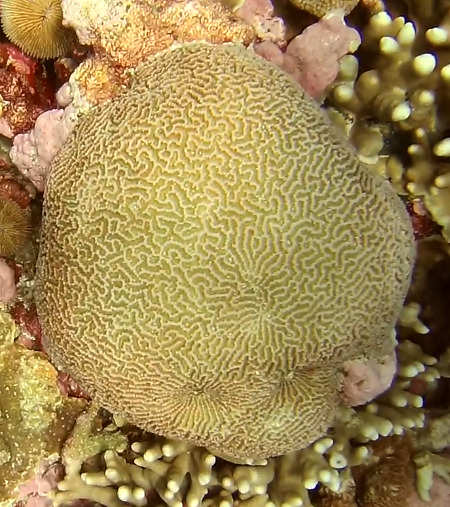
Massive colony. Astove W1, 10 m.

**Figure 97b.**
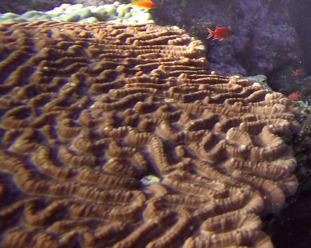
Encrusting colony. Astove W1, 10 m.

**Figure 98a.**
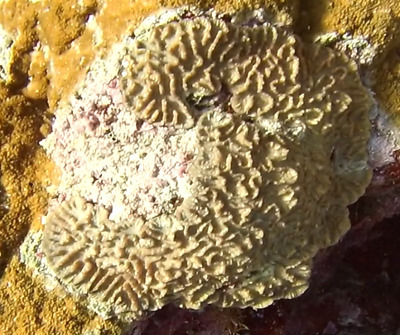
Encrusting colony. Astove W1, 10 m.

**Figure 98b.**
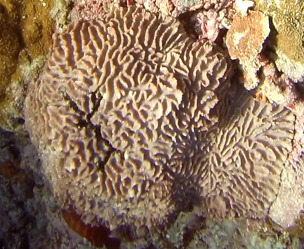
Encrusting colony. Astove W1, 10 m.

**Figure 99a.**
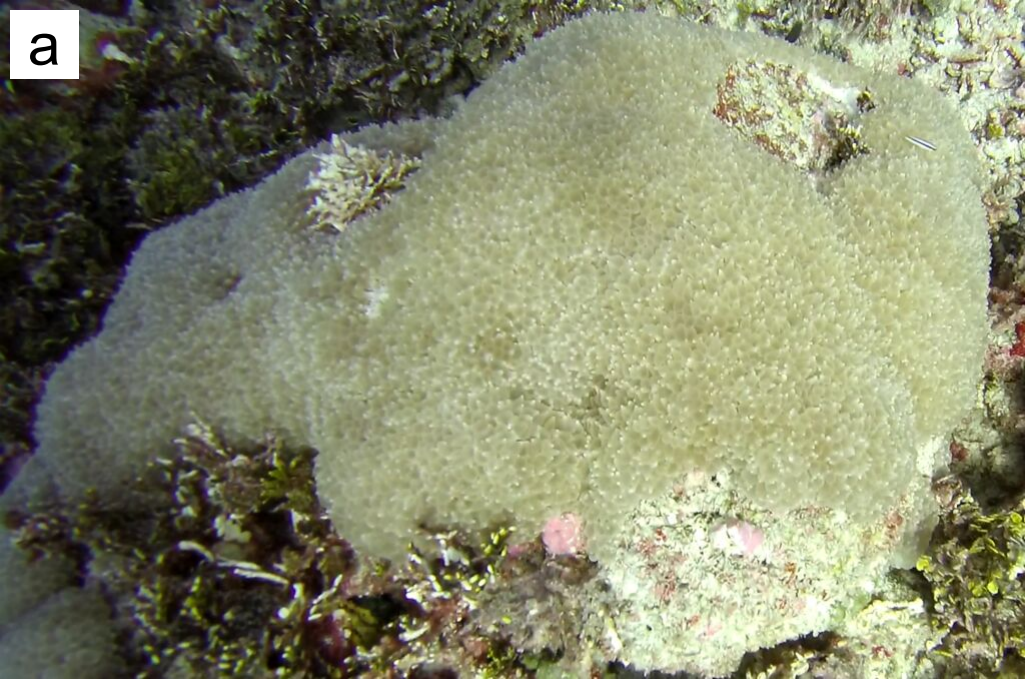
Massive colony. Aldabra N1, 10 m.

**Figure 99b.**
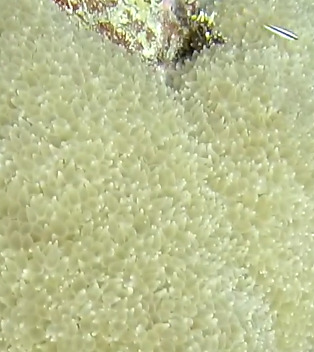
Massive colony. Aldabra N1, 10 m.

**Figure 100a.**
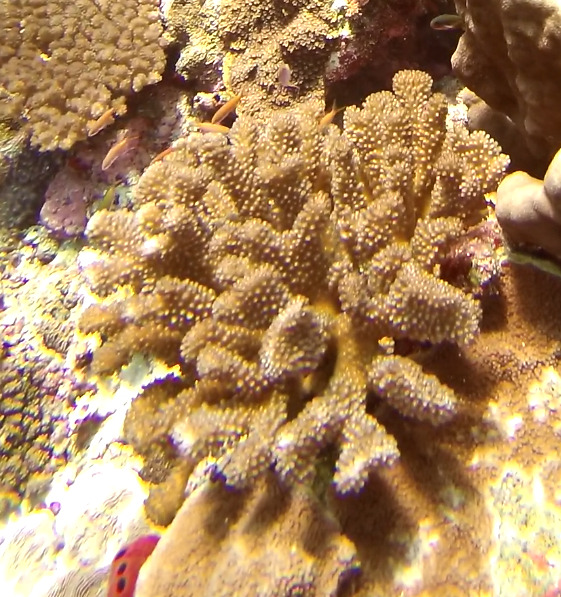
Branching colony. Astove W1, 10 m.

**Figure 100b.**
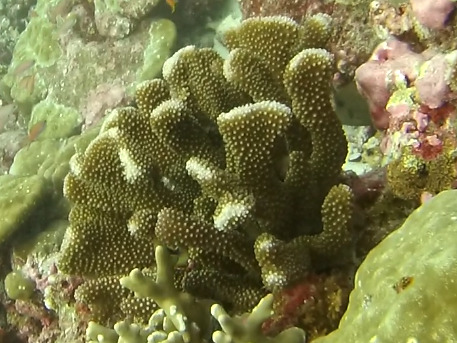
Branching colony. Alphonse N1, 10 m.

**Figure 101a.**
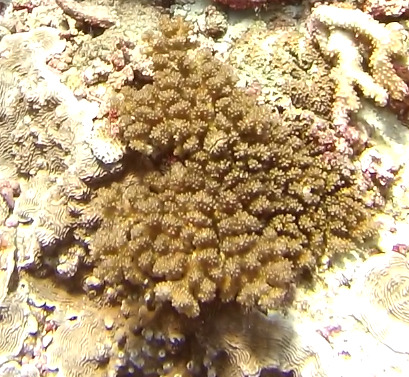
Branching colony. Astove W1, 10 m.

**Figure 101b.**
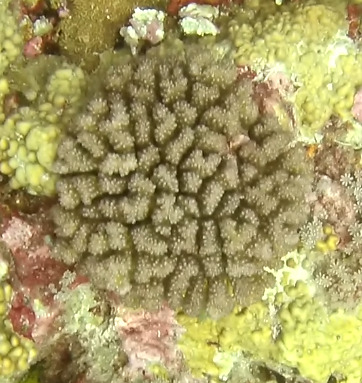
Branching colony. Alphonse N1, 10 m.

**Figure 103a.**
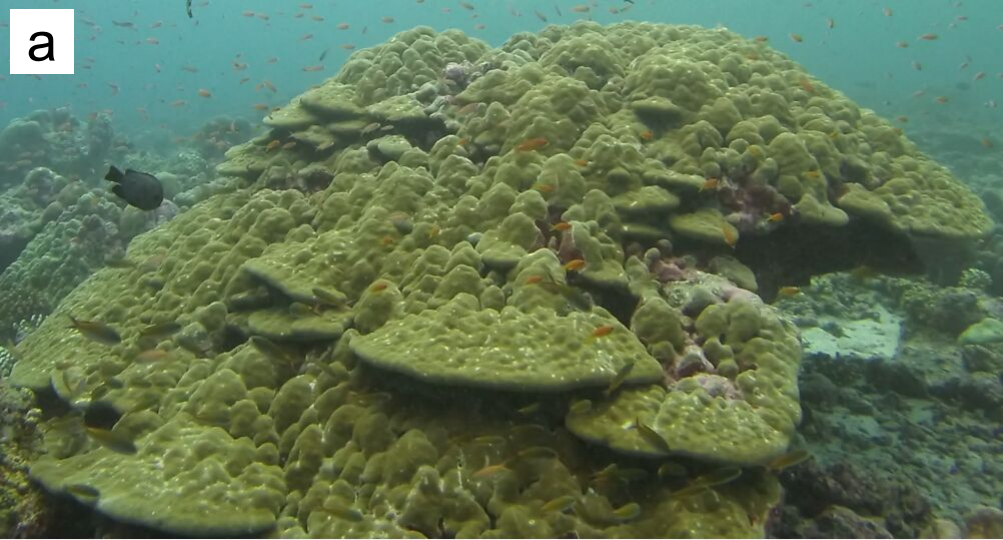
Massive colony. Alphonse N1, 10 m.

**Figure 103b.**
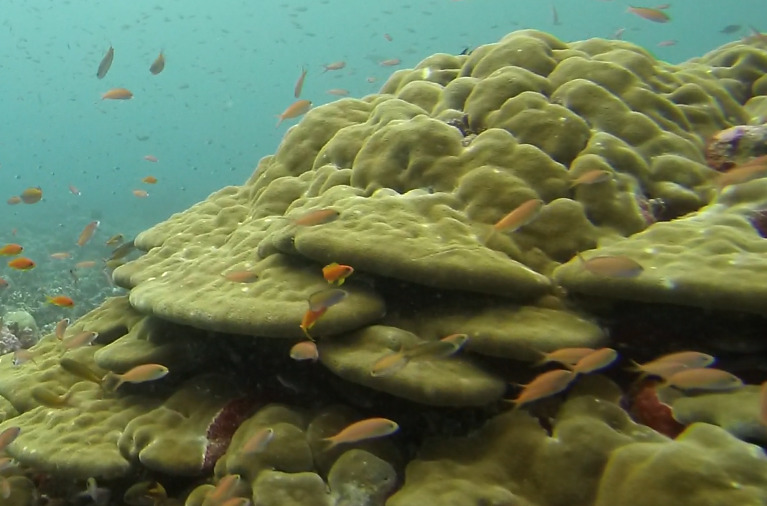
Massive colony. Alphonse N1, 10 m.

**Figure 103c.**
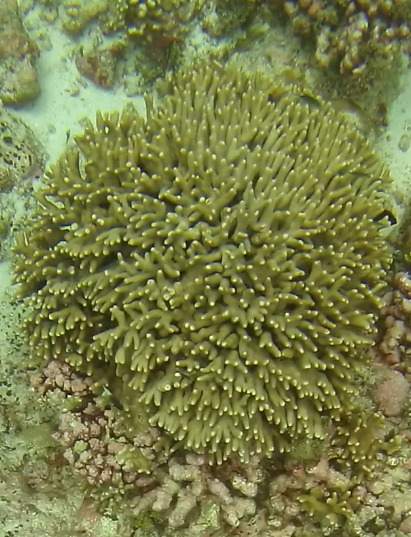
Branching colony. Poivre E1, 10 m.

**Figure 103d.**
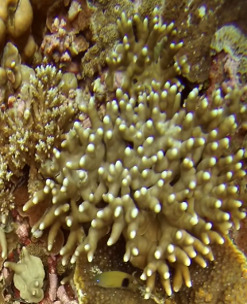
Branching colony. Astove W1, 10 m.

**Figure 103e.**
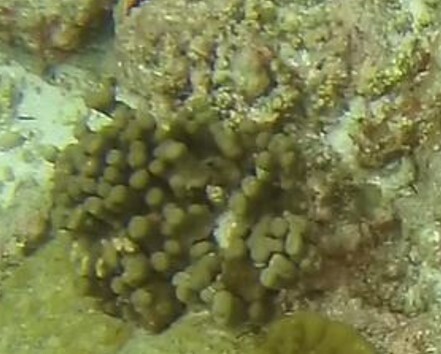
Sub-massive colony. Poivre E1, 10 m.

**Figure 103f.**
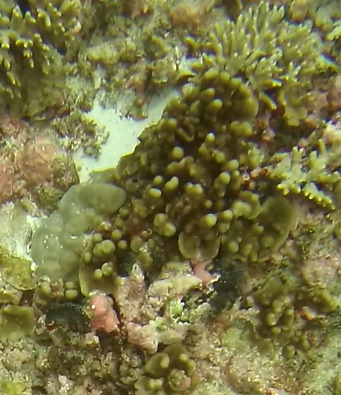
Sub-massive colony. Poivre E1, 10 m.

**Figure 105a.**
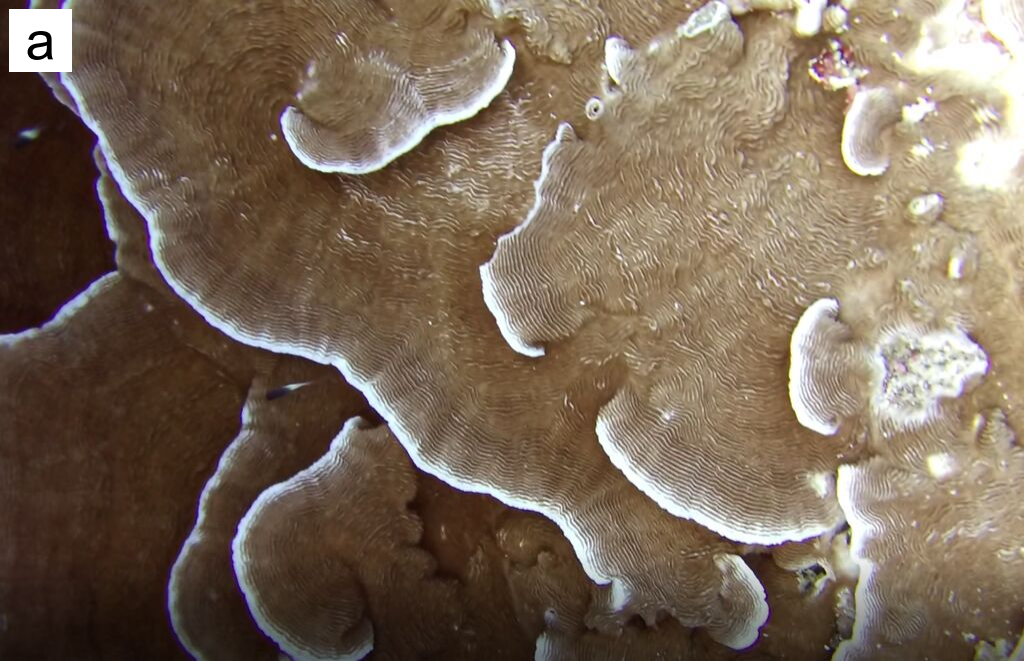
Laminar colony. Astove W1, 10 m.

**Figure 105b.**
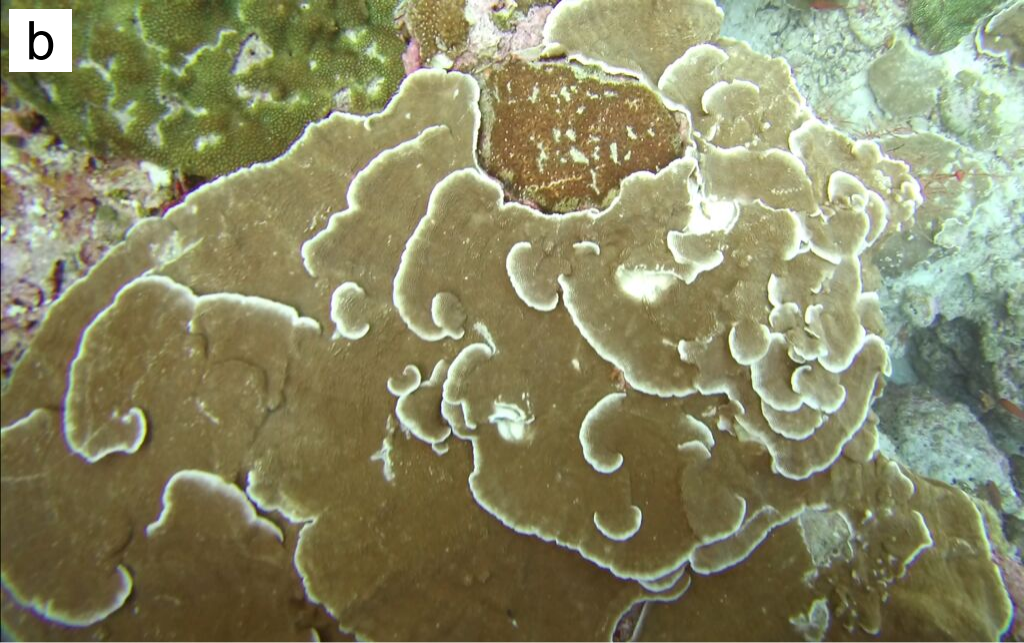
Laminar colony. Astove W1, 10 m.

**Figure 106a.**
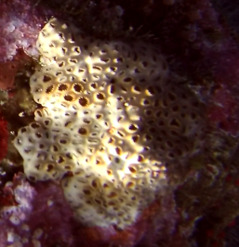
Astove W1, 10 m.

**Figure 106b.**
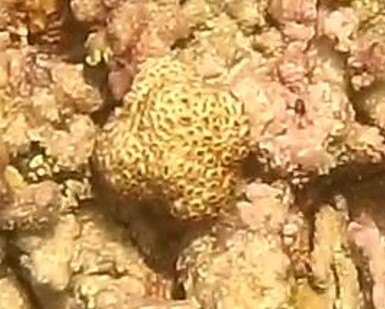
Astove W1, 10 m.

**Figure 107a.**
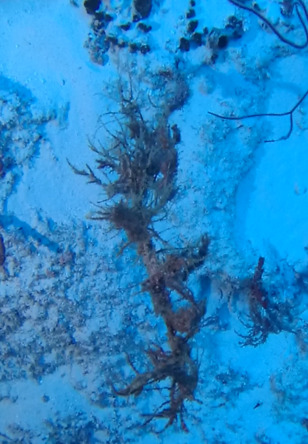
Aldabra N1, 60 m.

**Figure 107b.**
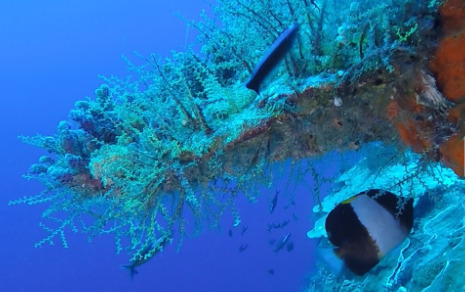
Astove W1, 30 m.

**Figure 108a.**
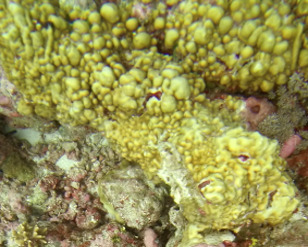
Alphonse N1, 10 m.

**Figure 108b.**
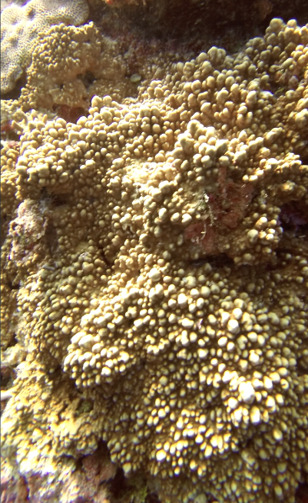
Astove W1, 10 m.

**Figure 108c.**
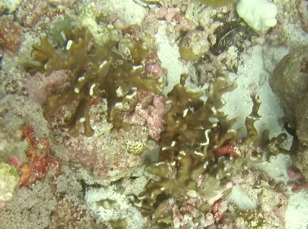
Alphonse N1, 10 m.

**Figure 108d.**
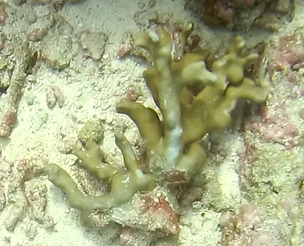
Alphonse N1, 10 m.

**Figure 110a.**
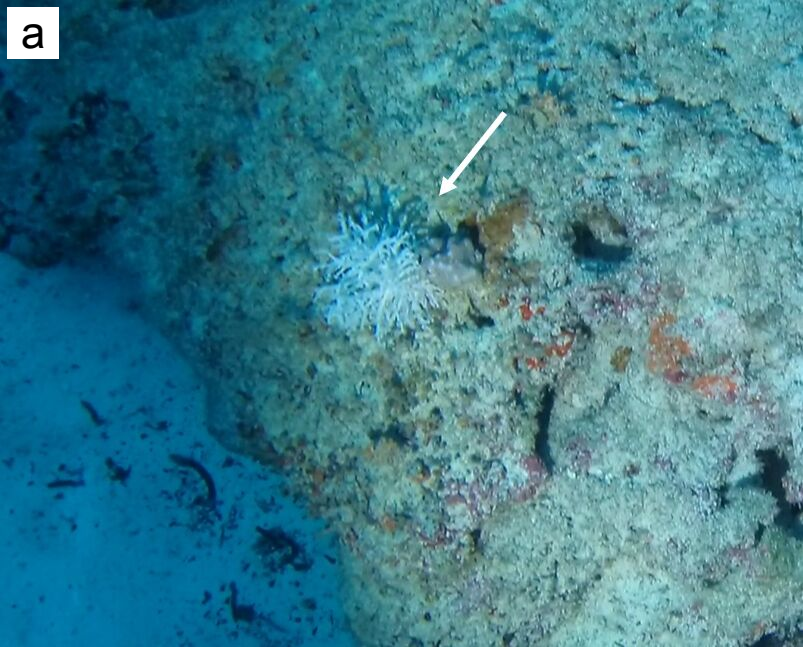
Aldabra W1, 60 m.

**Figure 110b.**
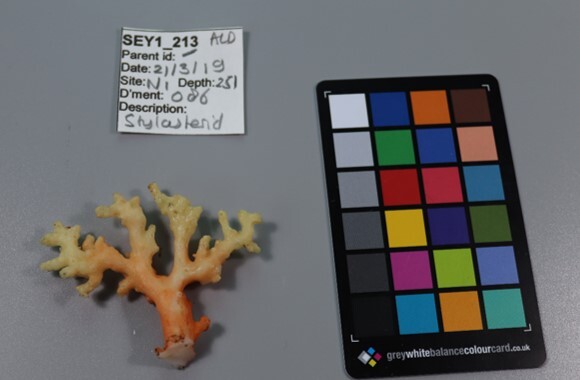
Aldabra N1, 250 m. (collected specimen SEY1_213)

**Figure 111a.**
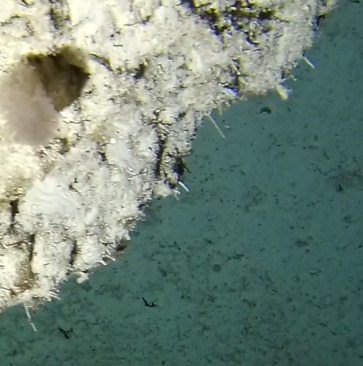
Aldabra W1, 250 m.

**Figure 111b.**
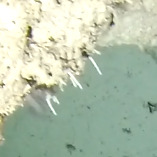
Aldabra W1, 250 m. Close-up of Fig. 111a.

**Figure 112a.**
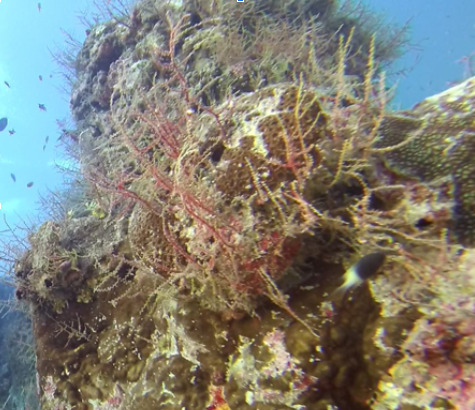
Astove W1, 10 m.

**Figure 112b.**
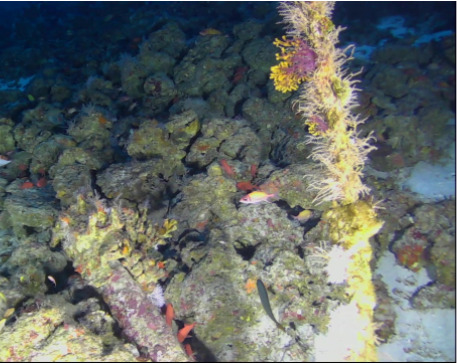
Astove W1, 60 m.

**Figure 112c.**
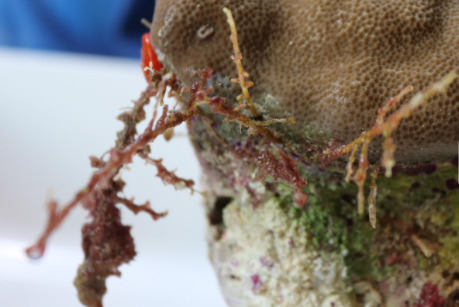
Specimen attached to a collected *Porites* coral.

**Figure 113a.**
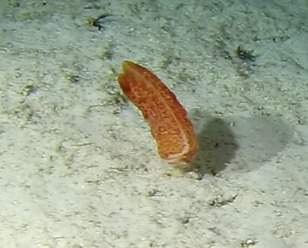
Aldabra W1, 250 m.

**Figure 113b.**
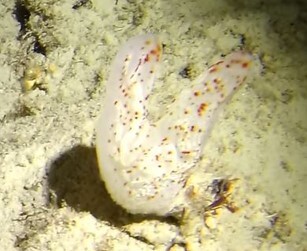
Aldabra W1, 250 m.

**Figure 113c.**
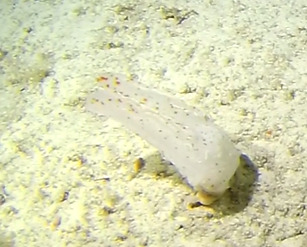
Aldabra W1, 250 m.

**Figure 120a.**
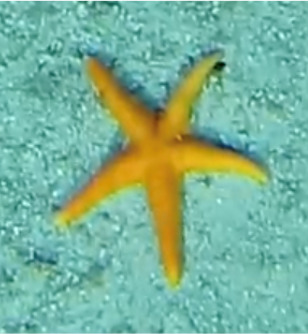
D'Arros N1, 350 m.

**Figure 120b.**
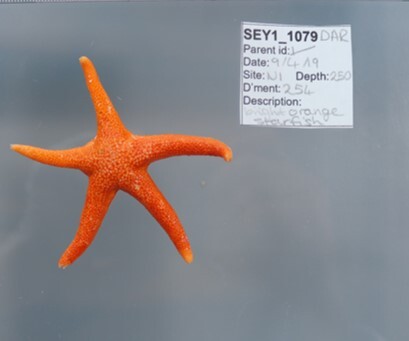
D'Arros N1, 350 m. Collected specimen SEY1_1079.

**Figure 121a.**
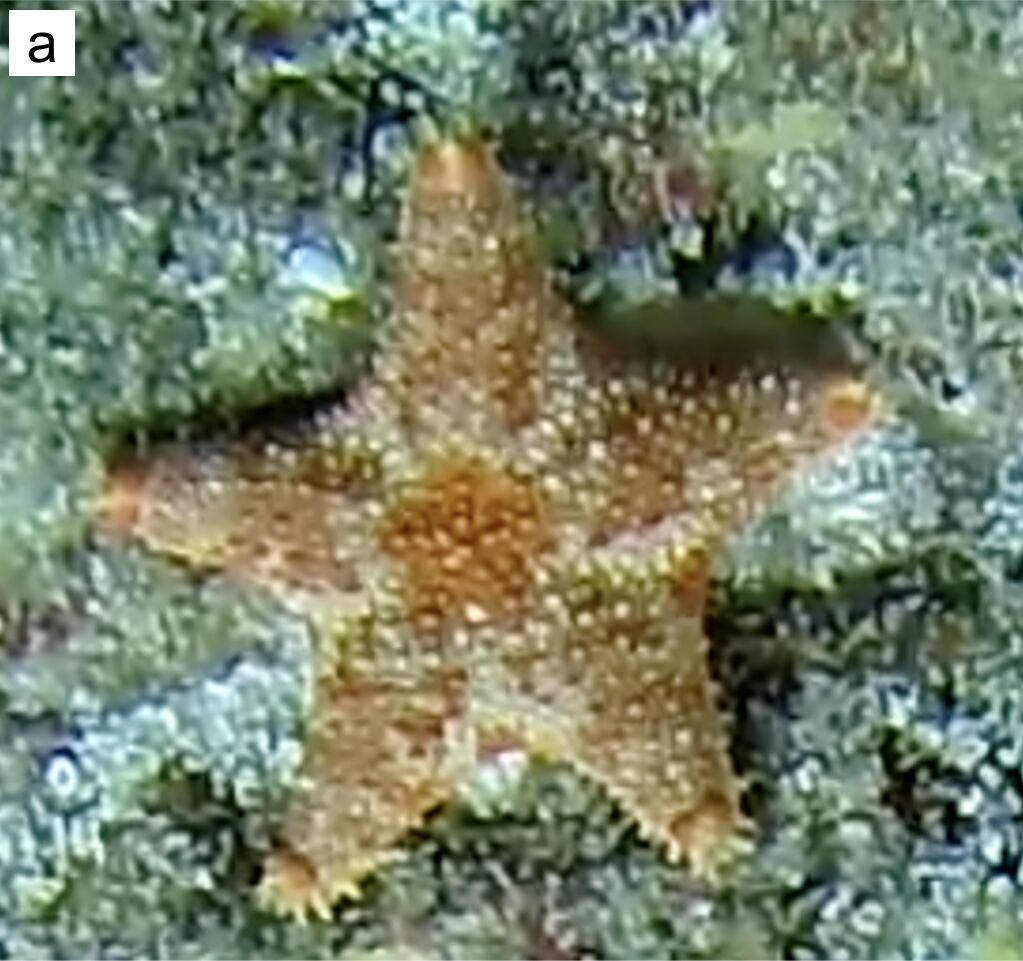
Poivre E1, 120 m.

**Figure 121b.**
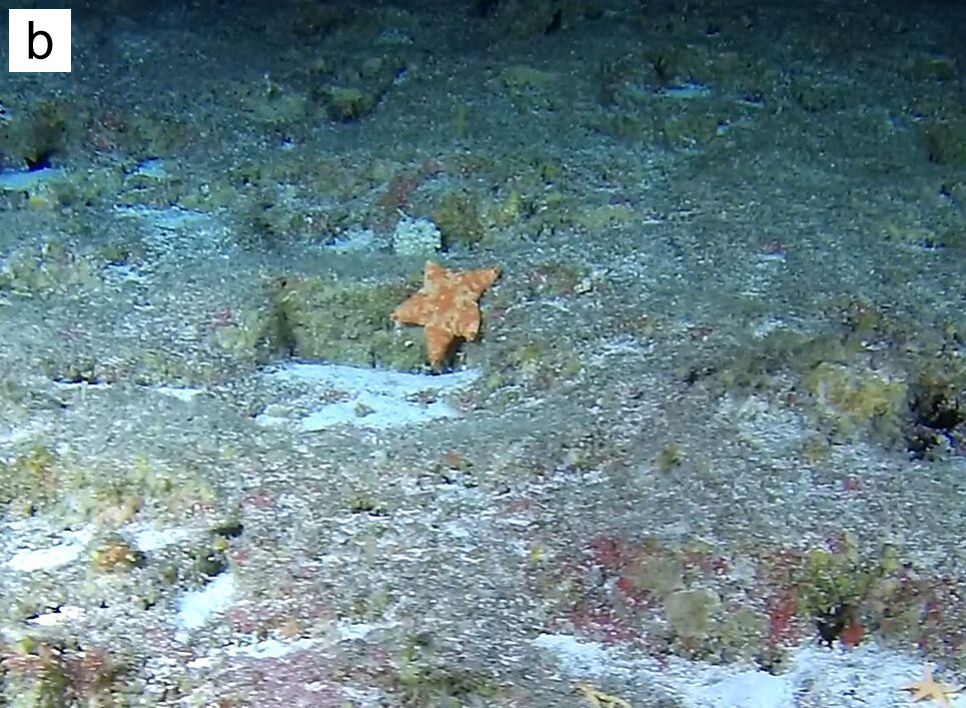
Poivre E1, 120 m.

**Figure 121c.**
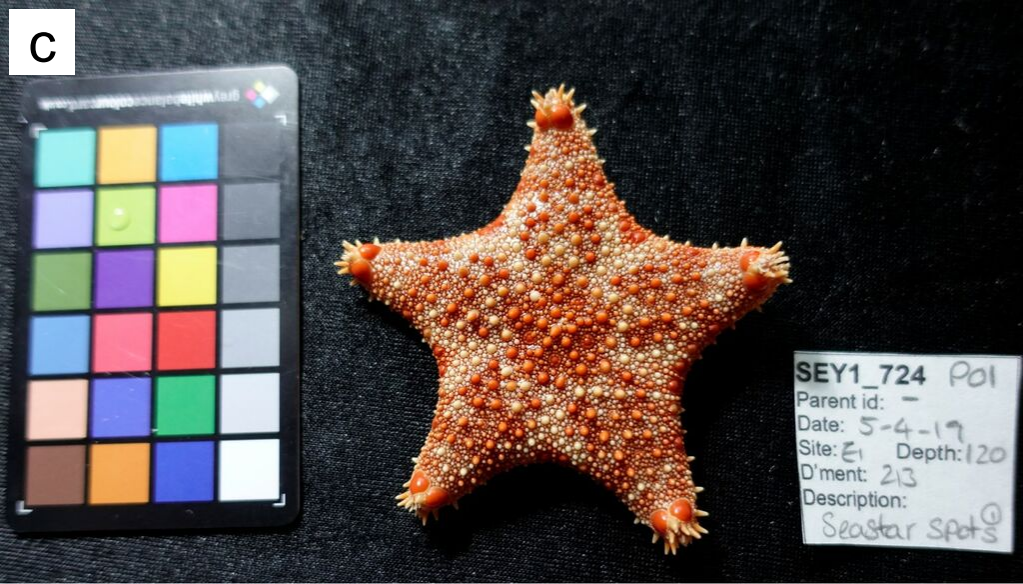
Poivre E1, 120 m. Collected specimen (SEY1_724)

**Figure 121d.**
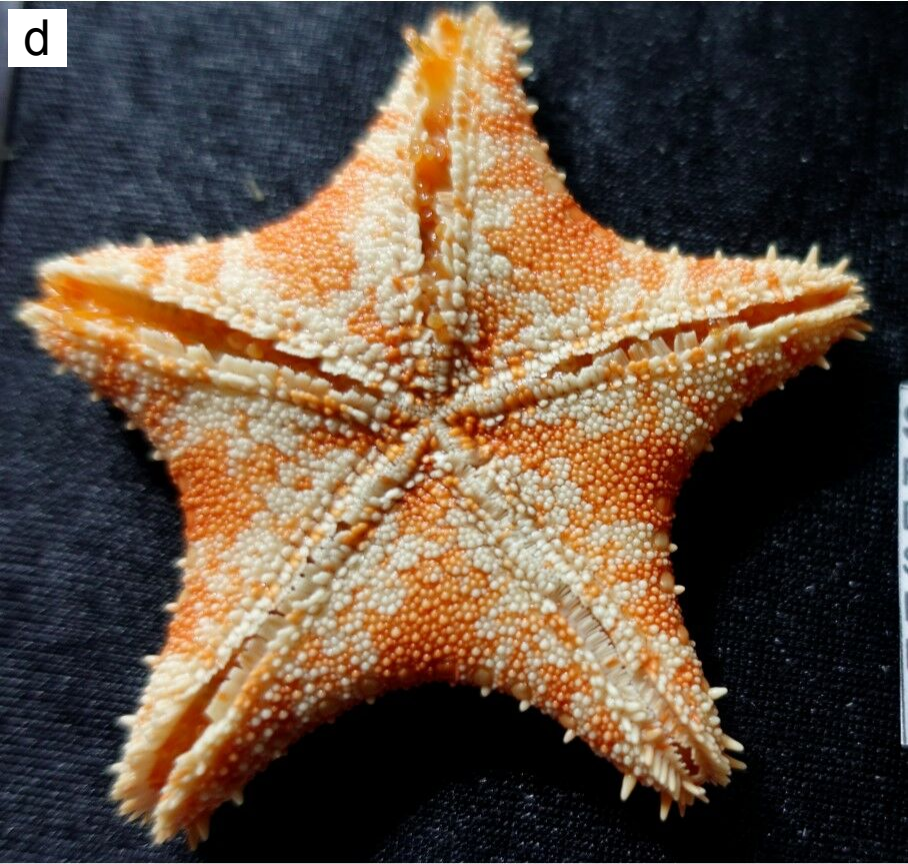
Poivre E1, 120 m. Back-side of collected specimen (SEY1_724)

**Figure 122a.**
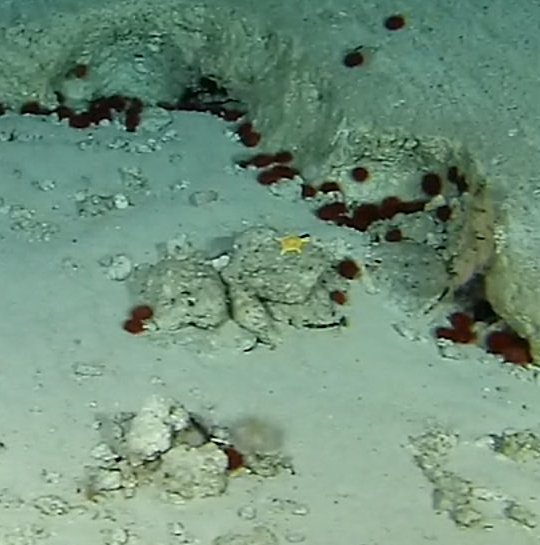
Alphonse N1, 252 m.

**Figure 122b.**
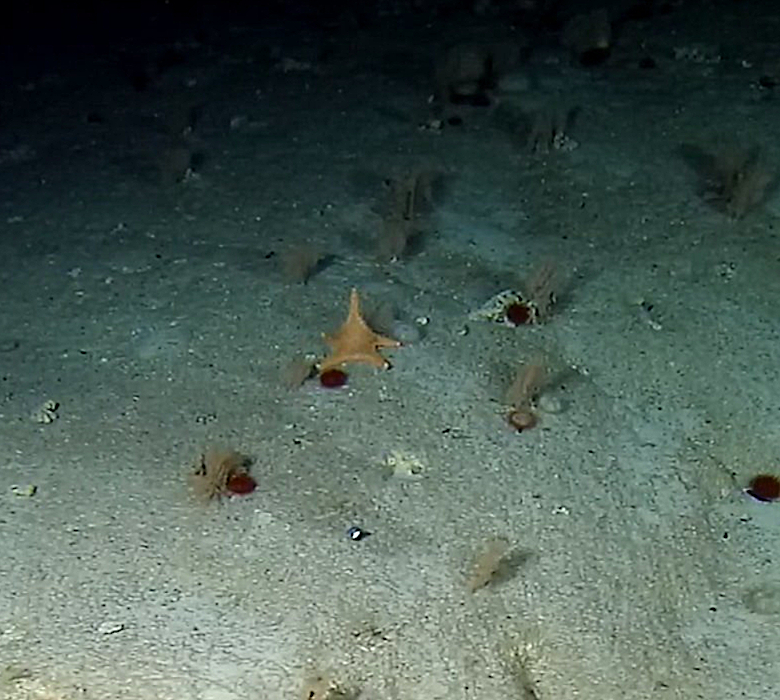
Alphonse N1, 238 m.

**Figure 124a.**
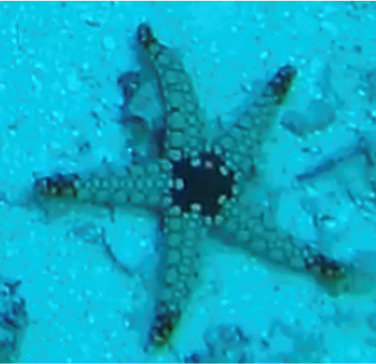
D'Arros N1, 30 m.

**Figure 124b.**
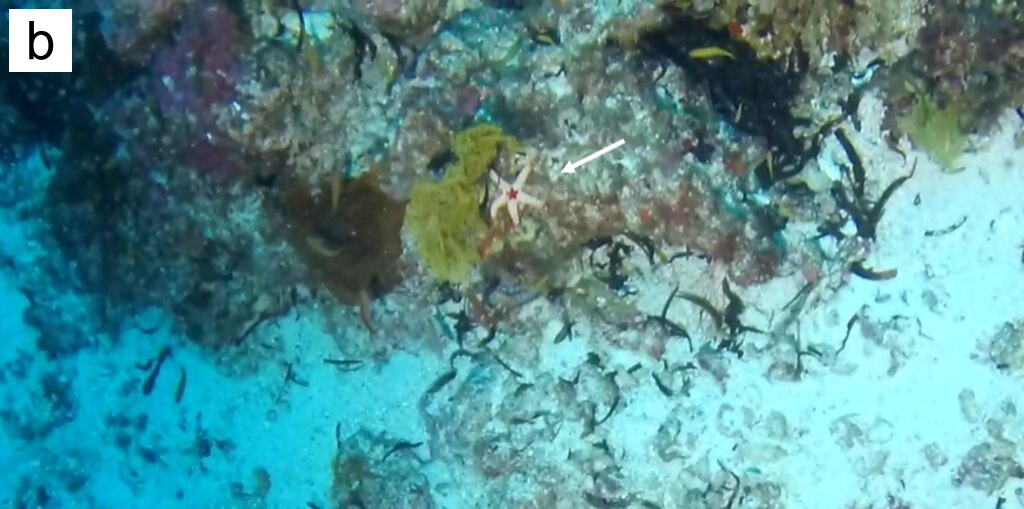
Alphonse N1, 60 m.

**Figure 124c.**
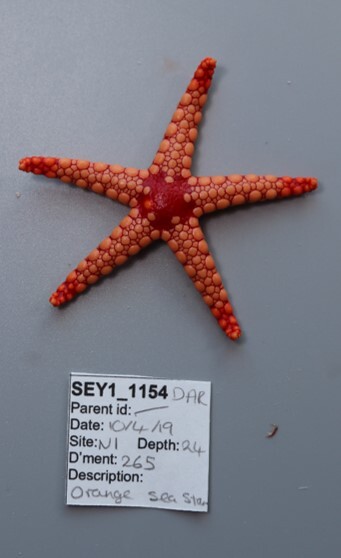
D'Arros N1, 30 m. Collected specimen (SEY1_1154)

**Figure 124d.**
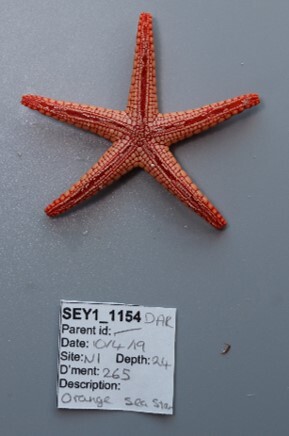
D'Arros N1, 30 m. Backside of collected specimen (SEY1_1154)

**Figure 125a.**
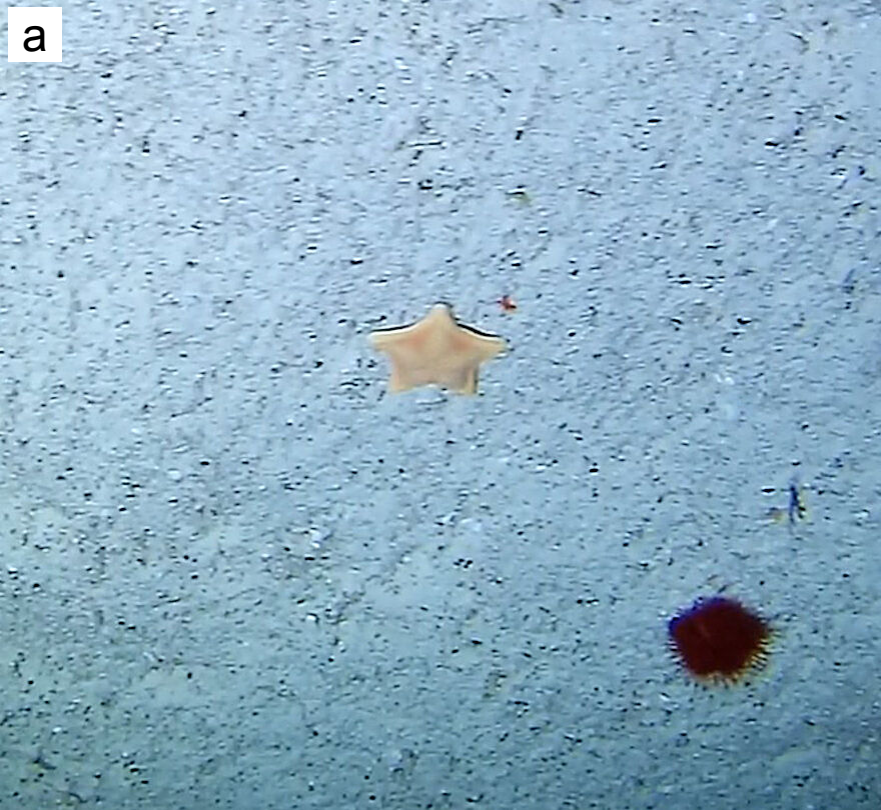
Alphonse N1, 250 m.

**Figure 125b.**
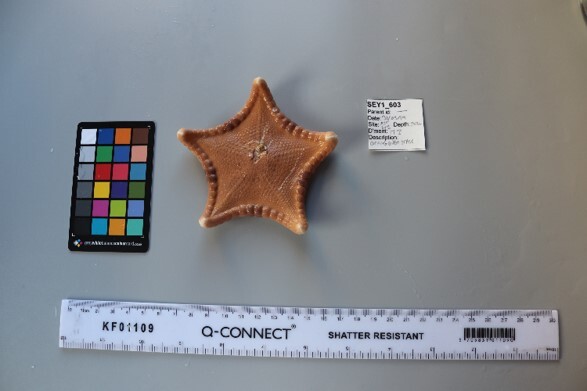
Astove W1, 250 m. Collected specimen (SEY1_603)

**Figure 125c.**
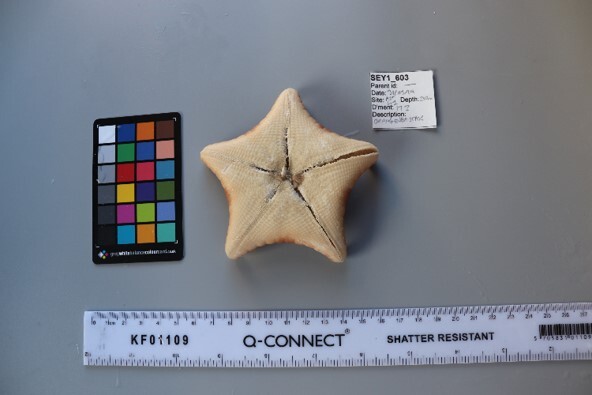
Astove W1, 250 m. Back-side of collected specimen (SEY1_603)

**Figure 126a.**
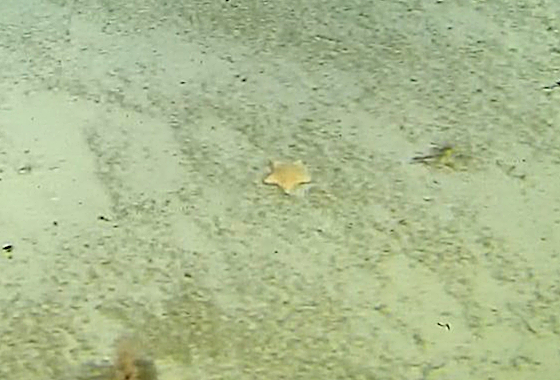
Alphonse N1, 255 m.

**Figure 126b.**
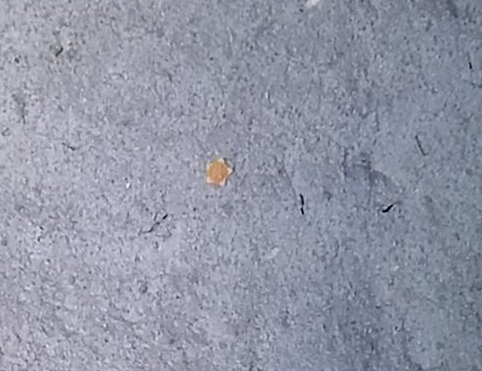
D'Arros N1, 350 m.

**Figure 127a.**
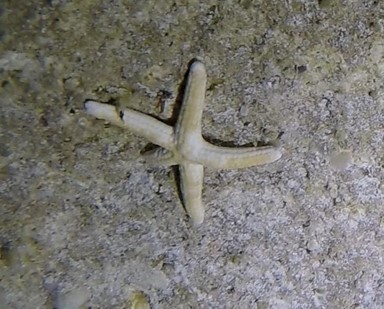
Astove W1, 250 m.

**Figure 127b.**
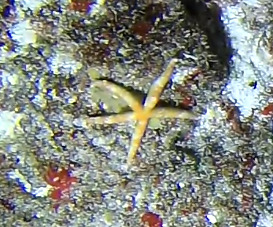
Poivre E1, 120 m.

**Figure 128a.**
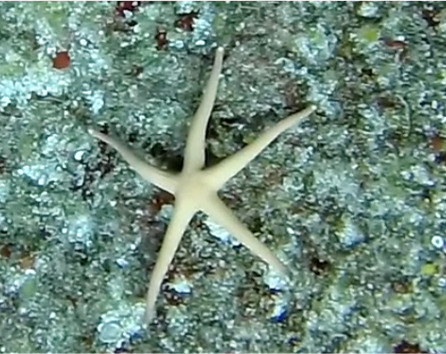
Poivre E1, 120 m.

**Figure 128b.**
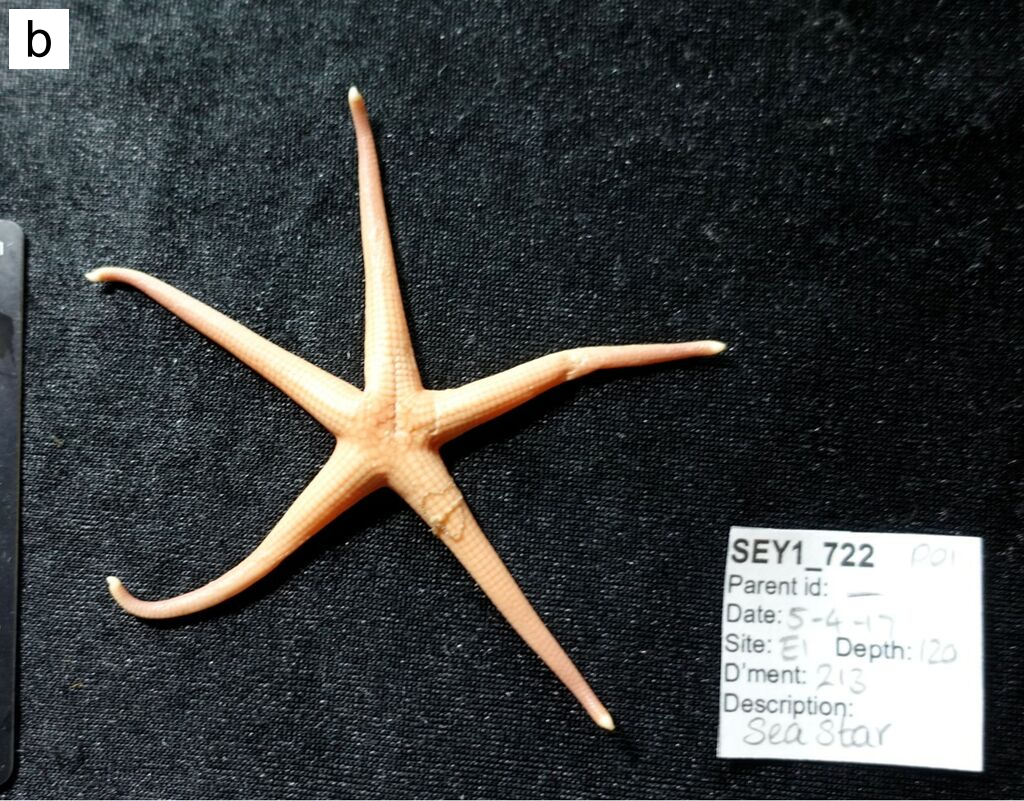
Poivre E1, 120 m. Collected specimen (SEY1_722).

**Figure 129a.**
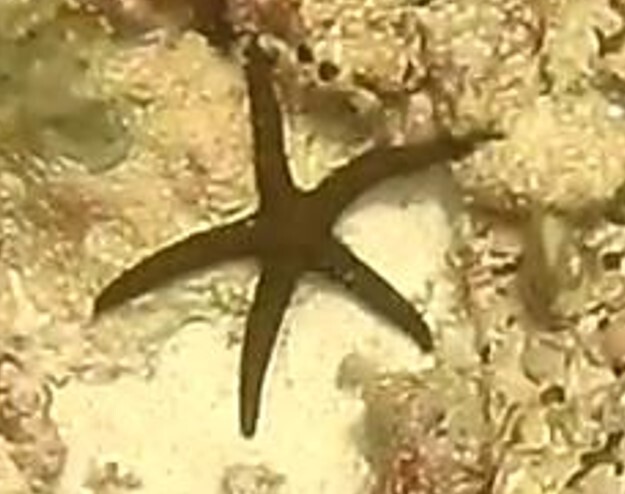
Poivre E1, 10 m.

**Figure 129b.**
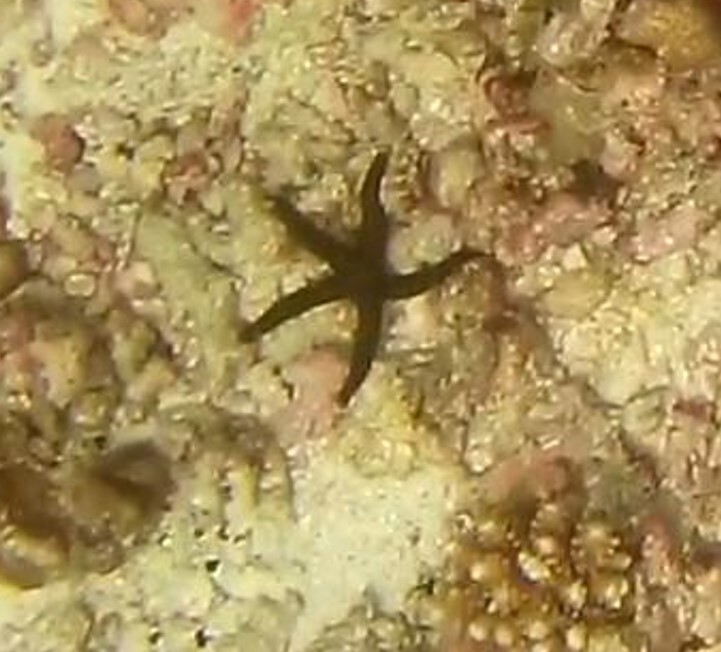
Poivre E1, 10 m.

**Figure 133a.**
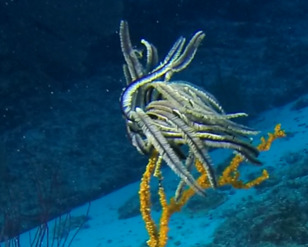
Aldabra N1, 60 m.

**Figure 133b.**
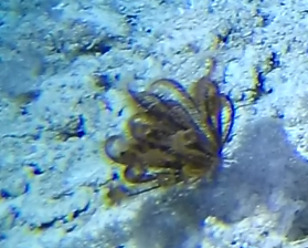
Aldabra W1, 250 m.

**Figure 133c.**
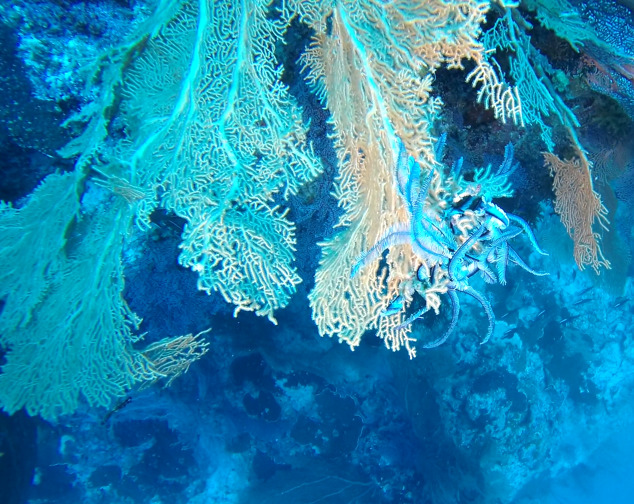
Aldabra N1, 30 m.

**Figure 133d.**
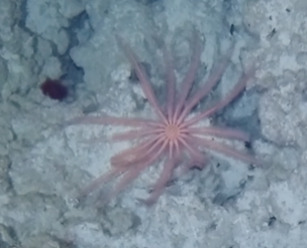
D'Arros N1, 350 m.

**Figure 134a.**
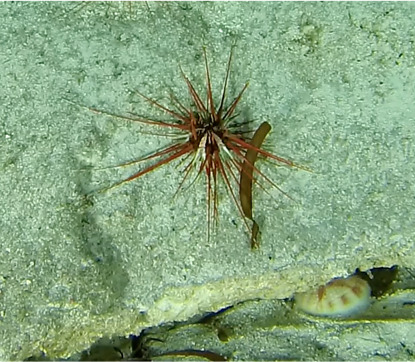
Desroches S1, 230 m.

**Figure 134b.**
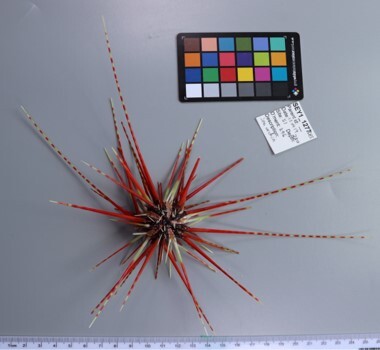
Desroches S1, 230 m. The collected specimen (SEY1_1277) has been identified as *Coelopleurusmaillardi*.

**Figure 136a.**
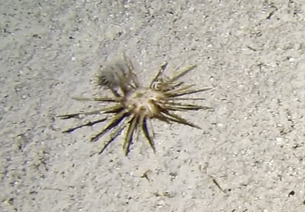
Alphonse N1, 250 m.

**Figure 136b.**
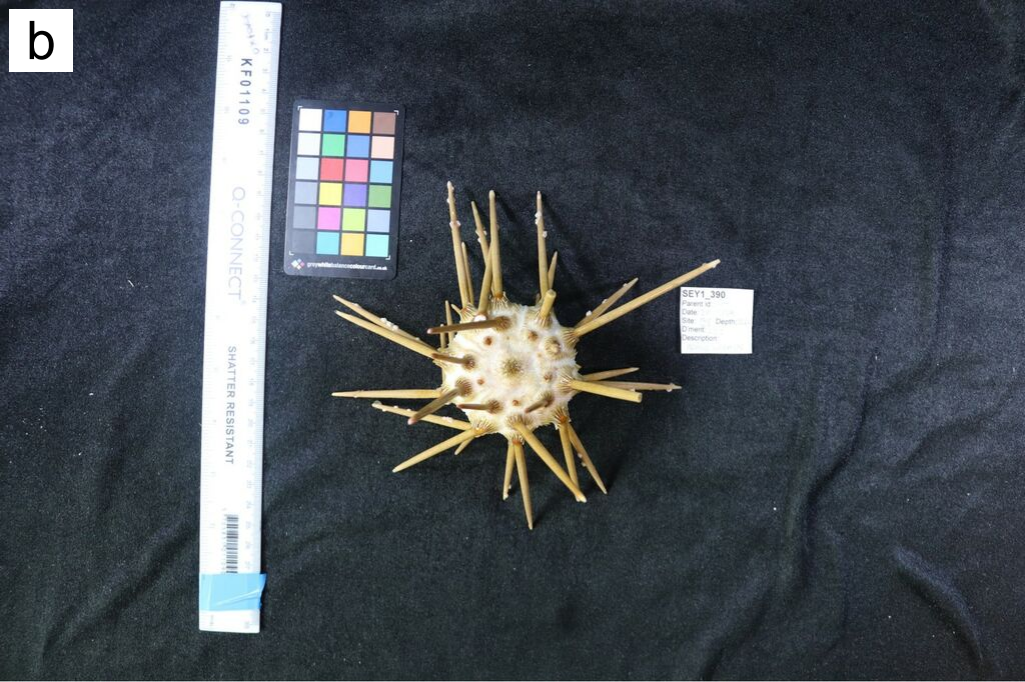
Aldabra W1, 250 m. The collected specimen (SEY1_390) has been identified as *Histocidaris* sp.

**Figure 138a.**
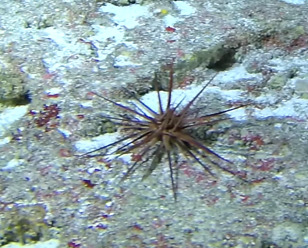
Poivre E1, 120 m.

**Figure 138b.**
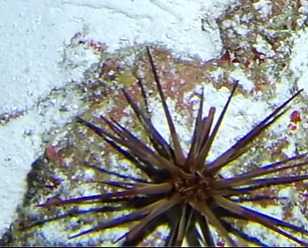
Poivre E1, 120 m.

**Figure 139a.**
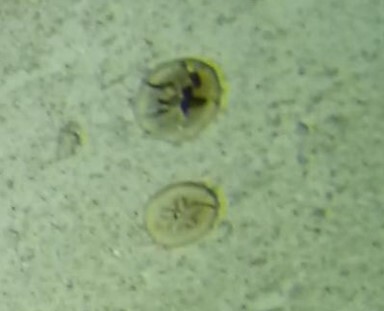
Desroches S1, 250 m.

**Figure 139b.**
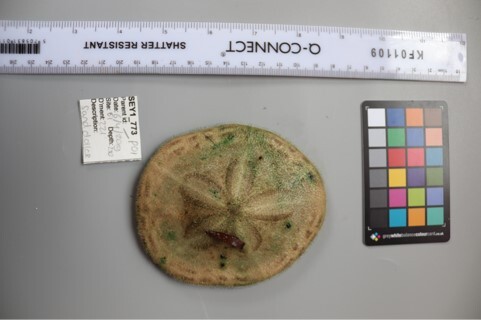
Poivre E1, 250 m. The collected specimen (SEY1_0773) was identified as *Clypeasterfervens*.

**Figure 141a.**
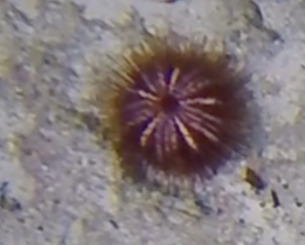
D'Arros N1, 350 m.

**Figure 141b.**
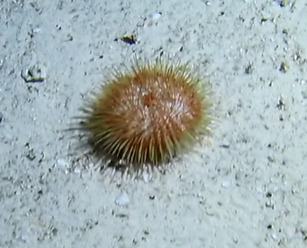
Alphonse N1, 250 m.

**Figure 141c.**
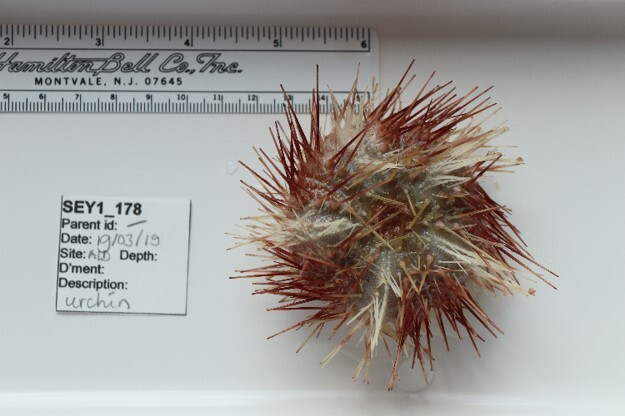
Aldabra N1, 250 m. The collected specimen (SEY1_178) was identified as Micropygacf.tuberculata.

**Figure 141d.**
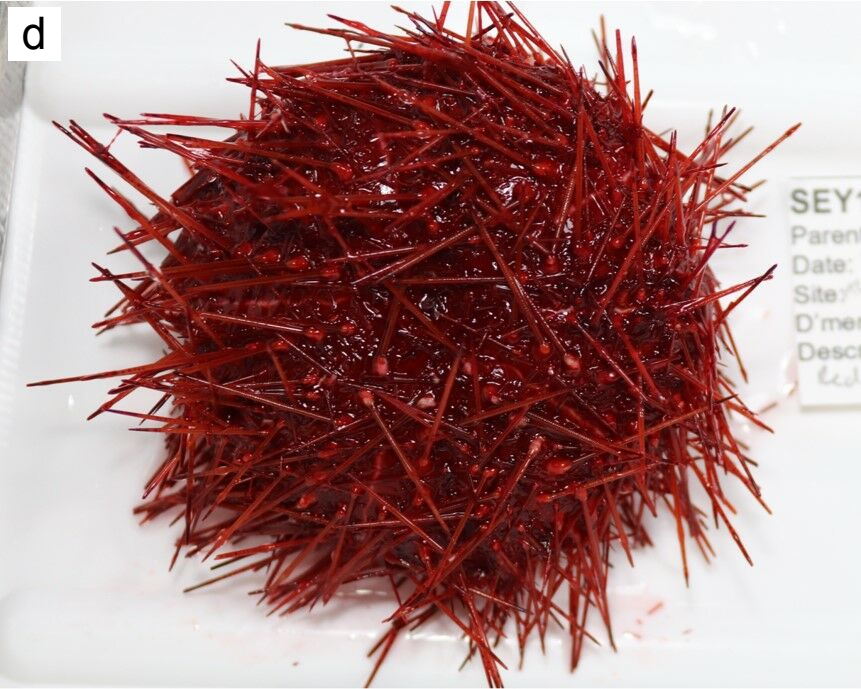
Alphonse N1, 250 m. The collected specimen (SEY1_122) was identified as Micropygacf.tuberculata.

**Figure 143a.**
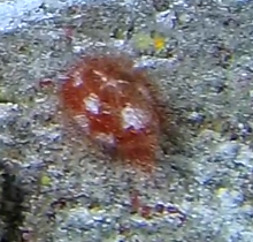
Poivre E1, 120 m.

**Figure 143b.**
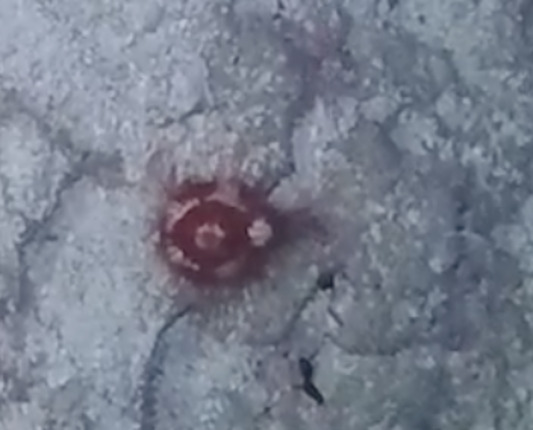
Astove W1, 350 m.

**Figure 145a.**
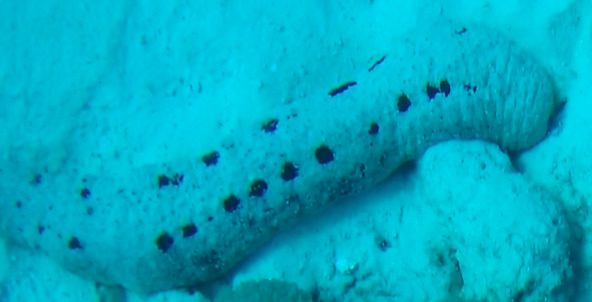
Desroches S1, 60 m.

**Figure 145b.**
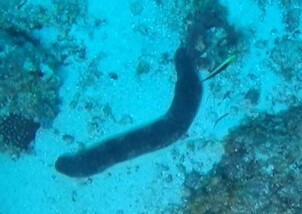
D'Arros N1, 30 m.

**Figure 146a.**
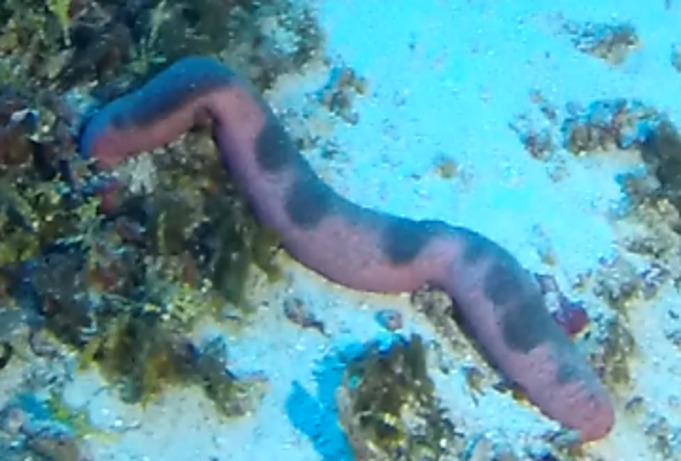
Astove W1, 60 m.

**Figure 146b.**
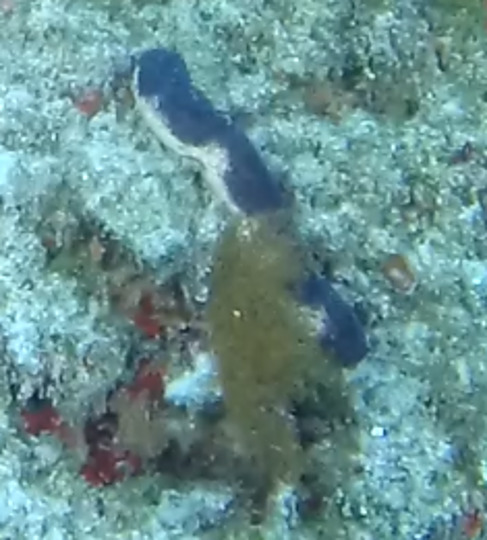
Astove W1, 60 m.

**Figure 152a.**
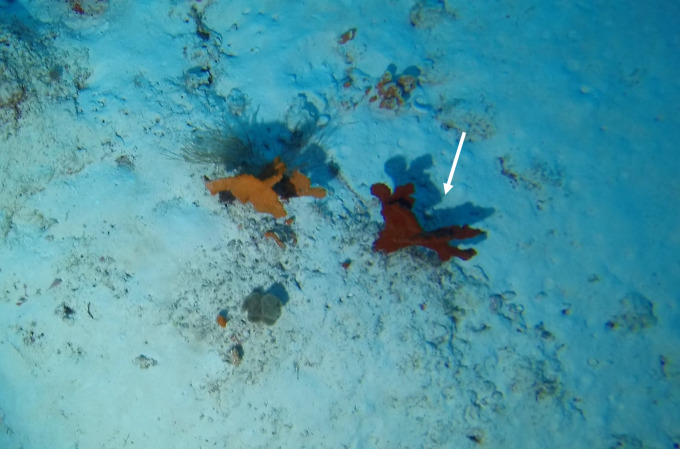
Aldabra W1, 30 m.

**Figure 152b.**
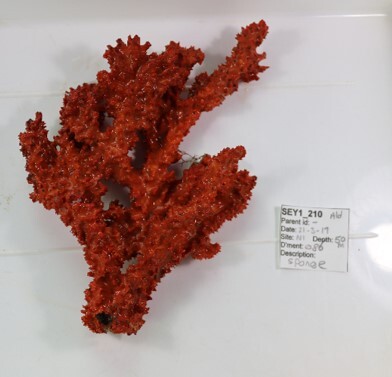
Aldabra N1, 30 m. Collected specimen (SEY1_210)

**Figure 153a.**
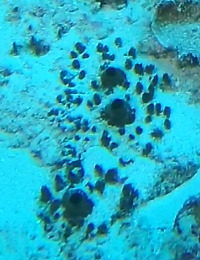
Aldabra N1, 30 m.

**Figure 153b.**
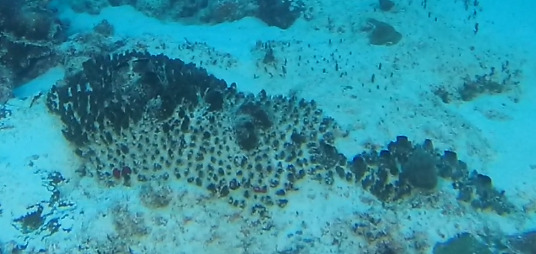
Aldabra W1, 30 m.

**Figure 154a.**
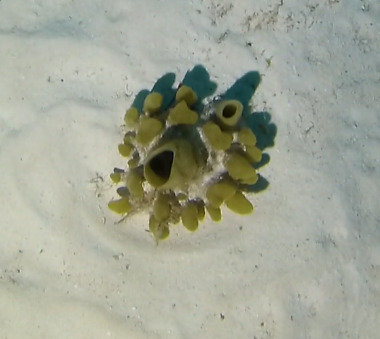
Aldabra W1, 60 m.

**Figure 154b.**
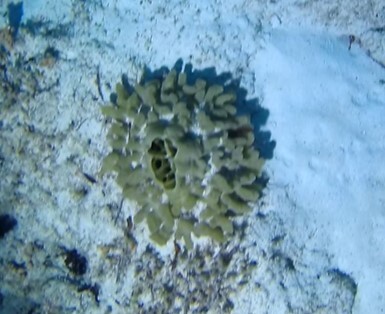
Aldabra W1, 60 m.

**Figure 154c.**
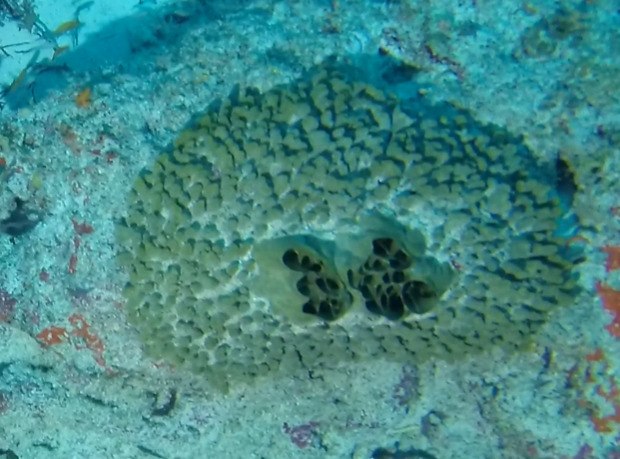
Aldabra W1, 60 m.

**Figure 155a.**
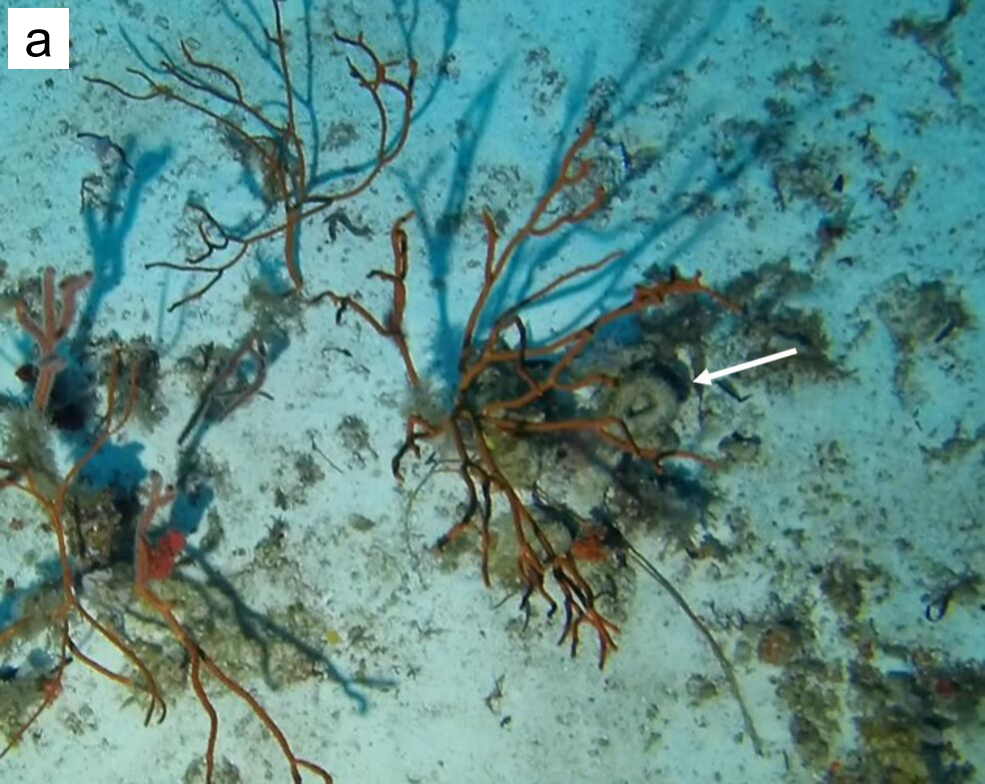
D'Arros N1, 60 m.

**Figure 155b.**
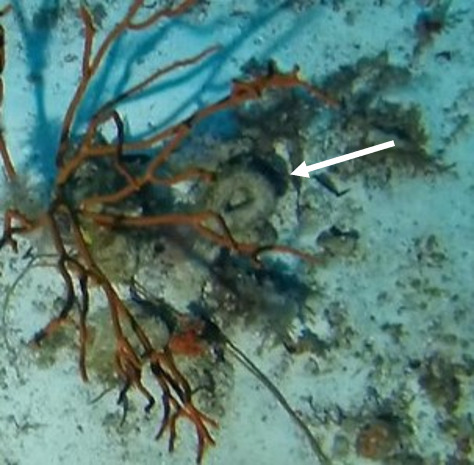
D'Arros N1, 60 m.

**Figure 156a.**
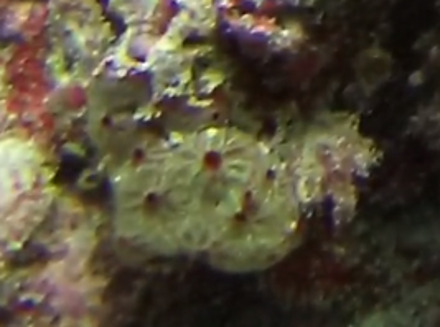
Aldabra N1, 10 m.

**Figure 156b.**
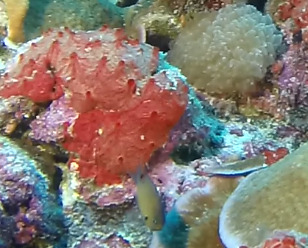
Aldabra N1, 30 m.

**Figure 156c.**
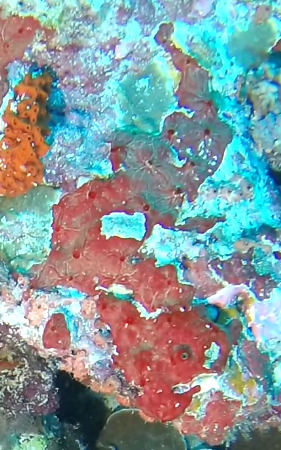
Aldabra N1, 60 m.

**Figure 157a.**
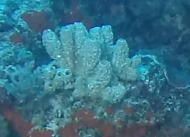
Aldabra W1, 30 m.

**Figure 157b.**
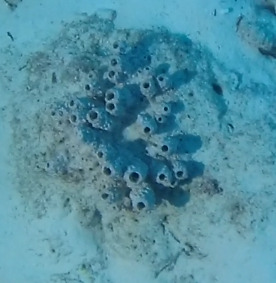
Aldabra W1, 30 m.

**Figure 158a.**
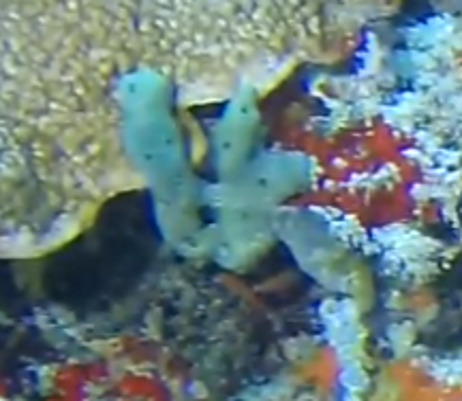
Alphonse N1, 60 m.

**Figure 158b.**
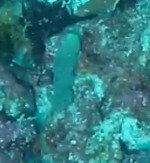
Alphonse N1, 60 m.

**Figure 159a.**
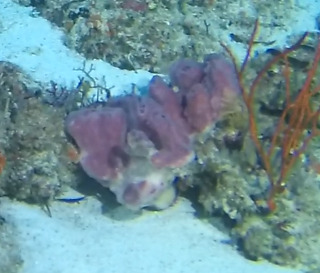
D'Arros N1, 60 m.

**Figure 159b.**
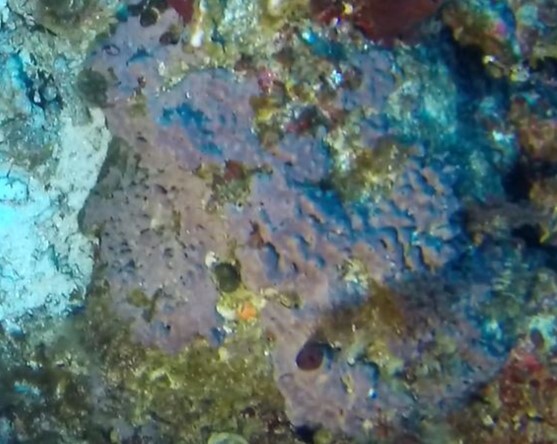
Astove W1, 60 m.

**Figure 159c.**
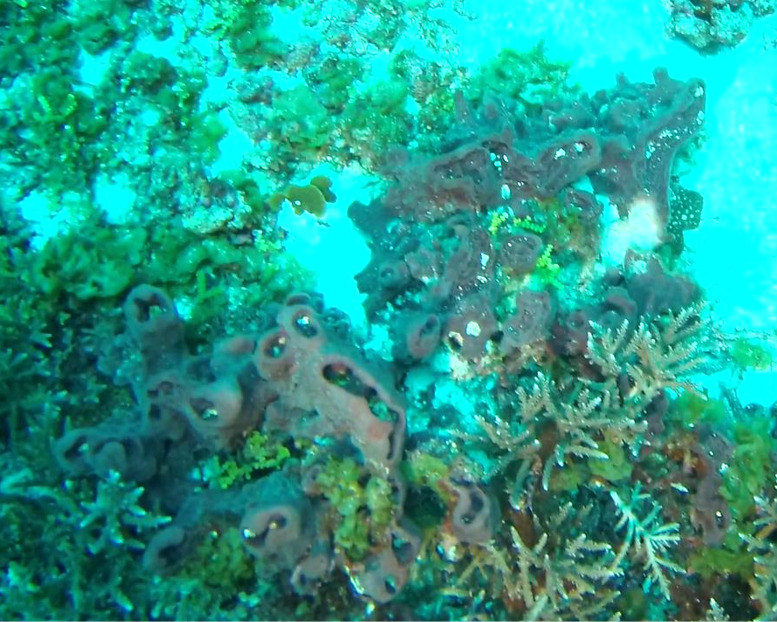
Poivre E1, 30 m.

**Figure 160a.**
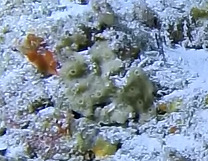
Aldabra W1, 120 m.

**Figure 160b.**
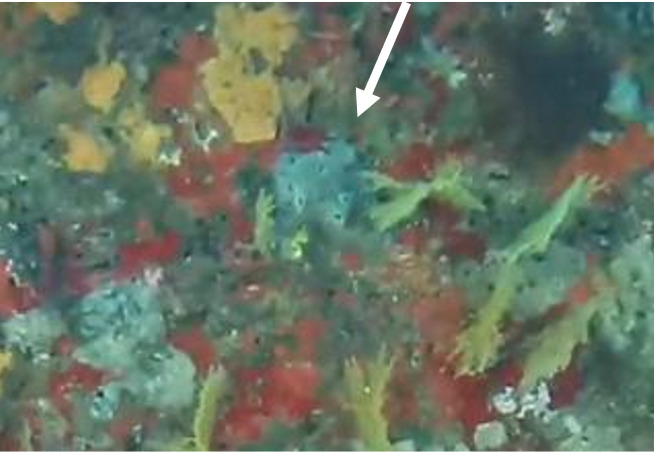
Alphonse N1, 60 m.

**Figure 160c.**
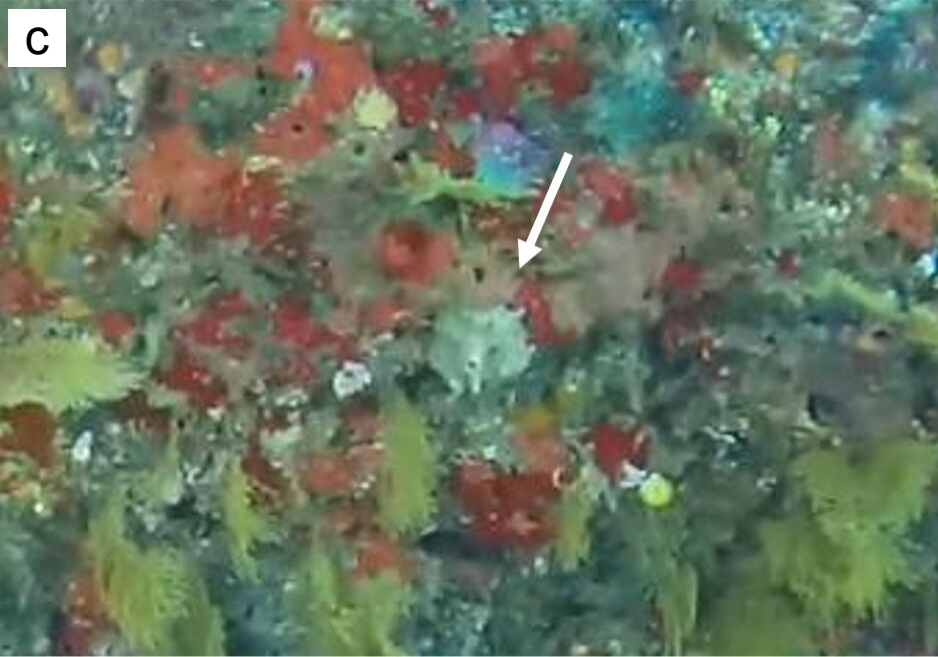
Alphonse N1, 60 m.

**Figure 162a.**
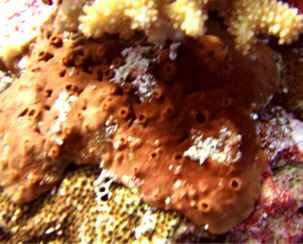
Astove W1, 10 m.

**Figure 162b.**
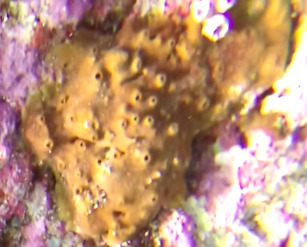
Astove W1, 10 m.

**Figure 163a.**
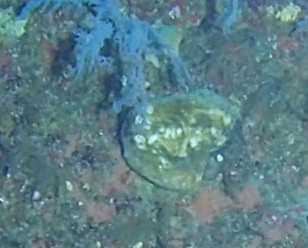
Astove W1, 60 m.

**Figure 163b.**
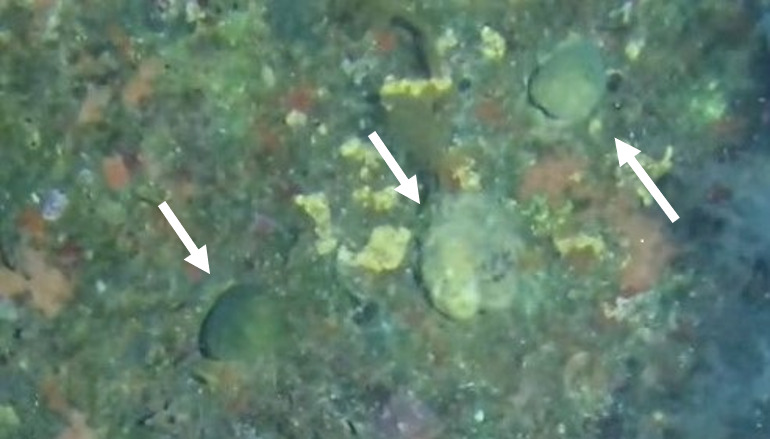
Astove W1, 60 m.

**Figure 164a.**
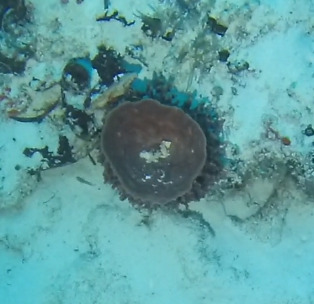
Aldabra W1, 30 m.

**Figure 164b.**
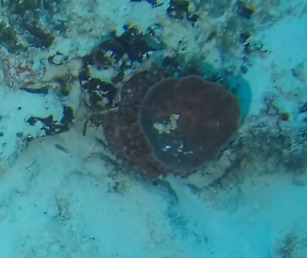
Aldabra W1, 30 m.

**Figure 165a.**
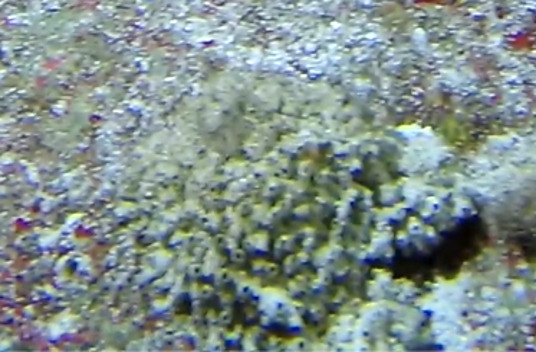
Poivre E1, 120 m.

**Figure 165b.**
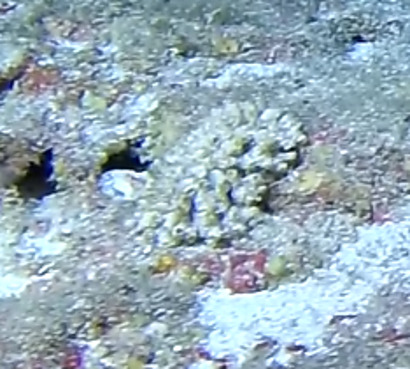
Poivre E1, 120 m.

**Figure 166a.**
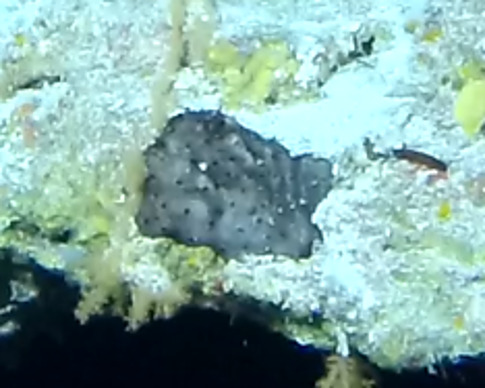
Aldabra N1, 120 m.

**Figure 166b.**
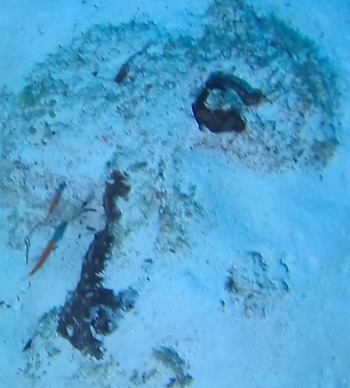
Aldabra W1, 60 m.

**Figure 166c.**
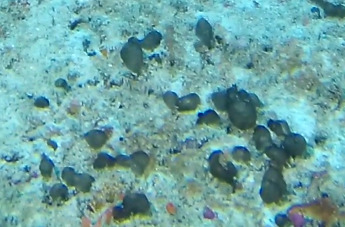
Aldabra W1, 60 m.

**Figure 167a.**
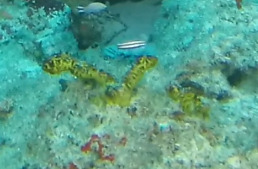
Aldabra W1, 60 m.

**Figure 167b.**
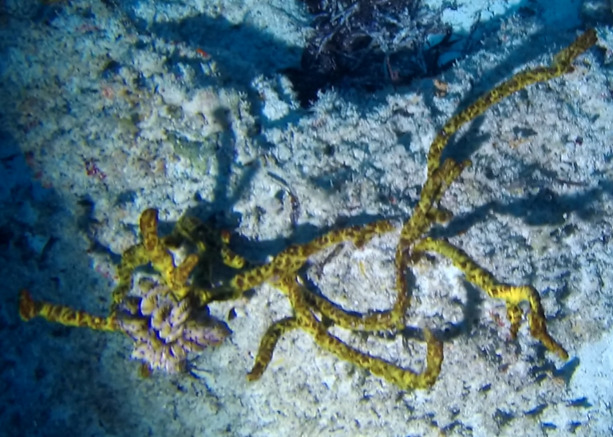
Aldabra W1, 60 m.

**Figure 168a.**
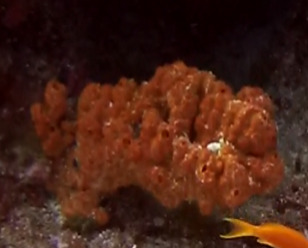
Astove W1, 10 m.

**Figure 168b.**
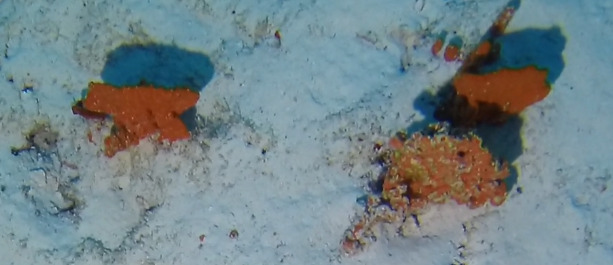
Aldabra W1, 30 m.

**Figure 168c.**
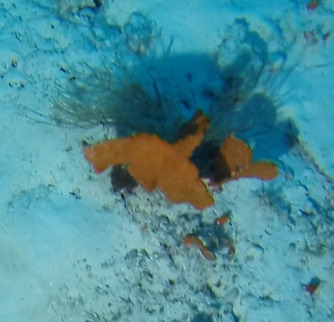
Aldabra W1, 30 m.

**Figure 168d.**
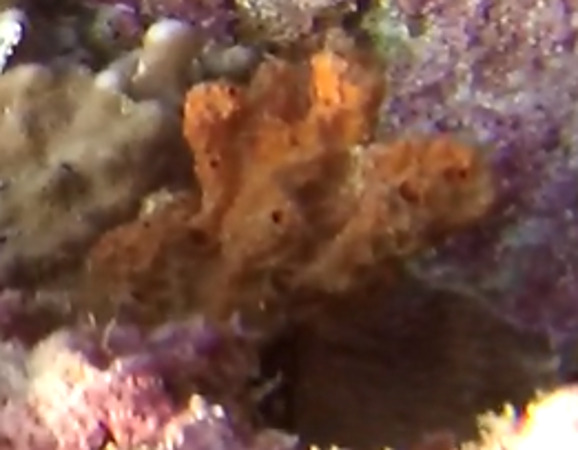
Astove W1, 10 m.

**Figure 169a.**
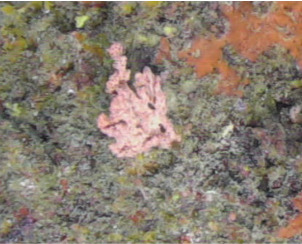
Aldabra N1, 30 m.

**Figure 169b.**
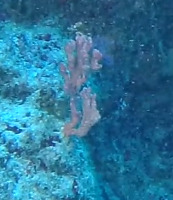
Aldabra N1 60 m.

**Figure 169c.**
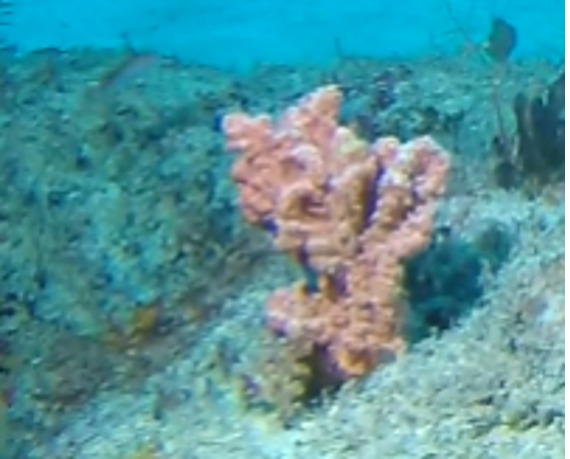
Aldabra W1, 60 m.

**Figure 169d.**
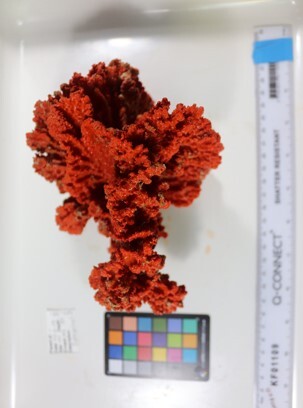
Aldabra W1, 60 m. Collected specimen (SEY1_351)

**Figure 170a.**
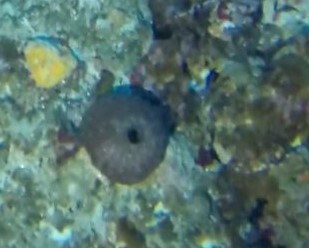
Astove W1, 60 m.

**Figure 170b.**
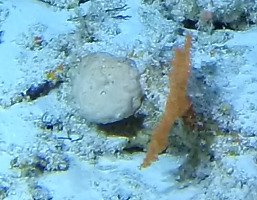
Aldabra W1, 120 m.

**Figure 171a.**
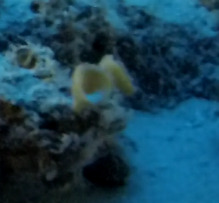
Aldabra N1, 160 m.

**Figure 171b.**
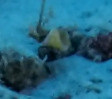
Aldabra N1, 160 m.

**Figure 171c.**
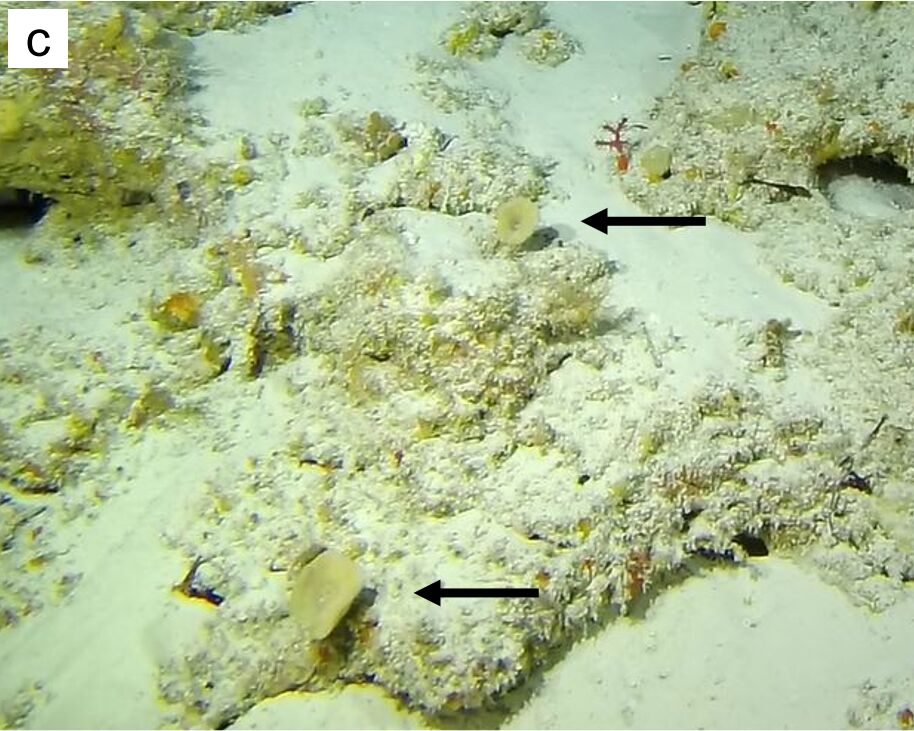
Aldabra W1, 120 m.

**Figure 172a.**
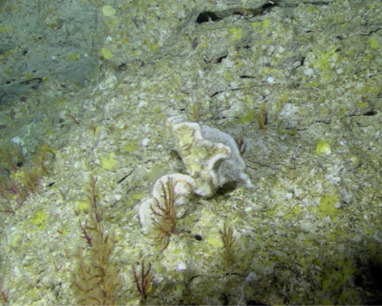
Aldabra W1, 120 m.

**Figure 172b.**
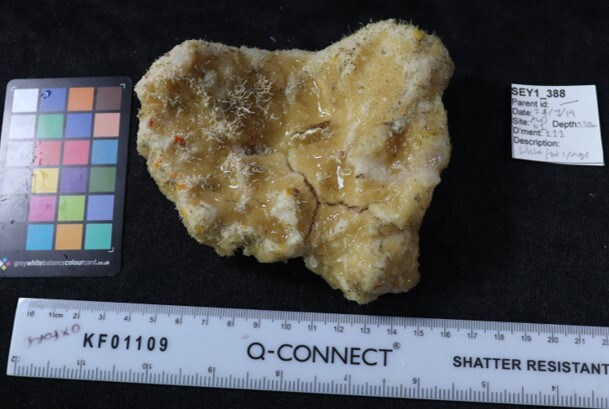
Aldabra W1, 120 m. Collected specimen SEY1_388.

**Figure 175a.**
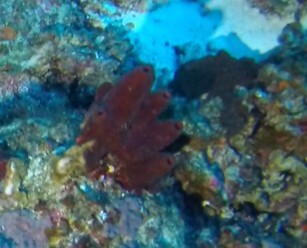
Astove W1, 60 m.

**Figure 175b.**
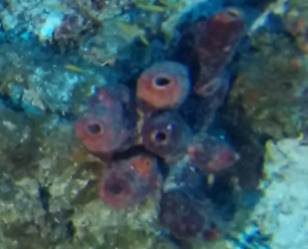
Astove W1, 60 m.

**Figure 175c.**
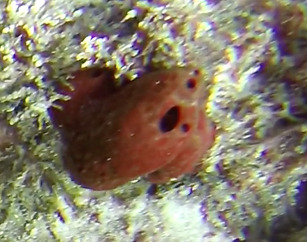
Aldabra N1, 10 m.

**Figure 176a.**
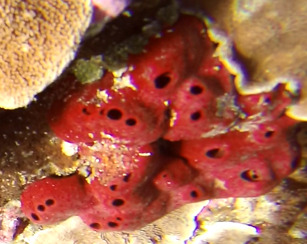
Astove W1, 10 m.

**Figure 176b.**
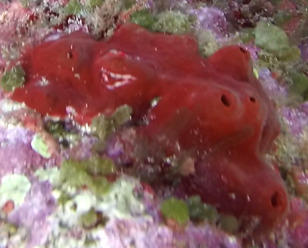
Astove W1, 10 m.

**Figure 177a.**
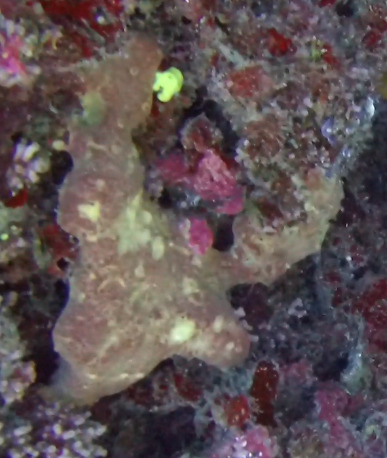
Astove W1, 10 m.

**Figure 177b.**
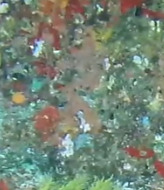
Alphonse N1, 60 m.

**Figure 178a.**
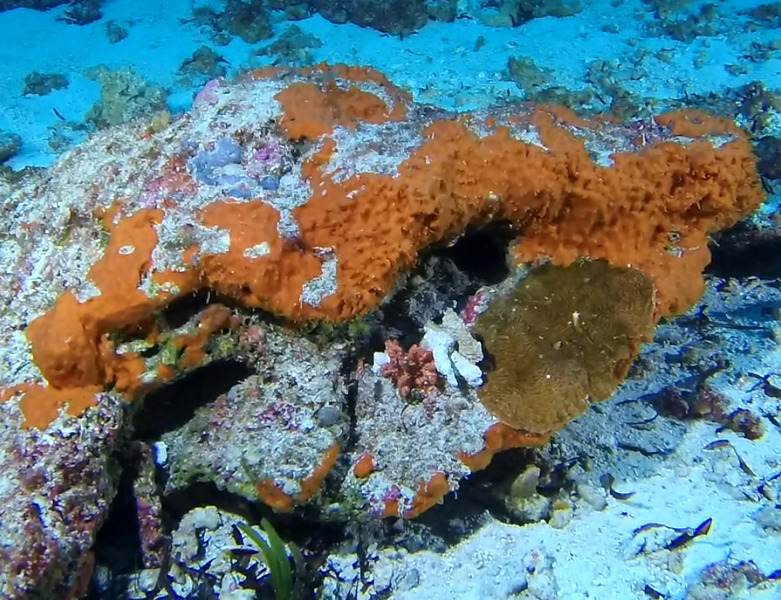
*Biemna* sp. Alphonse N1, 63 m.

**Figure 178b.**
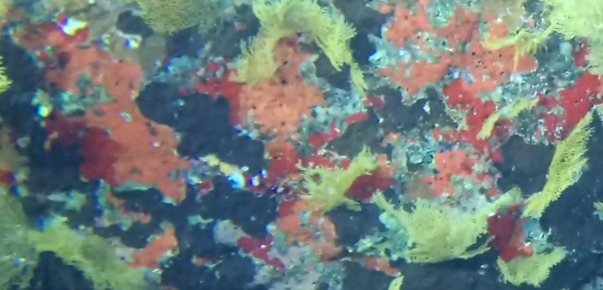
*Petrosia* sp. Alphonse N1, 60 m.

**Figure 178c.**
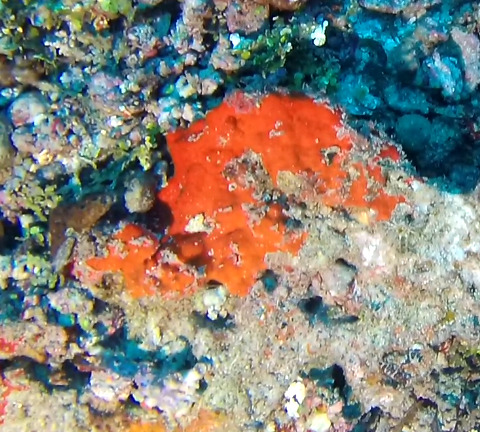
*Clathria* sp. Desroches S1 , 30 m.

**Figure 179a.**
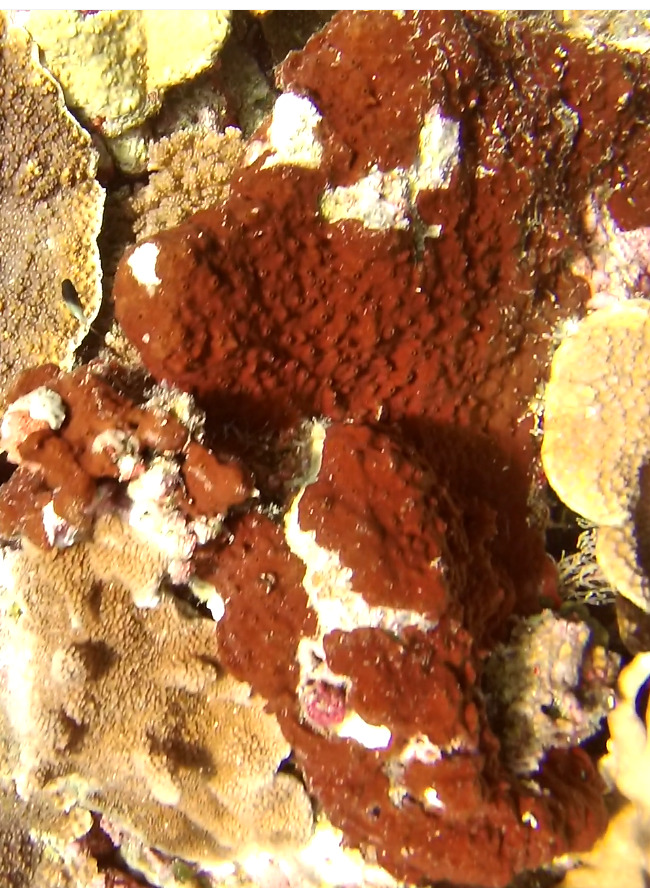
*Raspailia* sp. Astove W1. 10 m.

**Figure 179b.**
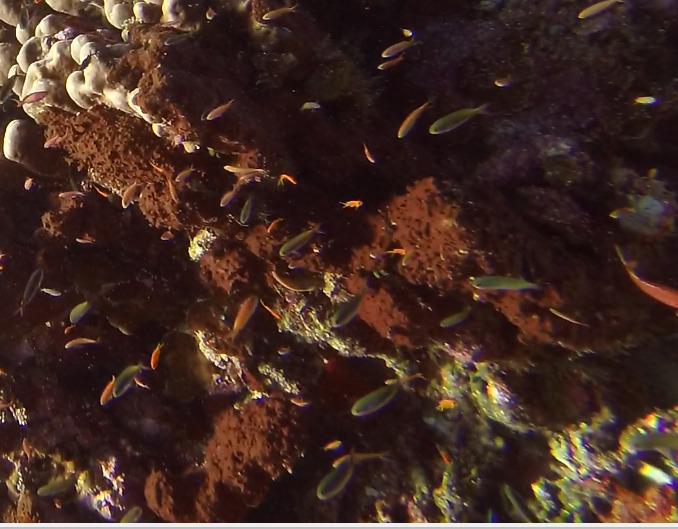
*Raspailia* sp. Astove W1, 10 m.

**Figure 179c.**
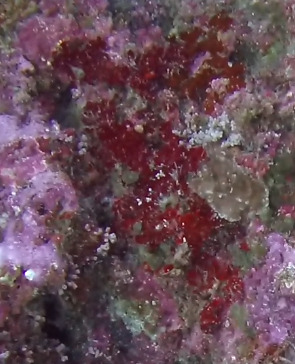
*Cliona* sp. Astove W1, 10 m.

**Figure 179d.**
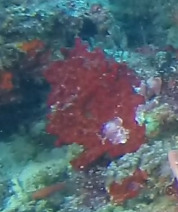
*Cliona* sp. Aldabra N1, 30 m.

**Figure 179e.**
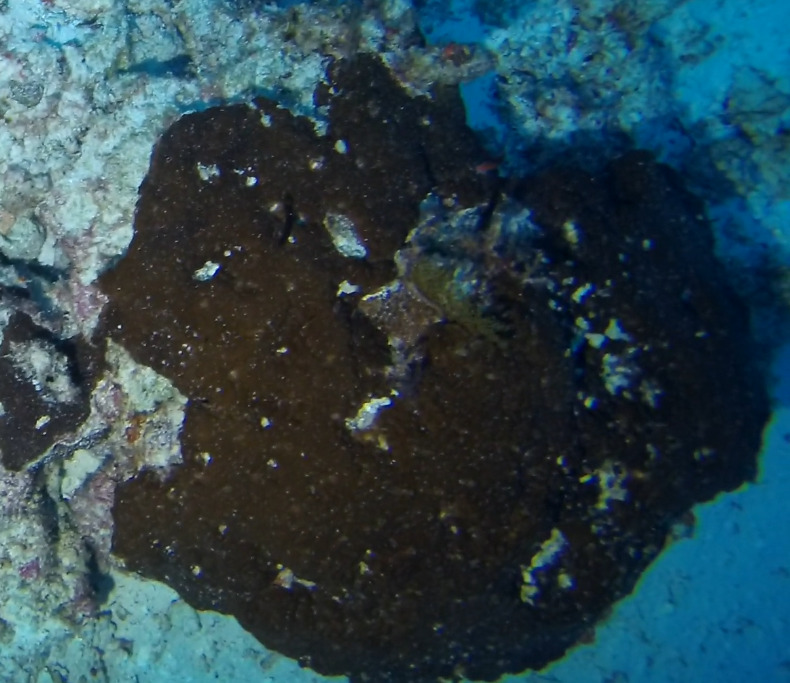
*Spirastrella* sp. Aldabra N1, 60 m.

**Figure 179f.**
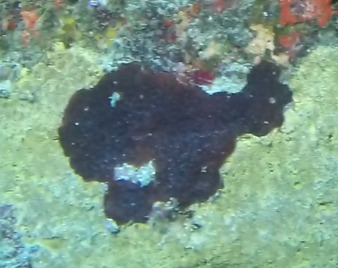
*Spirastrella* sp. Aldabra N1, 60 m.

**Figure 180a.**
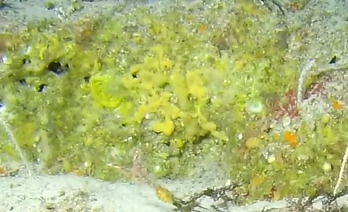
*Haliclona* sp. Aldabra N1, 120 m.

**Figure 180b.**
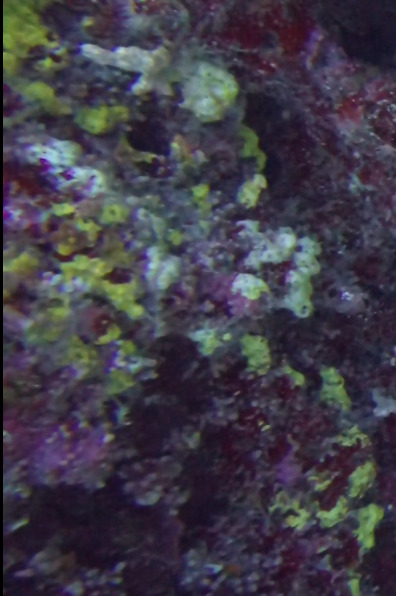
*Cliona* sp. Astove W1, 10 m.

**Figure 180c.**
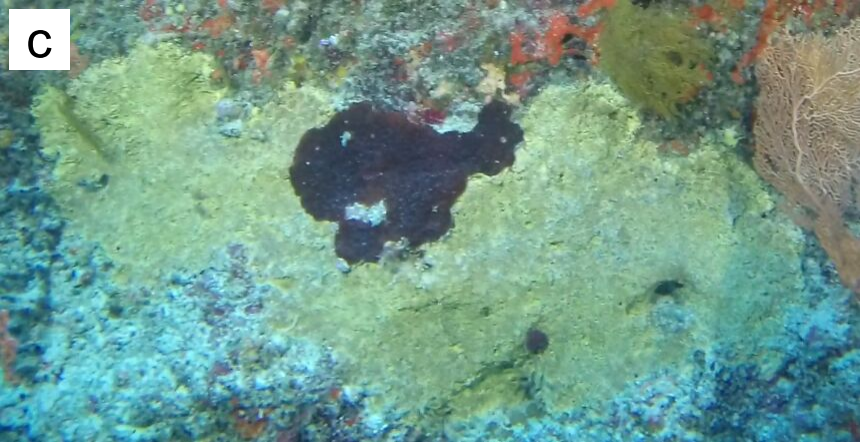
*Cliona* sp. Aldabra N1, 60 m.

**Figure 182a.**
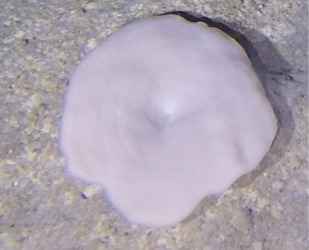
Astove W1, 120 m.

**Figure 182b.**
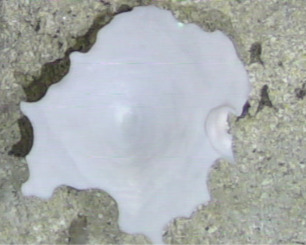
Aldabra N1, 120 m.

**Figure 182c.**
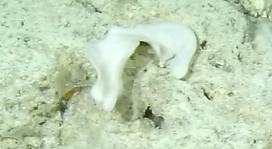
Aldabra W1, 250 m.

**Figure 183a.**
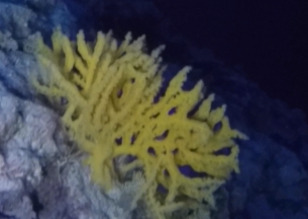
Astove W1, 250 m.

**Figure 183b.**
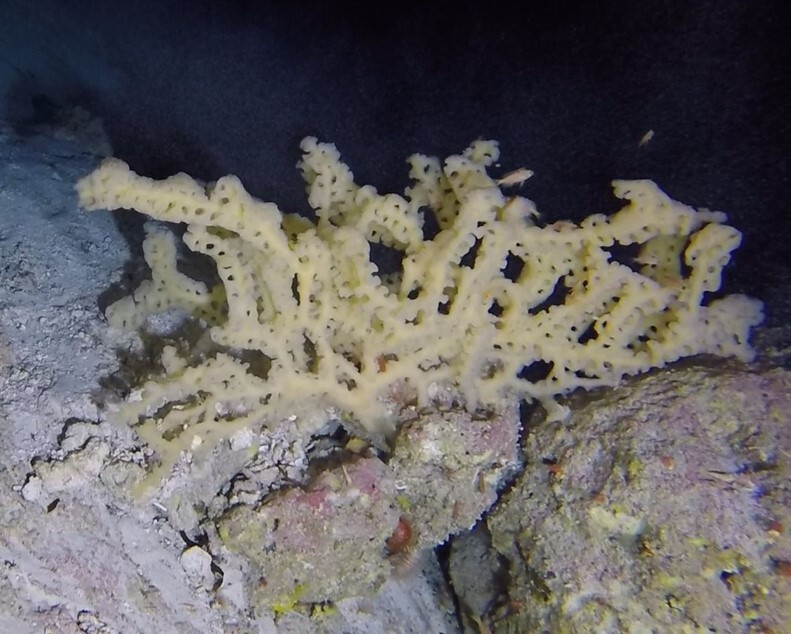
Astove W1, 250 m.

**Figure 183c.**
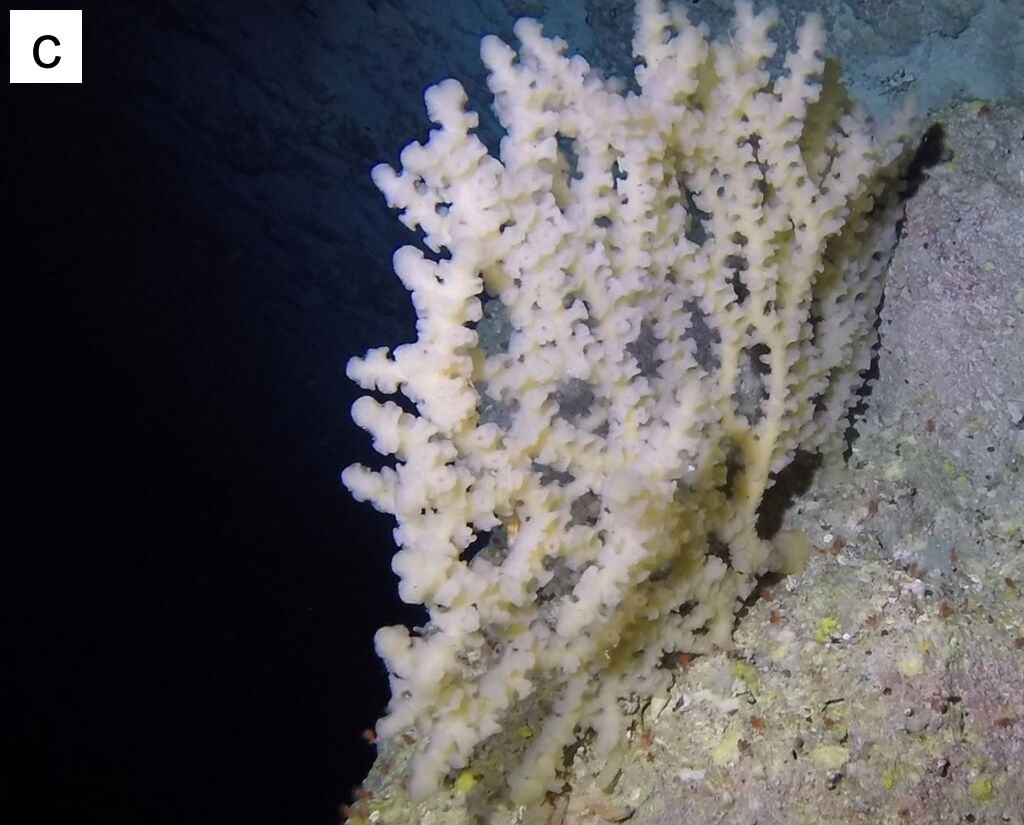
Astove W1, 250 m.

**Figure 183d.**
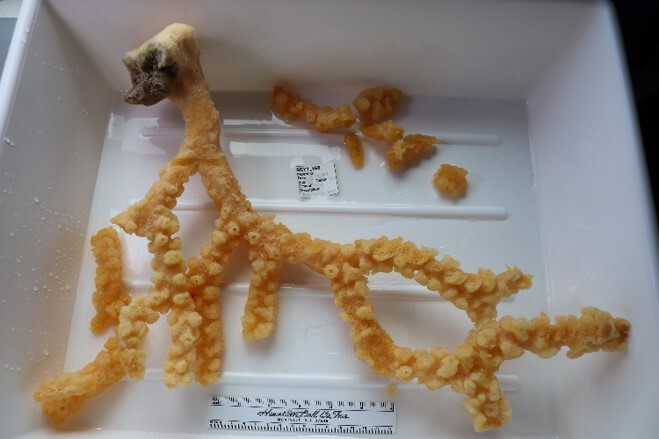
Aldabra W1, 120 m. Collected specimen (SEY1_163).

**Figure 184a.**
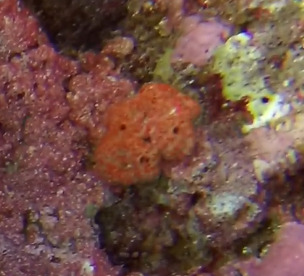
Astove W1, 10 m.

**Figure 184b.**
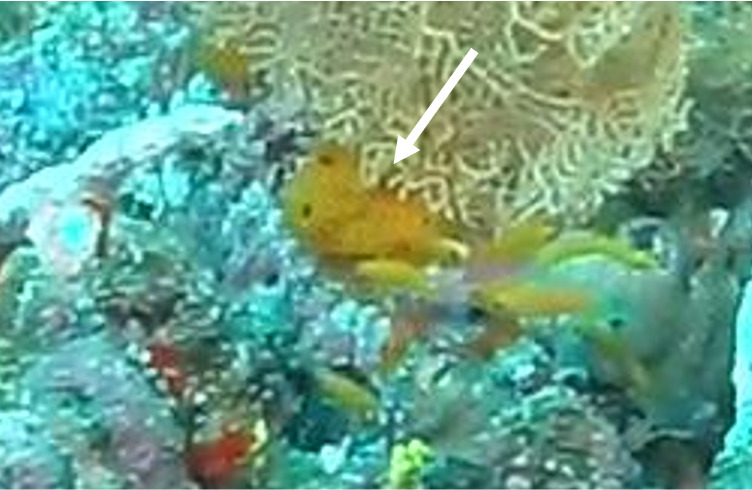
Alphonse N1, 30 m.

**Figure 184c.**
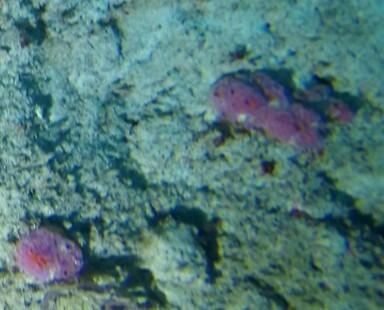
Aldabra W1, 60 m.

**Figure 185a.**
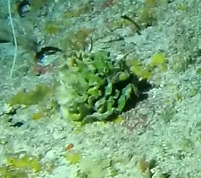
Aldabra N1, 120 m.

**Figure 185b.**
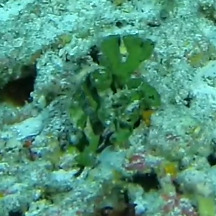
Aldabra N1, 120 m.

**Table 1.** List of the 184 morphotypes observed in shallow and deeper reef habitats in the Seychelles during the First Descent: Seychelles 2019 expedition. Open nomenclature (ON) signs applicable to image-based faunal analyses (e.g. indet., stet., inc.), as suggested by Horton et al. (2021), are also provided in the cases where species-level identification was not possible.

Phylum	Class	Order	Family	Genus	Species / Morphospecies Scientific Name with ON signs
Chlorophyta	Ulvophyceae	Bryopsidales	Caulerpaceae	* Caulerpa *	*Caulerpa* sp. indet. 1
Chlorophyta	Ulvophyceae	Bryopsidales	Caulerpaceae	* Caulerpa *	*Caulerpa* sp. indet. 2
Chlorophyta	Ulvophyceae	Bryopsidales	Codiaceae	* Codium *	*Codium* sp. indet.
Chlorophyta	Ulvophyceae	Bryopsidales	Halimedaceae	* Halimeda *	*Halimeda* spp. indet.
Chlorophyta	Ulvophyceae	Bryopsidales	Udoteaceae	* Udotea *	*Udotea* spp. indet.
Chlorophyta	Ulvophyceae	Cladophorales	Anadyomenaceae	* Microdictyon *	*Microdictyon* sp. indet.
Chlorophyta	Ulvophyceae	Cladophorales	Siphonocladaceae	* Dictyosphaeria *	*Dictyosphaeria* sp. indet.
Chlorophyta	Ulvophyceae	Ulvales	Ulvaceae	* Ulva *	*Ulva* sp. indet.
Ochrophyta	Phaeophyceae	Dictyotales	Dictyotaceae	* Lobophora *	*Lobophora* sp. indet.
Rhodophyta	Florideophyceae	Ceramiales	Dasyaceae	* Amphisbetema *	* Amphisbetema indica *
Rhodophyta	Florideophyceae	Corallinales			Corallinales stet.
Tracheophyta	Tracheophyta	Alismatales	Cymodoceaceae	* Thalassodendron *	* Thalassodendron ciliatum *
Tracheophyta	Tracheophyta	Alismatales	Hydrocharitaceae	* Halophila *	*Halophila* sp. indet
Cnidaria	Anthozoa	Actiniaria	Stichodactylidae	* Heteractis *	* Heteractis magnifica *
Cnidaria	Anthozoa	Actiniaria	Stichodactylidae	* Stichodactyla *	* Stichodactyla mertensii *
Cnidaria	Anthozoa	Actiniaria			Actiniaria fam. indet. sp. 1
Cnidaria	Anthozoa	Actiniaria			Actiniaria fam. indet. sp. 2
Cnidaria	Anthozoa	Actiniaria			Actiniaria fam. indet. sp. 3
Cnidaria	Anthozoa	Actiniaria			Actiniaria fam. indet. sp. 6
Cnidaria	Anthozoa	Antipatharia	Antipatharia	* Antipathes *	*Antipathes* sp. indet.
Cnidaria	Anthozoa	Antipatharia	Leiopathidae	* Leiopathes *	*Leiopathes* sp. indet.
Cnidaria	Anthozoa	Antipatharia	Myriopathidae	* Cupressopathes *	*Cupressopathes* sp. indet.
Cnidaria	Anthozoa	Antipatharia	Myriopathidae	* Myriopathes *	*Myriopathes* sp. indet.
Cnidaria	Anthozoa	Antipatharia	Schizopathidae	* Bathypathes *	*Bathypathes* sp. indet.
Cnidaria	Anthozoa	Antipatharia	Stylopathidae	* Stylopathes *	*Stylopathes* sp. indet.
Cnidaria	Octocorallia	Alcyonacea	Acanthogorgiidae	* Muricella *	*Muricella* sp. indet.
Cnidaria	Octocorallia	Alcyonacea	Alcyoniidae	* Lobophytum *	*Lobophytum* sp. indet.
Cnidaria	Octocorallia	Alcyonacea	Alcyoniidae	* Paraminabea *	*Paraminabea* sp. indet.
Cnidaria	Octocorallia	Alcyonacea	Alcyoniidae	* Sarcophyton *	*Sarcophyton* sp. indet.
Cnidaria	Octocorallia	Alcyonacea	Alcyoniidae	* Sinularia *	*Sinularia* sp. indet.
Cnidaria	Octocorallia	Alcyonacea	Anthothelidae	* Solenocaulon *	*Solenocaulon* sp. indet.
Cnidaria	Octocorallia	Alcyonacea	Ellisellidae		Ellisellidae gen. indet. sp. 1
Cnidaria	Octocorallia	Alcyonacea	Ellisellidae		Ellisellidae gen. indet. sp. 2
Cnidaria	Octocorallia	Alcyonacea	Ellisellidae	* Dichotella *	*Dichotella* sp. indet.
Cnidaria	Octocorallia	Alcyonacea	Ellisellidae	* Ellisella *	*Ellisella* sp. indet.
Cnidaria	Octocorallia	Alcyonacea	Ellisellidae	* Nicella *	*Nicella* sp. indet.
Cnidaria	Octocorallia	Alcyonacea	Ellisellidae	* Verrucella *	*Verrucella* sp. indet.
Cnidaria	Octocorallia	Alcyonacea	Gorgoniidae	* Rumphella *	*Rumphella* sp. indet.
Cnidaria	Octocorallia	Alcyonacea	Isididae	* Isis *	*Isis* sp. indet.
Cnidaria	Octocorallia	Alcyonacea	Melithaeidae		Melithaeidae gen. indet. sp. 1
Cnidaria	Octocorallia	Alcyonacea	Melithaeidae		Melithaeidae gen. indet. sp. 2
Cnidaria	Octocorallia	Alcyonacea	Melithaeidae		Melithaeidae gen. indet. sp. 3
Cnidaria	Octocorallia	Alcyonacea	Nephtheidae	* Dendronephthya *	*Dendronephthya* sp. indet. 1
Cnidaria	Octocorallia	Alcyonacea	Nephtheidae	* Dendronephthya *	*Dendronephthya* sp. indet. 2
Cnidaria	Octocorallia	Alcyonacea	Nephtheidae	* Litophyton *	*Litophyton* sp. indet.
Cnidaria	Octocorallia	Alcyonacea	Nephtheidae	* Scleronephthya *	*Scleronephthya* sp. indet.
Cnidaria	Octocorallia	Alcyonacea	Nidaliidae		Nidaliidae gen. indet. sp.
Cnidaria	Octocorallia	Alcyonacea	Plexauridae		Plexauridae gen. indet. sp. 2
Cnidaria	Octocorallia	Alcyonacea	Plexauridae		Plexauridae gen. indet. sp. 4
Cnidaria	Octocorallia	Alcyonacea	Plexauridae		Plexauridae gen. indet. sp. 5
Cnidaria	Octocorallia	Alcyonacea	Plexauridae		Plexauridae gen. indet. sp. 6
Cnidaria	Octocorallia	Alcyonacea	Plexauridae		Plexauridae gen. indet. sp. 7
Cnidaria	Octocorallia	Alcyonacea	Plexauridae		Plexauridae gen. indet. sp. 8
Cnidaria	Octocorallia	Alcyonacea	Plexauridae		Plexauridae gen. indet. sp. 9
Cnidaria	Octocorallia	Alcyonacea	Plexauridae		Plexauridae gen. indet. sp. 11
Cnidaria	Octocorallia	Alcyonacea	Plexauridae		Plexauridae gen. indet. sp. 13
Cnidaria	Octocorallia	Alcyonacea	Plexauridae		Plexauridae gen. indet. sp. 14
Cnidaria	Octocorallia	Alcyonacea	Plexauridae	* Astrogorgia *	*Astrogorgia* sp. indet.
Cnidaria	Octocorallia	Alcyonacea	Plexauridae	* Echinogorgia *	*Echinogorgia* gen. inc.
Cnidaria	Octocorallia	Alcyonacea	Plexauridae	* Paracis *	*Paracis* gen. inc.
Cnidaria	Octocorallia	Alcyonacea	Plexauridae	* Trimuricea *	*Trimuricea* sp. indet.
Cnidaria	Octocorallia	Alcyonacea	Primnoidae	* Primnoa *	*Primnoa* sp. indet.
Cnidaria	Octocorallia	Alcyonacea	Primnoidae	* Narella *	*Narella* sp. indet.
Cnidaria	Octocorallia	Alcyonacea	Subergorgiidae	* Annella *	*Annella* sp. indet.
Cnidaria	Octocorallia	Alcyonacea	Tubiporidae	* Tubipora *	*Tubipora* sp. indet.
Cnidaria	Octocorallia	Alcyonacea	Xeniidae	* Xenia *	*Xenia* sp. indet.
Cnidaria	Octocorallia	Alcyonacea			Alcyonacea fam. indet. sp. 1
Cnidaria	Octocorallia	Alcyonacea			Alcyonacea fam. indet. sp. 2
Cnidaria	Octocorallia	Alcyonacea			Alcyonacea fam. indet. sp. 3
Cnidaria	Octocorallia	Alcyonacea			Alcyonacea fam. indet. sp. 4
Cnidaria	Octocorallia	Alcyonacea			Alcyonacea fam. indet. sp. 5
Cnidaria	Octocorallia	Helioporacea	Helioporidae	* Heliopora *	*Heliopora* sp. indet.
Cnidaria	Octocorallia	Scleractinia	Acroporidae	* Acropora *	*Acropora* sp. indet.
Cnidaria	Octocorallia	Scleractinia	Acroporidae	* Astreopora *	*Astreopora* sp. indet.
Cnidaria	Octocorallia	Scleractinia	Acroporidae	* Isopora *	*Isopora* sp. indet.
Cnidaria	Octocorallia	Scleractinia	Acroporidae	* Montipora *	*Montipora* sp. indet.
Cnidaria	Octocorallia	Scleractinia	Agariciidae	* Gardineroseris *	*Gardineroseris* sp. indet.
Cnidaria	Octocorallia	Scleractinia	Agariciidae	* Leptoseris *	*Leptoseris* sp. indet.
Cnidaria	Octocorallia	Scleractinia	Agariciidae	* Pavona *	*Pavona* sp. indet.
Cnidaria	Octocorallia	Scleractinia	Dendrophyllidae	* Tubastraea *	*Tubastraea* sp. indet.
Cnidaria	Octocorallia	Scleractinia	Dendrophyllidae	* Tubastraea *	* Tubastraea micranthus *
Cnidaria	Octocorallia	Scleractinia	Dendrophyllidae	* Turbinaria *	*Turbinaria* sp. indet.
Cnidaria	Octocorallia	Scleractinia	Euphylliidae	* Galaxea *	*Galaxea* sp. indet.
Cnidaria	Octocorallia	Scleractinia	Fungiidae		Fungiidae gen. indet. sp. 1
Cnidaria	Octocorallia	Scleractinia	Fungiidae		Fungiidae gen. indet. sp. 2
Cnidaria	Octocorallia	Scleractinia	Fungiidae	* Halomitra *	*Halomitra* sp. indet.
Cnidaria	Octocorallia	Scleractinia	Leptastreidae	* Leptastrea *	*Leptastrea* sp. indet.
Cnidaria	Octocorallia	Scleractinia	Lobophylliidae	* Echinophyllia *	*Echinophyllia* sp. indet.
Cnidaria	Octocorallia	Scleractinia	Lobophylliidae	* Lobophyllia *	*Lobophyllia* sp. indet.
Cnidaria	Octocorallia	Scleractinia	Merulinidae	* Dipsastraea *	*Dipsastraea* sp. indet.
Cnidaria	Octocorallia	Scleractinia	Merulinidae	* Echinopora *	*Echinopora* sp. indet.
Cnidaria	Octocorallia	Scleractinia	Merulinidae	* Favites *	*Favites* sp. indet.
Cnidaria	Octocorallia	Scleractinia	Merulinidae	* Goniastrea *	*Goniastrea* sp. indet.
Cnidaria	Octocorallia	Scleractinia	Merulinidae	* Hydnophora *	*Hydnophora* sp. indet.
Cnidaria	Octocorallia	Scleractinia	Merulinidae	* Pectinia *	*Pectinia* sp. indet.
Cnidaria	Octocorallia	Scleractinia	Merulinidae	* Platygyra *	*Platygyra* sp. indet.
Cnidaria	Octocorallia	Scleractinia	Merulinidae	* Oulophyllia *	*Oulophyllia* sp. indet.
Cnidaria	Octocorallia	Scleractinia	Plerogyridae	* Physogyra *	*Physogyra* sp. indet.
Cnidaria	Octocorallia	Scleractinia	Pocilloporidae	* Pocillopora *	*Pocillopora* sp. indet.
Cnidaria	Octocorallia	Scleractinia	Pocilloporidae	* Pocillopora *	* Pocillopora damicornis *
Cnidaria	Octocorallia	Scleractinia	Pocilloporidae	* Stylophora *	*Stylophora* sp. indet.
Cnidaria	Octocorallia	Scleractinia	Poritidae	* Porites *	*Porites* sp. indet.
Cnidaria	Octocorallia	Scleractinia	Poritidae	* Goniopora *	*Goniopora* sp. indet.
Cnidaria	Octocorallia	Scleractinia	Scleractinia incertae sedis	* Pachyseris *	*Pachyseris* sp. indet.
Cnidaria	Octocorallia	Zoantharia			Zoantharia stet.
Cnidaria	Hydrozoa				Hydrozoa stet.
Cnidaria	Hydrozoa	Anthoathecata	Milleporidae	* Millepora *	*Millepora* sp. indet.
Cnidaria	Hydrozoa	Anthoathecata	Solanderiidae	* Solanderia *	*Solanderia* sp. indet.
Cnidaria	Hydrozoa	Leptolida	Stylasteridae		Stylasteridae gen. indet. sp. 1
Cnidaria	Hydrozoa	Leptolida	Stylasteridae		Stylasteridae gen. indet. sp. 2
Cnidaria	Hydrozoa	Leptothecata	Thyroscyphidae	* Thyroscyphus *	*Thyroscyphus* sp. indet.
Ctenophora	Tentaculata	Platyctenida	Lyroctenidae	* Lyrocteis *	*Lyrocteis* sp. indet.
Echinodermata	Asteroidea				Asteroidea ord. indet. sp. 1
Echinodermata	Asteroidea				Asteroidea ord. indet. sp. 2
Echinodermata	Asteroidea	Forcipulatida	Asteriidae	* Coronaster *	* Coronaster volsellatus *
Echinodermata	Asteroidea	Forcipulatida	Asteriidae	* Coronaster *	*Coronaster* sp. indet.
Echinodermata	Asteroidea	Forcipulatida	Asteriidae	* Sclerasterias *	*Sclerasterias* sp. indet.
Echinodermata	Asteroidea	Paxillosida	Astropectinidae		Astropectinidae gen. indet. sp.
Echinodermata	Asteroidea	Valvatida	Asterinidae	* Nepanthia *	*Nepanthia* sp. indet.
Echinodermata	Asteroidea	Valvatida	Asterodiscididae	* Asterodiscides *	*Asterodiscides* sp. indet.
Echinodermata	Asteroidea	Valvatida	Goniasteridae	* Astroceramus *	*Astroceramus* sp. indet.
Echinodermata	Asteroidea	Valvatida	Goniasteridae	* Calliaster *	* Calliaster chaos *
Echinodermata	Asteroidea	Valvatida	Goniasteridae	* Fromia *	* Fromia nodosa *
Echinodermata	Asteroidea	Valvatida	Goniasteridae	* Peltaster *	* Peltaster cycloplax *
Echinodermata	Asteroidea	Valvatida	Goniasteridae	* Sphaeriodiscus *	*Sphaeriodiscus* sp. indet.
Echinodermata	Asteroidea	Valvatida	Ophidiasteridae		Ophidiasteridae gen. indet. sp.
Echinodermata	Asteroidea	Valvatida	Ophidiasteridae	* Heteronardoa *	* Heteronardoa diamantinae *
Echinodermata	Asteroidea	Valvatida	Ophidiasteridae	* Leiaster *	*Leiaster* sp. indet.
Echinodermata	Asteroidea	Valvatida	Oreasteridae		Oreasteridae sp. indet.
Echinodermata	Asteroidea	Valvatida	Oreasteridae	* Culcita *	* Culcita schmideliana *
Echinodermata	Asteroidea	Valvatida	Oreasteridae	* Halityle *	* Halityle regularis *
Echinodermata	Crinoidea				Crinoidea stet.
Echinodermata	Echinoidea	Arbacioida	Arbaciidae	* Coelopleurus *	*Coelopleurus* sp. indet.
Echinodermata	Echinoidea	Aspidodiadematoida	* Aspidodiadematidae *		Aspidodiadematidae gen. indet. sp.
Echinodermata	Echinoidea	Cidaroida			Cidaroida fam. indet. sp. 1
Echinodermata	Echinoidea	Cidaroida			Cidaroida fam. indet. sp. 2
Echinodermata	Echinoidea	Cidaroida	Cidaridae	* Acanthocidaris *	*Acanthocidaris* sp. indet.
Echinodermata	Echinoidea	Clypeasteroida	Clypeasteridae	* Clypeaster *	*Clypeaster* sp. indet.
Echinodermata	Echinoidea	Diadematoida	Diadematidae	* Echinothrix *	* Echinothrix diadema *
Echinodermata	Echinoidea	Micropygoida	Micropygidae	* Micropyga *	*Micropyga* sp. indet.
Echinodermata	Echinoidea	Pedinoida	Pedinidae	* Caenopedina *	*Caenopedina* sp. indet.
Echinodermata	Echinoidea	Spatangoida			Spatangoida fam. indet. sp.
Echinodermata	Holothuroidea	Holothuriida	Holothuriidae	* Bohadschia *	*Bohadschia* sp. indet.
Echinodermata	Holothuroidea	Holothuriida	Holothuriidae	* Holothuria *	Holothuria (Halodeima) atra
Echinodermata	Holothuroidea	Holothuriida	Holothuriidae	* Holothuria *	Holothuria (Halodeima) edulis
Echinodermata	Holothuroidea	Synallactida	Stichopodidae	* Stichopus *	*Stichopus* sp. indet.
Echinodermata	Holothuroidea	Synallactida	Stichopodidae	* Thelenota *	* Thelenota ananas *
Annelida	Annelida	Sabellida	Sabellidae		Sabellidae stet.
Mollusca	Mollusca	Cardiida	Cardiidae	* Tridacna *	*Tridacna* sp. indet.
Porifera	Calcarea	Clathrinida	Leucettidae	* Leucetta *	*Leucettachagosensis* sp. inc.
Porifera	Demospongiae	Axinellida	Axinellidae	* Axinella *	* Axinella weltnerii *
Porifera	Demospongiae	Clionaida	Clionaidae	* Spheciospongia *	*Spheciospongia* sp. indet. 1
Porifera	Demospongiae	Clionaida	Clionaidae	* Spheciospongia *	*Spheciospongia* sp. indet. 2
Porifera	Demospongiae	Clionaida	Clionaidae	* Spheciospongia *	*Spheciospongia* sp. indet. 3
Porifera	Demospongiae	Dendroceratida	Darwinellidae	* Aplysilla *	*Aplysilla* sp. indet.
Porifera	Demospongiae	Haplosclerida	Callyspongiidae	* Callyspongia *	*Callyspongia* sp. indet.
Porifera	Demospongiae	Haplosclerida	Chalinidae	* Haliclona *	*Haliclona* sp. indet. 1
Porifera	Demospongiae	Haplosclerida	Chalinidae	* Haliclona *	*Haliclona* sp. indet. 2
Porifera	Demospongiae	Haplosclerida	Chalinidae	* Haliclona *	*Haliclona* sp. indet. 3
Porifera	Demospongiae	Haplosclerida	Petrosiidae		Petrosiidae gen. indet. sp. 1
Porifera	Demospongiae	Haplosclerida	Petrosiidae		Petrosiidae gen. indet. sp. 2
Porifera	Demospongiae	Haplosclerida	Petrosiidae	Petrosia (Strongylophora)	Petrosia (Strongylophora) sp. indet.
Porifera	Demospongiae	Haplosclerida	Petrosiidae	* Xestospongia *	*Xestospongia* sp. indet.
Porifera	Demospongiae	Haplosclerida	Phloeodictyidae	* Oceanapia *	*Oceanapia* sp. indet.
Porifera	Demospongiae	Poecilosclerida	Iotrochotidae	* Iotrochota *	* Iotrochota nigra *
Porifera	Demospongiae	Poecilosclerida	Iotrochotidae	* Iotrochota *	* Iotrochota sinki *
Porifera	Demospongiae	Poecilosclerida	Microcionidae	* Calthria *	*Clathria* sp. indet.
Porifera	Demospongiae	Scopalinida	Scopalinidae	* Stylissa *	* Stylissa carteri *
Porifera	Demospongiae	Tetractinellida	Ancorinidae	* Stelletta *	*Stelletta* sp. indet.
Porifera	Demospongiae	Tetractinellida	Corallistidae	* Corallistes *	*Corallistes* sp. indet.
Porifera	Demospongiae	Tetractinellida	Pachastrellidae	* Pachastrella *	*Pachastrella* sp. indet.
Porifera	Demospongiae	Tetractinellida	Scleritodermidae	* Scleritoderma *	*Scleritoderma* sp. indet.
Porifera	Demospongiae	Tetractinellida	Tetillidae	* Tetilla *	*Tetilla* sp. indet.
Porifera	Demospongiae	Tetractinellida	Theonellidae	* Theonella *	Theonella cf. swinhoei
Porifera	Demospongiae	Tetractinellida	Theonellidae	* Theonella *	*Theonella* sp. indet.
Porifera	Demospongiae				Demospongiae order indet. sp. 1
Porifera	Demospongiae				Demospongiae order indet. sp. 2
Porifera	Demospongiae				Demospongiae order indet. sp. 3
Porifera	Demospongiae				Demospongiae order indet. sp. 4
Porifera	Hexactinellida	Amphidiscosida	Hyalonematidae	* Hyalonema *	*Hyalonema* sp. indet.
Porifera	Hexactinellida	Lyssacinosida	Euplectellidae	* Heterotella *	* Heterotella corbicula *
Porifera	Hexactinellida	Sceptrulophora	Tretodictyidae	* Sclerothamnus *	*Sclerothamnus* sp. indet.
Porifera	Homoscleromorpha	Homosclerophorida	Plakinidae	* Plakortis *	*Plakortis* sp. indet.
Porifera					Unknown lettuce-like green sponge
